# Comprehensive analysis of complement-associated molecular features in hepatocellular carcinoma: Complement-associated molecular features in hepatocellular carcinoma

**DOI:** 10.3724/abbs.2022097

**Published:** 2022-08-02

**Authors:** Run Huang, Guiqi Zhu, Xiutao Fu, Weiren Liu, Chenyang Tao, Jun Gao, Weifeng Qu, Yuan Fang, Xifei Jiang, Zhenbin Ding, Jian Zhou, Yinghong Shi, Jia Fan, Zheng Tang

**Affiliations:** 1 Department of Liver Surgery and Transplantation Liver Cancer Institute Zhongshan Hospital Fudan University and Key Laboratory of Carcinogenesis and Cancer Invasion of Ministry of Education Shanghai 200032 China; 2 Research Unit of Liver cancer Recurrence and Metastasis Chinese Academy of Medical Sciences Shanghai 200032 China.

**Keywords:** hepatocellular carcinoma, complement, immune infiltration, prognosis, drug response

## Abstract

The complement cascade plays a “complementing” role in human immunity. However, the potential roles of complement system in impacting molecular and clinical features of hepatocellular carcinoma (HCC) remain unclear. In this study, eleven public datasets are analyzed to compare the complement status between normal and cancerous samples based on 18 classical complement-associated genes. The complement scores are constructed to quantify complement signatures of individual tumors. HCC patients in the The Cancer Genome Atlas (TCGA) cohort are focused to perform systematical analyses between complement status and immune infiltration, miRNA expression, DNA methylation, clinicopathological features, and drug response. The results show that the complement scores in normal tissues are dramatically higher than those of tumor tissues. Tumor samples in the TCGA cohort are classified into complement score-low and score-high groups. Pathway analysis reveals that tumor-promoting pathways are typically inhibited in complement score-high group. This study also shows that tumor-killing immune cells, such as CD8 ^+^ T cells and natural killer cells are abundant and tumor-suppressing miRNAs are upregulated in complement score-high samples. In addition, we identify that complement scores are negatively correlated with certain clinical features, including pathological grade, clinical-stage, and portal vein invasion. Moreover, various molecular features together with complement scores are found to be correlated with response to anti-cancer drugs. This study provides a comprehensive and multidimensional analysis conducive to understanding the role of complement in cancer.

## Introduction

Hepatocellular carcinoma (HCC), a severe malignant disease originated from the hepatocytes, ranks fourth in cancer-related death and is the sixth most common cancer worldwide [ 1, 2] . Due to the high heterogeneity of liver cancer and lack of effective systemic treatment, the prognosis of HCC patients is rather poor, especially for advanced cancer patients [3]. At present, systemic therapies such as targeted therapy and immunotherapy are recommended for advanced HCC patients. However, there is a lack of an effective evaluation system to assess the sensitivity of HCC to targeted therapy and to predict the prognosis of HCC patients.

The liver is the primary organ that synthesizes most complement proteins, and the complement system plays an indispensable role in innate and adaptive immunity. The complement cascade can be initiated by three distinct pathways (classical, lectin or alternative), which converge at the complement C3 and C5, and end up in the formation of membrane attack complex (MAC) that attacks potential pathogens and aberrant cells [4]. In the process of the complement cascade, a series of effector molecules are generated, synergizing and ‘complementing’ the immune system to clear immunocomplex, lyse intruded microorganisms, and promote inflammation [5].

In recent years, there has seen a surging interest in this ancient system with regards to its paradoxical role in cancer initiation and progression [ 6, 7] . The most important mechanism of complement in killing cancer cells is complement-dependent cytotoxicity (CDC). In monoclonal antibody (mAb)-based cancer therapy, large amount of mAbs seed on the surface antigens of cancer cells, forming the immunocomplexes that bind to the C1q, which turns on the classic pathway of the complement cascade [8]. Finally, the MACs are formed on the surface of the cancer cells, permeabilizing the cells and increasing the intracellular level of Ca ^2+^, which exerts cytotoxic effects on cancer cells [ 9, 10] . Additionally, in a cervical cancer model, complement activation was reported to manipulate the function of endothelial cells, which enabled successful homing of effector T cells to the tumor microenvironment [11]. Another study revealed that radiotherapy-mediated cell death could induce local complement activation and promote the production of anaphylatoxins (C3a and C5a) that are potent pro-inflammatory molecules [12]. And radiotherapy-induced dendritic cell (DC) activation and CD8 ^+^ T cell activation both depended on the accumulated anaphylatoxins that are favorable to tumor-specific immunity [12]. On the other hand, some studies have reported that anaphylatoxins are able to tune the tumor immune microenvironment in a manner of recruiting immunosuppressing cells such as myeloid-derived suppressor cells (MDSCs), M2 macrophages, and regulatory T cells, and inducing the production of immunosuppressing cytokines such as transforming growth factor-β (TGF-β) and IL-10 [ 13– 16] . Meanwhile, cancer cells can escape the lysis effect of MACs due to insufficient MACs formation and abundant complement control proteins (CCPs) expression on cancer cells [17]. Consequently, complement activation is a delicately regulated double sword dependent upon the composition of tumor microenvironment and type of cancers [6].

In the context of HCC, little investigation has explored the relationship between the complement activation and the malignant behavior of liver cancer cells, and the sensitivity of immunotherapy and targeted therapy. In this study, eleven datasets containing transcriptomic information of cancerous tissue and normal tissue were intensively analyzed, and the process of complement activation was found to be significantly inhibited. According to the expression signatures of 18 complement activation-related genes, we have first established the complement score for each sample. A comprehensive analysis was performed to compare the immune infiltration, miRNA expression, clinical characteristics, and drug sensitivity between the complement score-high and score-low cohorts. Moreover, a LASSO-COX regression model was established to predict the prognosis for HCC patients.

## Materials and Methods

### Data acquirement and differential expressed genes (DEGs) analysis

The flow chart of this study was illustrated in Supplementary Figure S1. Transcriptomic data (log2 intensity) of eleven datasets, including TCGA-LIHC (liver hepatocellular carcinoma in The Cancer Genome Atlas), ICGC-LIRI-JP (liver cancer from RIKEN, Japan in the database of International Cancer Genome Consortium), GSE22058, GSE46444, GSE54236, GSE63898, GSE64041, GSE76427, GSE36376, GSE14520, and GSE10143, were downloaded from the website of HCCDB ( http://lifeome.net/database/hccdb) [18]. The sample size of each dataset was listed in Supplementary Table S1. All of these data were normalized using the function normalizeBetweenArrays in “limma” R package before performing the downstream analysis ( Supplementary Figure S2). DEGs analyses between cancerous tissues and normal tissues were performed with “limma” package, obtaining a gene list with logFC in each dataset. These eleven logFC lists were integrated using the “RobustRankAggreg” (RRA) R package to acquire the integrated upregulated and downregulated genes for further analysis. The R packages of “clusterProfiler” and “enrichplot” were used to run the GSEA analysis of DEGs to obtain the results of GO and KEGG pathway enrichment. In these analyses, the samples of normal tissues served as the control. In addition, molecular data in the TCGA-LIHC cohort, including miRNA expression, DNA methylation, and clinical data, were downloaded using the R tool of TCGA Assembler [19].

### Classification of complement status

To depict the complement signature of each sample, 18 complement activation-related genes (CFB, C1QA, C1QB, C1QC, C1R, C1S, C2, C3, C4A, C5, C6, C7, C8A, C8B, C8G, C9, MASP1, and MASP2) were selected to construct the complement score using the method of single sample gene set enrichment analysis (ssGSEA) in “GSVA” package [20]. These genes are the most important in the activation of the complement cascade. The Wilcoxon test was used to assess the statistical difference of complement scores between tumor and normal tissues. To classify complement status, unsupervised hierarchical k-means clustering was used to cluster tumor samples in TCGA-LIHC based on the mRNA expression, and three clusters were obtained. Cluster 1, cluster 2, and cluster 3 were defined as complement score-low, complement score-intermediate, and complement score-high, respectively. To avoid the potential confounding factors, samples of the complement score-intermediate group were excluded before further analysis as previously described [21].

### Estimation of immune infiltration

To quantify the immune infiltration in the microenvironment of each sample, ssGSEA was applied to calculate the immune score based on the cell markers from a previous study that characterized the signatures of 28 types of human immune cells [22]. These included immune cells in innate immunity, such as natural killer (NK) cells, dendritic cells, macrophages, and mast cells, and immune cells in adaptive immunity, such as B cells, CD4 ^+^ T cells, and CD8 ^+^ T cells. The enrichment scores were used to represent the relative abundance of individual immune cells [23].

### miRNA and DNA methylation analysis

The downloaded RPM data in the TCGA cohort were transformed using the formula log2(RPM+1). Then the differentially expressed miRNAs between complement score-low and score-high groups were analyzed using the “limma” package. To predict the potential upstream miRNA of complement activation-related genes, intersected miRNAs from three miRNA databases including TargetScan, miRDB, and miRWalk were acquired, and the statistical difference of these miRNAs was assessed.

Furthermore, the DNA methylation levels at various regions, including TSS200 (200 nucleotides upstream of the transcription start site), TSS1500, gene body, first exon, 3′UTR (untranslated region), and 5′UTR of complement genes were also analyzed. The correlation tests between complement score and the expression of DNA methyltransferases were performed using the Spearman method.

### Prognosis model construction and validation

To discover the prognostic complement genes, a total of 351 patients in the TCGA-LIHC cohort were randomly divided into training set and validation set with a ratio of 7:3. The prognostic gene signature was constructed using the LASSO regression, and the optimal coefficients for significant genes were determined through 10-fold cross validation using the “glmnet” R package. A six-gene complement signature ( *C1QB*, *C1S*, *C2*, *C3*, *C5*, and *MASP2*) was established, and the risk score for each patient was calculated. Along with the risk score, clinical parameters including gender, race, tumor size, lymph nodes, metastasis, the AJCC stage, and pathological grade were analyzed by univariable and multivariate COX regression analysis. To confirm the potency of the prognosis model, the risk scores in the datasets of ICGC and GSE14520 were calculated using the above mentioned method, and the survival analysis based on the risk score was performed using the Log-rank test. Moreover, Wilcoxon or Kruskal−Wallis tests were performed to compare the complement score and risk score based on differential clinical characteristics in TCGA-LIHC and ICGC cohorts.

### Analysis of drug response

To analyze the relationship between drug response and complement activation, the imputed drug response to 138 anti-cancer drugs in TCGA patient samples was downloaded from a previous study [24]. Spearman correlation was performed to calculate the correlation between imputed drug response and complement score, and the relationship between imputed drug response and complement-associated miRNA expression, mRNA expression, and DNA methylation was also analyzed. To consolidate the complement activation-related drug response, the drug sensitivity of 1020 cancer cell lines and their gene expression profiles were downloaded from the website of GDSC (Genomics of Drug Sensitivity in Cancer; https://www.cancerrxgene.org/downloads/bulk_download). The complement score of each cancer cell line was computed, and the Spearman correlation between the natural log of the fitted IC _50_ and complement score was calculated.

### Immunohistochemistry assay

For IHC staining, the TMAs containing 118 tumor sections and 118 adjacent normal tissue sections were obtained from Zhongshan Hospital, Fudan University (Shanghai, China). Immunohistochemistry (IHC) staining was performed as previously described [25], using marker antibodies anti-human C7 (ab126786; Abcam, Cambridge, UK), and anti-human C9 (ab168345; Abcam), and scanned using Pannoramic MIDI (3DHISTECH Ltd. Budapest, Hungary). In order to quantify the staining intensity, the scanned image was analyzed with the image analysis system based on artificial intelligence, which calculated the modified Histochemistry-scores as follows: H-scores=(percent of weak staining×1)+(percent of moderate staining×2)+(percent of strong staining×3) [26]. Finally, the H-scores ranging from 0 to 300 were obtained.

### Mouse models and therapeutic protocols

All the experimental procedures rigorously complied with the Guide for the guidance of Animal Ethics Committee of Fudan University. Murine Hepa 1-6 cells (2×10 ^6^; National Collection of Authenticated Cell Cultures, Shanghai, China) were resuspended in 100 μL PBS and injected subcutaneously in the right flanks of 6-week-old C57BL/6 mice (Shanghai Jihui Laboratory Animal Care Co., Ltd., Shanghai, China). One day before the inoculation of cancer cells, mice were treated with cobra venom factor (CVF, 0.2 mg/kg, Catalog #ZX0006; Rongmin Biotechnology Center, Shanghai, China), and continued every other day until the end of the experiment. For the inhibitor administration, tumor-bearing mice were treated with sorafenib (10 mg/kg, T0093L; TargetMol, Wellesley Hills, USA), lenvatinib (10 mg/kg, T0520; TargetMol), and BI-2536 (20 mg/kg, T6173; TargetMol) at days 8, 10, and 12 after cell inoculation. Tumor volumes were measured every other day, and the tumor volumes were calculated using the formula: volume=0.5×width× width×length.

### Statistical analysis

The software R 4.0.3 was used to perform all the data processing and analysis. Continuous variables were analyzed using Wilcoxon or Kruskal−Wallis tests as indicated in the figure legends. The heatmaps in this study were drawn using the R package “pheatmap”. Univariate and multivariate survival analyses were conducted with the R package “survimer”, and the Kaplan-Meier survival curves with risk tables were drawn. All the graphs in this study were drawn using the R packages “ggplot2” or “ggpubr”. A *P*-value less than 0.05 was considered statistically significant for most statistical analyses, and more stringent criteria for DEGs analysis and correlation analysis were indicated in the figure legends.

## Results

### Inhibited complement activation in HCC tissues

**Figure 1:**
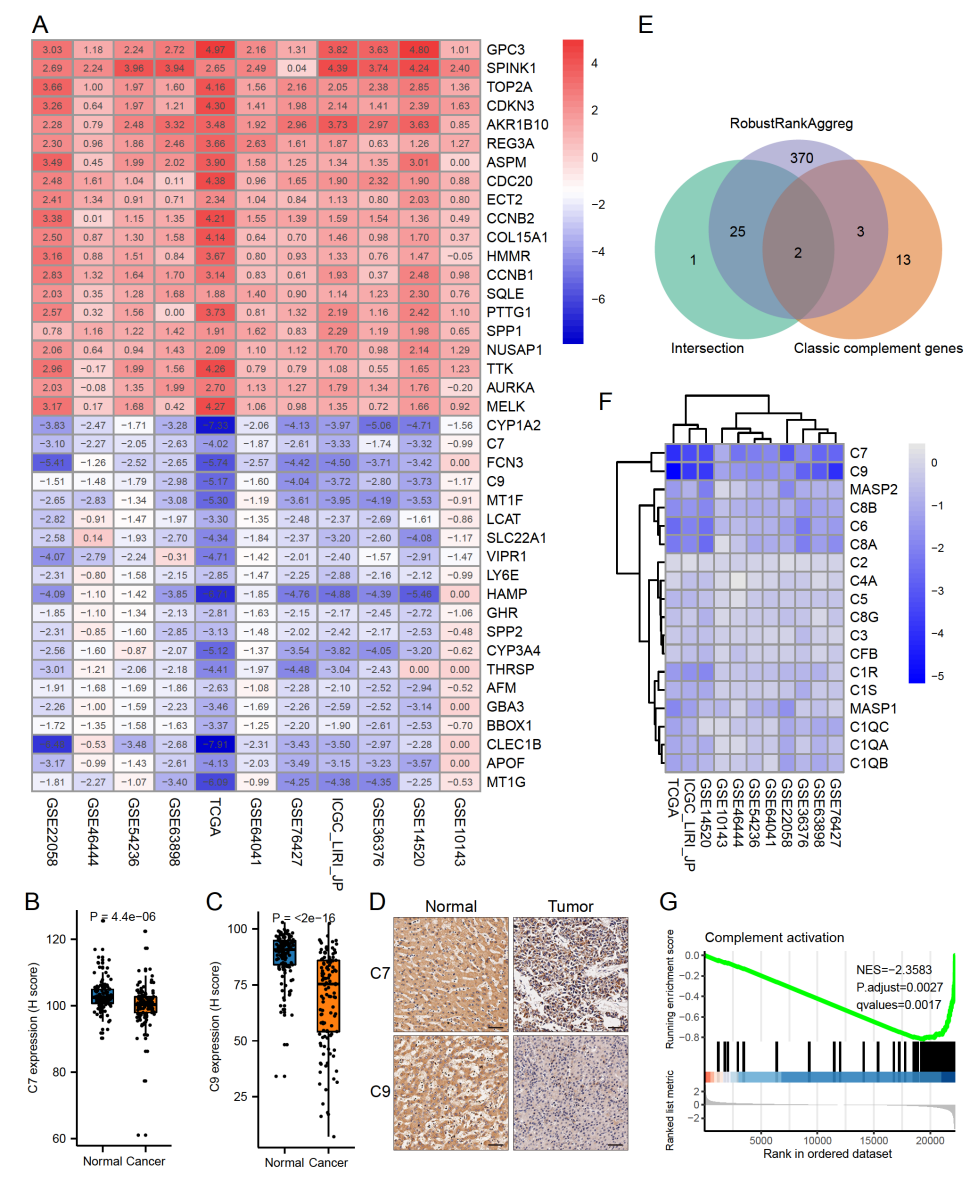
Figure 1 Inhibited complement cascade in liver cancer tissues (A) The heatmap of top 20 upregulated (red) and downregulated (blue) DEGs identified by the RRA method in each dataset. Normal tissues served as controls, and the number in each box refers to the fold change of gene expression. (B,C) Comparison of histochemistry-scores (H-scores) of C7 and C9 between normal and cancerous tissues in TMA. (D) Typical IHC plots representing the expressions of C7 and C9. Scale bar= 50 μm. (E) Venn diagram illustrating the intersection between classic complement activation-related genes and significant DEGs in RRA analysis and a simple intersection of significant DEGs in eleven datasets. (F) The heatmap illustrating the fold change of eighteen complement genes after differential analysis using the “limma” package. (G) GSEA plot of the process of complement activation showing the negative enrichment of complement genes.

To identify significant DEGs between cancerous and normal tissues in liver cancer, gene expression profiles of eleven datasets were integrated and analyzed using the method of robust rank aggregation (RRA). After integrated analysis, 400 DEGs were acquired, with 108 upregulated and 292 downregulated genes ( *P*<0.05, logFC>1; Supplementary Table S2). In the top 20 overlapping upregulated and downregulated DEGs, the expressions of three complement-related genes *FCN3*, *C7*, and *C9* were found to be significantly downregulated compared with non-cancerous tissues ( Figure 1A, and Supplementary Figure S3A,B). Consistently, the expressions of C7 and C9 in tumor tissues were confirmed to be downregulated in tissue microarray (TMA) compared with adjacent normal tissues ( Figure 1B–D). Moreover, survival analyses also revealed the trends that higher expressions of C7 and C9 might be favorable to better survival for the liver cancer patients ( Supplementary Figure S3C,D). FCN3 (Ficolin-3) might play an important role in the activation of the lectin complement pathway [27], and C7 and C9 consist of the core components of the MACs that lyse enthetic microorganisms and aberrant cells. And the Venn diagram also illustrated that the expressions of five complement genes were significantly decreased in the tumor tissues ( Figure 1E). To further consolidate the complement status in tumor tissues, the expression profiles of 18 classic genes in complement activation were visualized based on the logFC in the DEGs analysis, and almost all these complement genes were found to be significantly inhibited ( Figure 1F). Moreover, GSEA and KEGG pathway enrichment analyses were performed based on the integrated gene list with logFC generated in the RRA analysis. The GSEA analysis also revealed that the biological process of complement activation was dramatically suppressed in the cancerous tissues ( Figure 1G, and Supplementary Figure S4A–D). Meanwhile, other pathways in innate immunity, such as JAK-STAT signaling pathway, TNF signaling pathway, NOD-like receptor signaling pathway, and TOLL-like receptor signaling pathway, were all significantly prohibited, demonstrating the immunosuppression status in HCC tissues ( Supplementary Figure S4E–H). In KEGG enriched pathways, the complement cascade was typically suppressed, while pathways in cancer progression, such as DNA replication, spliceosome, and cell cycle, were activated compared with normal tissues ( Supplementary Figure S3E). These results indicated that the process of complement activation in HCC tissues was in an inhibited condition.

### Classification of complement status by a complement-associated signature

**Figure 2:**
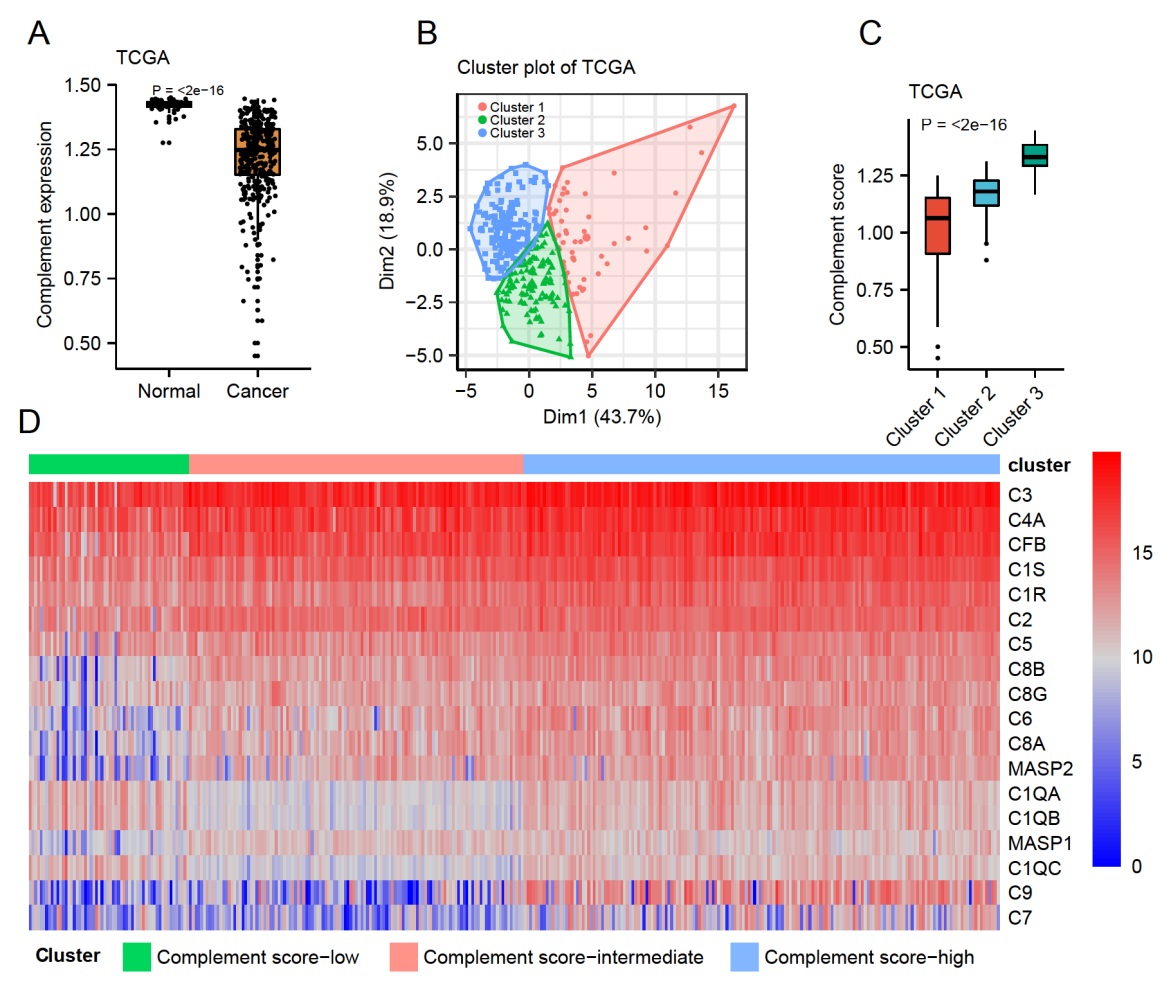
Figure 2 Clustering of tumor samples in TCGA-LIHC (A) Comparison of complement scores between normal and cancer tissues. (B) Three clusters were classified by the k-means clustering method. (C) Distribution of complement scores of three distinct clusters. (D) Heatmap of single complement gene expression based on three clusters. Complement score-low indicates the cluster 1 ( n=58), complement score-intermediate indicates the cluster 2 ( n=121), and complement score-high indicates the cluster 3 ( n=172).

To delineate the complement status of each sample, we focused on an eighteen-gene expression signature in classic complement activation, and the complement scores for both cancer and non-cancer tissues were calculated (see Materials and Methods). Tumor tissues showed significantly lower complement scores than normal tissues across eleven datasets, suggesting the robustness of the eighteen-gene signature and validating the suppressed status of complement activation in liver tumor tissues ( Figure 2A, and Supplementary Figure S5). To classify the complement status of tumor samples in TCGA-LIHC, unsupervised clustering was employed based on the eighteen-gene expression signature of complement activation ( Figure 2B). A total of 351 TCGA-LIHC samples were classified into three distinct clusters, showing distinct complement score distribution that was consistent with the expression level of each complement protein ( Figure 2C,D). The three clusters were defined as complement score-low cluster, complement score-intermediate cluster, and complement score-high cluster according to the levels of complement scores. In the complement score-high group, most complement gene expression was higher than that of complement score-low samples ( Figure 2D).

### Complement activation in regulating the immune status of tumor tissues

**Figure 3:**
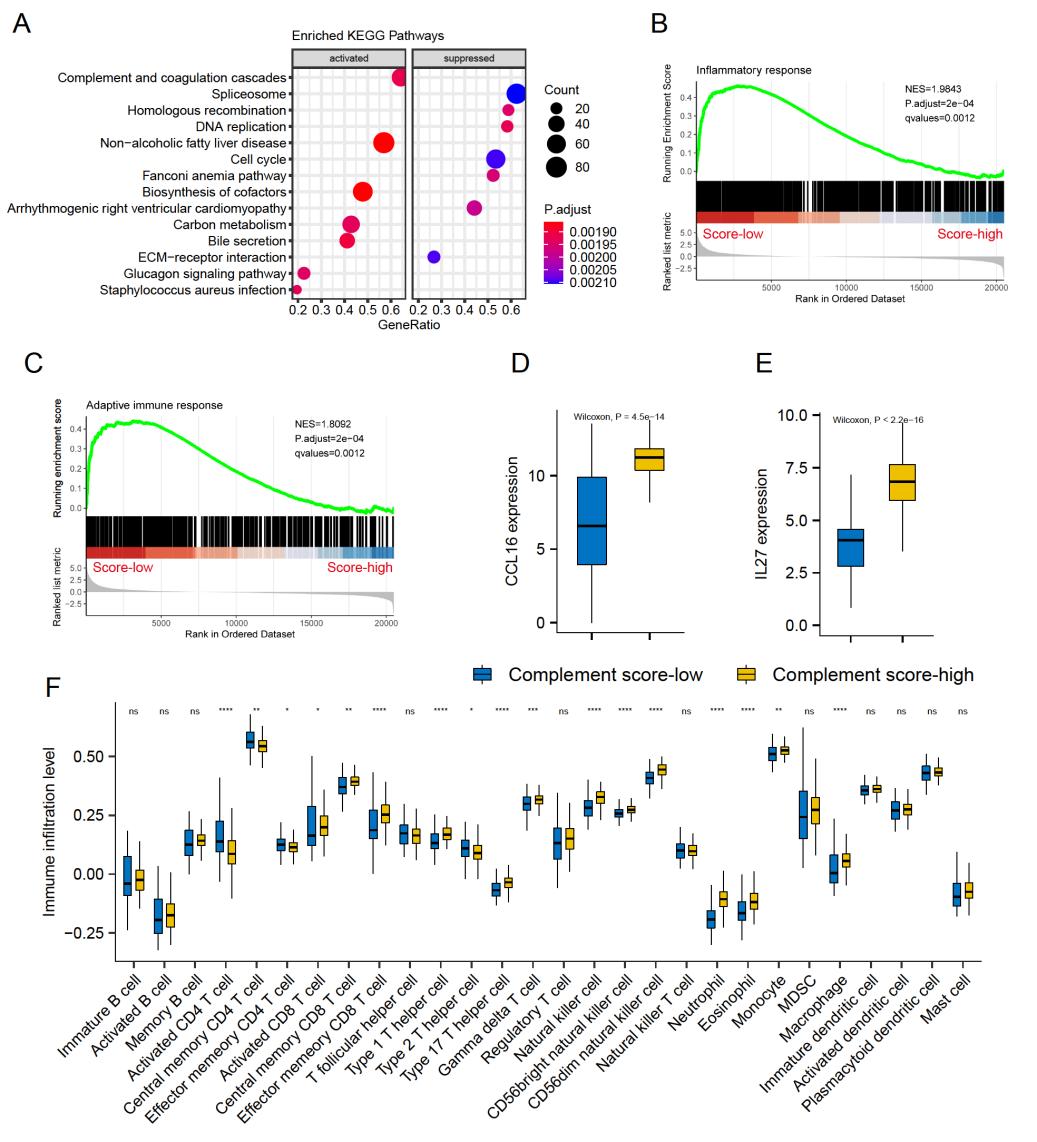
Figure 3 Complement activation regulates the immune status and immune infiltration of tumor tissues (A) KEGG enriched pathways between complement score-high and score-low groups. (B,C) GSEA plots showing that the complement score-high group promoted the inflammatory response and adaptive immunity. (D,E) Boxplots indicating the significantly higher expressions of CCL16 and IL-27 in complement score-high group. (F) The immune infiltration levels of 28 types of immune cells in the TCGA-LIHC cohort. The blue box refers to the complement score-low group, and the yellow box refers to the complement score-high group. * P<0.05; ** P<0.01; *** P<0.001; **** P<0.0001; ns, not significant.

To characterize the expression profiles of these complement-associated groups, DEGs analysis was performed between the complement score-high and complement score-low samples (see Materials and Methods). Distinct expression patterns were observed with 1193 significant DEGs between these two clusters (P.adjust<0.05, |logFC|>1.5; Supplementary Figure S6A,B). Compared with the complement score-low cohort, the pathway of complement and coagulation cascades was remarkably activated in the KEGG enrichment analysis expectedly ( Figure 3A). Moreover, the cancer-promoting pathways, including spliceosome, DNA replication, and cell cycle, were typically inhibited in complement score-high group, suggesting that complement activation was negatively correlated with the malignant biological features of cancer cells ( Figure 3A, and Supplementary Table S3).

Complement proteins constitute an indispensable part of innate and adaptive immunity, which prompted us to further explore the relationship between complement activation and immune cell infiltration. The infiltration of immune cells was quantified using the method of ssGSEA based on the cell markers from a previously published paper [22]. A remarkable decrease of both innate and adaptive immune cells was found in tumor samples of diverse datasets, strongly suggesting the immunosuppressive status of liver tumor tissues ( Supplementary Figure S7). According to the GO analysis using the GSEA method, complement cascade was found to be positively correlated with inflammatory response and adaptive immunity ( Figure 3B,C and Supplementary Table S4). And a dramatically higher expression of inflammation-promoting chemokines and cytokines, such as CCL16, IL-27, CCL15, and CXCL2, was observed in complement score-high cluster ( Figure 3D,E and Supplementary Figure S6C,D). To characterize the tumor immune microenvironment in distinct complement clusters, immune scores for various immune cells were calculated. Compared with the complement score-low cohort, although fewer CD4 ^+^ T cells infiltrated in the tumor microenvironment, the tumor-killing cells including CD8 ^+^ T cells and NK cells were observed to be abundant in complement score-high samples ( Figure 3F). Moreover, complement score-high samples were remarkably enriched in innate immune cells, such as neutrophils, eosinophils, monocytes, and macrophages, consistent with the augmented inflammation status ( Figure 3F).

### DNA methylation regulates the expression of complement-associated proteins

**Figure 4:**
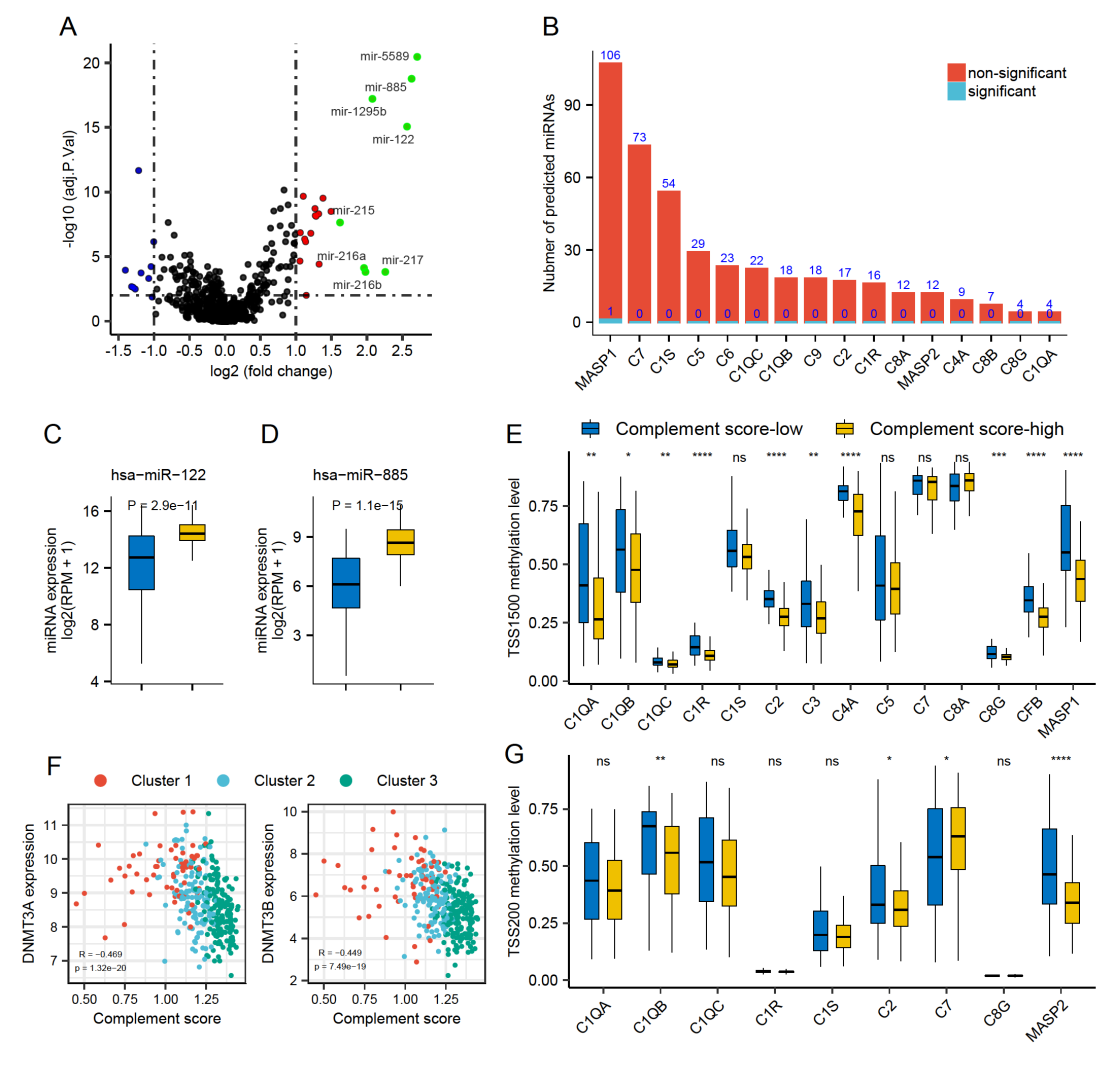
Figure 4 The relationship between complement activation and miRNA expression and methylation (A) The volcano plot showing the differentially expressed miRNAs between complement score-high and score-low groups. The red, blue, and green dots indicate the significant miRNAs compared with the complement score-low cohort (P.adjusted<0.05, |logFC|>1). The green dots refer to miRNAs highly expressed more than 2 times. (B) The number of predicted common miRNAs for complement-associated genes in three datasets (TargetScan, miRD(B) and miRWalk). Red bars indicate the numbers of not significantly differentially expressed miRNAs between two clusters, and the blue bars indicate the number of significant miRNAs. (C,D) Boxplots indicating significantly higher expression of has-miR-122 and has-miR-885 in complement score-high group. (E,G) Comparisons of DNA methylation levels of the promoter regions of TSS1500 and TSS200. (F) Scatter plots showing the correlation between the complement scores and the expression of DNA methyltransferases DNMT3A and DNMT3B. The blue box refers to the complement score-low group, and the yellow box refers to the complement score-high group. * P<0.05; ** P<0.01; *** P<0.001; **** P<0.0001; ns, not significant.

To investigate the regulatory mechanisms of complement protein expression, the miRNA expression profiles between complement score-low and score-high groups were analyzed, revealing 32 differentially expressed miRNAs (P.adjust<0.05, |logFC|>1; Figure 4A and Supplementary Table S5). However, few predicted miRNAs targeting the 18 complement-associated proteins showed a significant difference ( Figure 4B and Supplementary Table S6). In the differentially expressed miRNAs, tumor suppressor miRNAs, such as hsa-miR-122, and hsa-miR-885, were typically upregulated in the complement score-high group ( Figure 4C,D). These results suggested that differences in miRNA expression appeared to be the consequences of distinct complement activation, instead of upstream regulators of the complement cascade.

Subsequently, we sought to explore the influence of DNA methylation on the protein expression of the complement cascade. DNA methylation usually occurs at the CpG islands around the promoter region under the catalyses of DNA methyltransferases (DNMTs), impeding the binding of transcription factors to the promoter and inhibiting gene expression [ 28, 29] . In the analysis of promoter regions TSS200 and TSS1500, remarkable decreased methylation levels of most complement proteins were observed in the complement score-high group, consistent with the higher gene expression level ( Figure 4E,G). Meanwhile, lower methylation levels of certain complement proteins were also found at certain regions of 5′UTR, 3′UTR, 1stExon, and gene body ( Supplementary Figure S8A–D). Moreover, Spearman’s correlation also revealed negative correlations between the DNMTs and complement scores, indicating the importance of DNMTs in the epigenetic modification of complement-associated proteins ( Figure 4F and Supplementary Figure S8E). Together, these results showed that the expression of complement genes was regulated in a DNA methylation-dependent manner.

### Construction and validation of prognostic signatures for complement proteins

**Figure 5:**
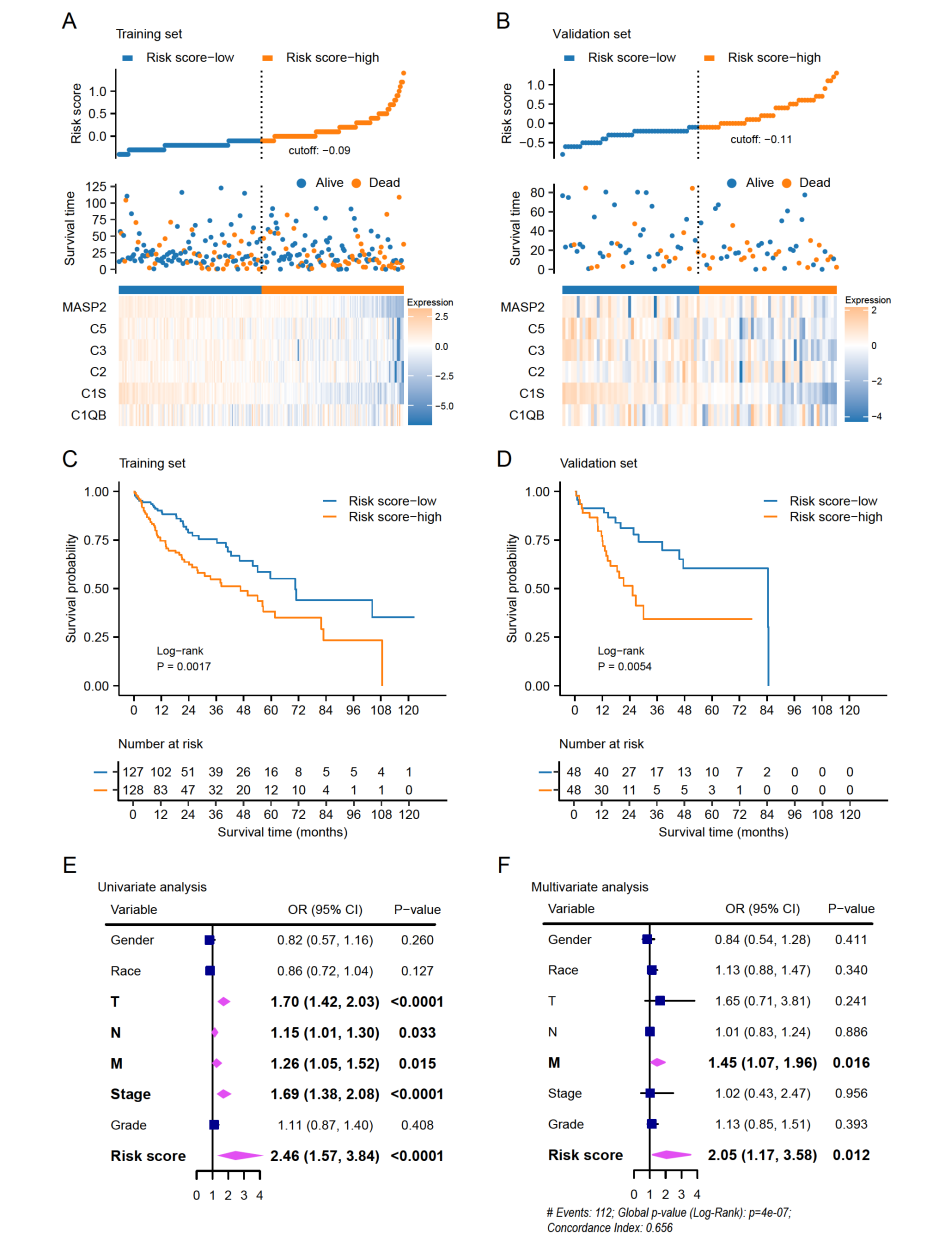
Figure 5 Construction and validation of prognostic signatures of complement-associated genes (A,B) Distribution of risk scores, survival status, and expression heatmap of six complement genes selected from the LASSO algorithm in the TCGA-LIHC cohort. (C,D) Kaplan-Meier survival curves based on the risk scores in the training and validation cohorts. The cutoff value is the median risk score. (E,F) Univariate and multivariate COX analyses of the TCGA-LIHC cohort.

To assess the prognostic role of complement status in HCC, a total of 351 patients in the TCGA-LIHC were randomly divided into the training cohort (n=255) and validation cohort (n=96) at a 7:3 ratio. No statistical significance was observed among the training and validation cohorts ( Supplementary Table S7). To predict the clinical prognosis more precisely, LASSO regression was performed based on the expression of 18 complement genes inure the training cohort. Six complement-associated genes ( *C1QB*, *C1S*, *C2*, *C3*, *C5*, and *MASP2*) were selected as significant candidates ( Supplementary Figure S9A,B). The risk score was constructed using the coefficients obtained from the LASSO regression process: risk score=2.498–(0.0175×C3 expression)–(0.0760×C1S expression)–(0.0240×C2 expression)–(0.0199×C5 expression)–(0.0812×MASP2 expression)+(0.0085×C1QB expression). This formula was applied in the training and validation cohorts, and patients were divided into the risk score-low group and risk score-high group according to the median risk score. The distribution of risk scores and survival status was illustrated in Figure 5A,B. And the heatmap revealed that the complement genes were highly expressed in the risk score-low cohort ( Figure 5A,B). The receiver operating characteristic (ROC) curve analyses showed relatively stable prognostic accuracy in the training and validation cohorts ( Supplementary Figure S9C). Survival analyses demonstrated that the overall survival (OS) in the risk score-low cohort was significantly superior to that in the score-high cohort ( Figure 5C,D). To confirm the potency of this prognosis model, the datasets of ICGC and GSE14520 were employed as external validation. The risk scores were calculated using the constructed formula, and survival analysis also showed a favorable prognosis in the risk score-low cohort in both datasets ( Supplementary Figure S9D,E). Moreover, univariate and multivariate COX analyses were conducted to further confirm the prognosis value of the risk score in the TCGA-LIHC cohort. Univariate analysis showed that T stage ( *P*<0.0001), N stage ( *P*=0.033), M stage ( *P*=0.015), TNM stage ( *P*<0.0001), and risk score ( *P*<0.0001) were significantly associated with OS ( Figure 5E). Meanwhile, multivariate analysis revealed that risk score could serve as an independent prognostic factor for HCC patients with a C-index score of 0.656 for this model ( Figure 5F). Together, these results demonstrated that the six-gene-based signature was potent in predicting the prognosis for HCC patients.

### Risk scores correlated with complement scores and clinical features

**Figure 6:**
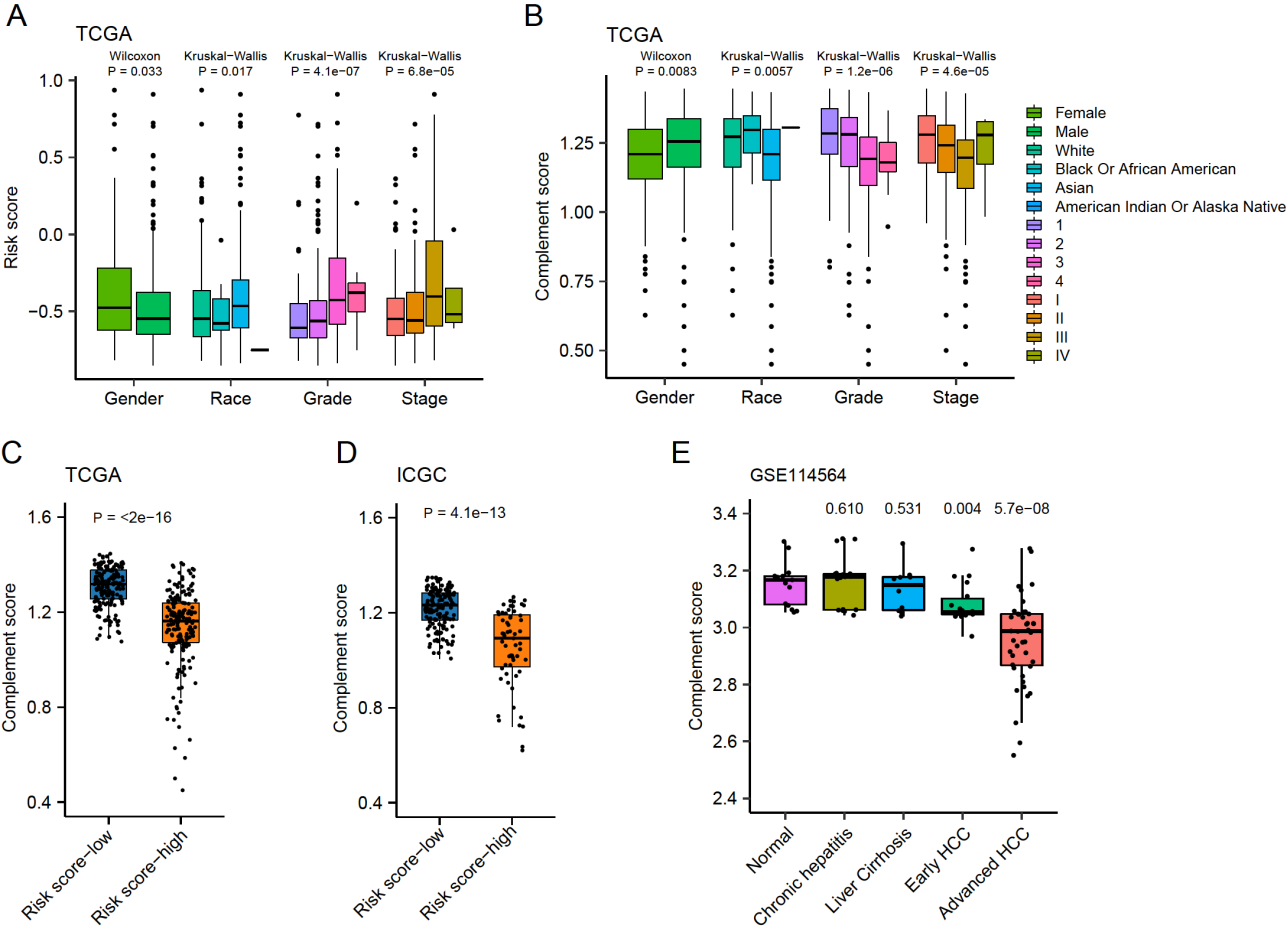
Figure 6 Distribution of risk scores and complement scores based on clinical features (A,B) Boxplots showing the distribution of the risk scores and complement scores in the TCGA-LIHC cohort. (C,D) Distribution of the complement scores in risk score-low and score-high groups. (E) Differences in complement scores of distinct sample types in GSE114564.

To evaluate the clinical values of the risk scores and complement scores, the relationship between the clinical features and these scores was analyzed using TCGA-LIHC and ICGC datasets, which contained relatively complete clinicopathologic features. Little distribution difference of risk scores and complement scores was found in gender, race, virus contamination, and liver fibrosis ( Figure 6A,B and Supplementary Figure S10). The pathological grade tended to be higher as risk scores increased, while the complement scores were negatively correlated with this feature ( Figure 6C,D). Similarly, the clinical-stage also seemed to have a positive correlation with the risk scores and a negative correlation with complement scores ( Figure 6A,B, and Supplementary Figure S10). Typically, compared with patients without portal vein invasion, the risk scores in patients with TYPE II, TYPE III, and TYPE IV portal vein invasion were remarkably increased ( Supplementary Figure S10A). And the complement scores of patients with portal vein invasion were significantly lower than those without portal vein invasion ( Supplementary Figure S10B). Consistent with these findings, risk scores were negatively correlated with complement scores in both TCGA-LIHC and ICGC datasets ( Figure 6C,D). Moreover, in the dataset of GSE114564, which consists of normal tissue, early HCC, and advanced HCC, complement scores were found to be negatively correlated with the progression of liver cancer ( Figure 6E). These results suggested that the risk score was associated with pathological grade, stage, portal vein invasion, and cancer progression.

### Integrative analysis of complement-associated molecular features on drug response

**Figure 7:**
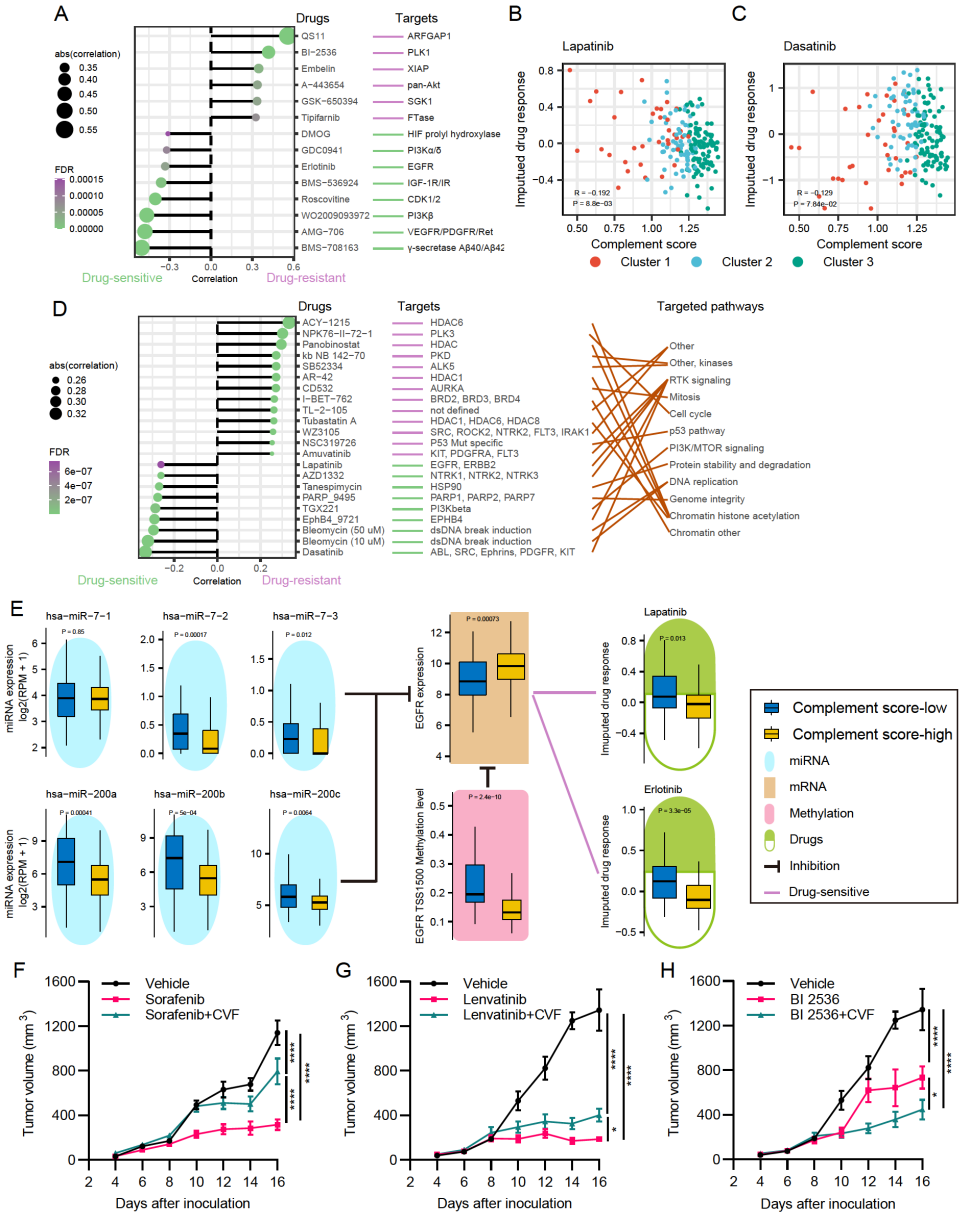
Figure 7 Effects of complement-associated features on drug response (A) Spearman’s correlations between complement scores and imputed drug response in the TCGA cohort (FDR<0.05 and the absolute value of correlation>0.2). The potential targets of these drugs are illustrated on the right side. A positive correlation indicates drug-resistant, and a negative correlation indicates drug-sensitive. (B,C) Scatter plots between complement scores and imputed drug response of EGFR-targeting drugs lapatinib and dasatinib in the TCGA cohort. (D) Spearman’s correlations between complement scores and the natural logarithm-transformed IC 50 in GDSC (FDR<0.05 and the absolute value of correlation>0.25). The potential targeted molecules and pathways of these drugs are illustrated on the right side. (E) EGFR is upregulated in the complement score-high group, and negatively correlated with the methylation level at the region of TSS1500. Six miRNAs are associated with the downregulation of EGFR expression. The six miRNA and the hypomethylation of TSS1500 are positively correlated with the imputed drug response of lapatinib and erlotinib (drug-resistant). (F–H) Tumor growth of Hepa 1-6 cells injected subcutaneously into C57BL/6 mice treated with indicated inhibitors. n=6 in each group. Data are presented as the mean±standard error. * P<0.05, **** P<0.0001, two-way ANOVA analysis with Tukey’s post-hoc test for multiple comparisons (F–H).

To further investigate the effects of complement cascade on drug response, integrative analyses were performed to evaluate the association between the complement-associated molecular features and sensitivity to anti-cancer drugs (see Materials and Methods). Based on a previously published paper, the imputed drug response to 138 anti-cancer drugs for TCGA samples was available by constructing statistical models related to gene expression [24]. Spearman’s correlation analyses revealed distinct relationships between complement scores and response to anti-cancer drugs ( Figure 7A). For example, imputed drug response to BI−2536 that targets serine/threonine-protein kinase 1 (PLK1) was positively correlated with complement scores, indicating that patients with higher complement scores were more resistant to PLK1 inhibitors ( Figure 7A). However, higher complement scores seemed to be associated with the higher sensitivity to drugs targeting important pathways, including the phosphoinositide 3-kinase (PI3K), epidermal growth factor receptor (EGFR), and vascular endothelial growth factor receptor (VEGFR) signaling pathways ( Figure 7A, and Supplementary Figure S11D). Imputed drug response to another EGFR-targeting drug lapatinib also showed negative correlations with complement scores (drug-sensitive; Figure 7B, and Supplementary Figure S11D).

To further corroborate the findings of anti-cancer drug response, the GDSC database that contains IC _50_ data of 1020 cancer cell lines was analyzed with complement scores. Similarly, correlation analyses were performed between the natural logarithm-transformed IC _50_ and complement scores ( Figure 7D). Consistent with the imputed drug response in the TCGA cohort, cancer cells with higher complement scores were more likely to be sensitive to drugs targeting EGFR and PI3K signaling pathways, and resistant to drugs that target PLK3 and chromatin histone acetylation ( Figure 7D, and Suppelmentary Figure S11A). And similar drug response to dasatinib was found in both TCGA and GDSC datasets, confirming the potency of complement scores in predicting drug response ( Figure 7C,D, and Suppelmentary Figure S11B).

Taken EGFR as an example, multiple molecular feature analyses were performed ( Figure 7E). The expression of EGFR was upregulated in the complement score-high group, while the DNA methylation level of the promoter region was downregulated. Higher methylation levels showed higher response scores to drugs lapatinib and erlotinib (drug-resistant). Meanwhile, the expression of six potential miRNAs targeting EGFR was significantly higher in complement score-high samples and was associated with lower imputed drug response scores (drug-sensitive) of corresponding EGFR-targeting drugs. On the contrary, as shown in Suppelmentary Figure S9E, the PLK1 expression was lower in complement score-high samples, and negatively correlated (drug-sensitive) with PLK1 inhibitors BI−2536 (r=–0.53, *P*=1.8×10 ^–10^) and GW843682X (r= –0.48, *P*=7.5×10 ^–9^), while the miRNA has-miR-328 targeting PLK1 showed positive correlation (drug-resistant) with BI−2536 (r=0.21, *P*=0.017) and GW843682X (r=0.25, *P*=5.9×10 ^–3^).

To further confirm these results under experimental conditions, CVF was administrated in immunocompetent mice to inhibit the complement cascade via C3 exhaustion [30]. Consistently, complement inhibition significantly promoted the tumor growth in the subcutaneous tumor model ( Suppelmentary Figure S11C). Moreover, CVF was found to enhance the drug resistance to TKIs, such as sorafenib and Lenvatinib [31], both of which are targeting the VEGFR and PDGFR ( Figure 7F,G). Nevertheless, complement inhibition increased the sensitivity of liver cancer cells to the inhibitor BI 2536 ( Figure 7H).

Taken together, these results demonstrated the clinical value of complement scores in predicting the response to anti-cancer drugs for HCC patients.

## Discussion

The complement system consists of an indispensable part of human immunity. This system is responsible for the clearance of immunocomplex, invaded bacteria, and aberrant somatic cells, including tumor cells [5]. Most complement-associated proteins are generated in the liver. However, the relationship between the complement cascade and the malignant behaviors, immune infiltration, prognosis prediction, and drug sensitivity of HCC has not been intensively investigated. In this study, we discovered that complement activation in the HCC tissues was dramatically inhibited compared to the normal liver tissues by comparing the complement scores based on the 18-gene signature in each sample. Therefore, understanding the influence of complement cascade on the molecular signatures of liver cancer is of vital importance in improving the therapeutic effect of liver cancer. Since the complement status is a relative signature, tumor samples in the TCGA-LIHC cohort were classified into complement score-low, complement score-intermediate, and complement score-high clusters based on the complement signature. In subsequent analyses, we focused on complement score-low, and score-high clusters to avoid the bias from the potential mixture [21]. This study provides a comprehensive view of complement-associated molecular features, including gene expression, miRNA expression, and DNA methylation, and complement-associated clinical values, such as predicting patient prognosis and drug response.

Cancer immunotherapy, which inflames the patients’ own immunity to fight against cancer, has been developed rapidly over the past decade. Monoclonal antibodies targeting the immune-checkpoint inhibitors (ICIs), such as programmed cell death protein 1 (PD-1) and programmed death-ligand 1 (PD-L1 or CD274), have greatly improved the prognosis of multiple tumor patients, although only 20%–40% of patients responded to this type of cancer therapy [32]. The primary effect of blocking ICIs is to reverse the exhausted status of CD8 ^+^ T cells, the most powerful effector cells in the immunosurveillance and cytotoxic effect in killing cancer cells [33]. Recently, a deep single-cell RNA sequencing study revealed that exhausted CD8 ^+^ T cells and regulatory T cells (Tregs) were preferentially enriched in the HCC tissues, indicating the disability of tumors in anti-cancer immunity and immunotherapy [34]. Moreover, together with decreased tumor-specific T cells, increased tumor-associated macrophages, neutrophils and fibroblasts, marrow-derived suppressor cells, and Tregs constitute an immunosuppressive microenvironment in HCC tissues [35]. Consistently, this study also revealed that the infiltration of most immune cells, including CD8 ^+^ T cells, NK cells, T helper cells, neutrophils, and macrophages, was decreased in the cancerous tissues compared with that in para-tumor tissues. Additionally, higher complement scores were proved to be associated with activated inflammation status and suppressed tumor-promoting pathways, such as DNA replication, and cell cycle. However, the immunosuppressive status of tumors is responsible for immune tolerance, poor infiltration of primed T cells, and decreased efficacy of immunotherapy [36]. With increased cytokines in the microenvironment, the complement-score high samples are in an inflamed state, favorable to the infiltration of immune cells. Meanwhile, CD8 ^+^ T cells and NK cells were also observed to be enriched in the microenvironment of score-high samples. These results suggested that complement cascade might be a driven factor of inflammation in HCC tissues, which is associated with the infiltration of effector cells.

Furthermore, the regulating mechanisms of complement-associated proteins were investigated. Little difference of predicted miRNAs was observed between the complement score-low and score-high groups, which probably implied that differentially expressed miRNAs are the consequences of distinct complement status. It was found in this study that the most highly expressed miRNAs in the complement score-high group are related to tumor-suppressing. For instance, lower expression of the liver-specific miRNA miR-122 is associated with the hyperactivity of oncogenic pathways and the development of hepatocarcinogenesis [37], and the miR-885-5p was reported to inhibit aerobic glycolysis by targeting the hexokinase 2 (HK2) and impede the malignant features of liver cancer [38]. Thus, higher expression of these miRNAs exerted a suppressive effect on tumor cells. On the other hand, higher complement scores are significantly correlated with lower methylation levels at the promoter regions of complement-associated genes, and complement scores are negatively associated with the expression of three DNMTs. The DNMT family, including the conical DNMT1, DNMT3A, and DNMT3B, plays a vital role in the epigenetic regulation of multiple genes, especially the transcriptional silencing by catalyzing the methylation of CpG islands at promoter regions [28]. Therefore, we speculate that lower expression of DNMTs may decrease the methylation levels of promoter regions, thereby promoting the expressions of complement-associated genes.

To evaluate the clinical benefit of the complement signature, its relationship with the patient prognosis, clinicopathological features, and response to anti-cancer drugs was investigated. According to the expression of complement-associated genes, a six-gene-based prognosis model was constructed, and the risk scores for each patient were calculated. With this prognosis model, the outcome of HCC patients could be relatively predicted, and the risk score could serve as an independent prognosis biomarker for these patients. In addition, both the complement scores and risk scores were found to be correlated with certain clinicopathological features, including pathological grade, TNM stage, and portal vein invasion. In this study, advanced HCC patients, namely those with higher pathological grade, late clinical-stage, and more severe portal vein invasion, seemed to have higher risk scores and lower complement scores. Another striking observation was the application of complement signature in predicting the drug response for HCC patients. In clinical practice, almost half of the HCC patients finally receive the systemic therapies, among which targeted therapies are especially critical for advanced HCC patients [39]. Choosing the optimal drugs in precision medicine is critical to overcoming drug resistance and improving the objective response rates (ORRs) and overall survival. In the phase III trials of sorafenib, the ORRs were only 10%–15% by mRECIST (modified Response Evaluation Criteria in Solid Tumors) criteria [40], while the ORR of patients who received the lenvatinib was 24.1% [41]. Sorafenib and lenvatinib are multitargeted receptor tyrosine kinase (RTK) inhibitors, both targeting the angiogenic RTKs (including VEGF receptors (VEGFRs) and PDGF receptors (PDGFRs)). In our analysis, we found that patients with higher complement scores were more likely to be sensitive to drugs targeting VEGFRs and PDGFRs. Thus, patients with lower complement scores might be the non-responders to sorafenib and lenvatinib. Taken together, these observations suggest that this study provides meaningful clinical insights and a method to predict the clinical benefit for certain potential anti-cancer drugs.

In summary, in this study we systematically investigated the complement-associated immune microenvironment, molecular features, and clinical relevance in HCC. Through multidimensional analysis, the gene expression, miRNA expression, tumor microenvironment, and certain clinicopathological features were tightly associated with the complement status in cancer cells. We also provided plenty of evidence for the clinical significance of risk scores and complement scores in predicting patient survival, and drug sensitivity. Liver tumors with higher complement scores are preferentially in a tumor-suppressive state, and the regulatory networks are interacting in complicated ways at multiple layers. However, due to limited methods of intervening the complement status of tumor cells, it is difficult to validate these findings in a more rigorous laboratory setting. Nonetheless, this comprehensive study calls attention to the necessity to include tumor complement status in future studies.

## Supporting information

Supplementary_table_2

Supplementary_table_1

Supplementary_figureS

Supplementary_table_3

Supplementary_table_4

Supplementary_table_7

Supplementary_table_5

## Supplementary Data

Supplementary Data is available at *Acta Biochimica et Biphysica Sinica* online.
