# Supplementary material for: Comprehensive analysis of complement-associated molecular features in hepatocellular carcinoma: Complement-associated molecular features in hepatocellular carcinoma
Source: Acta Biochim Biophys Sin (Shanghai). 2022 Aug 2;54(11):1694–707. doi: 10.3724/abbs.2022097 (PMC9828444; doi:10.3724/abbs.2022097)
Supplement: Supplementary_table_2 [file Supplementary_table_2.pdf]

Supplementary Table S2. Common significantly changed genes in 11 datasets using RobustRan

Genesymbol	Pvalue	adjPvalue	logFC
GPC3	1.00E-30	3.93E-26	2.80733
SPINK1	2.16E-30	8.46E-26	2.980629
TOP2A	5.24E-29	2.05E-24	2.250751
CDKN3	5.52E-26	2.16E-21	2.030668
AKR1B10	2.82E-25	1.10E-20	2.582864
REG3A	1.07E-22	4.20E-18	1.863546
ASPM	7.19E-22	2.82E-17	1.851481
CDC20	2.55E-21	9.99E-17	1.746291
ECT2	5.10E-21	2.00E-16	1.303443
CCNB2	9.82E-21	3.85E-16	1.637237
COL15A1	1.46E-20	5.70E-16	1.474723
HMMR	3.65E-20	1.43E-15	1.391569
CCNB1	6.55E-20	2.56E-15	1.619212
SQLE	9.50E-20	3.72E-15	1.360108
PTTG1	1.28E-19	5.01E-15	1.562419
SPP1	3.48E-19	1.36E-14	1.368753
NUSAP1	3.50E-19	1.37E-14	1.409686
TTK	4.07E-19	1.59E-14	1.516589
AURKA	6.10E-19	2.39E-14	1.371016
MELK	7.38E-19	2.89E-14	1.491126
KIF20A	1.08E-18	4.21E-14	1.482828
CAP2	1.08E-18	4.21E-14	1.595463
PRC1	2.17E-18	8.50E-14	1.527111
CCNA2	2.62E-18	1.02E-13	1.294176
S100P	3.09E-18	1.21E-13	1.366781
IGF2BP3	3.79E-18	1.48E-13	1.288914
NCAPG	4.44E-18	1.74E-13	1.392227
PAGE4	6.80E-18	2.66E-13	1.311831
RRM2	7.01E-18	2.74E-13	1.583673
UBE2C	8.69E-18	3.40E-13	1.5652
SULT1C2	1.41E-17	5.50E-13	1.393809
CENPF	1.50E-17	5.88E-13	1.528628
TRIP13	1.98E-17	7.74E-13	1.149565
MUC13	1.98E-17	7.74E-13	1.396735
LCN2	2.41E-17	9.44E-13	1.496729
PBK	4.05E-17	1.59E-12	1.419496
ALDH3A1	4.45E-17	1.74E-12	1.176479
ESM1	1.03E-16	4.01E-12	1.489969
TKT	1.09E-16	4.27E-12	1.099602
PLVAP	1.97E-16	7.71E-12	1.215488
HSPB1	4.59E-16	1.80E-11	1.004048
NQO1	7.68E-16	3.01E-11	1.31513
KIAA0101	1.07E-15	4.20E-11	1.17873
DKK1	1.28E-15	4.99E-11	1.03049
UBD	1.35E-15	5.30E-11	1.373314
MDK	2.22E-15	8.69E-11	1.488817
UBE2T	2.94E-15	1.15E-10	1.356907
CEP55	3.93E-15	1.54E-10	1.083589
DLGAP5	3.94E-15	1.54E-10	1.062938
MEP1A	4.64E-15	1.81E-10	1.029367
KIF2C	5.59E-15	2.19E-10	1.056044
BUB1B	5.61E-15	2.20E-10	1.308217
TXNRD1	5.66E-15	2.22E-10	1.004871
FAM83D	8.61E-15	3.37E-10	1.14477
BUB1	1.29E-14	5.03E-10	1.325468
THBS4	1.76E-14	6.90E-10	1.345413

REG1A	1.79E-14	7.01E-10	1.202165
NEK2	2.04E-14	7.98E-10	1.521737
KIFC1	3.46E-14	1.35E-09	1.266488
CENPA	4.42E-14	1.73E-09	1.258321
HJURP	8.11E-14	3.18E-09	1.037865
DTL	8.83E-14	3.46E-09	1.265525
TPX2	9.39E-14	3.67E-09	1.097369
NDC80	1.04E-13	4.08E-09	1.189237
MCM2	1.28E-13	5.00E-09	1.093842
AKR1C3	1.31E-13	5.13E-09	1.115798
FOXM1	1.38E-13	5.41E-09	1.247606
ACTG2	1.72E-13	6.72E-09	1.021849
CDCA3	2.79E-13	1.09E-08	1.198203
MAGEA1	3.32E-13	1.30E-08	1.312363
CDC25C	3.84E-13	1.50E-08	1.278258
CDCA5	6.50E-13	2.54E-08	1.129023
CYP17A1	6.53E-13	2.55E-08	1.026466
PEG10	7.46E-13	2.92E-08	1.193028
CCL20	9.60E-13	3.76E-08	1.220579
CKAP2L	1.26E-12	4.95E-08	1.097474
ANLN	1.28E-12	5.03E-08	1.397806
GMNN	2.45E-12	9.58E-08	1.020148
AURKB	2.78E-12	1.09E-07	1.089917
RACGAP1	2.94E-12	1.15E-07	1.057052
SFN	2.98E-12	1.17E-07	1.16683
CDC6	3.34E-12	1.31E-07	1.028083
MMP11	4.11E-12	1.61E-07	1.060668
COX7B2	7.04E-12	2.76E-07	1.250055
KIF4A	9.56E-12	3.74E-07	1.19933
ZWINT	1.16E-11	4.54E-07	1.011767
ROBO1	1.45E-11	5.69E-07	1.121471
THY1	1.45E-11	5.69E-07	1.020432
TGM3	1.58E-11	6.20E-07	1.240473
BIRC5	1.72E-11	6.72E-07	1.323353
SPC24	3.30E-11	1.29E-06	1.048878
NUF2	5.55E-11	2.17E-06	1.228067
MKI67	7.95E-11	3.11E-06	1.119317
HOXA13	1.16E-10	4.55E-06	1.264312
CDKN2A	1.28E-10	5.02E-06	1.066425
CENPE	1.52E-10	5.95E-06	1.006661
ZIC2	1.61E-10	6.31E-06	1.163671
CDCA8	2.12E-10	8.29E-06	1.051979
CENPM	5.83E-10	2.28E-05	1.024128
MND1	5.93E-10	2.32E-05	1.072787
CELSR3	6.64E-10	2.60E-05	1.053985
TROAP	1.10E-09	4.32E-05	1.079568
SLC7A11	1.11E-09	4.34E-05	1.02452
SPC25	2.36E-09	9.22E-05	1.061587
ACSL4	2.38E-09	9.30E-05	1.052973
E2F8	6.50E-09	0.000254	1.048208
EXO1	1.48E-08	0.000581	1.068985
EPS8L3	2.19E-08	0.000858	1.119803
CYP1A2	5.94E-31	2.32E-26	-3.64526
C7	1.33E-28	5.21E-24	-2.53871
FCN3	2.45E-28	9.58E-24	-3.29178
C9	6.15E-26	2.41E-21	-2.72726
MT1F	1.51E-24	5.92E-20	-2.96256
LCAT	3.66E-24	1.43E-19	-1.98541

SLC22A1	9.62E-24	3.77E-19	-2.42537
VIPR1	1.44E-23	5.64E-19	-2.35442
LY6E	1.64E-23	6.41E-19	-1.95957
HAMP	3.62E-23	1.42E-18	-3.50102
GHR	6.35E-23	2.48E-18	-1.946
SPP2	1.33E-22	5.20E-18	-1.98513
CYP3A4	2.01E-22	7.87E-18	-2.62172
THRSP	2.10E-22	8.22E-18	-2.25308
AFM	2.19E-22	8.58E-18	-1.9284
GBA3	2.56E-22	1.00E-17	-2.06754
BBOX1	2.71E-22	1.06E-17	-1.89405
CLEC1B	3.62E-22	1.42E-17	-3.23175
APOF	3.63E-22	1.42E-17	-2.5274
MT1G	6.48E-22	2.54E-17	-2.855
MARCO	7.07E-22	2.77E-17	-2.7214
CXCL14	7.07E-22	2.77E-17	-2.58312
NAT2	7.19E-22	2.82E-17	-2.46274
CLEC4G	1.08E-21	4.24E-17	-2.66053
ECM1	2.27E-21	8.88E-17	-1.81805
CYP4A11	2.94E-21	1.15E-16	-1.77288
ACSM3	4.09E-21	1.60E-16	-1.42876
MT1H	4.63E-21	1.81E-16	-2.78331
DNASE1L3	6.06E-21	2.37E-16	-2.17293
SLCO1B3	9.26E-21	3.62E-16	-2.45221
F9	9.43E-21	3.69E-16	-1.80658
HGFAC	9.82E-21	3.84E-16	-2.326
ADH4	2.57E-20	1.01E-15	-1.98606
HSD17B13	3.66E-20	1.43E-15	-2.35162
CCL19	5.13E-20	2.01E-15	-1.92269
CXCL12	5.59E-20	2.19E-15	-1.85763
HAO2	5.99E-20	2.35E-15	-2.18569
MFAP4	6.87E-20	2.69E-15	-1.87643
DBH	7.35E-20	2.88E-15	-2.00718
ADH1A	1.06E-19	4.16E-15	-1.36055
TDO2	1.14E-19	4.48E-15	-1.72413
CYP39A1	1.28E-19	5.01E-15	-2.03243
SRPX	1.28E-19	5.03E-15	-1.91132
FCN2	1.41E-19	5.53E-15	-2.64863
TAT	1.48E-19	5.78E-15	-1.71484
BHMT	1.83E-19	7.15E-15	-1.58394
C6	1.89E-19	7.41E-15	-1.45357
MT1X	2.00E-19	7.81E-15	-2.22049
RDH16	2.46E-19	9.62E-15	-1.97064
ENO3	2.61E-19	1.02E-14	-1.51469
PCK1	2.85E-19	1.12E-14	-1.86924
CD5L	2.95E-19	1.15E-14	-2.01049
CRHBP	3.30E-19	1.29E-14	-2.68282
AKR1D1	4.27E-19	1.67E-14	-1.767
CETP	4.34E-19	1.70E-14	-1.75549
PROZ	4.40E-19	1.72E-14	-1.5171
GCGR	7.41E-19	2.90E-14	-1.80425
PLAC8	9.65E-19	3.78E-14	-1.81466
DCN	1.02E-18	3.98E-14	-2.22171
CYP2E1	1.06E-18	4.15E-14	-1.95015
SLC10A1	1.25E-18	4.89E-14	-1.69736
DPT	1.28E-18	5.01E-14	-1.97286
OLFML3	1.96E-18	7.68E-14	-1.49418
CLRN3	5.21E-18	2.04E-13	-1.79843

FAM134B	5.57E-18	2.18E-13	-1.44993
MT1M	5.62E-18	2.20E-13	-2.74654
ADH1B	6.42E-18	2.51E-13	-1.64216
ADAMTSL2	7.00E-18	2.74E-13	-1.47654
PLG	7.38E-18	2.89E-13	-1.12892
CYP2A7	8.10E-18	3.17E-13	-1.90729
FOS	9.15E-18	3.58E-13	-2.05037
GYS2	1.04E-17	4.08E-13	-1.69036
GSTZ1	1.41E-17	5.53E-13	-1.3949
CYP2C9	1.47E-17	5.75E-13	-1.72958
CCL21	1.75E-17	6.87E-13	-1.40033
IGFALS	1.82E-17	7.14E-13	-1.94192
CYP2C19	2.05E-17	8.04E-13	-1.38365
IGFBP3	2.17E-17	8.49E-13	-1.66316
FOSB	2.35E-17	9.21E-13	-1.79564
COLEC10	2.58E-17	1.01E-12	-2.28221
EPHX2	2.79E-17	1.09E-12	-1.17928
SHBG	3.33E-17	1.30E-12	-1.86058
SAA4	3.48E-17	1.36E-12	-1.57537
GPD1	5.18E-17	2.03E-12	-1.28748
SDS	5.29E-17	2.07E-12	-1.74328
STAB2	5.71E-17	2.23E-12	-2.13485
KCNN2	5.79E-17	2.27E-12	-1.75642
FBP1	6.94E-17	2.72E-12	-1.68449
COLEC11	7.11E-17	2.78E-12	-1.43011
OGDHL	7.61E-17	2.98E-12	-1.32747
CYP2A6	7.92E-17	3.10E-12	-2.10746
MS4A6A	8.13E-17	3.18E-12	-1.20285
MBL2	8.84E-17	3.46E-12	-1.47064
CHST4	8.94E-17	3.50E-12	-1.89275
TMEM27	9.29E-17	3.64E-12	-1.76703
SRD5A2	9.79E-17	3.83E-12	-1.84512
CYP2J2	1.04E-16	4.07E-12	-1.0644
LECT2	1.04E-16	4.08E-12	-1.4199
GLYAT	1.09E-16	4.26E-12	-1.67672
NTF3	1.22E-16	4.76E-12	-1.19717
CLEC4M	1.24E-16	4.87E-12	-2.56049
ABCA8	1.44E-16	5.62E-12	-1.38444
HSD17B2	1.49E-16	5.84E-12	-1.1491
KMO	1.55E-16	6.06E-12	-1.55757
GSTA2	1.59E-16	6.24E-12	-1.30021
PZP	1.87E-16	7.33E-12	-1.8544
NNMT	2.01E-16	7.88E-12	-1.81471
MT1E	2.95E-16	1.15E-11	-1.96574
ALDH8A1	2.98E-16	1.17E-11	-1.36692
PROM1	3.25E-16	1.27E-11	-1.48294
CYP2C8	3.43E-16	1.34E-11	-1.8704
IL1RAP	3.76E-16	1.47E-11	-1.11074
CYP8B1	4.89E-16	1.91E-11	-1.49425
C8B	5.21E-16	2.04E-11	-1.18116
PON1	5.66E-16	2.21E-11	-1.09755
CNDP1	5.76E-16	2.25E-11	-2.00309
GZMK	5.92E-16	2.32E-11	-1.02655
CIDEB	6.31E-16	2.47E-11	-1.17887
ACAA1	6.72E-16	2.63E-11	-1.04
BCHE	6.94E-16	2.72E-11	-1.59988
PHGDH	8.94E-16	3.50E-11	-1.27927
VNN1	1.01E-15	3.93E-11	-1.39591

CDHR2	1.05E-15	4.09E-11	-1.64521
KLKB1	1.09E-15	4.25E-11	-1.39669
FBLN5	1.21E-15	4.73E-11	-1.1926
FTCD	1.30E-15	5.09E-11	-1.20558
CYP2B6	1.46E-15	5.70E-11	-1.71068
LYVE1	1.50E-15	5.88E-11	-1.61338
RDH5	1.52E-15	5.94E-11	-1.26234
ETFDH	1.96E-15	7.66E-11	-1.12745
CYP2C18	2.03E-15	7.94E-11	-1.04996
ADH6	2.03E-15	7.94E-11	-1.14236
CD163	2.26E-15	8.85E-11	-1.15136
FETUB	2.30E-15	8.99E-11	-1.10377
ID1	2.45E-15	9.59E-11	-1.5571
OIT3	2.45E-15	9.60E-11	-1.95904
ADH1C	2.55E-15	9.97E-11	-1.62275
ATF5	2.61E-15	1.02E-10	-1.19663
PRSS8	2.82E-15	1.10E-10	-1.23652
GCKR	2.88E-15	1.13E-10	-1.04547
HPGD	3.02E-15	1.18E-10	-1.41049
SLC39A5	3.25E-15	1.27E-10	-1.22821
ALDH6A1	3.82E-15	1.50E-10	-1.12838
BGN	4.19E-15	1.64E-10	-1.08093
PRG4	4.23E-15	1.66E-10	-1.06046
CA2	4.74E-15	1.85E-10	-1.17144
INMT	4.79E-15	1.87E-10	-1.50601
RBP1	5.65E-15	2.21E-10	-1.15327
HSD11B1	6.29E-15	2.46E-10	-1.41385
FCGR2B	6.37E-15	2.49E-10	-1.44092
RND3	7.64E-15	2.99E-10	-1.59404
OXT	7.95E-15	3.11E-10	-1.03057
ACSL1	7.97E-15	3.12E-10	-1.09787
SFRP5	8.05E-15	3.15E-10	-1.8312
HBB	8.44E-15	3.30E-10	-1.45569
ACADS	9.32E-15	3.65E-10	-1.05514
CPS1	1.18E-14	4.62E-10	-1.20212
HPD	1.29E-14	5.06E-10	-1.47848
F11	1.49E-14	5.82E-10	-1.09723
FXYD1	1.63E-14	6.36E-10	-1.35914
TMEM45A	1.76E-14	6.90E-10	-1.33402
CFP	1.99E-14	7.80E-10	-1.58919
CYP26A1	2.09E-14	8.19E-10	-1.7386
SLC19A3	2.28E-14	8.92E-10	-1.0742
ASPA	2.75E-14	1.08E-09	-1.19243
ANXA10	2.88E-14	1.13E-09	-1.60056
XDH	2.88E-14	1.13E-09	-1.17975
CD1D	2.99E-14	1.17E-09	-1.03633
SKAP1	3.48E-14	1.36E-09	-1.13171
GLYATL1	3.54E-14	1.39E-09	-1.23827
SERPINA11	3.63E-14	1.42E-09	-1.51164
HRG	4.23E-14	1.66E-09	-1.01631
EGR1	4.32E-14	1.69E-09	-1.57212
LIFR	4.99E-14	1.95E-09	-1.54653
IGF1	5.25E-14	2.06E-09	-1.33944
C8A	5.90E-14	2.31E-09	-1.37296
VSIG4	6.16E-14	2.41E-09	-1.02101
CDA	6.43E-14	2.52E-09	-1.04897
LUM	7.50E-14	2.94E-09	-1.38493
CLDN10	7.68E-14	3.01E-09	-1.57466

ALPL	7.91E-14	3.09E-09	-1.27068
ADRA1A	8.30E-14	3.25E-09	-1.39562
SOCS2	8.48E-14	3.32E-09	-1.35915
IL13RA2	8.92E-14	3.49E-09	-1.38068
C8orf4	9.91E-14	3.88E-09	-1.48758
TACSTD2	1.12E-13	4.40E-09	-1.35718
CXCL2	1.23E-13	4.80E-09	-1.4404
SLC22A10	1.23E-13	4.81E-09	-1.34148
KBTBD11	1.26E-13	4.94E-09	-1.40429
ALDOB	1.26E-13	4.94E-09	-1.07374
SLC27A5	1.29E-13	5.06E-09	-1.30949
GPR128	1.46E-13	5.70E-09	-1.43414
GLS2	1.68E-13	6.57E-09	-1.89224
ACADL	1.90E-13	7.45E-09	-1.12326
ASS1	2.10E-13	8.22E-09	-1.21555
OTC	2.29E-13	8.97E-09	-1.00204
SFRP1	2.36E-13	9.24E-09	-1.01582
CYP3A43	2.70E-13	1.06E-08	-1.23673
SLC46A3	3.19E-13	1.25E-08	-1.04572
GREM2	3.63E-13	1.42E-08	-1.20585
DEFB1	4.01E-13	1.57E-08	-1.06876
DNAJC12	4.99E-13	1.95E-08	-1.08444
MT2A	5.16E-13	2.02E-08	-1.59117
GNMT	5.67E-13	2.22E-08	-1.36255
CTH	6.52E-13	2.55E-08	-1.12412
HAL	6.94E-13	2.71E-08	-1.17013
SLC5A1	7.98E-13	3.12E-08	-1.41664
WDR72	7.99E-13	3.13E-08	-1.09005
ZG16	8.35E-13	3.27E-08	-1.32598
UGT2B10	8.99E-13	3.52E-08	-1.18851
PDGFRA	1.14E-12	4.47E-08	-1.24061
OAT	1.15E-12	4.51E-08	-1.10603
PGLYRP2	1.20E-12	4.72E-08	-1.18124
RCL1	1.36E-12	5.31E-08	-1.03963
THBS1	1.45E-12	5.69E-08	-1.00413
AZGP1	1.75E-12	6.85E-08	-1.0417
GABRP	2.27E-12	8.89E-08	-1.16346
APOA5	2.54E-12	9.96E-08	-1.32815
PTGDS	2.70E-12	1.06E-07	-1.3109
UROC1	2.76E-12	1.08E-07	-1.57055
ANGPTL6	2.94E-12	1.15E-07	-1.20452
ANK3	3.07E-12	1.20E-07	-1.05426
MT1A	3.14E-12	1.23E-07	-1.506
CFTR	3.23E-12	1.26E-07	-1.45557
CYR61	3.29E-12	1.29E-07	-1.22805
RSPO3	3.43E-12	1.34E-07	-1.58013
MXRA5	3.46E-12	1.35E-07	-1.02234
AKR7A3	3.74E-12	1.46E-07	-1.33997
MYOM2	3.79E-12	1.48E-07	-1.12469
HAO1	4.56E-12	1.79E-07	-1.0117
PHLDA1	4.71E-12	1.84E-07	-1.07305
IGJ	5.37E-12	2.10E-07	-1.55255
SERPINA4	5.51E-12	2.16E-07	-1.04385
CCL2	5.61E-12	2.19E-07	-1.01496
SULT1E1	6.38E-12	2.50E-07	-1.02417
PBLD	1.15E-11	4.49E-07	-1.09155
AGXT2	1.18E-11	4.60E-07	-1.00644
NGFR	1.49E-11	5.85E-07	-1.12459

MAN1C1	1.75E-11	6.83E-07	-1.01789
RAB25	2.09E-11	8.17E-07	-1.25009
ASPG	2.42E-11	9.47E-07	-1.34182
NPY1R	2.60E-11	1.02E-06	-1.41156
LPA	2.70E-11	1.06E-06	-1.45142
HPX	2.71E-11	1.06E-06	-1.0412
GADD45B	3.23E-11	1.27E-06	-1.08113
RANBP3L	3.30E-11	1.29E-06	-1.00234
KCNJ16	3.60E-11	1.41E-06	-1.19624
MFSD2A	3.74E-11	1.46E-06	-1.44511
NPC1L1	3.80E-11	1.49E-06	-1.00866
CLIC6	4.28E-11	1.67E-06	-1.15915
GRAMD1C	4.83E-11	1.89E-06	-1.00935
PTH1R	5.27E-11	2.06E-06	-1.22488
CYP4F2	5.54E-11	2.17E-06	-1.05755
LRAT	6.99E-11	2.74E-06	-1.20086
TTC36	7.38E-11	2.89E-06	-1.48661
CXCL6	9.83E-11	3.85E-06	-1.15958
SLC38A4	1.07E-10	4.17E-06	-1.00754
IGF2	1.09E-10	4.25E-06	-1.26192
NPW	1.33E-10	5.22E-06	-1.05394
C1QTNF1	1.51E-10	5.93E-06	-1.00436
HGF	2.57E-10	1.00E-05	-1.16504
AADAT	3.44E-10	1.35E-05	-1.21516
GPM6A	3.74E-10	1.46E-05	-1.31834
BMPER	4.03E-10	1.58E-05	-1.35452
SLC25A47	4.23E-10	1.66E-05	-1.07508
TFPI2	4.26E-10	1.67E-05	-1.11733
FAM83F	6.89E-10	2.69E-05	-1.00667
ESR1	7.40E-10	2.89E-05	-1.05346
PTGIS	9.70E-10	3.80E-05	-1.00458
CCL14	9.97E-10	3.90E-05	-1.04044
ANXA3	1.12E-09	4.39E-05	-1.00148
CYP2B7P1	1.12E-09	4.39E-05	-1.16403
C10orf116	1.26E-09	4.92E-05	-1.0244
CYP1A1	1.34E-09	5.26E-05	-1.08878
BCO2	1.66E-09	6.50E-05	-1.16968
TIMD4	2.38E-09	9.31E-05	-1.37099
ITLN1	2.42E-09	9.46E-05	-1.37794
KRT19	3.25E-09	0.000127	-1.19731
DIRAS3	3.43E-09	0.000134	-1.18948
IDO2	4.08E-09	0.00016	-1.14291
HEPACAM	5.06E-09	0.000198	-1.11873
MOGAT2	7.81E-09	0.000306	-1.05891
HHIP	8.24E-09	0.000323	-1.17099
BMP5	8.39E-09	0.000328	-1.03941
SYT9	8.90E-09	0.000348	-1.13176
CCBE1	1.32E-08	0.000518	-1.29745
CNTN3	2.47E-08	0.000968	-1.03426
HBA1	9.31E-08	0.003643	-1.04052
GPR182	1.02E-07	0.003992	-1.13611
PLCXD3	1.18E-07	0.004624	-1.08292
HAND2	2.27E-07	0.008883	-1.0953
GDF2	1.17E-06	0.045783	-1.36709

ıkAggreg package
