# Supplementary material for: Comprehensive analysis of complement-associated molecular features in hepatocellular carcinoma: Complement-associated molecular features in hepatocellular carcinoma
Source: Acta Biochim Biophys Sin (Shanghai). 2022 Aug 2;54(11):1694–707. doi: 10.3724/abbs.2022097 (PMC9828444; doi:10.3724/abbs.2022097)
Supplement: Supplementary_table_1 [file Supplementary_table_1.pdf]

Supplementary Table S1. Sample size of datasets used in this study

	Normal	Tumor	Platform
TCGA-LIHC	49	351	RNA-Seq
ICGC_LIRI_JP	177	212	RNA-Seq
GSE22058	97	100	Rosetta/Merck Human RSTA Custom Affymetrix 1.0 microarray
GSE46444	48	88	Illumina Human Whole-Genome DASL HT
GSE54236	80	81	Agilent-014850 Whole Human Genome Microarray 4x44K G4112F
GSE63898	168	228	Affymetrix Human Genome U219 Array
GSE64041	60	60	Affymetrix Human Gene 1.0 ST Array
GSE76427	52	115	Illumina HumanHT-12 V4.0 expression beadchip
GSE36376	193	240	Illumina HumanHT-12 V4.0 expression beadchip
GSE14520	220	225	Affymetrix Human Genome U133A 2.0 Array
GSE10143	82	80	Human 6k Transcriptionally Informative Gene Panel for DASL
