# Supplementary material for: Comprehensive analysis of complement-associated molecular features in hepatocellular carcinoma: Complement-associated molecular features in hepatocellular carcinoma
Source: Acta Biochim Biophys Sin (Shanghai). 2022 Aug 2;54(11):1694–707. doi: 10.3724/abbs.2022097 (PMC9828444; doi:10.3724/abbs.2022097)
Supplement: Supplementary_figureS [file Supplementary_figureS.pdf]

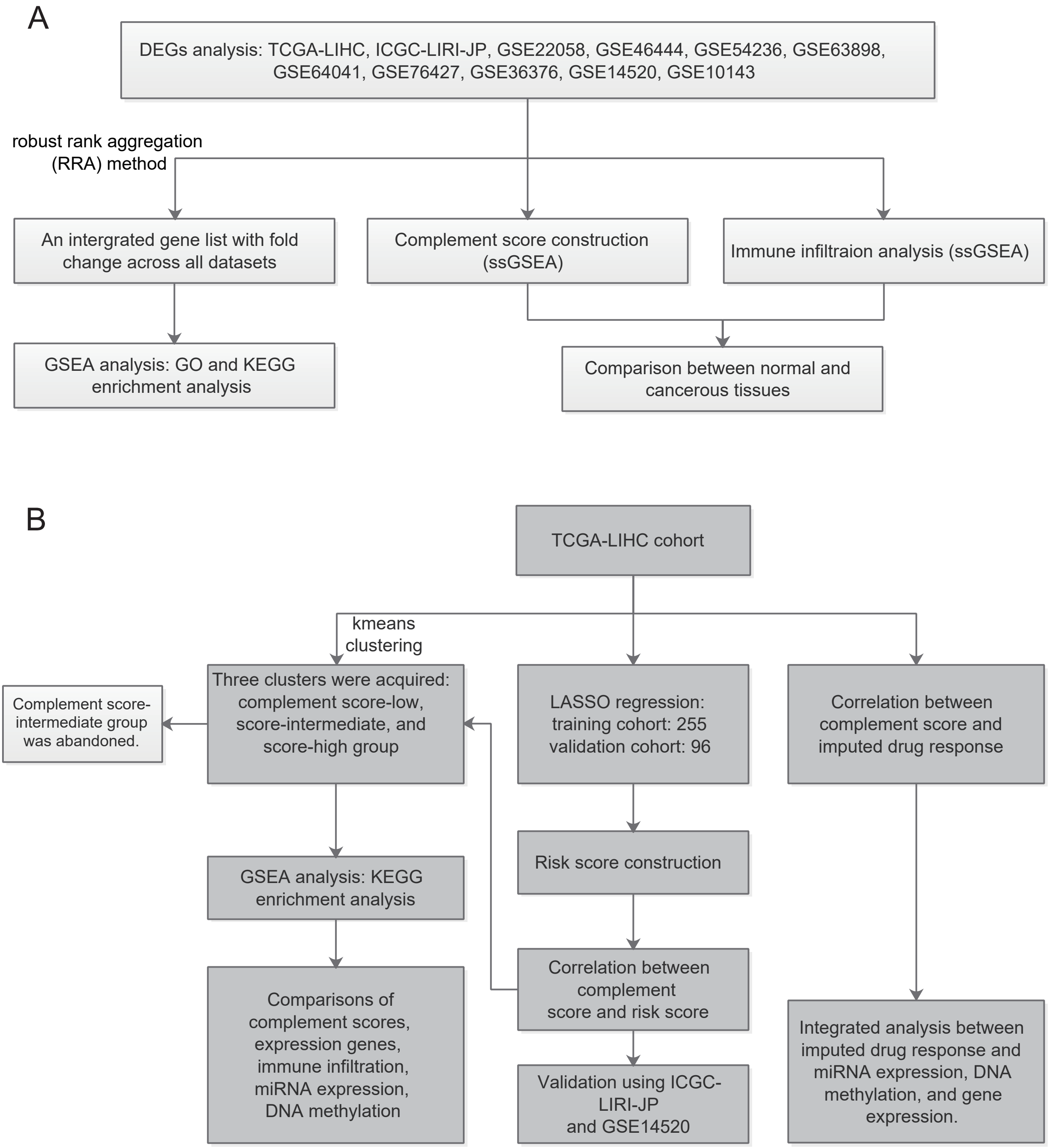

Supplementary Figure S1. The flow chart of data processing in this study.

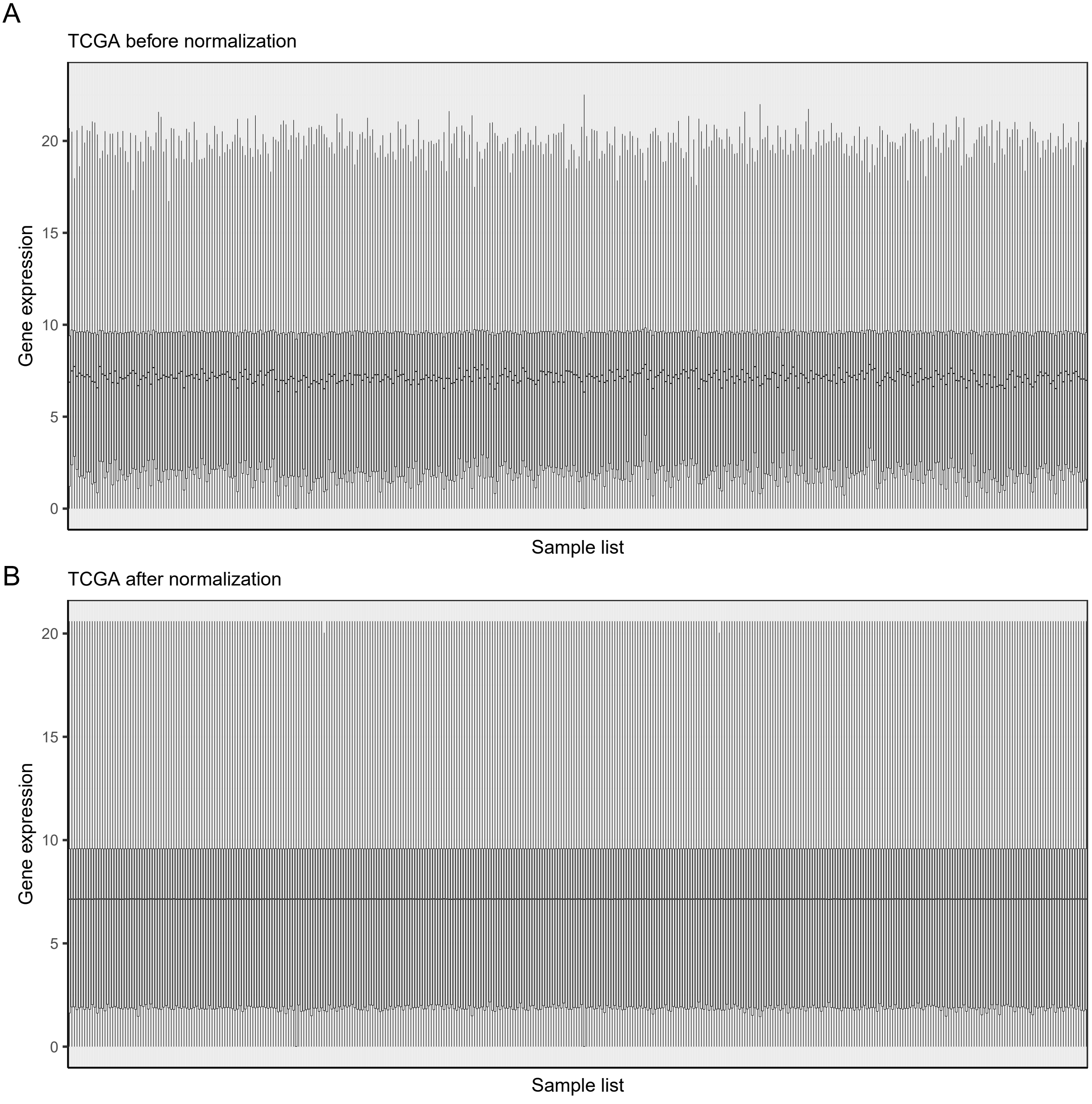

Supplementary Figure S2. An example of data normalization using the normalizeBetweenArrays function in “limma” R package.

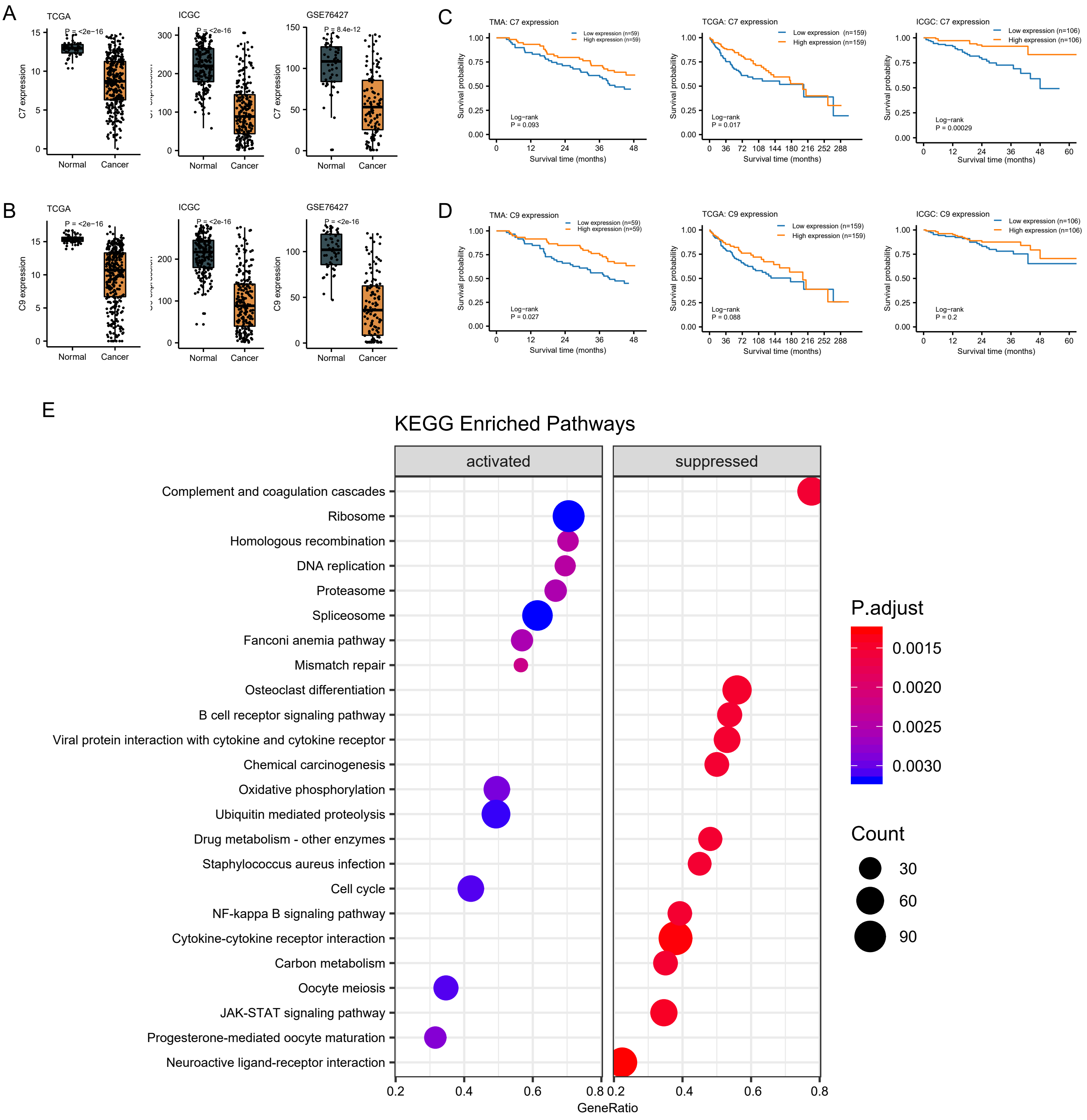

Supplementary Figure S3. Related to Figure 1. A and B, complement C7 and C9 expression in TCGA, ICGC, and GSE76427. C and D, survival curves in TMA, TCGA, and ICGC cohorts according the median expression of C7 and C9. E, KEGG enriched pathways of integrated gene list with fold change after RRA analysis.

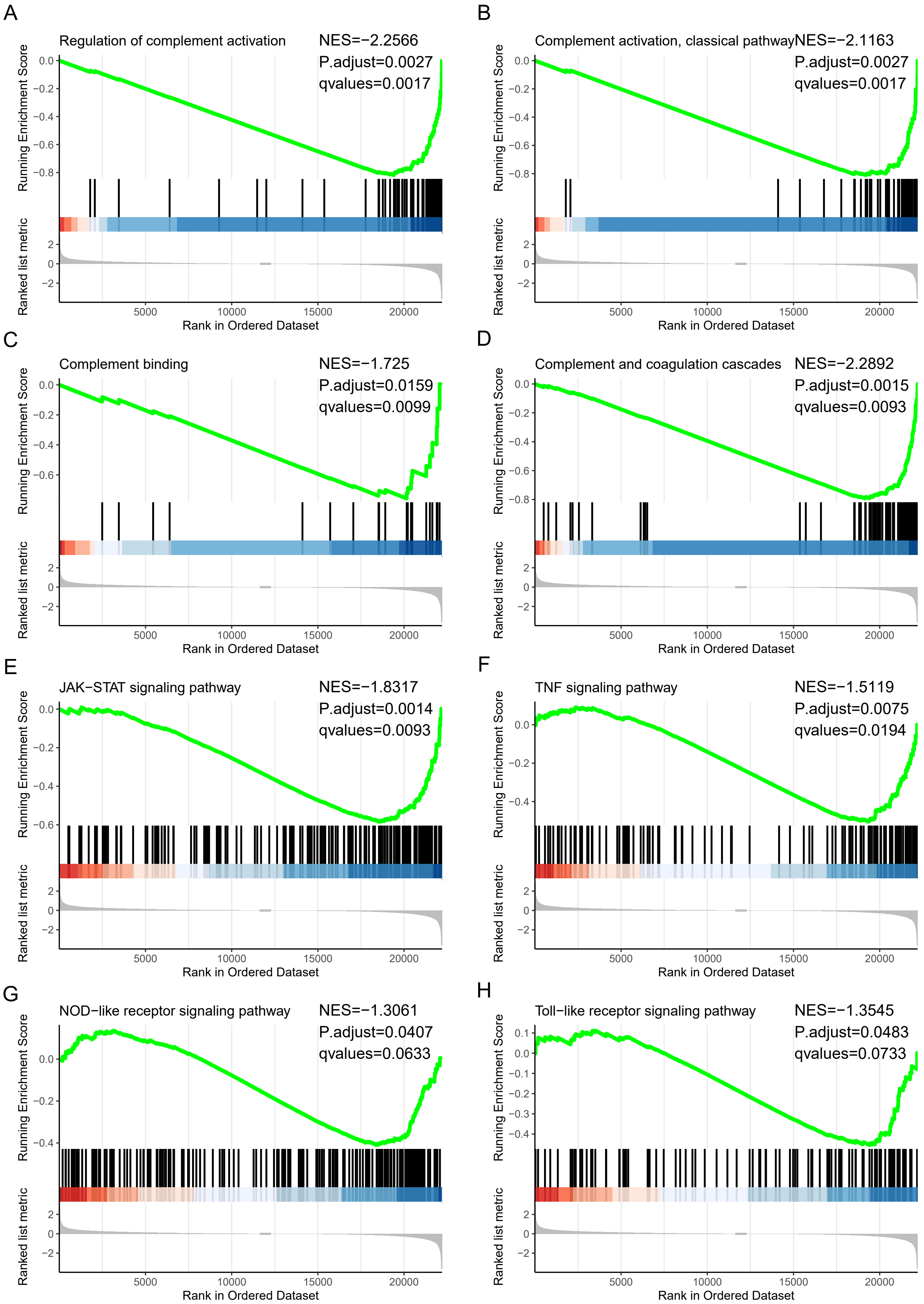

Supplementary Figure S4. Related to Figure 1. GSEA analysis of the pathways in complement activation and innate immunity.

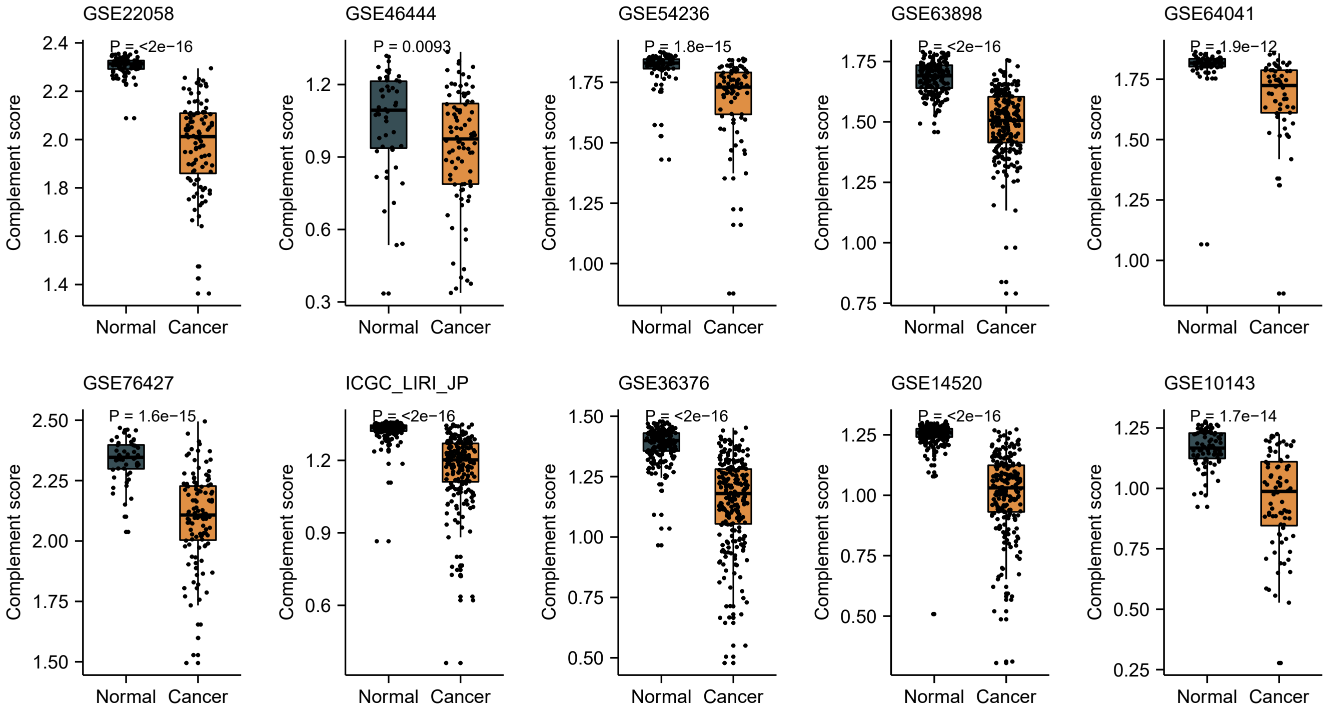

Supplementary Figure S5. Complement scores comparisons between tumor and non-tumor tissues in various datasets.

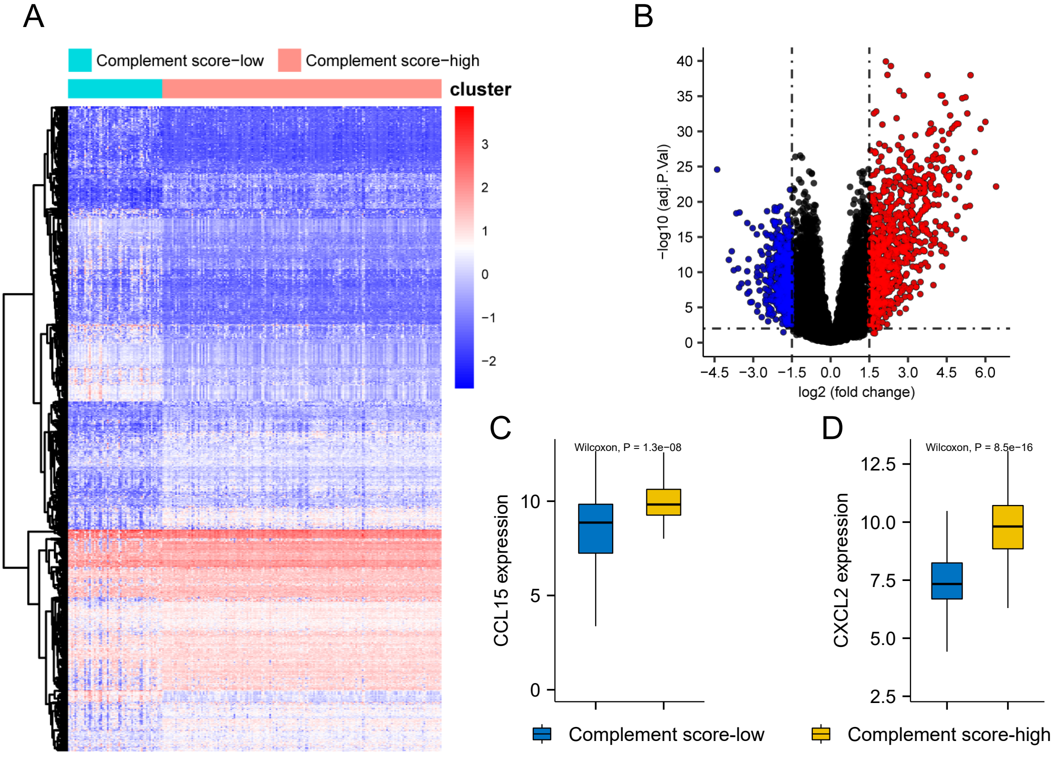

Supplementary Figure S6. Related to Figure 3. A and B, heatmap and volcano plot of the gene expression between complement score-high and score-low groups. The red dots and blue dots indicated the significant DEGs compared with the complement score-low cohort (P.adjusted < 0.05, |logFC| > 1.5). C and D, boxplots indicating the significantly higher expression of CCL15 and CXCL2 in complement score-high group.

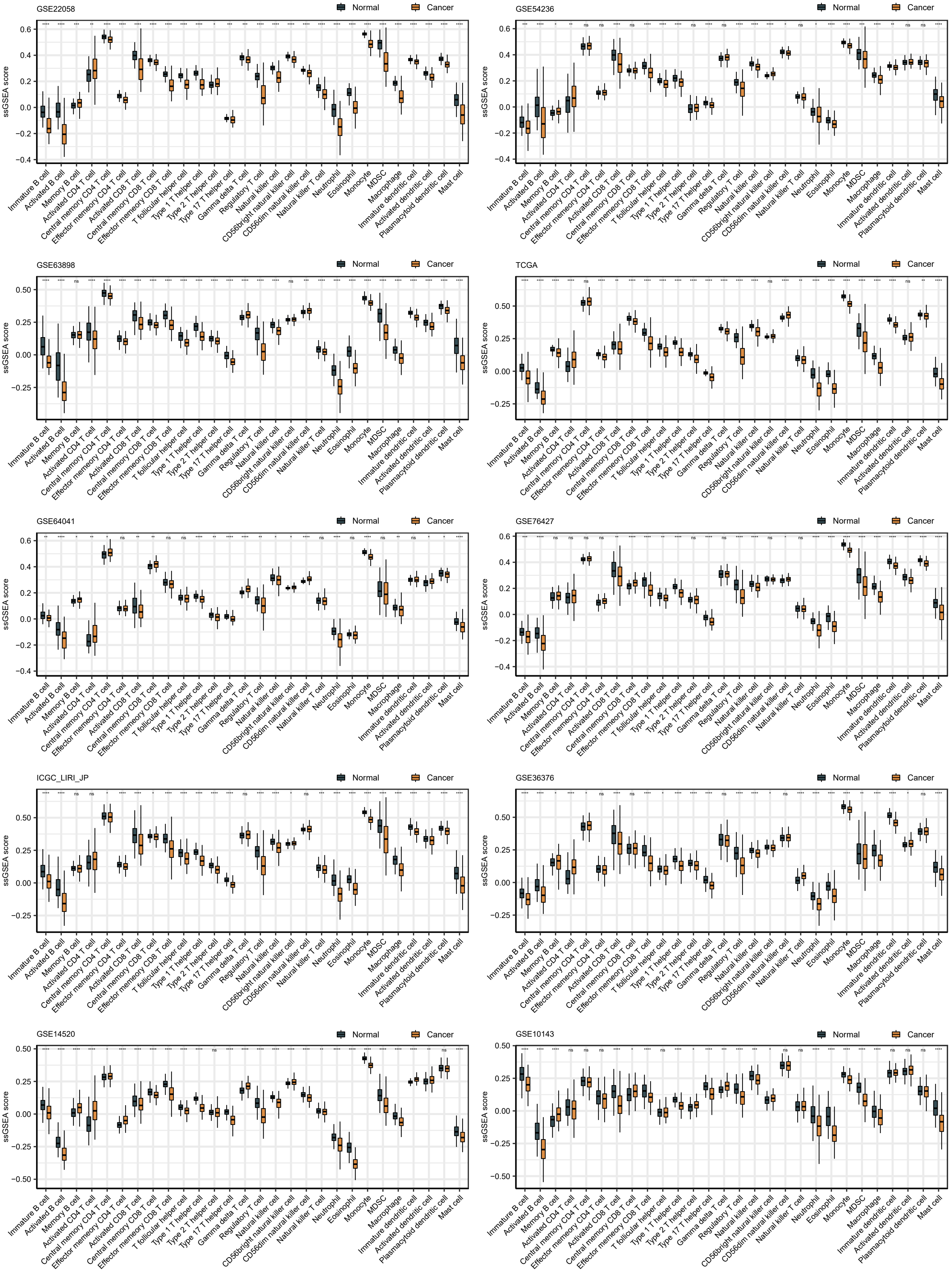

Supplementary Figure S7. Comparisons of the immune infiltration levels of 28 types of immune cells between normal and cancer tissues in 10 distinct datasets.

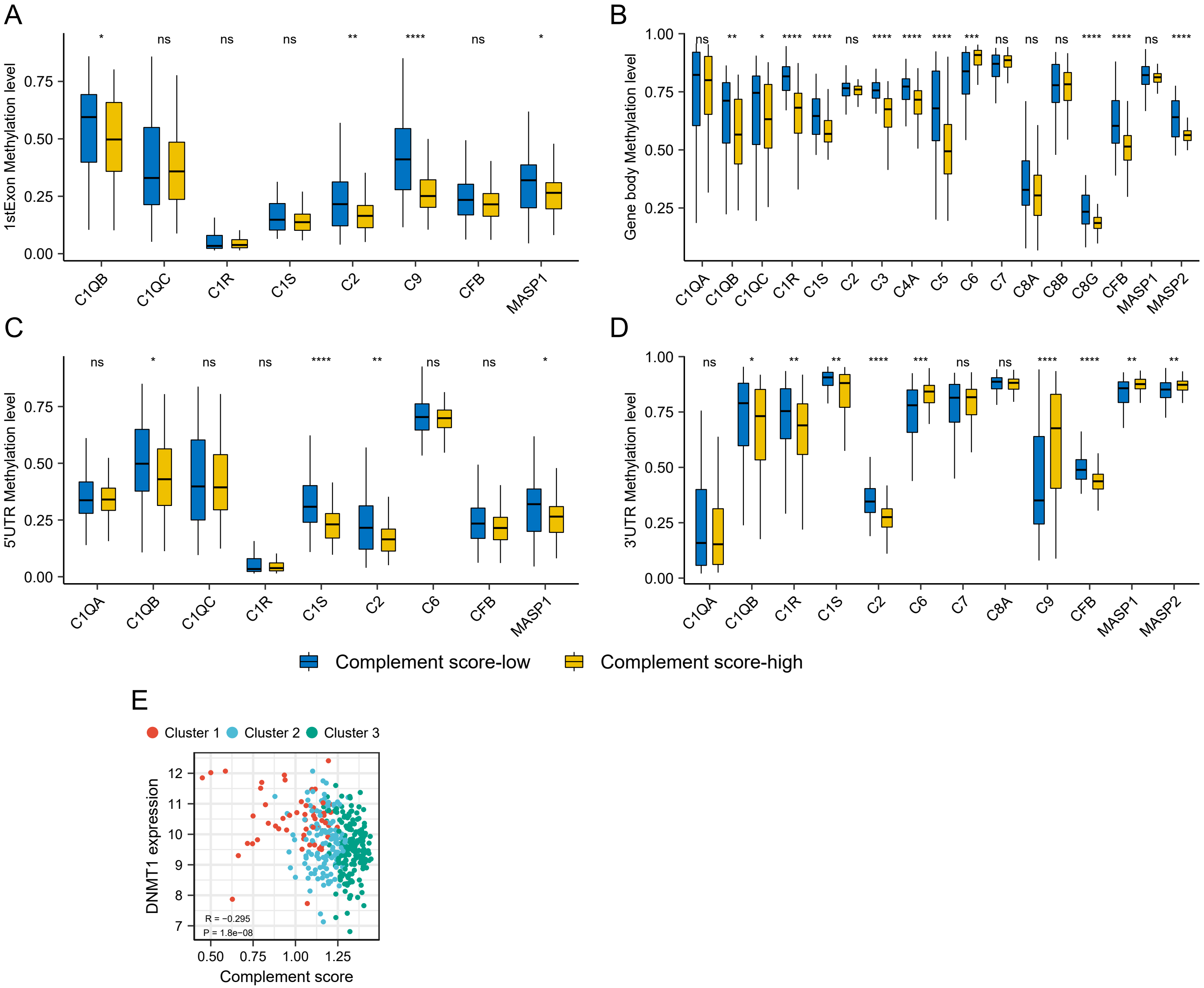

Supplementary Figure S8. Related to Figure 4. A to D, comparisons of DNA methylation levels of the promoter regions of 1stExon, gene body, 5'UTR, and 3'UTR. E, scatter plot of the correlation between the complement scores and the expression of DNA methyltransferase DNMT1.

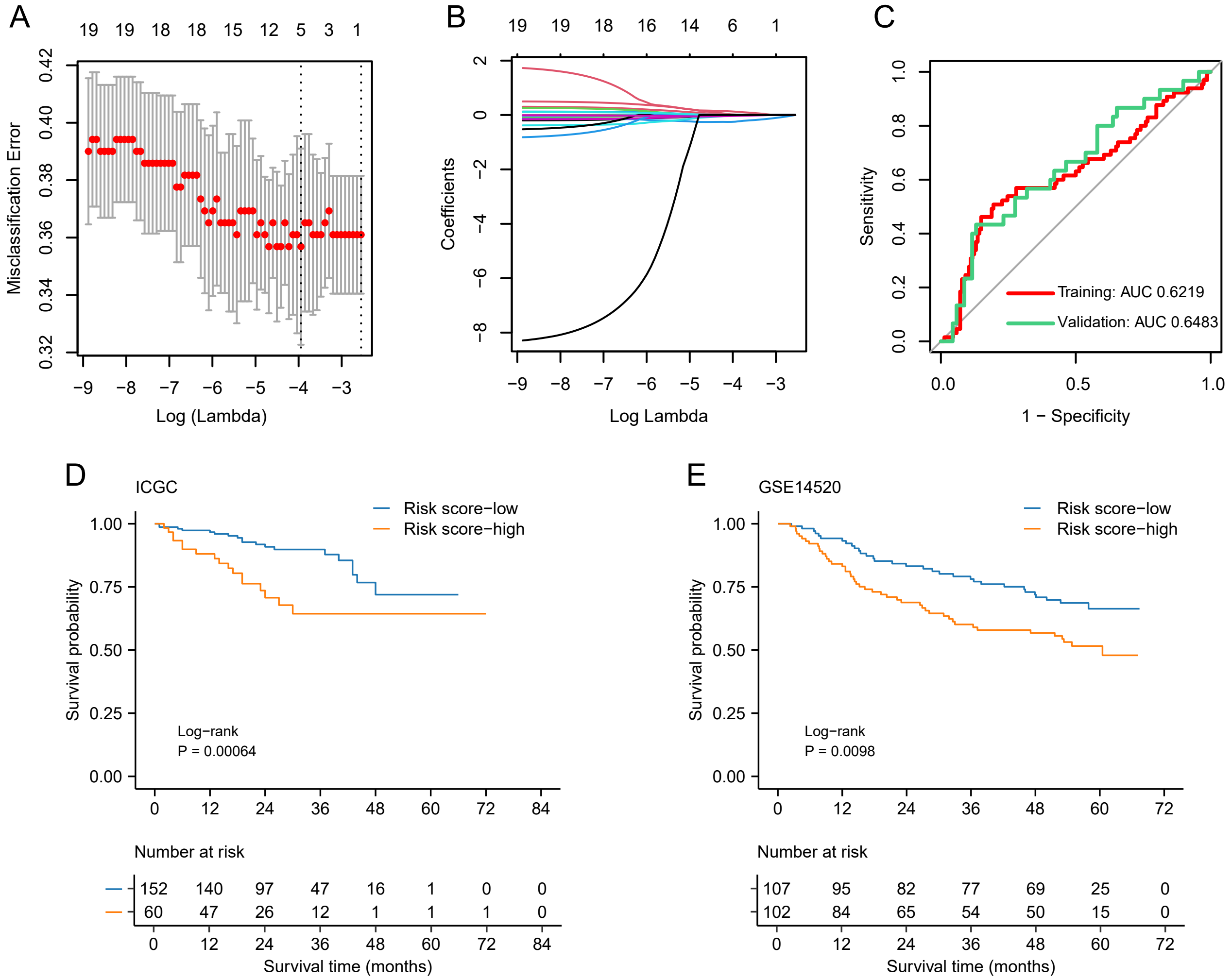

Supplementary Figure S9. Related to Figure 5. A, tuning the parameter selection in LASSO regression model in the TCGA training cohort. B, LASSO coefficient profiles of the training cohort. C, ROC curves based on the six-gene signature. D and E, Kaplan-Meier survival curves based on the risk scores in the datasets of ICGC and GSE14520.

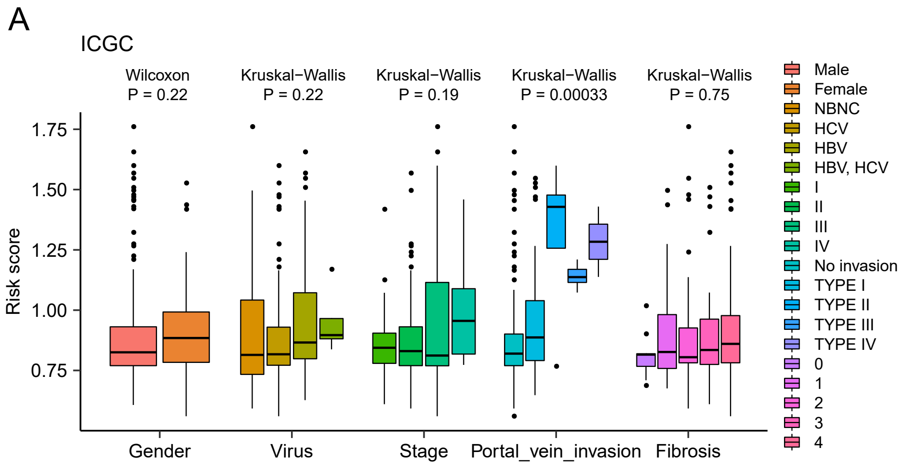

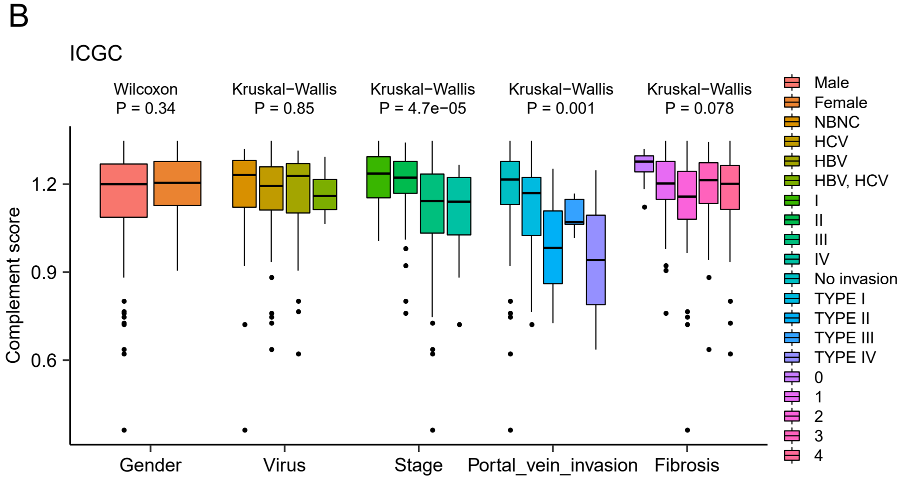

Supplementary Figure S10. Related to Figure 6. Distribution of the risk scores and complement scores in the ICGC cohort. Patients with portal vein invasion were classified according to the Japan's portal vein invasion classification.

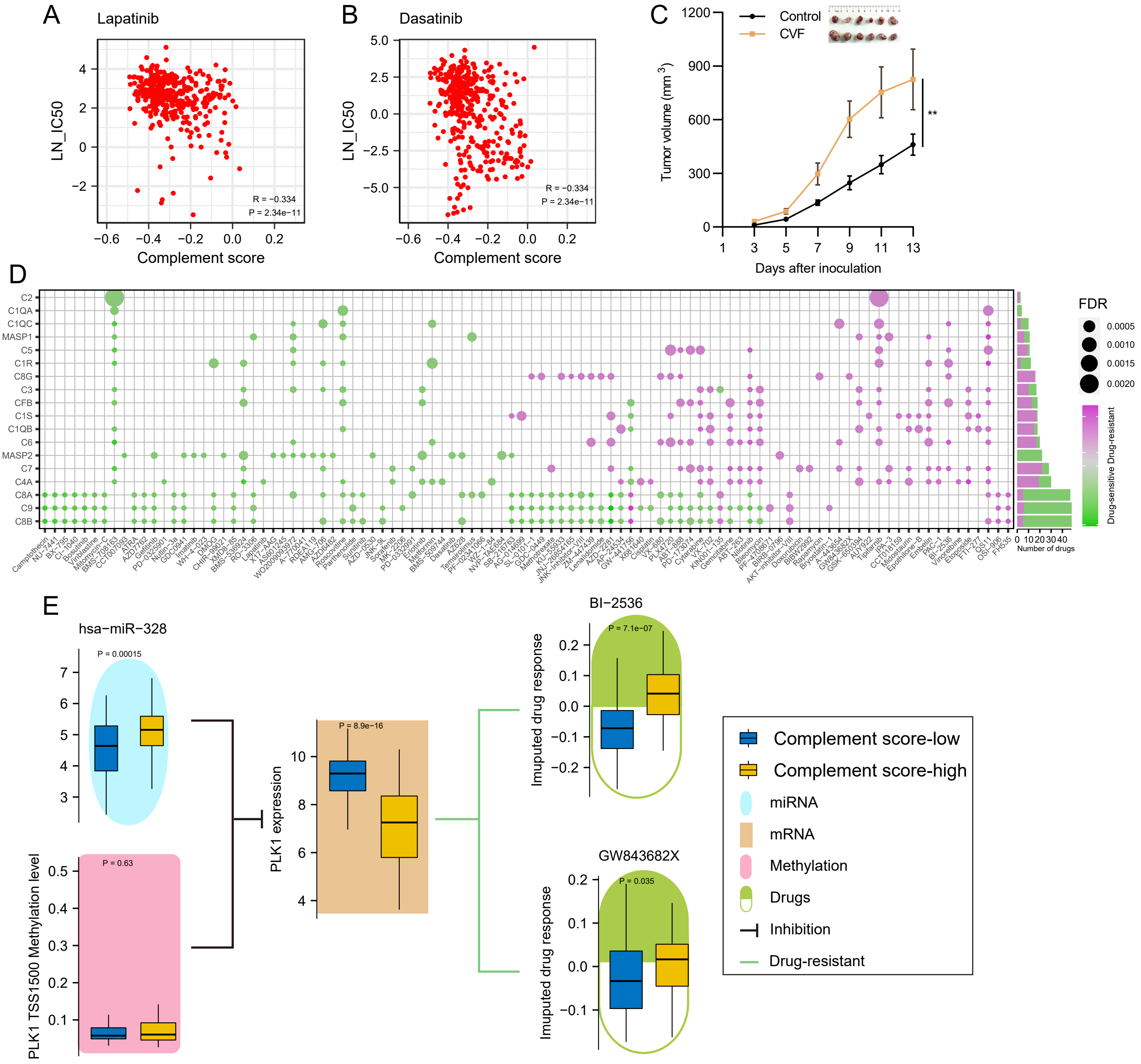

Supplementary Figure S11. Related to Figure 7. A and B, scatter plots between complement scores and the natural logarithm-transformed IC50 of EGFR-targeting drugs lapatinib and dasatinib in GDSC. C, Tumor growth of Hepa 1-6 cells injected subcutaneously into C57BL/6 mice treated with CVF. n = 6 in each group. Data are represented as mean ± standard error. ***P* < 0.01, two-way ANOVA analysis with Tukey's post-hoc test for multiple comparisons. D, Spearman's correlation between imputed drug response and the expression of 18 complement-associated genes in TCGA patients. The y-axis was ordered by the numbers of significant drugs (FDR < 0.05 and the absolute value of correlation > 0.3). The green dots indicated drug-sensitive, and the purple dots indicated drug-resistant. E, Integrated analysis of PLK1 expression with miRNA expression, DNA methylation, and drug response. PLK1 was downregulated in the complement score-high group, and negatively correlated with the predicted miRNA has-miR-328. And has-miR-328 showed positive correlation with the response to inhibitors BI−2536 and GW843682X targeting PLK1 (drug-resistant).
