# Supplementary material for: Comprehensive analysis of complement-associated molecular features in hepatocellular carcinoma: Complement-associated molecular features in hepatocellular carcinoma
Source: Acta Biochim Biophys Sin (Shanghai). 2022 Aug 2;54(11):1694–707. doi: 10.3724/abbs.2022097 (PMC9828444; doi:10.3724/abbs.2022097)
Supplement: Supplementary_table_3 [file Supplementary_table_3.pdf]

Supplementary Table S3. Enriched KEGG pathways after DEG anslysis between complement score-low and score-high groups

ID	Description	setSize	enrichmer	NES	pvalue	p.adjust	qvalues	rank
hsa05171	Coronavirus disease - COVID-19	224	0.525756	1.996177	0.001724	0.001724	0.008317	1764
hsa01240	Biosynthesis of cofactors	142	0.701085	2.511843	0.001862	0.001862	0.008317	2385
hsa04932	Non-alcoholic fatty liver disease	144	0.539651	1.935498	0.001862	0.001862	0.008317	3972
hsa04976	Bile secretion	85	0.717164	2.407379	0.001905	0.001905	0.008317	842
hsa04610	Complement and coagulation cascades	84	0.887591	2.976841	0.001919	0.001919	0.008317	963
hsa03030	DNA replication	36	-0.59922	-1.79535	0.001927	0.001927	0.008317	4346
hsa05150	Staphylococcus aureus infection	87	0.625928	2.100705	0.001931	0.001931	0.008317	802
hsa01200	Carbon metabolism	112	0.660229	2.290608	0.001934	0.001934	0.008317	1938
hsa04922	Glucagon signaling pathway	102	0.525661	1.802232	0.001942	0.001942	0.008317	1712
hsa03440	Homologous recombination	34	-0.61512	-1.81486	0.001949	0.001949	0.008317	4876
hsa00190	Oxidative phosphorylation	104	0.516102	1.773131	0.001953	0.001953	0.008317	3971
hsa03460	Fanconi anemia pathway	44	-0.59415	-1.84911	0.001961	0.001961	0.008317	4586
hsa04931	Insulin resistance	106	0.542357	1.862283	0.001961	0.001961	0.008317	3075
hsa04146	Peroxisome	82	0.755968	2.504802	0.001965	0.001965	0.008317	2129
hsa01230	Biosynthesis of amino acids	69	0.631702	2.040297	0.001984	0.001984	0.008317	896
hsa04920	Adipocytokine signaling pathway	69	0.590195	1.906237	0.001984	0.001984	0.008317	2463
hsa05204	Chemical carcinogenesis	76	0.843968	2.755506	0.001984	0.001984	0.008317	952
hsa05412	Arrhythmogenic right ventricular cardiomyopathy	77	-0.4606	-1.59033	0.001988	0.001988	0.008317	4862
hsa00220	Arginine biosynthesis	21	0.84483	2.175014	0.001996	0.001996	0.008317	896
hsa00770	Pantothenate and CoA biosynthesis	21	0.788447	2.029855	0.001996	0.001996	0.008317	1546
hsa00982	Drug metabolism - cytochrome P450	66	0.845496	2.704952	0.001996	0.001996	0.008317	1381
hsa00071	Fatty acid degradation	40	0.835141	2.478695	0.002	0.002	0.008317	1853
hsa00340	Histidine metabolism	20	0.803763	2.052206	0.002	0.002	0.008317	1519
hsa00860	Porphyrin and chlorophyll metabolism	40	0.78257	2.322666	0.002	0.002	0.008317	2251
hsa04975	Fat digestion and absorption	40	0.787439	2.337117	0.002	0.002	0.008317	1285
hsa00010	Glycolysis / Gluconeogenesis	65	0.642737	2.053458	0.002004	0.002004	0.008317	1633
hsa00983	Drug metabolism - other enzymes	77	0.764256	2.492039	0.002004	0.002004	0.008317	1989
hsa00260	Glycine, serine and threonine metabolism	39	0.842599	2.483627	0.002008	0.002008	0.008317	1430
hsa00380	Tryptophan metabolism	39	0.841653	2.480839	0.002008	0.002008	0.008317	1397
hsa00640	Propanoate metabolism	33	0.734531	2.086499	0.002008	0.002008	0.008317	1868
hsa00980	Metabolism of xenobiotics by cytochrome P450	72	0.840067	2.71759	0.002008	0.002008	0.008317	1381
hsa03320	PPAR signaling pathway	74	0.741591	2.406573	0.002008	0.002008	0.008317	1569
hsa00020	Citrate cycle (TCA cycle)	30	0.694444	1.93233	0.002016	0.002016	0.008317	2748
hsa00040	Pentose and glucuronate interconversions	30	0.83319	2.318398	0.002016	0.002016	0.008317	1399
hsa00410	beta-Alanine metabolism	30	0.718912	2.000412	0.002016	0.002016	0.008317	1546
hsa04216	Ferroptosis	41	0.646292	1.913139	0.002016	0.002016	0.008317	2150
hsa00830	Retinol metabolism	63	0.85068	2.693962	0.00202	0.00202	0.008317	909
hsa00620	Pyruvate metabolism	38	0.742229	2.16999	0.002033	0.002033	0.008317	1460
hsa00053	Ascorbate and aldarate metabolism	28	0.826443	2.283545	0.002037	0.002037	0.008317	1399

hsa00630	Glyoxylate and dicarboxylate metabolism	28	0.786869	2.174199	0.002037	0.002037	0.008317	1853
hsa00591	Linoleic acid metabolism	27	0.78162	2.139269	0.002053	0.002053	0.008317	1343
hsa00650	Butanoate metabolism	27	0.776222	2.124496	0.002053	0.002053	0.008317	1005
hsa00790	Folate biosynthesis	24	0.769876	2.03183	0.002053	0.002053	0.008317	2442
hsa00140	Steroid hormone biosynthesis	58	0.841056	2.608308	0.002062	0.002062	0.008317	1360
hsa00590	Arachidonic acid metabolism	58	0.65727	2.038344	0.002062	0.002062	0.008317	1343
hsa04979	Cholesterol metabolism	49	0.778131	2.335355	0.002062	0.002062	0.008317	806
hsa00350	Tyrosine metabolism	36	0.792188	2.283594	0.00207	0.00207	0.008317	1007
hsa00561	Glycerolipid metabolism	53	0.593099	1.812777	0.00207	0.00207	0.008317	2315
hsa00310	Lysine degradation	48	0.58088	1.738711	0.002079	0.002079	0.008317	1408
hsa04512	ECM-receptor interaction	86	-0.47006	-1.64648	0.002092	0.002092	0.008317	1840
hsa01212	Fatty acid metabolism	52	0.630536	1.916962	0.002096	0.002096	0.008317	1853
hsa04110	Cell cycle	118	-0.5635	-2.09315	0.002096	0.002096	0.008317	4111
hsa00330	Arginine and proline metabolism	45	0.626668	1.858801	0.002105	0.002105	0.008317	1692
hsa02010	ABC transporters	45	0.62011	1.83935	0.002105	0.002105	0.008317	1693
hsa03040	Spliceosome	117	-0.45031	-1.66755	0.002105	0.002105	0.008317	7733
hsa00120	Primary bile acid biosynthesis	17	0.904993	2.198769	0.00211	0.00211	0.008317	978
hsa00360	Phenylalanine metabolism	17	0.797736	1.938179	0.00211	0.00211	0.008317	1007
hsa00270	Cysteine and methionine metabolism	47	0.690884	2.049867	0.002114	0.002114	0.008317	1440
hsa00280	Valine, leucine and isoleucine degradation	47	0.763501	2.265325	0.002114	0.002114	0.008317	1868
hsa00130	Ubiquinone and other terpenoid-quinone biosynthesis	11	0.804385	1.805507	0.002137	0.002137	0.008317	1463
hsa04390	Hippo signaling pathway	151	-0.44892	-1.71265	0.002198	0.002198	0.008317	4019
hsa04310	Wnt signaling pathway	157	-0.44417	-1.702	0.002212	0.002212	0.008317	3699
hsa05206	MicroRNAs in cancer	160	-0.43226	-1.65813	0.002222	0.002222	0.008317	2255
hsa05168	Herpes simplex virus 1 infection	465	-0.43194	-1.8635	0.00274	0.00274	0.010094	4568
hsa04910	Insulin signaling pathway	137	0.430483	1.539068	0.003731	0.003731	0.013432	1704
hsa05322	Systemic lupus erythematosus	122	0.442244	1.555704	0.003788	0.003788	0.013432	513
hsa04152	AMPK signaling pathway	120	0.463257	1.625173	0.003817	0.003817	0.013432	1644
hsa00250	Alanine, aspartate and glutamate metabolism	37	0.647185	1.88399	0.004032	0.004032	0.013948	962
hsa00480	Glutathione metabolism	56	0.561328	1.738637	0.004082	0.004082	0.013948	2354
hsa05143	African trypanosomiasis	36	0.632408	1.823006	0.004141	0.004141	0.013948	1389
hsa01210	2-Oxocarboxylic acid metabolism	18	0.75703	1.875101	0.004246	0.004246	0.014102	1801
hsa00500	Starch and sucrose metabolism	33	0.635842	1.806165	0.006024	0.006024	0.019458	1943
hsa04614	Renin-angiotensin system	23	0.685468	1.78976	0.006024	0.006024	0.019458	2526
hsa05133	Pertussis	74	0.481729	1.563282	0.008032	0.008032	0.025354	1250
hsa00760	Nicotinate and nicotinamide metabolism	30	0.603929	1.680468	0.008065	0.008065	0.025354	2121
hsa04360	Axon guidance	179	-0.36477	-1.4171	0.008909	0.008909	0.027639	3620
hsa04977	Vitamin digestion and absorption	22	0.648898	1.671636	0.010183	0.010183	0.031183	1544
hsa05410	Hypertrophic cardiomyopathy	90	-0.42461	-1.50024	0.010417	0.010417	0.031352	4862
hsa04913	Ovarian steroidogenesis	50	0.554221	1.664627	0.010504	0.010504	0.031352	1521
hsa05414	Dilated cardiomyopathy	95	-0.40543	-1.44471	0.014315	0.014315	0.042191	4862

hsa05320	Autoimmune thyroid disease	50	0.537508	1.61443	0.014706	0.014706	0.042809	4131
hsa04550	Signaling pathways regulating pluripotency of stem cells	140	-0.37119	-1.4146	0.016985	0.016985	0.04884	3609
hsa00051	Fructose and mannose metabolism	30	0.575505	1.601376	0.018145	0.018145	0.051547	473
hsa04218	Cellular senescence	153	-0.35955	-1.37175	0.019824	0.019824	0.055646	4598
hsa00592	alpha-Linolenic acid metabolism	23	0.608509	1.588821	0.022088	0.022088	0.06056	1343
hsa01040	Biosynthesis of unsaturated fatty acids	23	0.60949	1.591381	0.022088	0.022088	0.06056	1284
hsa00061	Fatty acid biosynthesis	17	0.660945	1.605831	0.023207	0.023207	0.062895	2868
hsa03015	mRNA surveillance pathway	92	-0.40149	-1.42739	0.024691	0.024691	0.066159	8669
hsa04714	Thermogenesis	191	0.355252	1.332214	0.026549	0.026549	0.070079	3796
hsa04923	Regulation of lipolysis in adipocytes	54	0.471047	1.447335	0.026749	0.026749	0.070079	2476
hsa00240	Pyrimidine metabolism	52	0.472359	1.43607	0.02935	0.02935	0.076049	2011
hsa05165	Human papillomavirus infection	328	-0.2994	-1.24835	0.033592	0.033592	0.086093	4631
hsa00450	Selenocompound metabolism	14	0.679445	1.580836	0.034783	0.034783	0.087742	1839
hsa05222	Small cell lung cancer	92	-0.39332	-1.39834	0.034979	0.034979	0.087742	2141
hsa05310	Asthma	28	0.552206	1.5258	0.03666	0.03666	0.09077	4131
hsa00072	Synthesis and degradation of ketone bodies	10	0.729474	1.591064	0.036957	0.036957	0.09077	2331
hsa00512	Mucin type O-glycan biosynthesis	26	-0.52463	-1.45155	0.037549	0.037549	0.091276	3205
hsa04392	Hippo signaling pathway - multiple species	25	-0.52101	-1.45254	0.040619	0.040619	0.09773	2370
hsa04061	Viral protein interaction with cytokine and cytokine receptor	95	0.408394	1.386832	0.042885	0.042885	0.10214	2151
hsa05012	Parkinson disease	217	0.335412	1.272088	0.048027	0.048027	0.113244	4016
hsa04540	Gap junction	84	-0.38735	-1.34997	0.049896	0.049896	0.115343	4934
hsa04742	Taste transduction	84	-0.3882	-1.35291	0.049896	0.049896	0.115343	4313
