# Supplementary material for: Comprehensive analysis of complement-associated molecular features in hepatocellular carcinoma: Complement-associated molecular features in hepatocellular carcinoma
Source: Acta Biochim Biophys Sin (Shanghai). 2022 Aug 2;54(11):1694–707. doi: 10.3724/abbs.2022097 (PMC9828444; doi:10.3724/abbs.2022097)
Supplement: Supplementary_table_4 [file Supplementary_table_4.pdf]

Supplementary Table S4. Enriched GO terms after DEG anslysis between complement score-low and score-high groups

ONTOLOGY	ID	Description	setSize	enrichmer	NES	pvalue	p.adjust	qvalues
MF	GO:0016491	oxidoreductase activity	689	0.651266	2.79641	0.000168	0.000168	0.001229
MF	GO:0008289	lipid binding	680	0.388173	1.665066	0.000168	0.000168	0.001229
BP	GO:0006954	inflammatory response	669	0.463145	1.984298	0.000169	0.000169	0.001229
BP	GO:0030162	regulation of proteolysis	669	0.361579	1.549147	0.000169	0.000169	0.001229
BP	GO:0044283	small molecule biosynthetic process	664	0.581226	2.488995	0.000169	0.000169	0.001229
BP	GO:0032870	cellular response to hormone stimulus	656	0.364122	1.558202	0.000169	0.000169	0.001229
BP	GO:0008610	lipid biosynthetic process	643	0.51469	2.19894	0.00017	0.00017	0.001229
BP	GO:0009611	response to wounding	615	0.398919	1.700615	0.00017	0.00017	0.001229
CC	GO:0000323	lytic vacuole	615	0.333217	1.420523	0.00017	0.00017	0.001229
BP	GO:0055065	metal ion homeostasis	614	0.334056	1.423992	0.00017	0.00017	0.001229
CC	GO:0005764	lysosome	614	0.332258	1.416327	0.00017	0.00017	0.001229
BP	GO:0032787	monocarboxylic acid metabolic process	612	0.672171	2.864505	0.00017	0.00017	0.001229
CC	GO:0005740	mitochondrial envelope	612	0.458723	1.95488	0.00017	0.00017	0.001229
BP	GO:0031347	regulation of defense response	626	0.342332	1.45983	0.000171	0.000171	0.001229
BP	GO:0006820	anion transport	608	0.455341	1.938969	0.000171	0.000171	0.001229
BP	GO:0050778	positive regulation of immune response	605	0.400341	1.703709	0.000171	0.000171	0.001229
BP	GO:0071417	cellular response to organonitrogen compound	605	0.335914	1.42953	0.000171	0.000171	0.001229
CC	GO:0031966	mitochondrial membrane	579	0.459692	1.949378	0.000172	0.000172	0.001229
BP	GO:1901361	organic cyclic compound catabolic process	585	0.45119	1.914469	0.000172	0.000172	0.001229
BP	GO:0005975	carbohydrate metabolic process	577	0.450211	1.908385	0.000173	0.000173	0.001229
BP	GO:0009617	response to bacterium	567	0.472322	2.000511	0.000173	0.000173	0.001229
BP	GO:0019439	aromatic compound catabolic process	552	0.404269	1.709798	0.000173	0.000173	0.001229
MF	GO:0070011	peptidase activity, acting on L-amino acid peptides	533	0.349805	1.474603	0.000173	0.000173	0.001229
BP	GO:0055086	nucleobase-containing small molecule metabolic process	556	0.49307	2.085673	0.000173	0.000173	0.001229
BP	GO:0044270	cellular nitrogen compound catabolic process	537	0.369889	1.560651	0.000173	0.000173	0.001229
MF	GO:0008233	peptidase activity	554	0.35276	1.491852	0.000173	0.000173	0.001229
BP	GO:0046700	heterocycle catabolic process	536	0.372843	1.572484	0.000173	0.000173	0.001229
BP	GO:1901615	organic hydroxy compound metabolic process	510	0.59379	2.493584	0.000173	0.000173	0.001229
BP	GO:0042060	wound healing	511	0.431513	1.812018	0.000174	0.000174	0.001229
BP	GO:0010817	regulation of hormone levels	503	0.516352	2.166392	0.000174	0.000174	0.001229
CC	GO:0031012	extracellular matrix	503	0.434405	1.822578	0.000174	0.000174	0.001229
BP	GO:0017144	drug metabolic process	527	0.652741	2.74791	0.000174	0.000174	0.001229
BP	GO:0051259	protein complex oligomerization	514	0.387296	1.627334	0.000174	0.000174	0.001229
BP	GO:0090407	organophosphate biosynthetic process	528	0.384585	1.619199	0.000174	0.000174	0.001229
BP	GO:0010035	response to inorganic substance	521	0.433932	1.824871	0.000174	0.000174	0.001229
BP	GO:0009636	response to toxic substance	494	0.47463	1.988574	0.000175	0.000175	0.001229
BP	GO:1901652	response to peptide	484	0.379243	1.586598	0.000175	0.000175	0.001229

BP	GO:0009991	response to extracellular stimulus	498	0.430086	1.801761	0.000175	0.000175	0.001229
BP	GO:0006753	nucleoside phosphate metabolic process	481	0.481005	2.010883	0.000175	0.000175	0.001229
BP	GO:0006091	generation of precursor metabolites and energy	471	0.536247	2.237519	0.000176	0.000176	0.001229
MF	GO:0048037	cofactor binding	469	0.6681	2.786416	0.000176	0.000176	0.001229
BP	GO:0031667	response to nutrient levels	469	0.434377	1.811639	0.000176	0.000176	0.001229
BP	GO:0050878	regulation of body fluid levels	469	0.433847	1.809427	0.000176	0.000176	0.001229
BP	GO:0009117	nucleotide metabolic process	474	0.481179	2.008106	0.000176	0.000176	0.001229
BP	GO:0015711	organic anion transport	470	0.516621	2.155147	0.000176	0.000176	0.001229
CC	GO:0019866	organelle inner membrane	432	0.483499	2.000985	0.000178	0.000178	0.001229
BP	GO:0072521	purine-containing compound metabolic process	410	0.499849	2.061087	0.000179	0.000179	0.001229
BP	GO:0051186	cofactor metabolic process	418	0.639689	2.641082	0.000179	0.000179	0.001229
BP	GO:0002253	activation of immune response	418	0.461867	1.906911	0.000179	0.000179	0.001229
BP	GO:0051346	negative regulation of hydrolase activity	413	0.441147	1.819822	0.000179	0.000179	0.001229
BP	GO:0052547	regulation of peptidase activity	413	0.410677	1.694128	0.000179	0.000179	0.001229
BP	GO:0043434	response to peptide hormone	411	0.423051	1.743944	0.000179	0.000179	0.001229
BP	GO:0052548	regulation of endopeptidase activity	387	0.422733	1.73294	0.000179	0.000179	0.001229
BP	GO:0006163	purine nucleotide metabolic process	382	0.474421	1.942101	0.000179	0.000179	0.001229
BP	GO:0044282	small molecule catabolic process	419	0.696171	2.874015	0.000179	0.000179	0.001229
CC	GO:0005759	mitochondrial matrix	419	0.585254	2.416116	0.000179	0.000179	0.001229
BP	GO:0046486	glycerolipid metabolic process	386	0.494378	2.02596	0.000179	0.000179	0.001229
CC	GO:0062023	collagen-containing extracellular matrix	391	0.463689	1.902343	0.000179	0.000179	0.001229
BP	GO:0062012	regulation of small molecule metabolic process	390	0.474779	1.947207	0.000179	0.000179	0.001229
BP	GO:0002250	adaptive immune response	398	0.440508	1.809242	0.00018	0.00018	0.001229
BP	GO:0002697	regulation of immune effector process	378	0.50211	2.052635	0.00018	0.00018	0.001229
CC	GO:0005743	mitochondrial inner membrane	379	0.532962	2.178533	0.00018	0.00018	0.001229
BP	GO:0019693	ribose phosphate metabolic process	374	0.474735	1.938223	0.00018	0.00018	0.001229
CC	GO:0045177	apical part of cell	359	0.421025	1.713906	0.00018	0.00018	0.001229
BP	GO:1901653	cellular response to peptide	359	0.406342	1.654136	0.00018	0.00018	0.001229
BP	GO:0010876	lipid localization	371	0.602379	2.457309	0.000181	0.000181	0.001229
BP	GO:0048545	response to steroid hormone	362	0.41778	1.700985	0.000181	0.000181	0.001229
BP	GO:0006869	lipid transport	338	0.601545	2.436547	0.000181	0.000181	0.001229
BP	GO:0051260	protein homooligomerization	338	0.417617	1.69155	0.000181	0.000181	0.001229
BP	GO:0019216	regulation of lipid metabolic process	369	0.548278	2.235155	0.000181	0.000181	0.001229
BP	GO:0010038	response to metal ion	349	0.461164	1.871549	0.000181	0.000181	0.001229
MF	GO:0004857	enzyme inhibitor activity	341	0.464366	1.881133	0.000181	0.000181	0.001229
BP	GO:0009259	ribonucleotide metabolic process	364	0.481378	1.960417	0.000181	0.000181	0.001229
BP	GO:0006066	alcohol metabolic process	351	0.604599	2.454611	0.000181	0.000181	0.001229
BP	GO:0006631	fatty acid metabolic process	352	0.696304	2.827197	0.000182	0.000182	0.001229
BP	GO:0009150	purine ribonucleotide metabolic process	350	0.480865	1.951583	0.000182	0.000182	0.001229

BP	GO:0032102	negative regulation of response to external stimulus	346	0.42186	1.710116	0.000182	0.000182	0.001229
BP	GO:0001505	regulation of neurotransmitter levels	335	0.413392	1.671784	0.000182	0.000182	0.001229
BP	GO:0006520	cellular amino acid metabolic process	309	0.670815	2.693728	0.000182	0.000182	0.001229
BP	GO:0016053	organic acid biosynthetic process	325	0.668116	2.69508	0.000182	0.000182	0.001229
BP	GO:0045861	negative regulation of proteolysis	325	0.460536	1.857732	0.000182	0.000182	0.001229
BP	GO:0008202	steroid metabolic process	304	0.691144	2.770616	0.000182	0.000182	0.001229
CC	GO:0034774	secretory granule lumen	304	0.505989	2.028378	0.000182	0.000182	0.001229
BP	GO:0006790	sulfur compound metabolic process	348	0.571068	2.31585	0.000182	0.000182	0.001229
BP	GO:0043687	post-translational protein modification	348	0.400389	1.623698	0.000182	0.000182	0.001229
MF	GO:0022804	active transmembrane transporter activity	332	0.421282	1.702035	0.000182	0.000182	0.001229
MF	GO:0033218	amide binding	326	0.429617	1.732465	0.000182	0.000182	0.001229
CC	GO:0060205	cytoplasmic vesicle lumen	308	0.510272	2.047511	0.000182	0.000182	0.001229
BP	GO:0046394	carboxylic acid biosynthetic process	324	0.667439	2.691154	0.000182	0.000182	0.001229
CC	GO:0031983	vesicle lumen	310	0.515404	2.068913	0.000182	0.000182	0.001229
BP	GO:0050727	regulation of inflammatory response	319	0.411245	1.655526	0.000183	0.000183	0.001229
BP	GO:0016042	lipid catabolic process	312	0.6608	2.654246	0.000183	0.000183	0.001229
BP	GO:0007596	blood coagulation	312	0.509726	2.047424	0.000183	0.000183	0.001229
CC	GO:0005788	endoplasmic reticulum lumen	289	0.469852	1.873971	0.000183	0.000183	0.001229
BP	GO:0046942	carboxylic acid transport	318	0.521406	2.097861	0.000183	0.000183	0.001229
BP	GO:0071375	cellular response to peptide hormone stimulus	302	0.438658	1.756098	0.000183	0.000183	0.001229
BP	GO:0007599	hemostasis	317	0.507432	2.040682	0.000183	0.000183	0.001229
BP	GO:0015849	organic acid transport	320	0.51802	2.085868	0.000183	0.000183	0.001229
MF	GO:0008509	anion transmembrane transporter activity	320	0.411587	1.657304	0.000183	0.000183	0.001229
BP	GO:0050817	coagulation	316	0.50571	2.03216	0.000183	0.000183	0.001229
BP	GO:0009410	response to xenobiotic stimulus	282	0.677603	2.694641	0.000184	0.000184	0.001229
MF	GO:0050662	coenzyme binding	269	0.704309	2.787271	0.000184	0.000184	0.001229
BP	GO:0044262	cellular carbohydrate metabolic process	261	0.503725	1.985714	0.000185	0.000185	0.001229
BP	GO:0005996	monosaccharide metabolic process	263	0.602935	2.378529	0.000185	0.000185	0.001229
BP	GO:0072593	reactive oxygen species metabolic process	251	0.490754	1.929209	0.000186	0.000186	0.001229
BP	GO:0015980	energy derivation by oxidation of organic compounds	255	0.549056	2.159232	0.000186	0.000186	0.001229
BP	GO:0032868	response to insulin	253	0.449823	1.768923	0.000186	0.000186	0.001229
BP	GO:0002460	adaptive immune response based on somatic recombination o	249	0.476245	1.870116	0.000186	0.000186	0.001229
BP	GO:0010466	negative regulation of peptidase activity	238	0.507916	1.988175	0.000186	0.000186	0.001229
BP	GO:0002449	lymphocyte mediated immunity	238	0.475413	1.860942	0.000186	0.000186	0.001229
BP	GO:1901617	organic hydroxy compound biosynthetic process	248	0.534364	2.097182	0.000187	0.000187	0.001229
BP	GO:0006732	coenzyme metabolic process	237	0.670638	2.623763	0.000187	0.000187	0.001229
BP	GO:0016054	organic acid catabolic process	258	0.770683	3.031166	0.000187	0.000187	0.001229
BP	GO:0046395	carboxylic acid catabolic process	258	0.770683	3.031166	0.000187	0.000187	0.001229
BP	GO:0006898	receptor-mediated endocytosis	243	0.477307	1.870562	0.000187	0.000187	0.001229

BP	GO:0006959	humoral immune response	225	0.672499	2.615864	0.000187	0.000187	0.001229
BP	GO:0042445	hormone metabolic process	225	0.671781	2.613069	0.000187	0.000187	0.001229
BP	GO:0042742	defense response to bacterium	226	0.498922	1.940757	0.000187	0.000187	0.001229
BP	GO:0009165	nucleotide biosynthetic process	223	0.438331	1.702607	0.000187	0.000187	0.001229
BP	GO:0019318	hexose metabolic process	224	0.557497	2.166431	0.000187	0.000187	0.001229
BP	GO:0097237	cellular response to toxic substance	221	0.481523	1.868112	0.000187	0.000187	0.001229
BP	GO:1901293	nucleoside phosphate biosynthetic process	227	0.441943	1.719195	0.000187	0.000187	0.001229
MF	GO:0008514	organic anion transmembrane transporter activity	199	0.502142	1.924912	0.000187	0.000187	0.001229
BP	GO:0042180	cellular ketone metabolic process	228	0.62331	2.425467	0.000187	0.000187	0.001229
MF	GO:1901681	sulfur compound binding	228	0.565879	2.201986	0.000187	0.000187	0.001229
BP	GO:0010951	negative regulation of endopeptidase activity	228	0.516095	2.008264	0.000187	0.000187	0.001229
BP	GO:0045017	glycerolipid biosynthetic process	234	0.444244	1.733542	0.000188	0.000188	0.001229
BP	GO:0007584	response to nutrient	210	0.461265	1.778858	0.000188	0.000188	0.001229
BP	GO:0016051	carbohydrate biosynthetic process	198	0.476478	1.825718	0.000188	0.000188	0.001229
MF	GO:0000217	DNA secondary structure binding	22	-0.7251	-1.92206	0.000188	0.000188	0.001229
BP	GO:0051188	cofactor biosynthetic process	207	0.563092	2.166376	0.000188	0.000188	0.001229
MF	GO:0061134	peptidase regulator activity	207	0.518344	1.994219	0.000188	0.000188	0.001229
MF	GO:0005539	glycosaminoglycan binding	207	0.500382	1.925113	0.000188	0.000188	0.001229
BP	GO:0034341	response to interferon-gamma	192	0.514106	1.962821	0.000188	0.000188	0.001229
BP	GO:0072330	monocarboxylic acid biosynthetic process	216	0.675389	2.612356	0.000188	0.000188	0.001229
BP	GO:0030258	lipid modification	218	0.617357	2.389567	0.000188	0.000188	0.001229
BP	GO:0015850	organic hydroxy compound transport	235	0.545898	2.129939	0.000188	0.000188	0.001229
CC	GO:0000780	condensed nuclear chromosome, centromeric region	20	-0.80767	-2.09073	0.000188	0.000188	0.001229
CC	GO:0044815	DNA packaging complex	23	-0.73092	-1.95623	0.000188	0.000188	0.001229
BP	GO:0022616	DNA strand elongation	23	-0.73725	-1.97318	0.000188	0.000188	0.001229
BP	GO:0044242	cellular lipid catabolic process	208	0.697337	2.684456	0.000188	0.000188	0.001229
CC	GO:0098798	mitochondrial protein complex	203	0.514964	1.975804	0.000188	0.000188	0.001229
BP	GO:0031572	G2 DNA damage checkpoint	24	-0.72279	-1.95351	0.000189	0.000189	0.001229
MF	GO:0043177	organic acid binding	190	0.595537	2.267969	0.000189	0.000189	0.001229
BP	GO:0044272	sulfur compound biosynthetic process	179	0.489393	1.852238	0.000189	0.000189	0.001229
BP	GO:1901605	alpha-amino acid metabolic process	184	0.752632	2.854634	0.00019	0.00019	0.001229
BP	GO:0060669	embryonic placenta morphogenesis	25	-0.72382	-1.97358	0.00019	0.00019	0.001229
BP	GO:0046890	regulation of lipid biosynthetic process	177	0.566143	2.13949	0.00019	0.00019	0.001229
BP	GO:0006694	steroid biosynthetic process	178	0.639396	2.417565	0.00019	0.00019	0.001229
MF	GO:0031406	carboxylic acid binding	178	0.599928	2.268339	0.00019	0.00019	0.001229
BP	GO:1901568	fatty acid derivative metabolic process	153	0.67326	2.499802	0.00019	0.00019	0.001229
BP	GO:0006006	glucose metabolic process	187	0.590138	2.24177	0.00019	0.00019	0.001229
BP	GO:0016052	carbohydrate catabolic process	187	0.466963	1.773862	0.00019	0.00019	0.001229
MF	GO:0016825	hydrolase activity, acting on acid phosphorus-nitrogen bonds	172	0.567161	2.135999	0.00019	0.00019	0.001229

MF	GO:0017171	serine hydrolase activity	172	0.567161	2.135999	0.00019	0.00019	0.001229
MF	GO:0061135	endopeptidase regulator activity	172	0.554046	2.086608	0.00019	0.00019	0.001229
BP	GO:0015718	monocarboxylic acid transport	152	0.63372	2.351005	0.00019	0.00019	0.001229
MF	GO:0008201	heparin binding	152	0.556422	2.06424	0.00019	0.00019	0.001229
MF	GO:0030414	peptidase inhibitor activity	173	0.557029	2.098898	0.000191	0.000191	0.001229
BP	GO:0071466	cellular response to xenobiotic stimulus	174	0.770836	2.906132	0.000191	0.000191	0.001229
BP	GO:0006575	cellular modified amino acid metabolic process	174	0.585266	2.206514	0.000191	0.000191	0.001229
BP	GO:0071346	cellular response to interferon-gamma	174	0.519721	1.959401	0.000191	0.000191	0.001229
BP	GO:0016125	sterol metabolic process	150	0.655025	2.427217	0.000191	0.000191	0.001229
BP	GO:0045333	cellular respiration	169	0.553915	2.081339	0.000191	0.000191	0.001229
BP	GO:0060706	cell differentiation involved in embryonic placenta developmer	26	-0.73657	-2.02678	0.000191	0.000191	0.001229
BP	GO:0010971	positive regulation of G2/M transition of mitotic cell cycle	26	-0.75114	-2.06687	0.000191	0.000191	0.001229
BP	GO:0008608	attachment of spindle microtubules to kinetochore	26	-0.78881	-2.17053	0.000191	0.000191	0.001229
BP	GO:0022900	electron transport chain	164	0.610618	2.286656	0.000191	0.000191	0.001229
MF	GO:0008236	serine-type peptidase activity	168	0.560693	2.10544	0.000191	0.000191	0.001229
BP	GO:0031960	response to corticosteroid	156	0.52648	1.956009	0.000192	0.000192	0.001229
BP	GO:0046165	alcohol biosynthetic process	161	0.49042	1.829182	0.000192	0.000192	0.001229
BP	GO:0006270	DNA replication initiation	28	-0.74934	-2.09669	0.000192	0.000192	0.001229
MF	GO:0005342	organic acid transmembrane transporter activity	146	0.544356	2.010377	0.000192	0.000192	0.001229
MF	GO:0046943	carboxylic acid transmembrane transporter activity	146	0.544356	2.010377	0.000192	0.000192	0.001229
BP	GO:0014074	response to purine-containing compound	146	0.479312	1.77016	0.000192	0.000192	0.001229
MF	GO:0016829	lyase activity	158	0.579039	2.154758	0.000192	0.000192	0.001229
BP	GO:1903034	regulation of response to wounding	158	0.571672	2.127342	0.000192	0.000192	0.001229
BP	GO:1902652	secondary alcohol metabolic process	147	0.661495	2.443629	0.000192	0.000192	0.001229
BP	GO:0006633	fatty acid biosynthetic process	147	0.656183	2.424008	0.000192	0.000192	0.001229
MF	GO:0004252	serine-type endopeptidase activity	147	0.603876	2.230779	0.000192	0.000192	0.001229
MF	GO:0004866	endopeptidase inhibitor activity	166	0.566577	2.123499	0.000192	0.000192	0.001229
BP	GO:0010565	regulation of cellular ketone metabolic process	166	0.555665	2.082603	0.000192	0.000192	0.001229
BP	GO:0051262	protein tetramerization	143	0.488444	1.799182	0.000192	0.000192	0.001229
MF	GO:0016705	oxidoreductase activity, acting on paired donors, with incorpor	141	0.73716	2.706724	0.000193	0.000193	0.001229
MF	GO:0015293	symporter activity	141	0.474483	1.74222	0.000193	0.000193	0.001229
BP	GO:0032506	cytokinetic process	38	-0.66309	-1.98944	0.000193	0.000193	0.001229
BP	GO:1905819	negative regulation of chromosome separation	38	-0.69894	-2.09702	0.000193	0.000193	0.001229
BP	GO:0033048	negative regulation of mitotic sister chromatid segregation	40	-0.68033	-2.06396	0.000193	0.000193	0.001229
BP	GO:0007094	mitotic spindle assembly checkpoint	32	-0.72842	-2.10213	0.000193	0.000193	0.001229
BP	GO:0031577	spindle checkpoint	32	-0.72842	-2.10213	0.000193	0.000193	0.001229
BP	GO:0071173	spindle assembly checkpoint	32	-0.72842	-2.10213	0.000193	0.000193	0.001229
BP	GO:0071174	mitotic spindle checkpoint	32	-0.72842	-2.10213	0.000193	0.000193	0.001229
BP	GO:1902751	positive regulation of cell cycle G2/M phase transition	29	-0.73076	-2.056	0.000193	0.000193	0.001229

BP	GO:0051384	response to glucocorticoid	142	0.559971	2.057719	0.000193	0.000193	0.001229
BP	GO:0042133	neurotransmitter metabolic process	140	0.63414	2.323766	0.000193	0.000193	0.001229
BP	GO:0006090	pyruvate metabolic process	140	0.492172	1.803536	0.000193	0.000193	0.001229
BP	GO:2000816	negative regulation of mitotic sister chromatid separation	37	-0.69929	-2.08829	0.000194	0.000194	0.001229
BP	GO:0033046	negative regulation of sister chromatid segregation	41	-0.68957	-2.09965	0.000194	0.000194	0.001229
BP	GO:0007080	mitotic metaphase plate congression	41	-0.76457	-2.32802	0.000194	0.000194	0.001229
MF	GO:0017147	Wnt-protein binding	35	-0.66862	-1.97074	0.000194	0.000194	0.001229
BP	GO:1902100	negative regulation of metaphase/anaphase transition of cell c	35	-0.70875	-2.08902	0.000194	0.000194	0.001229
MF	GO:0005506	iron ion binding	137	0.709573	2.590185	0.000194	0.000194	0.001229
BP	GO:0008203	cholesterol metabolic process	137	0.665268	2.428457	0.000194	0.000194	0.001229
BP	GO:0006720	isoprenoid metabolic process	136	0.725463	2.64558	0.000194	0.000194	0.001229
BP	GO:0061041	regulation of wound healing	131	0.634832	2.303947	0.000194	0.000194	0.001229
BP	GO:0009108	coenzyme biosynthetic process	130	0.605835	2.195882	0.000194	0.000194	0.001229
BP	GO:0001889	liver development	130	0.526407	1.907992	0.000194	0.000194	0.001229
BP	GO:0033865	nucleoside bisphosphate metabolic process	132	0.654802	2.379138	0.000194	0.000194	0.001229
BP	GO:0033875	ribonucleoside bisphosphate metabolic process	132	0.654802	2.379138	0.000194	0.000194	0.001229
BP	GO:0034032	purine nucleoside bisphosphate metabolic process	132	0.654802	2.379138	0.000194	0.000194	0.001229
BP	GO:0009308	amine metabolic process	132	0.607375	2.206817	0.000194	0.000194	0.001229
BP	GO:0007586	digestion	132	0.49493	1.798262	0.000194	0.000194	0.001229
BP	GO:0046683	response to organophosphorus	132	0.479759	1.743142	0.000194	0.000194	0.001229
MF	GO:0016614	oxidoreductase activity, acting on CH-OH group of donors	125	0.737219	2.659802	0.000194	0.000194	0.001229
CC	GO:0051233	spindle midzone	33	-0.6733	-1.95368	0.000194	0.000194	0.001229
BP	GO:0051985	negative regulation of chromosome segregation	42	-0.68925	-2.11178	0.000194	0.000194	0.001229
BP	GO:0061008	hepaticobiliary system development	133	0.52252	1.899601	0.000194	0.000194	0.001229
BP	GO:0045841	negative regulation of mitotic metaphase/anaphase transition	34	-0.70912	-2.07288	0.000194	0.000194	0.001229
BP	GO:0072329	monocarboxylic acid catabolic process	124	0.78801	2.838785	0.000194	0.000194	0.001229
BP	GO:0098754	detoxification	124	0.683118	2.460913	0.000194	0.000194	0.001229
BP	GO:0044106	cellular amine metabolic process	124	0.596897	2.150306	0.000194	0.000194	0.001229
MF	GO:0046906	tetrapyrrole binding	135	0.713453	2.598407	0.000195	0.000195	0.001229
BP	GO:0042737	drug catabolic process	134	0.763263	2.776411	0.000195	0.000195	0.001229
BP	GO:0045834	positive regulation of lipid metabolic process	134	0.616685	2.243226	0.000195	0.000195	0.001229
BP	GO:0006639	acylglycerol metabolic process	114	0.752387	2.677214	0.000195	0.000195	0.001229
MF	GO:0016874	ligase activity	114	0.514265	1.829906	0.000195	0.000195	0.001229
CC	GO:0005777	peroxisome	122	0.722271	2.592681	0.000195	0.000195	0.001229
CC	GO:0042579	microbody	122	0.722271	2.592681	0.000195	0.000195	0.001229
BP	GO:0002576	platelet degranulation	122	0.662784	2.379146	0.000195	0.000195	0.001229
BP	GO:0042157	lipoprotein metabolic process	122	0.552557	1.983472	0.000195	0.000195	0.001229
BP	GO:0006638	neutral lipid metabolic process	115	0.748372	2.663097	0.000195	0.000195	0.001229
MF	GO:0005319	lipid transporter activity	123	0.69205	2.488644	0.000195	0.000195	0.001229

BP	GO:0010675	regulation of cellular carbohydrate metabolic process	123	0.567793	2.041811	0.000195	0.000195	0.001229
BP	GO:1905952	regulation of lipid localization	128	0.602535	2.178444	0.000195	0.000195	0.001229
BP	GO:0062013	positive regulation of small molecule metabolic process	128	0.55161	1.994325	0.000195	0.000195	0.001229
MF	GO:0016616	oxidoreductase activity, acting on the CH-OH group of donors	116	0.729013	2.598022	0.000195	0.000195	0.001229
MF	GO:0052689	carboxylic ester hydrolase activity	116	0.603271	2.149908	0.000195	0.000195	0.001229
BP	GO:0009063	cellular amino acid catabolic process	118	0.797507	2.848428	0.000195	0.000195	0.001229
BP	GO:0006721	terpenoid metabolic process	118	0.743082	2.654041	0.000195	0.000195	0.001229
BP	GO:0034754	cellular hormone metabolic process	126	0.752369	2.715082	0.000195	0.000195	0.001229
BP	GO:0055088	lipid homeostasis	126	0.711198	2.566506	0.000195	0.000195	0.001229
BP	GO:0006766	vitamin metabolic process	126	0.593274	2.140952	0.000195	0.000195	0.001229
BP	GO:0055076	transition metal ion homeostasis	126	0.551085	1.988705	0.000195	0.000195	0.001229
MF	GO:0020037	heme binding	127	0.730456	2.638316	0.000195	0.000195	0.001229
BP	GO:0016999	antibiotic metabolic process	119	0.706794	2.52614	0.000195	0.000195	0.001229
MF	GO:0019842	vitamin binding	121	0.685327	2.456293	0.000195	0.000195	0.001229
MF	GO:0016810	hydrolase activity, acting on carbon-nitrogen (but not peptide)	121	0.551774	1.977621	0.000195	0.000195	0.001229
CC	GO:0072562	blood microparticle	110	0.832455	2.945101	0.000195	0.000195	0.001229
BP	GO:0019218	regulation of steroid metabolic process	111	0.619359	2.193508	0.000196	0.000196	0.001229
BP	GO:0019724	B cell mediated immunity	111	0.606658	2.148528	0.000196	0.000196	0.001229
BP	GO:0016101	diterpenoid metabolic process	108	0.739008	2.605296	0.000196	0.000196	0.001229
BP	GO:0016064	immunoglobulin mediated immune response	108	0.613803	2.163901	0.000196	0.000196	0.001229
BP	GO:0015748	organophosphate ester transport	112	0.585519	2.07568	0.000196	0.000196	0.001229
BP	GO:0006805	xenobiotic metabolic process	120	0.823345	2.945845	0.000196	0.000196	0.001229
BP	GO:0001676	long-chain fatty acid metabolic process	105	0.744679	2.610216	0.000197	0.000197	0.001229
BP	GO:0002526	acute inflammatory response	105	0.721006	2.527237	0.000197	0.000197	0.001229
MF	GO:0009055	electron transfer activity	105	0.663511	2.325708	0.000197	0.000197	0.001229
BP	GO:0046916	cellular transition metal ion homeostasis	105	0.567455	1.989017	0.000197	0.000197	0.001229
BP	GO:0045839	negative regulation of mitotic nuclear division	49	-0.66971	-2.1135	0.000197	0.000197	0.001229
BP	GO:0030071	regulation of mitotic metaphase/anaphase transition	49	-0.70877	-2.23675	0.000197	0.000197	0.001229
BP	GO:0051310	metaphase plate congression	51	-0.70041	-2.23079	0.000197	0.000197	0.001229
BP	GO:1902099	regulation of metaphase/anaphase transition of cell cycle	51	-0.7165	-2.28202	0.000197	0.000197	0.001229
BP	GO:0007091	metaphase/anaphase transition of mitotic cell cycle	52	-0.69801	-2.23181	0.000198	0.000198	0.001229
BP	GO:0051591	response to cAMP	95	0.561522	1.937893	0.000198	0.000198	0.001229
BP	GO:0042136	neurotransmitter biosynthetic process	95	0.553831	1.911347	0.000198	0.000198	0.001229
BP	GO:0009062	fatty acid catabolic process	102	0.775026	2.701204	0.000198	0.000198	0.001229
BP	GO:0001523	retinoid metabolic process	102	0.735479	2.563369	0.000198	0.000198	0.001229
BP	GO:1990748	cellular detoxification	102	0.640703	2.233046	0.000198	0.000198	0.001229
BP	GO:1901606	alpha-amino acid catabolic process	99	0.813741	2.824305	0.000198	0.000198	0.001229
BP	GO:0010906	regulation of glucose metabolic process	99	0.620889	2.15496	0.000198	0.000198	0.001229
BP	GO:0019395	fatty acid oxidation	96	0.746885	2.580304	0.000198	0.000198	0.001229

BP	GO:0019730	antimicrobial humoral response	96	0.565295	1.952956	0.000198	0.000198	0.001229
BP	GO:0034340	response to type I interferon	96	0.554377	1.915236	0.000198	0.000198	0.001229
BP	GO:0034440	lipid oxidation	98	0.740456	2.565064	0.000198	0.000198	0.001229
BP	GO:0006637	acyl-CoA metabolic process	98	0.680386	2.35697	0.000198	0.000198	0.001229
BP	GO:0035383	thioester metabolic process	98	0.680386	2.35697	0.000198	0.000198	0.001229
BP	GO:0022904	respiratory electron transport chain	98	0.592112	2.051175	0.000198	0.000198	0.001229
BP	GO:0032368	regulation of lipid transport	98	0.579434	2.007258	0.000198	0.000198	0.001229
BP	GO:0033559	unsaturated fatty acid metabolic process	101	0.694371	2.417386	0.000198	0.000198	0.001229
BP	GO:0055092	sterol homeostasis	77	0.760047	2.535769	0.000198	0.000198	0.001229
BP	GO:0030193	regulation of blood coagulation	77	0.731552	2.440702	0.000198	0.000198	0.001229
BP	GO:0008652	cellular amino acid biosynthetic process	77	0.708507	2.363814	0.000198	0.000198	0.001229
BP	GO:0019319	hexose biosynthetic process	77	0.705471	2.353688	0.000198	0.000198	0.001229
CC	GO:0031091	platelet alpha granule	88	0.647241	2.205895	0.000198	0.000198	0.001229
MF	GO:0004867	serine-type endopeptidase inhibitor activity	88	0.621953	2.11971	0.000198	0.000198	0.001229
BP	GO:0010965	regulation of mitotic sister chromatid separation	55	-0.68627	-2.21885	0.000199	0.000199	0.001229
CC	GO:0005871	kinesin complex	53	-0.67483	-2.16182	0.000199	0.000199	0.001229
BP	GO:1901992	positive regulation of mitotic cell cycle phase transition	74	-0.58949	-2.01853	0.000199	0.000199	0.001229
CC	GO:0005876	spindle microtubule	54	-0.66453	-2.13816	0.000199	0.000199	0.001229
BP	GO:0044784	metaphase/anaphase transition of cell cycle	54	-0.70564	-2.27044	0.000199	0.000199	0.001229
BP	GO:0006641	triglyceride metabolic process	92	0.791255	2.712358	0.000199	0.000199	0.001229
BP	GO:0015918	sterol transport	92	0.642389	2.202057	0.000199	0.000199	0.001229
BP	GO:0098869	cellular oxidant detoxification	92	0.614664	2.107018	0.000199	0.000199	0.001229
BP	GO:0051187	cofactor catabolic process	63	0.680228	2.194265	0.000199	0.000199	0.001229
MF	GO:0016651	oxidoreductase activity, acting on NAD(P)H	97	0.553066	1.912786	0.000199	0.000199	0.001229
BP	GO:0015908	fatty acid transport	93	0.604501	2.076982	0.000199	0.000199	0.001229
BP	GO:0060337	type I interferon signaling pathway	93	0.558869	1.920195	0.000199	0.000199	0.001229
BP	GO:0071357	cellular response to type I interferon	93	0.558869	1.920195	0.000199	0.000199	0.001229
BP	GO:0006690	icosanoid metabolic process	103	0.702145	2.447556	0.000199	0.000199	0.001229
BP	GO:0006119	oxidative phosphorylation	103	0.535509	1.866693	0.000199	0.000199	0.001229
BP	GO:0060191	regulation of lipase activity	94	0.606456	2.086689	0.000199	0.000199	0.001229
MF	GO:0005496	steroid binding	94	0.581671	2.001409	0.000199	0.000199	0.001229
MF	GO:0004497	monooxygenase activity	91	0.808691	2.768105	0.000199	0.000199	0.001229
BP	GO:0043648	dicarboxylic acid metabolic process	91	0.682898	2.337523	0.000199	0.000199	0.001229
BP	GO:0051289	protein homotetramerization	91	0.590586	2.021543	0.000199	0.000199	0.001229
BP	GO:0042632	cholesterol homeostasis	76	0.761894	2.536185	0.000199	0.000199	0.001229
MF	GO:0050660	flavin adenine dinucleotide binding	76	0.729744	2.429166	0.000199	0.000199	0.001229
BP	GO:0055072	iron ion homeostasis	76	0.592154	1.971157	0.000199	0.000199	0.001229
CC	GO:0098948	intrinsic component of postsynaptic specialization membrane	73	-0.55408	-1.88989	0.000199	0.000199	0.001229
BP	GO:0033045	regulation of sister chromatid segregation	73	-0.6533	-2.22831	0.000199	0.000199	0.001229

CC	GO:0000784	nuclear chromosome, telomeric region	83	-0.53883	-1.88531	0.000199	0.000199	0.001229
BP	GO:0006081	cellular aldehyde metabolic process	58	0.760528	2.413691	0.000199	0.000199	0.001229
CC	GO:0005778	peroxisomal membrane	58	0.650281	2.0638	0.000199	0.000199	0.001229
CC	GO:0031903	microbody membrane	58	0.650281	2.0638	0.000199	0.000199	0.001229
BP	GO:0017001	antibiotic catabolic process	58	0.650176	2.063467	0.000199	0.000199	0.001229
CC	GO:0099060	integral component of postsynaptic specialization membrane	72	-0.56061	-1.90584	0.000199	0.000199	0.001229
BP	GO:0062014	negative regulation of small molecule metabolic process	85	0.661534	2.242115	0.000199	0.000199	0.001229
BP	GO:0019748	secondary metabolic process	61	0.720486	2.308677	0.0002	0.0002	0.001229
BP	GO:1901607	alpha-amino acid biosynthetic process	61	0.701205	2.246894	0.0002	0.0002	0.001229
BP	GO:0046686	response to cadmium ion	61	0.628458	2.013789	0.0002	0.0002	0.001229
BP	GO:1903035	negative regulation of response to wounding	86	0.662846	2.249437	0.0002	0.0002	0.001229
CC	GO:0098800	inner mitochondrial membrane protein complex	86	0.595583	2.021174	0.0002	0.0002	0.001229
CC	GO:0031093	platelet alpha granule lumen	64	0.715704	2.313037	0.0002	0.0002	0.001229
BP	GO:0006956	complement activation	66	0.879403	2.860775	0.0002	0.0002	0.001229
BP	GO:0097006	regulation of plasma lipoprotein particle levels	82	0.716817	2.413039	0.0002	0.0002	0.001229
BP	GO:0006625	protein targeting to peroxisome	67	0.778971	2.539014	0.0002	0.0002	0.001229
BP	GO:0072662	protein localization to peroxisome	67	0.778971	2.539014	0.0002	0.0002	0.001229
BP	GO:0072663	establishment of protein localization to peroxisome	67	0.778971	2.539014	0.0002	0.0002	0.001229
CC	GO:0098803	respiratory chain complex	67	0.655524	2.136647	0.0002	0.0002	0.001229
CC	GO:0000794	condensed nuclear chromosome	84	-0.54679	-1.91603	0.0002	0.0002	0.001229
BP	GO:0032465	regulation of cytokinesis	84	-0.54722	-1.91754	0.0002	0.0002	0.001229
CC	GO:0000777	condensed chromosome kinetochore	84	-0.65884	-2.30869	0.0002	0.0002	0.001229
BP	GO:0033260	nuclear DNA replication	57	-0.63439	-2.06244	0.0002	0.0002	0.001229
BP	GO:0051784	negative regulation of nuclear division	57	-0.65693	-2.13572	0.0002	0.0002	0.001229
BP	GO:0045833	negative regulation of lipid metabolic process	75	0.629938	2.090784	0.0002	0.0002	0.001229
BP	GO:0051298	centrosome duplication	60	-0.59208	-1.94434	0.0002	0.0002	0.001229
BP	GO:1905818	regulation of chromosome separation	60	-0.67313	-2.21051	0.0002	0.0002	0.001229
BP	GO:0034308	primary alcohol metabolic process	84	0.757021	2.558685	2.00E-04	2.00E-04	0.001229
BP	GO:0019217	regulation of fatty acid metabolic process	84	0.649012	2.193619	2.00E-04	2.00E-04	0.001229
BP	GO:0042446	hormone biosynthetic process	84	0.581341	1.964895	2.00E-04	2.00E-04	0.001229
BP	GO:0007031	peroxisome organization	78	0.753682	2.514846	2.00E-04	2.00E-04	0.001229
BP	GO:1900046	regulation of hemostasis	78	0.729268	2.433382	2.00E-04	2.00E-04	0.001229
BP	GO:0120178	steroid hormone biosynthetic process	57	0.670576	2.121251	2.00E-04	2.00E-04	0.001229
CC	GO:0005746	mitochondrial respirasome	71	0.663869	2.185557	0.0002	0.0002	0.001229
BP	GO:0005977	glycogen metabolic process	71	0.636611	2.09582	0.0002	0.0002	0.001229
BP	GO:0002920	regulation of humoral immune response	68	0.806345	2.635866	0.0002	0.0002	0.001229
BP	GO:0009064	glutamine family amino acid metabolic process	68	0.700568	2.290092	0.0002	0.0002	0.001229
BP	GO:0033108	mitochondrial respiratory chain complex assembly	68	0.65978	2.156758	0.0002	0.0002	0.001229
CC	GO:0000792	heterochromatin	67	-0.58008	-1.94491	0.0002	0.0002	0.001229

BP	GO:0050000	chromosome localization	67	-0.64438	-2.16048	0.0002	0.0002	0.001229
BP	GO:0034401	chromatin organization involved in regulation of transcription	82	-0.54018	-1.88487	0.0002	0.0002	0.001229
MF	GO:0003777	microtubule motor activity	82	-0.56862	-1.98414	0.0002	0.0002	0.001229
BP	GO:0061640	cytoskeleton-dependent cytokinesis	82	-0.59098	-2.06215	0.0002	0.0002	0.001229
BP	GO:0051304	chromosome separation	82	-0.64888	-2.26419	0.0002	0.0002	0.001229
BP	GO:0045132	meiotic chromosome segregation	66	-0.62407	-2.08479	0.0002	0.0002	0.001229
BP	GO:0044786	cell cycle DNA replication	66	-0.63087	-2.1075	0.0002	0.0002	0.001229
BP	GO:0051303	establishment of chromosome localization	66	-0.64901	-2.16809	0.0002	0.0002	0.001229
BP	GO:0019915	lipid storage	65	0.626012	2.028567	0.0002	0.0002	0.001229
BP	GO:0006695	cholesterol biosynthetic process	65	0.621255	2.013151	0.0002	0.0002	0.001229
BP	GO:1902653	secondary alcohol biosynthetic process	65	0.621255	2.013151	0.0002	0.0002	0.001229
BP	GO:0000281	mitotic cytokinesis	70	-0.63497	-2.1459	0.0002	0.0002	0.001229
BP	GO:1901989	positive regulation of cell cycle phase transition	86	-0.58128	-2.04266	0.0002	0.0002	0.001229
BP	GO:0097711	ciliary basal body-plasma membrane docking	86	-0.5857	-2.0582	0.0002	0.0002	0.001229
BP	GO:0046503	glycerolipid catabolic process	62	0.693794	2.22791	0.0002	0.0002	0.001229
MF	GO:0033293	monocarboxylic acid binding	62	0.672827	2.160579	0.0002	0.0002	0.001229
BP	GO:0006334	nucleosome assembly	61	-0.59245	-1.95092	0.0002	0.0002	0.001229
BP	GO:0030301	cholesterol transport	79	0.705805	2.359916	0.0002	0.0002	0.001229
CC	GO:0070469	respirasome	79	0.657821	2.199477	0.0002	0.0002	0.001229
MF	GO:0016209	antioxidant activity	79	0.625986	2.093034	0.0002	0.0002	0.001229
BP	GO:1901616	organic hydroxy compound catabolic process	72	0.670266	2.20944	0.0002	0.0002	0.001229
BP	GO:0006073	cellular glucan metabolic process	72	0.636283	2.097418	0.0002	0.0002	0.001229
BP	GO:0044042	glucan metabolic process	72	0.636283	2.097418	0.0002	0.0002	0.001229
BP	GO:0043574	peroxisomal transport	69	0.772684	2.530938	0.0002	0.0002	0.001229
BP	GO:0006635	fatty acid beta-oxidation	69	0.727377	2.382534	0.0002	0.0002	0.001229
BP	GO:0016126	sterol biosynthetic process	69	0.617637	2.02308	0.0002	0.0002	0.001229
BP	GO:0046364	monosaccharide biosynthetic process	83	0.690058	2.325833	0.000201	0.000201	0.001229
BP	GO:0015914	phospholipid transport	83	0.60719	2.046527	0.000201	0.000201	0.001229
BP	GO:0006112	energy reserve metabolic process	83	0.599109	2.019291	0.000201	0.000201	0.001229
BP	GO:0050818	regulation of coagulation	80	0.720503	2.413697	0.000201	0.000201	0.001229
BP	GO:0042773	ATP synthesis coupled electron transport	80	0.565343	1.893908	0.000201	0.000201	0.001229
BP	GO:0042775	mitochondrial ATP synthesis coupled electron transport	80	0.565343	1.893908	0.000201	0.000201	0.001229
BP	GO:0051306	mitotic sister chromatid separation	58	-0.67243	-2.19419	0.000201	0.000201	0.001229
BP	GO:0061045	negative regulation of wound healing	73	0.727425	2.403153	0.000201	0.000201	0.001229
MF	GO:0003678	DNA helicase activity	76	-0.6409	-2.20251	0.000201	0.000201	0.001229
BP	GO:0007127	meiosis I	91	-0.53003	-1.8787	0.000201	0.000201	0.001229
BP	GO:0044773	mitotic DNA damage checkpoint	89	-0.53767	-1.89838	0.000201	0.000201	0.001229
BP	GO:0060606	tube closure	89	-0.57634	-2.03492	0.000201	0.000201	0.001229
BP	GO:0000724	double-strand break repair via homologous recombination	103	-0.59054	-2.1358	0.000201	0.000201	0.001229

BP	GO:0000725	recombinational repair	103	-0.59054	-2.1358	0.000201	0.000201	0.001229
BP	GO:0001841	neural tube formation	103	-0.59123	-2.13831	0.000201	0.000201	0.001229
BP	GO:0042472	inner ear morphogenesis	94	-0.51766	-1.84527	0.000201	0.000201	0.001229
CC	GO:0005930	axoneme	94	-0.51938	-1.85142	0.000201	0.000201	0.001229
BP	GO:0060993	kidney morphogenesis	94	-0.53835	-1.91902	0.000201	0.000201	0.001229
BP	GO:0051983	regulation of chromosome segregation	94	-0.64084	-2.28439	0.000201	0.000201	0.001229
BP	GO:0048704	embryonic skeletal system morphogenesis	93	-0.5933	-2.11118	0.000201	0.000201	0.001229
CC	GO:0072686	mitotic spindle	97	-0.56479	-2.02358	0.000201	0.000201	0.001229
MF	GO:0016627	oxidoreductase activity, acting on the CH-CH group of donors	56	0.768342	2.422479	0.000201	0.000201	0.001229
MF	GO:0051287	NAD binding	56	0.657993	2.074562	0.000201	0.000201	0.001229
BP	GO:0033013	tetrapyrrole metabolic process	56	0.626046	1.973836	0.000201	0.000201	0.001229
BP	GO:0006767	water-soluble vitamin metabolic process	81	0.602243	2.01999	0.000201	0.000201	0.001229
BP	GO:0033047	regulation of mitotic sister chromatid segregation	63	-0.65059	-2.15364	0.000201	0.000201	0.001229
BP	GO:0050819	negative regulation of coagulation	54	0.776884	2.435566	0.000201	0.000201	0.001229
MF	GO:0016597	amino acid binding	54	0.700279	2.195405	0.000201	0.000201	0.001229
BP	GO:0006094	gluconeogenesis	74	0.703928	2.328343	0.000201	0.000201	0.001229
BP	GO:0046889	positive regulation of lipid biosynthetic process	74	0.658065	2.176644	0.000201	0.000201	0.001229
BP	GO:0090181	regulation of cholesterol metabolic process	55	0.666564	2.095729	0.000201	0.000201	0.001229
BP	GO:1900047	negative regulation of hemostasis	53	0.787387	2.46253	0.000201	0.000201	0.001229
BP	GO:0001843	neural tube closure	88	-0.57855	-2.03943	0.000201	0.000201	0.001229
BP	GO:0006282	regulation of DNA repair	101	-0.5084	-1.83028	0.000202	0.000202	0.001229
BP	GO:0034728	nucleosome organization	98	-0.55924	-2.00499	0.000202	0.000202	0.001229
MF	GO:0003697	single-stranded DNA binding	96	-0.50729	-1.81393	0.000202	0.000202	0.001229
BP	GO:0044774	mitotic DNA integrity checkpoint	96	-0.54193	-1.93779	0.000202	0.000202	0.001229
BP	GO:0061982	meiosis I cell cycle process	96	-0.54965	-1.96542	0.000202	0.000202	0.001229
BP	GO:0051225	spindle assembly	96	-0.55143	-1.97176	0.000202	0.000202	0.001229
BP	GO:0051963	regulation of synapse assembly	95	-0.50807	-1.81228	0.000202	0.000202	0.001229
CC	GO:0097014	ciliary plasm	95	-0.51598	-1.84049	0.000202	0.000202	0.001229
BP	GO:0014020	primary neural tube formation	95	-0.59275	-2.11432	0.000202	0.000202	0.001229
BP	GO:0007052	mitotic spindle organization	95	-0.62703	-2.23661	0.000202	0.000202	0.001229
CC	GO:0000779	condensed chromosome, centromeric region	95	-0.65329	-2.3303	0.000202	0.000202	0.001229
BP	GO:0006275	regulation of DNA replication	100	-0.48842	-1.75491	0.000202	0.000202	0.001229
BP	GO:0030330	DNA damage response, signal transduction by p53 class medi	100	-0.49022	-1.7614	0.000202	0.000202	0.001229
BP	GO:0030195	negative regulation of blood coagulation	52	0.790388	2.461068	0.000202	0.000202	0.001229
BP	GO:0006576	cellular biogenic amine metabolic process	52	0.732078	2.279507	0.000202	0.000202	0.001229
MF	GO:0016655	oxidoreductase activity, acting on NAD(P)H, quinone or similar	52	0.7249	2.257155	0.000202	0.000202	0.001229
BP	GO:0034381	plasma lipoprotein particle clearance	52	0.7169	2.232246	0.000202	0.000202	0.001229
BP	GO:0010043	response to zinc ion	52	0.686298	2.136958	0.000202	0.000202	0.001229
BP	GO:0030449	regulation of complement activation	50	0.884163	2.73675	0.000203	0.000203	0.001229

CC	GO:0005782	peroxisomal matrix	50	0.829671	2.568081	0.000203	0.000203	0.001229
CC	GO:0031907	microbody lumen	50	0.829671	2.568081	0.000203	0.000203	0.001229
BP	GO:0050994	regulation of lipid catabolic process	51	0.708547	2.199081	0.000203	0.000203	0.001229
BP	GO:0090303	positive regulation of wound healing	51	0.660826	2.050971	0.000203	0.000203	0.001229
BP	GO:0010257	NADH dehydrogenase complex assembly	49	0.713207	2.200074	0.000203	0.000203	0.001229
BP	GO:0032981	mitochondrial respiratory chain complex I assembly	49	0.713207	2.200074	0.000203	0.000203	0.001229
BP	GO:0031638	zymogen activation	49	0.68739	2.120433	0.000203	0.000203	0.001229
MF	GO:0050661	NADP binding	49	0.670953	2.069729	0.000203	0.000203	0.001229
BP	GO:0032508	DNA duplex unwinding	105	-0.57599	-2.08429	0.000203	0.000203	0.001229
CC	GO:0036064	ciliary basal body	107	-0.49101	-1.78148	0.000204	0.000204	0.001229
MF	GO:0008094	DNA-dependent ATPase activity	106	-0.60895	-2.20745	0.000204	0.000204	0.001229
BP	GO:0031497	chromatin assembly	109	-0.56584	-2.05934	0.000204	0.000204	0.001229
BP	GO:0071825	protein-lipid complex subunit organization	48	0.808487	2.481491	0.000204	0.000204	0.001229
BP	GO:0019369	arachidonic acid metabolic process	48	0.783838	2.405833	0.000204	0.000204	0.001229
BP	GO:2001251	negative regulation of chromosome organization	120	-0.57207	-2.1128	0.000204	0.000204	0.001229
BP	GO:0030326	embryonic limb morphogenesis	120	-0.59856	-2.21062	0.000204	0.000204	0.001229
BP	GO:0035113	embryonic appendage morphogenesis	120	-0.59856	-2.21062	0.000204	0.000204	0.001229
CC	GO:0000776	kinetochore	112	-0.64135	-2.34325	0.000204	0.000204	0.001229
BP	GO:0032392	DNA geometric change	113	-0.56368	-2.06158	0.000204	0.000204	0.001229
BP	GO:0006953	acute-phase response	47	0.824727	2.520185	0.000205	0.000205	0.001229
BP	GO:0032374	regulation of cholesterol transport	43	0.77301	2.321745	0.000205	0.000205	0.001229
MF	GO:0016903	oxidoreductase activity, acting on the aldehyde or oxo group o	43	0.730383	2.193712	0.000205	0.000205	0.001229
BP	GO:0031023	microtubule organizing center organization	121	-0.50765	-1.8767	0.000205	0.000205	0.001229
BP	GO:0006261	DNA-dependent DNA replication	127	-0.61014	-2.27388	0.000205	0.000205	0.001229
CC	GO:0098936	intrinsic component of postsynaptic membrane	118	-0.46078	-1.69541	0.000205	0.000205	0.001229
BP	GO:1902850	microtubule cytoskeleton organization involved in mitosis	118	-0.62355	-2.29431	0.000205	0.000205	0.001229
CC	GO:0000781	chromosome, telomeric region	116	-0.51368	-1.88509	0.000205	0.000205	0.001229
BP	GO:0002455	humoral immune response mediated by circulating immunoglo	45	0.82802	2.507226	0.000205	0.000205	0.001229
MF	GO:0030170	pyridoxal phosphate binding	45	0.810347	2.453712	0.000205	0.000205	0.001229
MF	GO:0070279	vitamin B6 binding	45	0.810347	2.453712	0.000205	0.000205	0.001229
BP	GO:0008206	bile acid metabolic process	45	0.801866	2.42803	0.000205	0.000205	0.001229
BP	GO:0042304	regulation of fatty acid biosynthetic process	45	0.726234	2.19902	0.000205	0.000205	0.001229
MF	GO:0008028	monocarboxylic acid transmembrane transporter activity	45	0.716083	2.168281	0.000205	0.000205	0.001229
CC	GO:0099055	integral component of postsynaptic membrane	115	-0.4714	-1.72806	0.000206	0.000206	0.001229
BP	GO:0042770	signal transduction in response to DNA damage	122	-0.49372	-1.8271	0.000206	0.000206	0.001229
BP	GO:0001838	embryonic epithelial tube formation	122	-0.5556	-2.05609	0.000206	0.000206	0.001229
BP	GO:0006333	chromatin assembly or disassembly	134	-0.55436	-2.08264	0.000206	0.000206	0.001229
BP	GO:0042471	ear morphogenesis	114	-0.5021	-1.83736	0.000206	0.000206	0.001229
BP	GO:0007098	centrosome cycle	114	-0.50801	-1.85897	0.000206	0.000206	0.001229

BP	GO:0048706	embryonic skeletal system development	124	-0.52615	-1.95219	0.000206	0.000206	0.001229
BP	GO:0009066	aspartate family amino acid metabolic process	46	0.77751	2.365166	0.000206	0.000206	0.001229
MF	GO:0016830	carbon-carbon lyase activity	46	0.716848	2.180633	0.000206	0.000206	0.001229
BP	GO:0006120	mitochondrial electron transport, NADH to ubiquinone	46	0.688286	2.093749	0.000206	0.000206	0.001229
BP	GO:0055090	acylglycerol homeostasis	34	0.856065	2.453351	0.000206	0.000206	0.001229
BP	GO:0070328	triglyceride homeostasis	34	0.856065	2.453351	0.000206	0.000206	0.001229
BP	GO:0006699	bile acid biosynthetic process	34	0.829389	2.376901	0.000206	0.000206	0.001229
BP	GO:0045923	positive regulation of fatty acid metabolic process	34	0.741068	2.123788	0.000206	0.000206	0.001229
BP	GO:0071827	plasma lipoprotein particle organization	44	0.818162	2.464513	0.000206	0.000206	0.001229
BP	GO:0032371	regulation of sterol transport	44	0.766185	2.307944	0.000206	0.000206	0.001229
BP	GO:0046461	neutral lipid catabolic process	42	0.801494	2.393407	0.000206	0.000206	0.001229
BP	GO:0046464	acylglycerol catabolic process	42	0.801494	2.393407	0.000206	0.000206	0.001229
BP	GO:0000070	mitotic sister chromatid segregation	138	-0.65801	-2.48317	0.000206	0.000206	0.001229
MF	GO:0016229	steroid dehydrogenase activity	33	0.855323	2.434822	0.000206	0.000206	0.001229
BP	GO:0046460	neutral lipid biosynthetic process	33	0.843792	2.401997	0.000206	0.000206	0.001229
BP	GO:0046463	acylglycerol biosynthetic process	33	0.843792	2.401997	0.000206	0.000206	0.001229
BP	GO:0006536	glutamate metabolic process	33	0.765426	2.178916	0.000206	0.000206	0.001229
BP	GO:0072524	pyridine-containing compound metabolic process	33	0.76329	2.172835	0.000206	0.000206	0.001229
BP	GO:0051028	mRNA transport	132	-0.46916	-1.75775	0.000206	0.000206	0.001229
BP	GO:0072175	epithelial tube formation	130	-0.54718	-2.04597	0.000206	0.000206	0.001229
BP	GO:0001738	morphogenesis of a polarized epithelium	137	-0.4729	-1.783	0.000206	0.000206	0.001229
BP	GO:0032200	telomere organization	137	-0.50627	-1.90881	0.000206	0.000206	0.001229
BP	GO:0000723	telomere maintenance	136	-0.50519	-1.90112	0.000206	0.000206	0.001229
BP	GO:0000077	DNA damage checkpoint	129	-0.50256	-1.87603	0.000206	0.000206	0.001229
CC	GO:0034358	plasma lipoprotein particle	35	0.900927	2.598078	0.000207	0.000207	0.001229
CC	GO:1990777	lipoprotein particle	35	0.900927	2.598078	0.000207	0.000207	0.001229
BP	GO:0019433	triglyceride catabolic process	35	0.821148	2.368013	0.000207	0.000207	0.001229
MF	GO:0019825	oxygen binding	35	0.799058	2.304312	0.000207	0.000207	0.001229
CC	GO:0032994	protein-lipid complex	37	0.895091	2.60309	0.000207	0.000207	0.001229
CC	GO:0000922	spindle pole	140	-0.51697	-1.95432	0.000207	0.000207	0.001229
BP	GO:0000096	sulfur amino acid metabolic process	39	0.7475	2.19951	0.000207	0.000207	0.001229
MF	GO:0016877	ligase activity, forming carbon-sulfur bonds	39	0.719808	2.118028	0.000207	0.000207	0.001229
BP	GO:0140013	meiotic nuclear division	142	-0.55905	-2.1162	0.000207	0.000207	0.001229
BP	GO:0035107	appendage morphogenesis	142	-0.60931	-2.30644	0.000207	0.000207	0.001229
BP	GO:0035108	limb morphogenesis	142	-0.60931	-2.30644	0.000207	0.000207	0.001229
BP	GO:0034367	protein-containing complex remodeling	29	0.867876	2.395019	0.000207	0.000207	0.001229
MF	GO:0015020	glucuronosyltransferase activity	29	0.785224	2.166929	0.000207	0.000207	0.001229
BP	GO:0072525	pyridine-containing compound biosynthetic process	29	0.780169	2.15298	0.000207	0.000207	0.001229
MF	GO:0016878	acid-thiol ligase activity	29	0.769638	2.123916	0.000207	0.000207	0.001229

BP	GO:0006958	complement activation, classical pathway	32	0.921615	2.600816	0.000207	0.000207	0.001229
BP	GO:0090207	regulation of triglyceride metabolic process	32	0.826658	2.332846	0.000207	0.000207	0.001229
BP	GO:0042573	retinoic acid metabolic process	32	0.810652	2.287675	0.000207	0.000207	0.001229
BP	GO:0008210	estrogen metabolic process	32	0.810117	2.286166	0.000207	0.000207	0.001229
BP	GO:0019835	cytolysis	40	0.836074	2.472421	0.000207	0.000207	0.001229
BP	GO:0042572	retinol metabolic process	40	0.82262	2.432638	0.000207	0.000207	0.001229
BP	GO:0033344	cholesterol efflux	40	0.772485	2.284379	0.000207	0.000207	0.001229
BP	GO:1905953	negative regulation of lipid localization	40	0.715417	2.115618	0.000207	0.000207	0.001229
MF	GO:0008395	steroid hydroxylase activity	36	0.894185	2.584672	0.000208	0.000208	0.001229
MF	GO:0016709	oxidoreductase activity, acting on paired donors, with incorpor	36	0.81671	2.360728	0.000208	0.000208	0.001229
MF	GO:0120013	lipid transfer activity	36	0.785605	2.270818	0.000208	0.000208	0.001229
BP	GO:0006778	porphyrin-containing compound metabolic process	36	0.742502	2.146226	0.000208	0.000208	0.001229
BP	GO:0031570	DNA integrity checkpoint	139	-0.51975	-1.96138	0.000208	0.000208	0.001229
BP	GO:0009072	aromatic amino acid family metabolic process	38	0.823929	2.409886	0.000208	0.000208	0.001229
BP	GO:0009069	serine family amino acid metabolic process	38	0.806142	2.357862	0.000208	0.000208	0.001229
BP	GO:0065005	protein-lipid complex assembly	31	0.854474	2.38866	0.000208	0.000208	0.001229
BP	GO:0019432	triglyceride biosynthetic process	31	0.845454	2.363445	0.000208	0.000208	0.001229
MF	GO:0071949	FAD binding	31	0.756285	2.114176	0.000208	0.000208	0.001229
BP	GO:0045931	positive regulation of mitotic cell cycle	143	-0.47667	-1.80471	0.000208	0.000208	0.001229
BP	GO:0035148	tube formation	145	-0.50182	-1.90381	0.000208	0.000208	0.001229
CC	GO:0005875	microtubule associated complex	147	-0.49985	-1.8993	0.000209	0.000209	0.001229
BP	GO:0016331	morphogenesis of embryonic epithelium	147	-0.51669	-1.96329	0.000209	0.000209	0.001229
BP	GO:0140056	organelle localization by membrane tethering	157	-0.47735	-1.83038	0.000209	0.000209	0.001229
BP	GO:0065004	protein-DNA complex assembly	157	-0.47965	-1.83921	0.000209	0.000209	0.001229
BP	GO:0021915	neural tube development	157	-0.50949	-1.95361	0.000209	0.000209	0.001229
BP	GO:1903046	meiotic cell cycle process	157	-0.55466	-2.12684	0.000209	0.000209	0.001229
BP	GO:0022406	membrane docking	165	-0.46927	-1.80991	0.000209	0.000209	0.001229
CC	GO:0000775	chromosome, centromeric region	165	-0.61578	-2.37499	0.000209	0.000209	0.001229
MF	GO:0016712	oxidoreductase activity, acting on paired donors, with incorpor	30	0.860338	2.384978	0.000209	0.000209	0.001229
BP	GO:1901661	quinone metabolic process	30	0.755609	2.094654	0.000209	0.000209	0.001229
BP	GO:0042759	long-chain fatty acid biosynthetic process	30	0.753228	2.088055	0.000209	0.000209	0.001229
BP	GO:0034368	protein-lipid complex remodeling	28	0.874994	2.39458	0.000209	0.000209	0.001229
BP	GO:0034369	plasma lipoprotein particle remodeling	28	0.874994	2.39458	0.000209	0.000209	0.001229
BP	GO:0042730	fibrinolysis	28	0.861164	2.356729	0.000209	0.000209	0.001229
MF	GO:0033764	steroid dehydrogenase activity, acting on the CH-OH group of	28	0.836941	2.29044	0.000209	0.000209	0.001229
BP	GO:0008209	androgen metabolic process	28	0.824249	2.255706	0.000209	0.000209	0.001229
BP	GO:0000910	cytokinesis	148	-0.50859	-1.93324	0.000209	0.000209	0.001229
BP	GO:0034377	plasma lipoprotein particle assembly	27	0.868856	2.357819	0.000209	0.000209	0.001229
BP	GO:0045940	positive regulation of steroid metabolic process	27	0.800087	2.171198	0.000209	0.000209	0.001229

BP	GO:1901796	regulation of signal transduction by p53 class mediator	168	-0.4717	-1.82222	0.000209	0.000209	0.001229
BP	GO:0000819	sister chromatid segregation	168	-0.60672	-2.34378	0.000209	0.000209	0.001229
CC	GO:0030496	midbody	154	-0.48553	-1.85432	0.00021	0.00021	0.001229
BP	GO:0007088	regulation of mitotic nuclear division	154	-0.55083	-2.1037	0.00021	0.00021	0.001229
BP	GO:0070507	regulation of microtubule cytoskeleton organization	169	-0.46609	-1.80163	0.00021	0.00021	0.001229
CC	GO:0034364	high-density lipoprotein particle	26	0.939045	2.52878	0.00021	0.00021	0.001229
BP	GO:0009074	aromatic amino acid family catabolic process	26	0.880717	2.371705	0.00021	0.00021	0.001229
BP	GO:0072376	protein activation cascade	26	0.869971	2.342767	0.00021	0.00021	0.001229
BP	GO:0072378	blood coagulation, fibrin clot formation	26	0.869971	2.342767	0.00021	0.00021	0.001229
CC	GO:0032838	plasma membrane bounded cell projection cytoplasm	175	-0.43471	-1.68949	0.00021	0.00021	0.001229
BP	GO:0051236	establishment of RNA localization	174	-0.42333	-1.64303	0.00021	0.00021	0.001229
BP	GO:0006338	chromatin remodeling	174	-0.4597	-1.78417	0.00021	0.00021	0.001229
MF	GO:0042393	histone binding	174	-0.47879	-1.85828	0.00021	0.00021	0.001229
BP	GO:0007051	spindle organization	150	-0.54947	-2.09085	0.00021	0.00021	0.001229
BP	GO:0007093	mitotic cell cycle checkpoint	150	-0.56338	-2.1438	0.00021	0.00021	0.001229
BP	GO:0048736	appendage development	171	-0.5989	-2.31791	0.00021	0.00021	0.001229
BP	GO:0060173	limb development	171	-0.5989	-2.31791	0.00021	0.00021	0.001229
BP	GO:0010469	regulation of signaling receptor activity	151	-0.43841	-1.67005	0.00021	0.00021	0.001229
BP	GO:0006323	DNA packaging	151	-0.56104	-2.1372	0.00021	0.00021	0.001229
BP	GO:0048839	inner ear development	183	-0.4355	-1.70241	0.00021	0.00021	0.001229
BP	GO:0010389	regulation of G2/M transition of mitotic cell cycle	180	-0.52842	-2.06167	0.000211	0.000211	0.001229
BP	GO:0051783	regulation of nuclear division	178	-0.53384	-2.07878	0.000211	0.000211	0.001229
MF	GO:0004386	helicase activity	153	-0.47418	-1.80881	0.000211	0.000211	0.001229
BP	GO:0050770	regulation of axonogenesis	177	-0.4326	-1.68244	0.000211	0.000211	0.001229
BP	GO:2001020	regulation of response to DNA damage stimulus	184	-0.45176	-1.7672	0.000211	0.000211	0.001229
CC	GO:0000793	condensed chromosome	188	-0.56113	-2.20048	0.000212	0.000212	0.001229
BP	GO:0006302	double-strand break repair	189	-0.54367	-2.13366	0.000212	0.000212	0.001229
BP	GO:0015721	bile acid and bile salt transport	24	0.856713	2.257971	0.000213	0.000213	0.001229
BP	GO:0045922	negative regulation of fatty acid metabolic process	24	0.814157	2.145809	0.000213	0.000213	0.001229
BP	GO:0009435	NAD biosynthetic process	24	0.790907	2.084531	0.000213	0.000213	0.001229
BP	GO:0006403	RNA localization	208	-0.41801	-1.66113	0.000213	0.000213	0.001229
BP	GO:1901991	negative regulation of mitotic cell cycle phase transition	209	-0.4476	-1.78002	0.000213	0.000213	0.001229
BP	GO:0042537	benzene-containing compound metabolic process	23	0.857812	2.241356	0.000213	0.000213	0.001229
BP	GO:0006063	uronic acid metabolic process	23	0.857241	2.239863	0.000213	0.000213	0.001229
BP	GO:0019585	glucuronate metabolic process	23	0.857241	2.239863	0.000213	0.000213	0.001229
BP	GO:0042402	cellular biogenic amine catabolic process	20	0.872888	2.222522	0.000213	0.000213	0.001229
MF	GO:0004745	retinol dehydrogenase activity	20	0.85086	2.166434	0.000213	0.000213	0.001229
BP	GO:0051004	regulation of lipoprotein lipase activity	20	0.847395	2.157612	0.000213	0.000213	0.001229
CC	GO:0071682	endocytic vesicle lumen	20	0.844979	2.151462	0.000213	0.000213	0.001229

BP	GO:0009068	aspartate family amino acid catabolic process	20	0.842986	2.146388	0.000213	0.000213	0.001229
MF	GO:0015645	fatty acid ligase activity	20	0.839155	2.136633	0.000213	0.000213	0.001229
MF	GO:0001848	complement binding	20	0.836137	2.128949	0.000213	0.000213	0.001229
BP	GO:0009952	anterior/posterior pattern specification	213	-0.44412	-1.77003	0.000213	0.000213	0.001229
CC	GO:0099568	cytoplasmic region	217	-0.40337	-1.61168	0.000214	0.000214	0.001229
BP	GO:0050803	regulation of synapse structure or activity	207	-0.41225	-1.63723	0.000214	0.000214	0.001229
MF	GO:0140097	catalytic activity, acting on DNA	192	-0.48953	-1.92346	0.000214	0.000214	0.001229
BP	GO:0045665	negative regulation of neuron differentiation	211	-0.39696	-1.57996	0.000214	0.000214	0.001229
BP	GO:0098813	nuclear chromosome segregation	219	-0.5874	-2.35083	0.000214	0.000214	0.001229
BP	GO:0051321	meiotic cell cycle	215	-0.50641	-2.0205	0.000214	0.000214	0.001229
BP	GO:0042738	exogenous drug catabolic process	22	0.918053	2.380028	0.000214	0.000214	0.001229
BP	GO:0031639	plasminogen activation	22	0.829457	2.150344	0.000214	0.000214	0.001229
CC	GO:0046930	pore complex	22	0.817067	2.118222	0.000214	0.000214	0.001229
BP	GO:0051216	cartilage development	197	-0.41777	-1.64694	0.000214	0.000214	0.001229
BP	GO:0007163	establishment or maintenance of cell polarity	197	-0.41928	-1.6529	0.000214	0.000214	0.001229
BP	GO:0050807	regulation of synapse organization	198	-0.42102	-1.66159	0.000214	0.000214	0.001229
BP	GO:0009310	amine catabolic process	21	0.868354	2.228376	0.000214	0.000214	0.001229
BP	GO:0009404	toxin metabolic process	21	0.82705	2.122384	0.000214	0.000214	0.001229
BP	GO:0043583	ear development	210	-0.43108	-1.71441	0.000214	0.000214	0.001229
BP	GO:0060560	developmental growth involved in morphogenesis	228	-0.41525	-1.67011	0.000214	0.000214	0.001229
BP	GO:0000086	G2/M transition of mitotic cell cycle	227	-0.5167	-2.07725	0.000214	0.000214	0.001229
BP	GO:1901988	negative regulation of cell cycle phase transition	221	-0.43706	-1.74999	0.000214	0.000214	0.001229
MF	GO:0008017	microtubule binding	223	-0.42279	-1.69464	0.000214	0.000214	0.001229
BP	GO:0071824	protein-DNA complex subunit organization	195	-0.4661	-1.8339	0.000214	0.000214	0.001229
BP	GO:0000082	G1/S transition of mitotic cell cycle	226	-0.43692	-1.7548	0.000215	0.000215	0.001229
BP	GO:0140014	mitotic nuclear division	241	-0.58243	-2.35942	0.000215	0.000215	0.001229
BP	GO:1902749	regulation of cell cycle G2/M phase transition	193	-0.51464	-2.02213	0.000215	0.000215	0.001229
BP	GO:0044843	cell cycle G1/S phase transition	239	-0.43901	-1.77667	0.000215	0.000215	0.001229
BP	GO:0006260	DNA replication	239	-0.54042	-2.18708	0.000215	0.000215	0.001229
BP	GO:0040029	regulation of gene expression, epigenetic	247	-0.38735	-1.57359	0.000215	0.000215	0.001229
BP	GO:0044839	cell cycle G2/M phase transition	242	-0.50329	-2.03922	0.000215	0.000215	0.001229
BP	GO:0032886	regulation of microtubule-based process	194	-0.46054	-1.80976	0.000215	0.000215	0.001229
BP	GO:0000075	cell cycle checkpoint	194	-0.53215	-2.09118	0.000215	0.000215	0.001229
BP	GO:0048705	skeletal system morphogenesis	232	-0.43855	-1.7677	0.000215	0.000215	0.001229
BP	GO:0006310	DNA recombination	232	-0.50098	-2.01934	0.000215	0.000215	0.001229
BP	GO:0072331	signal transduction by p53 class mediator	248	-0.42348	-1.72056	0.000215	0.000215	0.001229
BP	GO:0090068	positive regulation of cell cycle process	265	-0.48072	-1.96917	0.000216	0.000216	0.001229
BP	GO:0090596	sensory organ morphogenesis	249	-0.43383	-1.76269	0.000216	0.000216	0.001229
BP	GO:0010976	positive regulation of neuron projection development	259	-0.36985	-1.51015	0.000216	0.000216	0.001229

BP	GO:0031503	protein-containing complex localization	255	-0.42307	-1.72323	0.000217	0.000217	0.001229
BP	GO:0071103	DNA conformation change	262	-0.53372	-2.18179	0.000217	0.000217	0.001229
BP	GO:0007018	microtubule-based movement	252	-0.46001	-1.8704	0.000217	0.000217	0.001229
BP	GO:0060485	mesenchyme development	261	-0.4083	-1.66758	0.000217	0.000217	0.001229
BP	GO:0097485	neuron projection guidance	270	-0.38267	-1.56917	0.000218	0.000218	0.001229
BP	GO:0007059	chromosome segregation	271	-0.57737	-2.36881	0.000218	0.000218	0.001229
BP	GO:0007411	axon guidance	269	-0.38	-1.55693	0.000218	0.000218	0.001229
BP	GO:0050768	negative regulation of neurogenesis	272	-0.36822	-1.51116	0.000219	0.000219	0.001229
BP	GO:0048562	embryonic organ morphogenesis	282	-0.46493	-1.91749	0.000219	0.000219	0.001229
CC	GO:0034702	ion channel complex	287	-0.37808	-1.56156	0.000219	0.000219	0.001229
CC	GO:0098687	chromosomal region	279	-0.55663	-2.29116	0.000219	0.000219	0.001229
BP	GO:0060271	cilium assembly	301	-0.4443	-1.84361	0.00022	0.00022	0.001229
CC	GO:0005819	spindle	301	-0.49559	-2.05644	0.00022	0.00022	0.001229
BP	GO:0051961	negative regulation of nervous system development	293	-0.36426	-1.50669	0.000221	0.000221	0.001229
BP	GO:0051052	regulation of DNA metabolic process	293	-0.36667	-1.51665	0.000221	0.000221	0.001229
BP	GO:0045930	negative regulation of mitotic cell cycle	290	-0.44606	-1.84221	0.000221	0.000221	0.001229
BP	GO:0060562	epithelial tube morphogenesis	318	-0.43389	-1.81087	0.000221	0.000221	0.001229
BP	GO:0000377	RNA splicing, via transesterification reactions with bulged ader	302	-0.37851	-1.57084	0.000221	0.000221	0.001229
BP	GO:0000398	mRNA splicing, via spliceosome	302	-0.37851	-1.57084	0.000221	0.000221	0.001229
BP	GO:0044782	cilium organization	315	-0.43992	-1.83549	0.000221	0.000221	0.001229
BP	GO:0033044	regulation of chromosome organization	299	-0.44471	-1.84392	0.000221	0.000221	0.001229
CC	GO:0000790	nuclear chromatin	292	-0.36641	-1.5141	0.000221	0.000221	0.001229
MF	GO:0015631	tubulin binding	303	-0.39848	-1.65443	0.000221	0.000221	0.001229
BP	GO:0060070	canonical Wnt signaling pathway	310	-0.37291	-1.55232	0.000221	0.000221	0.001229
BP	GO:0000375	RNA splicing, via transesterification reactions	305	-0.37914	-1.57514	0.000221	0.000221	0.001229
BP	GO:0010721	negative regulation of cell development	311	-0.36557	-1.52201	0.000221	0.000221	0.001229
BP	GO:0010948	negative regulation of cell cycle process	311	-0.44402	-1.84867	0.000221	0.000221	0.001229
CC	GO:0032279	asymmetric synapse	308	-0.40836	-1.69859	0.000221	0.000221	0.001229
BP	GO:0030111	regulation of Wnt signaling pathway	333	-0.3728	-1.56404	0.000222	0.000222	0.001229
MF	GO:0022836	gated channel activity	331	-0.37732	-1.58106	0.000222	0.000222	0.001229
MF	GO:0045296	cadherin binding	304	-0.35881	-1.48918	0.000222	0.000222	0.001229
CC	GO:0014069	postsynaptic density	304	-0.41075	-1.70476	0.000222	0.000222	0.001229
BP	GO:0031346	positive regulation of cell projection organization	346	-0.36107	-1.51961	0.000222	0.000222	0.001229
CC	GO:0098984	neuron to neuron synapse	328	-0.38543	-1.61268	0.000222	0.000222	0.001229
BP	GO:0001654	eye development	350	-0.35417	-1.49275	0.000222	0.000222	0.001229
BP	GO:0003002	regionalization	342	-0.42983	-1.80624	0.000223	0.000223	0.001229
BP	GO:0045666	positive regulation of neuron differentiation	343	-0.39575	-1.66325	0.000223	0.000223	0.001229
CC	GO:0098978	glutamatergic synapse	337	-0.38642	-1.62199	0.000223	0.000223	0.001229
CC	GO:0099572	postsynaptic specialization	327	-0.40394	-1.68946	0.000223	0.000223	0.001229

BP	GO:0051656	establishment of organelle localization	367	-0.36275	-1.53629	0.000223	0.000223	0.001229
BP	GO:0045787	positive regulation of cell cycle	349	-0.43237	-1.82146	0.000223	0.000223	0.001229
BP	GO:0000280	nuclear division	356	-0.54613	-2.30613	0.000223	0.000223	0.001229
BP	GO:0010639	negative regulation of organelle organization	338	-0.3632	-1.52402	0.000223	0.000223	0.001229
BP	GO:0048880	sensory system development	359	-0.34846	-1.47161	0.000224	0.000224	0.001229
BP	GO:0022613	ribonucleoprotein complex biogenesis	375	-0.36546	-1.55148	0.000224	0.000224	0.001229
BP	GO:0008380	RNA splicing	378	-0.36722	-1.55976	0.000225	0.000225	0.001229
BP	GO:0043588	skin development	398	-0.37802	-1.61387	0.000226	0.000226	0.001229
BP	GO:1901987	regulation of cell cycle phase transition	408	-0.46037	-1.96981	0.000226	0.000226	0.001229
CC	GO:0005874	microtubule	377	-0.38683	-1.64188	0.000226	0.000226	0.001229
BP	GO:0048285	organelle fission	389	-0.49337	-2.1021	0.000226	0.000226	0.001229
MF	GO:0016887	ATPase activity	393	-0.34491	-1.47061	0.000226	0.000226	0.001229
BP	GO:1901990	regulation of mitotic cell cycle phase transition	383	-0.46394	-1.97282	0.000226	0.000226	0.001229
BP	GO:0050808	synapse organization	384	-0.40059	-1.70407	0.000226	0.000226	0.001229
MF	GO:0005216	ion channel activity	404	-0.36058	-1.54094	0.000226	0.000226	0.001229
BP	GO:0034329	cell junction assembly	387	-0.3559	-1.51501	0.000226	0.000226	0.001229
BP	GO:0016569	covalent chromatin modification	421	-0.39409	-1.69109	0.000227	0.000227	0.001229
MF	GO:0004674	protein serine/threonine kinase activity	420	-0.37817	-1.62217	0.000227	0.000227	0.001229
CC	GO:0097060	synaptic membrane	412	-0.34813	-1.49045	0.000227	0.000227	0.001229
BP	GO:0016570	histone modification	406	-0.39024	-1.66855	0.000227	0.000227	0.001229
BP	GO:0048568	embryonic organ development	417	-0.44622	-1.91232	0.000227	0.000227	0.001229
CC	GO:0005667	transcription regulator complex	403	-0.35919	-1.53383	0.000227	0.000227	0.001229
CC	GO:0000785	chromatin	435	-0.39053	-1.68101	0.000228	0.000228	0.001229
BP	GO:0007389	pattern specification process	433	-0.43557	-1.87339	0.000228	0.000228	0.001229
BP	GO:0006397	mRNA processing	438	-0.3635	-1.56466	0.000229	0.000229	0.001229
BP	GO:0008544	epidermis development	438	-0.37103	-1.59708	0.000229	0.000229	0.001229
BP	GO:0050769	positive regulation of neurogenesis	438	-0.39059	-1.68128	0.000229	0.000229	0.001229
CC	GO:0005813	centrosome	444	-0.45425	-1.95733	0.00023	0.00023	0.001229
BP	GO:0007409	axonogenesis	455	-0.36777	-1.58808	0.00023	0.00023	0.001229
BP	GO:0006281	DNA repair	461	-0.4269	-1.84527	0.000231	0.000231	0.001229
BP	GO:0010975	regulation of neuron projection development	460	-0.39399	-1.70252	0.000231	0.000231	0.001229
BP	GO:0120031	plasma membrane bounded cell projection assembly	467	-0.39447	-1.70676	0.000231	0.000231	0.001229
BP	GO:0000226	microtubule cytoskeleton organization	477	-0.4928	-2.13621	0.000232	0.000232	0.001229
BP	GO:0051493	regulation of cytoskeleton organization	482	-0.32492	-1.40931	0.000232	0.000232	0.001229
BP	GO:0030031	cell projection assembly	479	-0.39266	-1.70209	0.000232	0.000232	0.001229
BP	GO:0016055	Wnt signaling pathway	483	-0.36563	-1.58584	0.000233	0.000233	0.001229
MF	GO:0003682	chromatin binding	491	-0.41052	-1.78232	0.000233	0.000233	0.001229
BP	GO:0198738	cell-cell signaling by wnt	485	-0.36617	-1.58803	0.000233	0.000233	0.001229
CC	GO:0000228	nuclear chromosome	485	-0.43827	-1.90069	0.000233	0.000233	0.001229

BP	GO:0061564	axon development	493	-0.36322	-1.57699	0.000233	0.000233	0.001229
BP	GO:0001501	skeletal system development	495	-0.35424	-1.53854	0.000234	0.000234	0.001229
BP	GO:0051962	positive regulation of nervous system development	495	-0.39045	-1.69579	0.000234	0.000234	0.001229
BP	GO:0002009	morphogenesis of an epithelium	523	-0.41961	-1.83214	0.000235	0.000235	0.001229
BP	GO:0044772	mitotic cell cycle phase transition	515	-0.48307	-2.10734	0.000235	0.000235	0.001229
MF	GO:0000978	RNA polymerase II cis-regulatory region sequence-specific DN	524	-0.32643	-1.42584	0.000235	0.000235	0.001229
CC	GO:0005929	cilium	500	-0.36025	-1.56587	0.000235	0.000235	0.001229
BP	GO:0007423	sensory organ development	528	-0.3811	-1.66566	0.000236	0.000236	0.001229
BP	GO:0010720	positive regulation of cell development	504	-0.33449	-1.45543	0.000236	0.000236	0.001229
BP	GO:0044770	cell cycle phase transition	547	-0.47371	-2.07617	0.000236	0.000236	0.001229
BP	GO:0007507	heart development	531	-0.33219	-1.45189	0.000237	0.000237	0.001229
MF	GO:0000987	cis-regulatory region sequence-specific DNA binding	541	-0.33553	-1.46947	0.000237	0.000237	0.001229
BP	GO:0051301	cell division	532	-0.51644	-2.25775	0.000237	0.000237	0.001229
MF	GO:0004672	protein kinase activity	568	-0.3817	-1.67842	0.000237	0.000237	0.001229
BP	GO:0048667	cell morphogenesis involved in neuron differentiation	560	-0.34955	-1.53516	0.000237	0.000237	0.001229
BP	GO:0048598	embryonic morphogenesis	560	-0.41996	-1.84441	0.000237	0.000237	0.001229
BP	GO:0045786	negative regulation of cell cycle	561	-0.38077	-1.67227	0.000238	0.000238	0.001229
BP	GO:0051640	organelle localization	561	-0.3917	-1.72025	0.000238	0.000238	0.001229
BP	GO:0007346	regulation of mitotic cell cycle	577	-0.4352	-1.91608	0.000238	0.000238	0.001229
BP	GO:1905114	cell surface receptor signaling pathway involved in cell-cell sig	583	-0.345	-1.5199	0.000238	0.000238	0.001229
CC	GO:0098794	postsynapse	583	-0.35034	-1.54343	0.000238	0.000238	0.001229
BP	GO:0043009	chordate embryonic development	603	-0.4066	-1.79567	0.00024	0.00024	0.001232
BP	GO:0045664	regulation of neuron differentiation	605	-0.39613	-1.7501	0.00024	0.00024	0.001232
BP	GO:0120035	regulation of plasma membrane bounded cell projection orgar	607	-0.3858	-1.70478	0.00024	0.00024	0.001232
BP	GO:0120039	plasma membrane bounded cell projection morphogenesis	636	-0.3761	-1.66795	0.000241	0.000241	0.001232
BP	GO:0006325	chromatin organization	637	-0.40347	-1.78959	0.000241	0.000241	0.001232
BP	GO:0034330	cell junction organization	626	-0.3619	-1.60283	0.000242	0.000242	0.001232
BP	GO:0009792	embryo development ending in birth or egg hatching	620	-0.40154	-1.77686	0.000242	0.000242	0.001232
BP	GO:0048858	cell projection morphogenesis	640	-0.3775	-1.67386	0.000242	0.000242	0.001232
CC	GO:0005815	microtubule organizing center	628	-0.42132	-1.86619	0.000242	0.000242	0.001232
BP	GO:0048812	neuron projection morphogenesis	623	-0.37543	-1.6615	0.000243	0.000243	0.001232
BP	GO:0031344	regulation of cell projection organization	615	-0.38299	-1.69311	0.000243	0.000243	0.001232
BP	GO:0048729	tissue morphogenesis	646	-0.39835	-1.7669	0.000243	0.000243	0.001233
MF	GO:0016773	phosphotransferase activity, alcohol group as acceptor	662	-0.31863	-1.41564	0.000244	0.000244	0.001233
BP	GO:0032990	cell part morphogenesis	656	-0.36736	-1.63077	0.000244	0.000244	0.001233
BP	GO:0007017	microtubule-based process	660	-0.46106	-2.04792	0.000244	0.000244	0.001233
CC	GO:0099513	polymeric cytoskeletal fiber	667	-0.34185	-1.5194	0.000245	0.000245	0.001236
BP	GO:0007268	chemical synaptic transmission	679	-0.3199	-1.42385	0.000246	0.000246	0.001239
BP	GO:0098916	anterograde trans-synaptic signaling	679	-0.3199	-1.42385	0.000246	0.000246	0.001239

BP	GO:0007420	brain development	690	-0.3389	-1.51	0.000247	0.000247	0.001242
BP	GO:0010564	regulation of cell cycle process	692	-0.4472	-1.99232	0.000248	0.000248	0.001242
BP	GO:0099537	trans-synaptic signaling	687	-0.3214	-1.43112	0.000248	0.000248	0.001243
CC	GO:0016604	nuclear body	693	-0.31693	-1.41173	0.000248	0.000248	0.001243
BP	GO:0071407	cellular response to organic cyclic compound	572	0.332209	1.40656	0.000346	0.000346	0.001731
BP	GO:0009896	positive regulation of catabolic process	402	0.361201	1.485988	0.000358	0.000358	0.001787
MF	GO:0004175	endopeptidase activity	377	0.375604	1.535716	0.000359	0.000359	0.001789
CC	GO:0009897	external side of plasma membrane	303	0.40838	1.635257	0.000365	0.000365	0.00181
BP	GO:0032496	response to lipopolysaccharide	307	0.416817	1.671357	0.000365	0.000365	0.00181
BP	GO:0001101	response to acid chemical	321	0.40339	1.624972	0.000365	0.000365	0.00181
CC	GO:0016324	apical plasma membrane	299	0.411587	1.646171	0.000365	0.000365	0.00181
BP	GO:0002237	response to molecule of bacterial origin	318	0.405773	1.632613	0.000366	0.000366	0.00181
MF	GO:0015291	secondary active transmembrane transporter activity	224	0.431354	1.676241	0.000375	0.000375	0.001846
BP	GO:0032869	cellular response to insulin stimulus	201	0.430528	1.651639	0.000375	0.000375	0.001846
BP	GO:0016485	protein processing	202	0.441855	1.695699	0.000375	0.000375	0.001846
BP	GO:0033500	carbohydrate homeostasis	217	0.43532	1.68446	0.000376	0.000376	0.001846
BP	GO:0042593	glucose homeostasis	217	0.43532	1.68446	0.000376	0.000376	0.001846
MF	GO:0017116	single-stranded DNA helicase activity	20	-0.75907	-1.96493	0.000376	0.000376	0.001846
BP	GO:0009743	response to carbohydrate	212	0.418708	1.61537	0.000376	0.000376	0.001846
BP	GO:0008217	regulation of blood pressure	173	0.457133	1.722489	0.000381	0.000381	0.001862
BP	GO:0006109	regulation of carbohydrate metabolic process	181	0.463278	1.752847	0.000382	0.000382	0.001862
BP	GO:0042594	response to starvation	175	0.466213	1.758663	0.000382	0.000382	0.001862
BP	GO:0007143	female meiotic nuclear division	26	-0.70566	-1.94175	0.000382	0.000382	0.001862
BP	GO:0003018	vascular process in circulatory system	164	0.447173	1.674585	0.000382	0.000382	0.001862
BP	GO:0001906	cell killing	159	0.479111	1.784677	0.000383	0.000383	0.001864
BP	GO:0040001	establishment of mitotic spindle localization	30	-0.66731	-1.89947	0.000384	0.000384	0.001864
BP	GO:0030261	chromosome condensation	31	-0.6899	-1.97885	0.000385	0.000385	0.001868
BP	GO:0051293	establishment of spindle localization	40	-0.6389	-1.93826	0.000386	0.000386	0.001869
CC	GO:0030176	integral component of endoplasmic reticulum membrane	131	0.467327	1.696034	0.000388	0.000388	0.001869
CC	GO:0031227	intrinsic component of endoplasmic reticulum membrane	138	0.463284	1.692819	0.000388	0.000388	0.001869
BP	GO:0008643	carbohydrate transport	134	0.471397	1.714733	0.000389	0.000389	0.001869
BP	GO:0046434	organophosphate catabolic process	128	0.475165	1.717941	0.00039	0.00039	0.001869
BP	GO:0046717	acid secretion	119	0.492406	1.759901	0.000391	0.000391	0.001869
BP	GO:1901657	glycosyl compound metabolic process	120	0.487428	1.743968	0.000392	0.000392	0.001869
CC	GO:0099061	integral component of postsynaptic density membrane	48	-0.63241	-1.9897	0.000392	0.000392	0.001869
BP	GO:0090307	mitotic spindle assembly	49	-0.66477	-2.09789	0.000394	0.000394	0.001869
BP	GO:1903036	positive regulation of response to wounding	61	0.616799	1.976429	0.000399	0.000399	0.001869
BP	GO:0006879	cellular iron ion homeostasis	59	0.612448	1.948877	0.000399	0.000399	0.001869
BP	GO:0060193	positive regulation of lipase activity	70	0.567409	1.863275	0.000399	0.000399	0.001869

BP	GO:0015909	long-chain fatty acid transport	66	0.581775	1.892565	0.0004	0.0004	0.001869
BP	GO:0001892	embryonic placenta development	85	-0.49818	-1.74943	0.000401	0.000401	0.001869
BP	GO:0042743	hydrogen peroxide metabolic process	55	0.609977	1.917815	0.000403	0.000403	0.001869
MF	GO:0016765	transferase activity, transferring alkyl or aryl (other than methy	55	0.580581	1.825393	0.000403	0.000403	0.001869
BP	GO:0046164	alcohol catabolic process	53	0.631724	1.975698	0.000403	0.000403	0.001869
BP	GO:0044273	sulfur compound catabolic process	53	0.602659	1.884798	0.000403	0.000403	0.001869
BP	GO:0006406	mRNA export from nucleus	95	-0.47932	-1.70972	0.000404	0.000404	0.001869
BP	GO:0071427	mRNA-containing ribonucleoprotein complex export from nuc	95	-0.47932	-1.70972	0.000404	0.000404	0.001869
BP	GO:1903793	positive regulation of anion transport	48	0.634256	1.946722	0.000408	0.000408	0.001869
BP	GO:0071166	ribonucleoprotein complex localization	112	-0.46142	-1.68586	0.000409	0.000409	0.001869
BP	GO:0071426	ribonucleoprotein complex export from nucleus	111	-0.46082	-1.68175	0.000409	0.000409	0.001869
BP	GO:0051055	negative regulation of lipid biosynthetic process	47	0.618014	1.888517	0.000409	0.000409	0.001869
BP	GO:0006733	oxidoreduction coenzyme metabolic process	43	0.698866	2.099052	0.000409	0.000409	0.001869
BP	GO:0006405	RNA export from nucleus	119	-0.4625	-1.70425	0.00041	0.00041	0.001869
BP	GO:0001736	establishment of planar polarity	122	-0.46422	-1.71792	0.000411	0.000411	0.001869
BP	GO:0007164	establishment of tissue polarity	122	-0.46422	-1.71792	0.000411	0.000411	0.001869
BP	GO:0009112	nucleobase metabolic process	34	0.688799	1.973992	0.000412	0.000412	0.001869
MF	GO:0042056	chemoattractant activity	34	0.672312	1.926741	0.000412	0.000412	0.001869
MF	GO:0016790	thiolester hydrolase activity	34	0.669971	1.920033	0.000412	0.000412	0.001869
BP	GO:0006111	regulation of gluconeogenesis	33	0.699184	1.990345	0.000412	0.000412	0.001869
BP	GO:0033762	response to glucagon	35	0.724194	2.08842	0.000413	0.000413	0.001869
MF	GO:0016620	oxidoreductase activity, acting on the aldehyde or oxo group c	35	0.699931	2.01845	0.000413	0.000413	0.001869
CC	GO:0005747	mitochondrial respiratory chain complex I	41	0.678728	2.017841	0.000413	0.000413	0.001869
CC	GO:0030964	NADH dehydrogenase complex	41	0.678728	2.017841	0.000413	0.000413	0.001869
CC	GO:0045271	respiratory chain complex I	41	0.678728	2.017841	0.000413	0.000413	0.001869
BP	GO:0070741	response to interleukin-6	39	0.658982	1.939047	0.000414	0.000414	0.001869
BP	GO:0071548	response to dexamethasone	39	0.652296	1.919374	0.000414	0.000414	0.001869
BP	GO:0042168	heme metabolic process	29	0.737263	2.034575	0.000415	0.000415	0.001869
BP	GO:0046320	regulation of fatty acid oxidation	29	0.719635	1.985927	0.000415	0.000415	0.001869
MF	GO:1901618	organic hydroxy compound transmembrane transporter activit	40	0.683772	2.022037	0.000415	0.000415	0.001869
BP	GO:1901658	glycosyl compound catabolic process	40	0.653631	1.932904	0.000415	0.000415	0.001869
BP	GO:0050873	brown fat cell differentiation	40	0.63586	1.880354	0.000415	0.000415	0.001869
BP	GO:0006084	acetyl-CoA metabolic process	36	0.70013	2.02375	0.000415	0.000415	0.001869
BP	GO:0050892	intestinal absorption	38	0.679157	1.986447	0.000415	0.000415	0.001869
MF	GO:0003954	NADH dehydrogenase activity	38	0.66057	1.932083	0.000415	0.000415	0.001869
MF	GO:0008137	NADH dehydrogenase (ubiquinone) activity	38	0.66057	1.932083	0.000415	0.000415	0.001869
MF	GO:0050136	NADH dehydrogenase (quinone) activity	38	0.66057	1.932083	0.000415	0.000415	0.001869
BP	GO:0051302	regulation of cell division	157	-0.44478	-1.7055	0.000417	0.000417	0.001869
BP	GO:0070207	protein homotrimerization	30	0.720005	1.995954	0.000418	0.000418	0.001869

BP	GO:0019362	pyridine nucleotide metabolic process	27	0.755294	2.049644	0.000418	0.000418	0.001869
BP	GO:0046496	nicotinamide nucleotide metabolic process	27	0.755294	2.049644	0.000418	0.000418	0.001869
BP	GO:0006706	steroid catabolic process	27	0.700474	1.900879	0.000418	0.000418	0.001869
CC	GO:0005681	spliceosomal complex	163	-0.4441	-1.70998	0.000419	0.000419	0.001869
BP	GO:0050820	positive regulation of coagulation	26	0.795264	2.141588	0.00042	0.00042	0.001869
BP	GO:0019359	nicotinamide nucleotide biosynthetic process	26	0.784395	2.112318	0.00042	0.00042	0.001869
BP	GO:0019363	pyridine nucleotide biosynthetic process	26	0.784395	2.112318	0.00042	0.00042	0.001869
MF	GO:0016628	oxidoreductase activity, acting on the CH-CH group of donors	26	0.779808	2.099966	0.00042	0.00042	0.001869
MF	GO:0017048	Rho GTPase binding	162	-0.44395	-1.70804	0.00042	0.00042	0.001869
BP	GO:0099111	microtubule-based transport	171	-0.40498	-1.56737	0.00042	0.00042	0.001869
BP	GO:0050657	nucleic acid transport	171	-0.42307	-1.63739	0.00042	0.00042	0.001869
BP	GO:0050658	RNA transport	171	-0.42307	-1.63739	0.00042	0.00042	0.001869
BP	GO:0030194	positive regulation of blood coagulation	25	0.79761	2.126139	0.000422	0.000422	0.001869
BP	GO:1900048	positive regulation of hemostasis	25	0.79761	2.126139	0.000422	0.000422	0.001869
BP	GO:0032369	negative regulation of lipid transport	25	0.786233	2.095812	0.000422	0.000422	0.001869
MF	GO:0016405	CoA-ligase activity	25	0.78039	2.080237	0.000422	0.000422	0.001869
MF	GO:0004033	aldo-keto reductase (NADP) activity	25	0.750486	2.000524	0.000422	0.000422	0.001869
BP	GO:0071280	cellular response to copper ion	25	0.74604	1.988673	0.000422	0.000422	0.001869
BP	GO:0008211	glucocorticoid metabolic process	25	0.735289	1.960014	0.000422	0.000422	0.001869
MF	GO:0016645	oxidoreductase activity, acting on the CH-NH group of donors	24	0.759383	2.001446	0.000425	0.000425	0.001869
BP	GO:0042219	cellular modified amino acid catabolic process	24	0.752359	1.982935	0.000425	0.000425	0.001869
BP	GO:0050996	positive regulation of lipid catabolic process	24	0.738607	1.946689	0.000425	0.000425	0.001869
BP	GO:0042430	indole-containing compound metabolic process	23	0.762791	1.993077	0.000427	0.000427	0.001869
BP	GO:0009067	aspartate family amino acid biosynthetic process	23	0.729772	1.906804	0.000427	0.000427	0.001869
BP	GO:0015740	C4-dicarboxylate transport	23	0.726866	1.899209	0.000427	0.000427	0.001869
MF	GO:0008106	alcohol dehydrogenase (NADP+) activity	20	0.782006	1.991122	0.000427	0.000427	0.001869
CC	GO:0042827	platelet dense granule	20	0.767626	1.954509	0.000427	0.000427	0.001869
BP	GO:0001763	morphogenesis of a branching structure	196	-0.39653	-1.56238	0.000428	0.000428	0.001869
BP	GO:0019400	alditol metabolic process	22	0.787514	2.041609	0.000428	0.000428	0.001869
BP	GO:0042135	neurotransmitter catabolic process	21	0.813053	2.086464	0.000428	0.000428	0.001869
BP	GO:0006067	ethanol metabolic process	21	0.783059	2.009494	0.000428	0.000428	0.001869
BP	GO:1901685	glutathione derivative metabolic process	21	0.754145	1.935294	0.000428	0.000428	0.001869
BP	GO:1901687	glutathione derivative biosynthetic process	21	0.754145	1.935294	0.000428	0.000428	0.001869
BP	GO:0009081	branched-chain amino acid metabolic process	21	0.741904	1.903881	0.000428	0.000428	0.001869
BP	GO:0009083	branched-chain amino acid catabolic process	21	0.741904	1.903881	0.000428	0.000428	0.001869
BP	GO:0060828	regulation of canonical Wnt signaling pathway	263	-0.36145	-1.47815	0.000434	0.000434	0.001891
BP	GO:0034470	ncRNA processing	322	-0.34775	-1.45293	0.000441	0.000441	0.001922
CC	GO:1902495	transmembrane transporter complex	310	-0.35473	-1.47666	0.000442	0.000442	0.001923
BP	GO:0150063	visual system development	354	-0.34432	-1.45331	0.000445	0.000445	0.001935

BP	GO:0099177	regulation of trans-synaptic signaling	421	-0.32993	-1.41579	0.000453	0.000453	0.001965
CC	GO:0030424	axon	571	-0.31727	-1.3963	0.000472	0.000472	0.002045
MF	GO:0003712	transcription coregulator activity	530	-0.31552	-1.379	0.000472	0.000472	0.002045
BP	GO:0007276	gamete generation	602	-0.32198	-1.42156	0.000481	0.000481	0.002081
CC	GO:1990904	ribonucleoprotein complex	638	-0.31078	-1.37845	0.000483	0.000483	0.002086
BP	GO:0055080	cation homeostasis	685	0.322981	1.38656	0.000503	0.000503	0.00217
BP	GO:0098771	inorganic ion homeostasis	696	0.321615	1.381361	0.000504	0.000504	0.00217
BP	GO:1901699	cellular response to nitrogen compound	650	0.323584	1.383956	0.000508	0.000508	0.002188
BP	GO:0006875	cellular metal ion homeostasis	543	0.332531	1.404824	0.000518	0.000518	0.002224
BP	GO:0035592	establishment of protein localization to extracellular region	540	0.336816	1.422029	0.000518	0.000518	0.002224
BP	GO:0071692	protein localization to extracellular region	546	0.331007	1.398481	0.000519	0.000519	0.002228
BP	GO:0006605	protein targeting	394	0.359422	1.475421	0.000539	0.000539	0.002307
MF	GO:0030246	carbohydrate binding	245	0.404689	1.587822	0.00056	0.00056	0.002395
MF	GO:0016746	transferase activity, transferring acyl groups	227	0.410391	1.596456	0.000562	0.000562	0.002402
BP	GO:0034080	CENP-A containing nucleosome assembly	23	-0.69667	-1.86458	0.000565	0.000565	0.002407
BP	GO:0061641	CENP-A containing chromatin organization	23	-0.69667	-1.86458	0.000565	0.000565	0.002407
BP	GO:0034284	response to monosaccharide	190	0.431102	1.641754	0.000568	0.000568	0.002419
BP	GO:0009746	response to hexose	185	0.4355	1.653228	0.000569	0.000569	0.002421
BP	GO:0071347	cellular response to interleukin-1	173	0.444581	1.67519	0.000572	0.000572	0.002431
CC	GO:0005720	nuclear heterochromatin	27	-0.67368	-1.86698	0.000575	0.000575	0.002439
BP	GO:0043486	histone exchange	38	-0.61603	-1.84826	0.000578	0.000578	0.002452
BP	GO:0070316	regulation of G0 to G1 transition	41	-0.62044	-1.88915	0.000581	0.000581	0.00246
MF	GO:0008376	acetylgalactosaminyltransferase activity	42	-0.61345	-1.87953	0.000583	0.000583	0.00246
BP	GO:0035150	regulation of tube size	135	0.4607	1.677876	0.000584	0.000584	0.00246
BP	GO:0035296	regulation of tube diameter	134	0.459998	1.673269	0.000584	0.000584	0.00246
BP	GO:0097746	regulation of blood vessel diameter	134	0.459998	1.673269	0.000584	0.000584	0.00246
BP	GO:0051653	spindle localization	45	-0.60846	-1.89142	0.000584	0.000584	0.00246
BP	GO:0045023	G0 to G1 transition	43	-0.61547	-1.89393	0.000587	0.000587	0.002467
CC	GO:0099146	intrinsic component of postsynaptic density membrane	49	-0.62239	-1.96414	0.000591	0.000591	0.002484
BP	GO:0009060	aerobic respiration	77	0.552701	1.843996	0.000595	0.000595	0.002495
BP	GO:0046470	phosphatidylcholine metabolic process	77	0.544687	1.817257	0.000595	0.000595	0.002495
BP	GO:0010833	telomere maintenance via telomere lengthening	74	-0.51498	-1.7634	0.000596	0.000596	0.002496
BP	GO:1905954	positive regulation of lipid localization	78	0.544616	1.817246	6.00E-04	6.00E-04	0.002496
BP	GO:0031640	killing of cells of other organism	57	0.601686	1.903328	6.00E-04	6.00E-04	0.002496
BP	GO:0046209	nitric oxide metabolic process	71	0.545089	1.794515	0.0006	0.0006	0.002496
BP	GO:0006826	iron ion transport	71	0.544553	1.792751	0.0006	0.0006	0.002496
BP	GO:0070988	demethylation	65	0.574424	1.861399	0.0006	0.0006	0.002496
CC	GO:0005657	replication fork	61	-0.55528	-1.82855	0.000601	0.000601	0.002496
MF	GO:0016811	hydrolase activity, acting on carbon-nitrogen (but not peptide)	72	0.561279	1.850179	0.000601	0.000601	0.002496

BP	GO:2001057	reactive nitrogen species metabolic process	72	0.549737	1.812131	0.000601	0.000601	0.002496
BP	GO:0000079	regulation of cyclin-dependent protein serine/threonine kinase	93	-0.47375	-1.68577	0.000603	0.000603	0.002498
BP	GO:0006749	glutathione metabolic process	53	0.58145	1.818467	0.000604	0.000604	0.002501
MF	GO:0004222	metalloendopeptidase activity	96	-0.4805	-1.71815	0.000605	0.000605	0.002501
BP	GO:0003044	regulation of systemic arterial blood pressure mediated by a c	48	0.592949	1.81994	0.000612	0.000612	0.002528
BP	GO:0072089	stem cell proliferation	111	-0.45995	-1.6786	0.000613	0.000613	0.002528
BP	GO:0045912	negative regulation of carbohydrate metabolic process	43	0.610346	1.833179	0.000614	0.000614	0.002528
BP	GO:0007292	female gamete generation	121	-0.44673	-1.65151	0.000614	0.000614	0.002528
BP	GO:0005978	glycogen biosynthetic process	45	0.594383	1.799777	0.000616	0.000616	0.002528
BP	GO:0009250	glucan biosynthetic process	45	0.594383	1.799777	0.000616	0.000616	0.002528
BP	GO:0010883	regulation of lipid storage	44	0.620833	1.870108	0.000618	0.000618	0.002528
BP	GO:0006775	fat-soluble vitamin metabolic process	42	0.626682	1.871386	0.000618	0.000618	0.002528
BP	GO:0045540	regulation of cholesterol biosynthetic process	42	0.611458	1.825925	0.000618	0.000618	0.002528
BP	GO:0106118	regulation of sterol biosynthetic process	42	0.611458	1.825925	0.000618	0.000618	0.002528
BP	GO:0010828	positive regulation of glucose transmembrane transport	41	0.634212	1.885495	0.00062	0.00062	0.002529
BP	GO:0008207	C21-steroid hormone metabolic process	37	0.664679	1.93301	0.00062	0.00062	0.002529
BP	GO:0010677	negative regulation of cellular carbohydrate metabolic process	37	0.664652	1.93293	0.00062	0.00062	0.002529
MF	GO:0071813	lipoprotein particle binding	28	0.668423	1.82926	0.000627	0.000627	0.002549
MF	GO:0071814	protein-lipid complex binding	28	0.668423	1.82926	0.000627	0.000627	0.002549
BP	GO:0007416	synapse assembly	164	-0.42394	-1.63331	0.000629	0.000629	0.00255
BP	GO:0000038	very long-chain fatty acid metabolic process	26	0.710141	1.912358	0.00063	0.00063	0.00255
BP	GO:0071377	cellular response to glucagon stimulus	26	0.705873	1.900864	0.00063	0.00063	0.00255
MF	GO:0016702	oxidoreductase activity, acting on single donors with incorpora	26	0.703876	1.895485	0.00063	0.00063	0.00255
BP	GO:0071826	ribonucleoprotein complex subunit organization	172	-0.40625	-1.57252	0.000631	0.000631	0.002553
MF	GO:0070325	lipoprotein particle receptor binding	25	0.688575	1.835493	0.000633	0.000633	0.002553
BP	GO:0006730	one-carbon metabolic process	25	0.688138	1.834327	0.000633	0.000633	0.002553
BP	GO:0003081	regulation of systemic arterial blood pressure by renin-angiote	25	0.683794	1.822746	0.000633	0.000633	0.002553
BP	GO:0048762	mesenchymal cell differentiation	206	-0.385	-1.52824	0.000639	0.000639	0.002574
MF	GO:0005244	voltage-gated ion channel activity	192	-0.38809	-1.52486	0.000641	0.000641	0.002577
MF	GO:0022832	voltage-gated channel activity	192	-0.38809	-1.52486	0.000641	0.000641	0.002577
MF	GO:0016289	CoA hydrolase activity	22	0.722978	1.874301	0.000642	0.000642	0.002578
BP	GO:0022412	cellular process involved in reproduction in multicellular organ	317	-0.3479	-1.4516	0.000662	0.000662	0.002652
CC	GO:1990351	transporter complex	317	-0.35081	-1.46376	0.000662	0.000662	0.002652
BP	GO:0001503	ossification	365	-0.33483	-1.41767	0.000669	0.000669	0.002676
BP	GO:0001701	in utero embryonic development	357	-0.33997	-1.43503	0.000672	0.000672	0.002688
MF	GO:0017016	Ras GTPase binding	395	-0.33021	-1.40857	0.000678	0.000678	0.002706
BP	GO:0050804	modulation of chemical synaptic transmission	420	-0.32767	-1.40555	0.00068	0.00068	0.002712
MF	GO:0001228	DNA-binding transcription activator activity, RNA polymerase	429	-0.32704	-1.40511	0.000686	0.000686	0.002733
CC	GO:0098793	presynapse	469	-0.31851	-1.37917	0.000693	0.000693	0.002759

BP	GO:0051129	negative regulation of cellular component organization	626	-0.30247	-1.33963	0.000725	0.000725	0.002883
BP	GO:0048589	developmental growth	620	-0.3037	-1.34393	0.000726	0.000726	0.002883
BP	GO:0051604	protein maturation	262	0.39809	1.569733	0.000742	0.000742	0.002945
MF	GO:0016747	transferase activity, transferring acyl groups other than amino-	197	0.415576	1.59199	0.00075	0.00075	0.002974
BP	GO:0070374	positive regulation of ERK1 and ERK2 cascade	189	0.420962	1.602526	0.000757	0.000757	0.002999
BP	GO:2000377	regulation of reactive oxygen species metabolic process	169	0.431351	1.620806	0.000764	0.000764	0.003017
BP	GO:0000132	establishment of mitotic spindle orientation	26	-0.67245	-1.85036	0.000764	0.000764	0.003017
BP	GO:0032467	positive regulation of cytokinesis	38	-0.61479	-1.84455	0.000771	0.000771	0.003042
BP	GO:0034724	DNA replication-independent nucleosome organization	32	-0.64994	-1.87567	0.000772	0.000772	0.003042
BP	GO:0034508	centromere complex assembly	32	-0.65965	-1.90368	0.000772	0.000772	0.003042
BP	GO:0070317	negative regulation of G0 to G1 transition	39	-0.62699	-1.89145	0.000773	0.000773	0.003042
BP	GO:0030865	cortical cytoskeleton organization	46	-0.59429	-1.85742	0.000778	0.000778	0.003054
BP	GO:0010718	positive regulation of epithelial to mesenchymal transition	46	-0.60067	-1.87736	0.000778	0.000778	0.003054
BP	GO:0090329	regulation of DNA-dependent DNA replication	50	-0.59431	-1.88331	0.00079	0.00079	0.003098
CC	GO:1990204	oxidoreductase complex	100	0.522726	1.818472	0.000791	0.000791	0.003098
BP	GO:0060333	interferon-gamma-mediated signaling pathway	89	0.532904	1.818597	0.000797	0.000797	0.003118
BP	GO:0050830	defense response to Gram-positive bacterium	84	0.536713	1.814057	8.00E-04	8.00E-04	0.003128
BP	GO:0070206	protein trimerization	50	0.588393	1.821254	0.00081	0.00081	0.003164
BP	GO:0007368	determination of left/right symmetry	111	-0.45307	-1.65347	0.000818	0.000818	0.003186
MF	GO:0004879	nuclear receptor activity	47	0.583773	1.783881	0.000818	0.000818	0.003186
MF	GO:0098531	ligand-activated transcription factor activity	47	0.583773	1.783881	0.000818	0.000818	0.003186
CC	GO:0005814	centriole	116	-0.45712	-1.67754	0.000822	0.000822	0.003195
BP	GO:0044058	regulation of digestive system process	39	0.629452	1.852155	0.000828	0.000828	0.003218
MF	GO:0016831	carboxy-lyase activity	31	0.674262	1.884881	0.000832	0.000832	0.003227
MF	GO:0015248	sterol transporter activity	31	0.664103	1.856483	0.000832	0.000832	0.003227
BP	GO:0010977	negative regulation of neuron projection development	144	-0.41769	-1.58292	0.000833	0.000833	0.003227
BP	GO:0030705	cytoskeleton-dependent intracellular transport	167	-0.41715	-1.61053	0.000836	0.000836	0.00323
MF	GO:0016701	oxidoreductase activity, acting on single donors with incorpora	27	0.673622	1.82801	0.000837	0.000837	0.00323
BP	GO:0009065	glutamine family amino acid catabolic process	27	0.6731	1.826594	0.000837	0.000837	0.00323
BP	GO:0061138	morphogenesis of a branching epithelium	182	-0.40015	-1.56449	0.000838	0.000838	0.003233
BP	GO:0016458	gene silencing	184	-0.39625	-1.55002	0.000845	0.000845	0.003255
BP	GO:0002274	myeloid leukocyte activation	616	0.320979	1.368163	0.000852	0.000852	0.003279
BP	GO:0097164	ammonium ion metabolic process	23	0.709658	1.854247	0.000853	0.000853	0.003279
BP	GO:0016137	glycoside metabolic process	20	0.750794	1.911651	0.000854	0.000854	0.003279
BP	GO:0003007	heart morphogenesis	246	-0.36412	-1.479	0.000856	0.000856	0.003285
BP	GO:0001655	urogenital system development	323	-0.34552	-1.44422	0.000881	0.000881	0.003376
BP	GO:0032535	regulation of cellular component size	343	-0.341	-1.43315	0.00089	0.00089	0.00341
BP	GO:0018205	peptidyl-lysine modification	338	-0.34099	-1.43083	0.000893	0.000893	0.003417
MF	GO:0001216	DNA-binding transcription activator activity	430	-0.32643	-1.40278	0.000914	0.000914	0.003494

BP	GO:0031055	chromatin remodeling at centromere	25	-0.67456	-1.83926	0.00095	0.00095	0.003626
BP	GO:0006336	DNA replication-independent nucleosome assembly	31	-0.64772	-1.85783	0.000962	0.000962	0.003669
BP	GO:0030866	cortical actin cytoskeleton organization	41	-0.59475	-1.81094	0.000969	0.000969	0.003686
BP	GO:0045143	homologous chromosome segregation	41	-0.60186	-1.83258	0.000969	0.000969	0.003686
BP	GO:0050729	positive regulation of inflammatory response	137	0.437863	1.598348	0.00097	0.00097	0.003687
BP	GO:0002698	negative regulation of immune effector process	116	0.474024	1.689306	0.000974	0.000974	0.003699
BP	GO:0022600	digestive system process	97	0.51997	1.798323	0.000995	0.000995	0.003771
BP	GO:1901570	fatty acid derivative biosynthetic process	89	0.511511	1.745592	0.000996	0.000996	0.003771
BP	GO:2001022	positive regulation of response to DNA damage stimulus	83	-0.47806	-1.67269	0.000997	0.000997	0.003771
BP	GO:0097549	chromatin organization involved in negative regulation of trans	72	-0.52085	-1.77065	0.000997	0.000997	0.003771
CC	GO:0000313	organellar ribosome	82	0.515373	1.734914	0.000999	0.000999	0.003771
CC	GO:0005761	mitochondrial ribosome	82	0.515373	1.734914	0.000999	0.000999	0.003771
BP	GO:0072395	signal transduction involved in cell cycle checkpoint	66	-0.54117	-1.80784	0.001001	0.001001	0.003771
BP	GO:0001942	hair follicle development	86	-0.48593	-1.70761	0.001002	0.001002	0.003771
BP	GO:0060021	roof of mouth development	87	-0.47901	-1.68593	0.001003	0.001003	0.003771
BP	GO:0098773	skin epidermis development	87	-0.4802	-1.69013	0.001003	0.001003	0.003771
CC	GO:0099634	postsynaptic specialization membrane	97	-0.46179	-1.65452	0.001005	0.001005	0.003771
BP	GO:0043255	regulation of carbohydrate biosynthetic process	81	0.526173	1.764841	0.001005	0.001005	0.003771
BP	GO:0022404	molting cycle process	88	-0.47738	-1.68278	0.001007	0.001007	0.003771
BP	GO:0022405	hair cycle process	88	-0.47738	-1.68278	0.001007	0.001007	0.003771
BP	GO:0071156	regulation of cell cycle arrest	99	-0.45555	-1.63559	0.001009	0.001009	0.003774
MF	GO:0016788	hydrolase activity, acting on ester bonds	658	0.316676	1.355274	0.001017	0.001017	0.0038
BP	GO:0046326	positive regulation of glucose import	35	0.64284	1.853812	0.001033	0.001033	0.003854
BP	GO:0046688	response to copper ion	37	0.635099	1.846984	0.001034	0.001034	0.003854
MF	GO:0048020	CCR chemokine receptor binding	39	0.624399	1.837288	0.001035	0.001035	0.003854
BP	GO:0071549	cellular response to dexamethasone stimulus	29	0.687427	1.897046	0.001036	0.001036	0.003854
MF	GO:0005504	fatty acid binding	32	0.659708	1.861708	0.001036	0.001036	0.003854
BP	GO:0002790	peptide secretion	571	0.323984	1.371661	0.001041	0.001041	0.003861
BP	GO:0009164	nucleoside catabolic process	31	0.645728	1.805116	0.001041	0.001041	0.003861
BP	GO:0003013	circulatory system process	521	0.335977	1.41293	0.001043	0.001043	0.003866
BP	GO:0022618	ribonucleoprotein complex assembly	165	-0.4107	-1.58404	0.001044	0.001044	0.003866
BP	GO:0019827	stem cell population maintenance	154	-0.40855	-1.56031	0.001049	0.001049	0.003882
MF	GO:0004364	glutathione transferase activity	25	0.675411	1.800401	0.001055	0.001055	0.0039
BP	GO:0002446	neutrophil mediated immunity	476	0.34108	1.42384	0.001057	0.001057	0.003902
MF	GO:0051537	2 iron, 2 sulfur cluster binding	20	0.737599	1.878054	0.001067	0.001067	0.003932
BP	GO:0050995	negative regulation of lipid catabolic process	20	0.729496	1.857421	0.001067	0.001067	0.003932
BP	GO:0031348	negative regulation of defense response	193	0.406911	1.555538	0.001123	0.001123	0.004133
BP	GO:0060713	labyrinthine layer morphogenesis	20	-0.7197	-1.863	0.001129	0.001129	0.004151
BP	GO:0071248	cellular response to metal ion	179	0.426236	1.613201	0.001136	0.001136	0.004175

BP	GO:0009749	response to glucose	180	0.426356	1.612764	0.001142	0.001142	0.00419
BP	GO:0036297	interstrand cross-link repair	41	-0.59096	-1.7994	0.001162	0.001162	0.004262
BP	GO:0045740	positive regulation of DNA replication	34	-0.6151	-1.79806	0.001166	0.001166	0.004268
BP	GO:0098534	centriole assembly	34	-0.61536	-1.79882	0.001166	0.001166	0.004268
BP	GO:0007131	reciprocal meiotic recombination	43	-0.58437	-1.79823	0.001173	0.001173	0.004288
BP	GO:0090102	cochlea development	50	-0.57535	-1.82322	0.001185	0.001185	0.004327
MF	GO:0015294	solute:cation symporter activity	102	0.50143	1.747637	0.001189	0.001189	0.004337
CC	GO:0005623	cell	79	0.526728	1.761157	0.001203	0.001203	0.004385
BP	GO:0071158	positive regulation of cell cycle arrest	76	-0.50218	-1.72579	0.001204	0.001204	0.004385
BP	GO:1904029	regulation of cyclin-dependent protein kinase activity	97	-0.45688	-1.63694	0.001206	0.001206	0.004386
BP	GO:0000018	regulation of DNA recombination	77	-0.49058	-1.68966	0.001209	0.001209	0.004394
BP	GO:0009306	protein secretion	539	0.329191	1.38912	0.001213	0.001213	0.004402
BP	GO:0042303	molting cycle	111	-0.44463	-1.62267	0.001226	0.001226	0.004444
BP	GO:0042633	hair cycle	111	-0.44463	-1.62267	0.001226	0.001226	0.004444
BP	GO:0007224	smoothened signaling pathway	127	-0.4227	-1.57533	0.00123	0.00123	0.004451
BP	GO:0032892	positive regulation of organic acid transport	33	0.644026	1.833329	0.001237	0.001237	0.004468
BP	GO:0010907	positive regulation of glucose metabolic process	33	0.643703	1.832409	0.001237	0.001237	0.004468
MF	GO:0022843	voltage-gated cation channel activity	137	-0.41661	-1.57077	0.001238	0.001238	0.004469
BP	GO:0030177	positive regulation of Wnt signaling pathway	168	-0.39623	-1.53066	0.001255	0.001255	0.004527
BP	GO:1901888	regulation of cell junction assembly	177	-0.39535	-1.53755	0.001266	0.001266	0.00456
BP	GO:0046677	response to antibiotic	306	0.380914	1.526884	0.001279	0.001279	0.004602
BP	GO:0046135	pyrimidine nucleoside catabolic process	22	0.706919	1.832668	0.001284	0.001284	0.004612
BP	GO:0043567	regulation of insulin-like growth factor receptor signaling path	22	0.698114	1.809842	0.001284	0.001284	0.004612
BP	GO:0044818	mitotic G2/M transition checkpoint	24	-0.67807	-1.83266	0.001321	0.001321	0.004741
MF	GO:0016298	lipase activity	116	0.469674	1.673802	0.001364	0.001364	0.004889
BP	GO:0044264	cellular polysaccharide metabolic process	99	0.497034	1.72509	0.001387	0.001387	0.004958
BP	GO:0048232	male gamete generation	476	-0.30506	-1.32267	0.001387	0.001387	0.004958
BP	GO:0008015	blood circulation	510	0.332192	1.395021	0.001387	0.001387	0.004958
BP	GO:0018958	phenol-containing compound metabolic process	96	0.497667	1.719317	0.001388	0.001388	0.004958
BP	GO:0003073	regulation of systemic arterial blood pressure	89	0.507609	1.732277	0.001394	0.001394	0.004974
BP	GO:0006278	RNA-dependent DNA biosynthetic process	67	-0.52438	-1.75816	0.001401	0.001401	0.00499
BP	GO:0034502	protein localization to chromosome	66	-0.5255	-1.75549	0.001401	0.001401	0.00499
BP	GO:2000779	regulation of double-strand break repair	61	-0.54558	-1.79658	0.001403	0.001403	0.00499
BP	GO:0010771	negative regulation of cell morphogenesis involved in different	93	-0.46141	-1.64188	0.001406	0.001406	0.004999
CC	GO:0030175	filopodium	99	-0.45157	-1.62128	0.001412	0.001412	0.005016
MF	GO:0004601	peroxidase activity	52	0.564693	1.758312	0.001417	0.001417	0.005026
BP	GO:0061061	muscle structure development	593	-0.30006	-1.32233	0.00144	0.00144	0.005102
BP	GO:0006099	tricarboxylic acid cycle	34	0.624987	1.791116	0.001441	0.001441	0.005102
BP	GO:0072073	kidney epithelium development	140	-0.40866	-1.54485	0.001449	0.001449	0.005126

BP	GO:0031331	positive regulation of cellular catabolic process	342	0.365418	1.480876	0.001452	0.001452	0.005132
BP	GO:0061351	neural precursor cell proliferation	144	-0.4137	-1.56781	0.001458	0.001458	0.005149
BP	GO:0035567	non-canonical Wnt signaling pathway	146	-0.41374	-1.57023	0.001462	0.001462	0.005156
BP	GO:0007050	cell cycle arrest	214	-0.37047	-1.47694	0.001497	0.001497	0.005276
BP	GO:0016072	rRNA metabolic process	198	-0.3746	-1.47841	0.001499	0.001499	0.005277
MF	GO:0008574	ATP-dependent microtubule motor activity, plus-end-directed	26	-0.63724	-1.75346	0.001527	0.001527	0.005372
BP	GO:0072001	renal system development	286	-0.35128	-1.45057	0.001537	0.001537	0.0054
BP	GO:0018209	peptidyl-serine modification	306	-0.34597	-1.43811	0.001546	0.001546	0.005425
CC	GO:0045211	postsynaptic membrane	307	-0.34393	-1.43019	0.001547	0.001547	0.005425
BP	GO:0043010	camera-type eye development	308	-0.33998	-1.41418	0.00155	0.00155	0.005428
BP	GO:0010569	regulation of double-strand break repair via homologous reco	35	-0.61579	-1.81502	0.00155	0.00155	0.005428
BP	GO:0032103	positive regulation of response to external stimulus	484	0.336609	1.408233	0.001575	0.001575	0.005508
MF	GO:0031267	small GTPase binding	409	-0.32045	-1.3713	0.001584	0.001584	0.005534
CC	GO:0005811	lipid droplet	76	0.525766	1.750164	0.001594	0.001594	0.005563
BP	GO:0046323	glucose import	59	0.545677	1.736404	0.001597	0.001597	0.005563
BP	GO:0072401	signal transduction involved in DNA integrity checkpoint	65	-0.52927	-1.76363	0.001598	0.001598	0.005563
BP	GO:0072422	signal transduction involved in DNA damage checkpoint	65	-0.52927	-1.76363	0.001598	0.001598	0.005563
MF	GO:0016684	oxidoreductase activity, acting on peroxide as acceptor	56	0.559904	1.765299	0.001608	0.001608	0.005592
BP	GO:0031109	microtubule polymerization or depolymerization	102	-0.44876	-1.61809	0.001615	0.001615	0.005611
BP	GO:0009799	specification of symmetry	121	-0.43106	-1.59356	0.001638	0.001638	0.005687
BP	GO:0016571	histone methylation	116	-0.43237	-1.58669	0.001643	0.001643	0.005698
BP	GO:0071276	cellular response to cadmium ion	36	0.624059	1.803864	0.00166	0.00166	0.005747
MF	GO:0019840	isoprenoid binding	36	0.620645	1.793996	0.00166	0.00166	0.005747
BP	GO:0098727	maintenance of cell number	156	-0.39828	-1.52528	0.001672	0.001672	0.005782
BP	GO:0071383	cellular response to steroid hormone stimulus	230	0.391477	1.525736	0.001684	0.001684	0.00582
BP	GO:0071241	cellular response to inorganic substance	202	0.396592	1.521993	0.001689	0.001689	0.00583
BP	GO:0050777	negative regulation of immune response	143	0.429672	1.582695	0.00173	0.00173	0.005966
BP	GO:0016572	histone phosphorylation	35	-0.60816	-1.79253	0.001744	0.001744	0.006002
CC	GO:0150034	distal axon	273	-0.35453	-1.4558	0.001748	0.001748	0.006002
BP	GO:0035825	homologous recombination	44	-0.58173	-1.8013	0.001748	0.001748	0.006002
BP	GO:0035088	establishment or maintenance of apical/basal cell polarity	42	-0.57418	-1.75919	0.001748	0.001748	0.006002
BP	GO:0061245	establishment or maintenance of bipolar cell polarity	42	-0.57418	-1.75919	0.001748	0.001748	0.006002
CC	GO:0098552	side of membrane	487	0.333093	1.393364	0.001756	0.001756	0.006024
BP	GO:0002283	neutrophil activation involved in immune response	466	0.336686	1.403939	0.001761	0.001761	0.006033
BP	GO:0070192	chromosome organization involved in meiotic cell cycle	49	-0.5583	-1.7619	0.001774	0.001774	0.006074
MF	GO:0005261	cation channel activity	309	-0.33832	-1.40696	0.001777	0.001777	0.006077
BP	GO:1903409	reactive oxygen species biosynthetic process	102	0.483392	1.68477	0.001783	0.001783	0.006092
BP	GO:0006644	phospholipid metabolic process	395	0.346686	1.423612	0.001794	0.001794	0.006124
BP	GO:0070125	mitochondrial translational elongation	86	0.500369	1.698053	0.001796	0.001796	0.006127

BP	GO:0048247	lymphocyte chemotaxis	60	0.543438	1.735394	0.0018	0.0018	0.006133
MF	GO:0015370	solute:sodium symporter activity	73	0.528622	1.746378	0.001805	0.001805	0.006146
BP	GO:0034660	ncRNA metabolic process	390	-0.3232	-1.37724	0.001807	0.001807	0.006146
BP	GO:0061326	renal tubule development	95	-0.4535	-1.61762	0.001817	0.001817	0.006175
CC	GO:0032993	protein-DNA complex	104	-0.43782	-1.58343	0.001823	0.001823	0.006191
BP	GO:0048260	positive regulation of receptor-mediated endocytosis	49	0.565856	1.745531	0.001826	0.001826	0.006194
MF	GO:0015267	channel activity	443	-0.31236	-1.34667	0.001828	0.001828	0.006196
MF	GO:0022803	passive transmembrane transporter activity	444	-0.31217	-1.34509	0.001837	0.001837	0.00622
MF	GO:0042277	peptide binding	272	0.377411	1.496164	0.001839	0.001839	0.00622
MF	GO:0005548	phospholipid transporter activity	47	0.566687	1.73167	0.001841	0.001841	0.006223
MF	GO:0005501	retinoid binding	35	0.624702	1.801505	0.001859	0.001859	0.006277
BP	GO:0015893	drug transport	199	0.405094	1.55289	0.001874	0.001874	0.006321
BP	GO:0070555	response to interleukin-1	196	0.399198	1.527873	0.001877	0.001877	0.006325
BP	GO:0051984	positive regulation of chromosome segregation	25	-0.64044	-1.74622	0.0019	0.0019	0.006398
BP	GO:0031507	heterochromatin assembly	40	-0.57815	-1.75396	0.001931	0.001931	0.006491
BP	GO:0036230	granulocyte activation	479	0.33329	1.392383	0.001931	0.001931	0.006491
BP	GO:0002791	regulation of peptide secretion	461	0.336792	1.402639	0.001942	0.001942	0.006522
BP	GO:0043312	neutrophil degranulation	463	0.334824	1.394995	0.001944	0.001944	0.006522
MF	GO:0005089	Rho guanyl-nucleotide exchange factor activity	74	-0.48851	-1.67277	0.001987	0.001987	0.006661
BP	GO:0009913	epidermal cell differentiation	333	-0.33218	-1.39361	0.001994	0.001994	0.006674
BP	GO:0032204	regulation of telomere maintenance	72	-0.50284	-1.70944	0.001994	0.001994	0.006674
MF	GO:0051213	dioxygenase activity	78	0.516102	1.722102	0.002	0.002	0.006687
BP	GO:0030900	forebrain development	369	-0.32339	-1.37074	0.002008	0.002008	0.006706
BP	GO:0072080	nephron tubule development	93	-0.44728	-1.59159	0.002009	0.002009	0.006706
BP	GO:0072163	mesonephric epithelium development	98	-0.44545	-1.59705	0.002016	0.002016	0.006711
BP	GO:0072164	mesonephric tubule development	98	-0.44545	-1.59705	0.002016	0.002016	0.006711
BP	GO:0034968	histone lysine methylation	96	-0.44435	-1.58889	0.002016	0.002016	0.006711
BP	GO:0050704	regulation of interleukin-1 secretion	50	0.561359	1.737575	0.002025	0.002025	0.006731
BP	GO:0072577	endothelial cell apoptotic process	51	0.557571	1.730505	0.002028	0.002028	0.006731
BP	GO:0010676	positive regulation of cellular carbohydrate metabolic process	51	0.557034	1.728839	0.002028	0.002028	0.006731
BP	GO:0009855	determination of bilateral symmetry	120	-0.42748	-1.5788	0.002042	0.002042	0.006773
CC	GO:0034399	nuclear periphery	124	-0.41801	-1.55095	0.002058	0.002058	0.006818
BP	GO:2001252	positive regulation of chromosome organization	155	-0.39808	-1.52268	0.002089	0.002089	0.006915
BP	GO:0031345	negative regulation of cell projection organization	168	-0.38785	-1.4983	0.002092	0.002092	0.006921
MF	GO:0008022	protein C-terminus binding	181	-0.38164	-1.49081	0.0021	0.0021	0.006941
BP	GO:0042119	neutrophil activation	474	0.335504	1.40016	0.002116	0.002116	0.006987
BP	GO:0051567	histone H3-K9 methylation	36	-0.59667	-1.77228	0.002122	0.002122	0.007001
MF	GO:0047617	acyl-CoA hydrolase activity	20	0.705509	1.796347	0.002134	0.002134	0.007028
BP	GO:0010884	positive regulation of lipid storage	20	0.704694	1.794271	0.002134	0.002134	0.007028

BP	GO:0005976	polysaccharide metabolic process	110	0.467114	1.652579	0.002149	0.002149	0.00707
BP	GO:0050918	positive chemotaxis	63	0.53321	1.720018	0.002188	0.002188	0.007192
BP	GO:0044070	regulation of anion transport	89	0.495338	1.690402	0.002191	0.002191	0.007195
BP	GO:0031100	animal organ regeneration	70	0.513714	1.68695	0.002196	0.002196	0.007204
BP	GO:0007004	telomere maintenance via telomerase	65	-0.52256	-1.74126	0.002197	0.002197	0.007204
BP	GO:0018105	peptidyl-serine phosphorylation	290	-0.34324	-1.41757	0.002207	0.002207	0.007228
BP	GO:0001657	ureteric bud development	97	-0.44885	-1.60818	0.002211	0.002211	0.007235
MF	GO:0003774	motor activity	134	-0.40406	-1.518	0.002263	0.002263	0.0074
MF	GO:0001530	lipopolysaccharide binding	29	0.637652	1.759685	0.00228	0.00228	0.007448
BP	GO:0050832	defense response to fungus	36	0.611307	1.767002	0.002283	0.002283	0.007451
BP	GO:0034765	regulation of ion transmembrane transport	448	-0.30803	-1.3284	0.002288	0.002288	0.007463
BP	GO:0010543	regulation of platelet activation	30	0.627179	1.738629	0.002298	0.002298	0.007488
BP	GO:0050708	regulation of protein secretion	434	0.339524	1.405356	0.002312	0.002312	0.007528
BP	GO:0045005	DNA-dependent DNA replication maintenance of fidelity	32	-0.60914	-1.75791	0.002317	0.002317	0.007532
BP	GO:0048333	mesodermal cell differentiation	32	-0.6114	-1.76442	0.002317	0.002317	0.007532
CC	GO:0070971	endoplasmic reticulum exit site	23	0.67914	1.774508	0.002346	0.002346	0.007617
BP	GO:0120162	positive regulation of cold-induced thermogenesis	96	0.485722	1.678051	0.00238	0.00238	0.007719
BP	GO:0006873	cellular ion homeostasis	623	0.314452	1.341322	0.002381	0.002381	0.007719
BP	GO:0050829	defense response to Gram-negative bacterium	76	0.516278	1.718582	0.00239	0.00239	0.007742
BP	GO:0071384	cellular response to corticosteroid stimulus	57	0.54784	1.732996	0.0024	0.0024	0.007766
BP	GO:0001822	kidney development	271	-0.35193	-1.44387	0.002403	0.002403	0.007768
CC	GO:0031594	neuromuscular junction	76	-0.48389	-1.66294	0.002409	0.002409	0.007779
BP	GO:0018022	peptidyl-lysine methylation	97	-0.44678	-1.60075	0.002412	0.002412	0.007779
MF	GO:0048365	Rac GTPase binding	63	-0.51739	-1.71273	0.002413	0.002413	0.007779
BP	GO:0046324	regulation of glucose import	52	0.557008	1.734384	0.002429	0.002429	0.007825
BP	GO:0006897	endocytosis	526	0.321545	1.353504	0.002431	0.002431	0.007825
BP	GO:0002275	myeloid cell activation involved in immune response	519	0.322721	1.356629	0.002435	0.002435	0.007825
BP	GO:0097305	response to alcohol	224	0.385665	1.498691	0.002435	0.002435	0.007825
BP	GO:0007413	axonal fasciculation	22	-0.6577	-1.7434	0.002439	0.002439	0.007825
BP	GO:0106030	neuron projection fasciculation	22	-0.6577	-1.7434	0.002439	0.002439	0.007825
BP	GO:2000351	regulation of endothelial cell apoptotic process	45	0.570934	1.728774	0.002466	0.002466	0.007902
MF	GO:0015085	calcium ion transmembrane transporter activity	136	-0.39934	-1.50277	0.002476	0.002476	0.00793
BP	GO:0010743	regulation of macrophage derived foam cell differentiation	29	0.634764	1.751714	0.002487	0.002487	0.007957
MF	GO:0015079	potassium ion transmembrane transporter activity	153	-0.38979	-1.48689	0.00253	0.00253	0.008086
BP	GO:0071805	potassium ion transmembrane transport	201	-0.36937	-1.46095	0.00257	0.00257	0.008207
BP	GO:2000379	positive regulation of reactive oxygen species metabolic proce	92	0.482863	1.655216	0.002585	0.002585	0.00825
BP	GO:0050764	regulation of phagocytosis	91	0.484216	1.657444	0.002589	0.002589	0.008256
CC	GO:0071013	catalytic step 2 spliceosome	80	-0.46863	-1.62854	0.002592	0.002592	0.008258
CC	GO:0098839	postsynaptic density membrane	70	-0.49207	-1.66298	0.002603	0.002603	0.008286

BP	GO:0046034	ATP metabolic process	249	0.377535	1.482504	0.002606	0.002606	0.008288
BP	GO:0007264	small GTPase mediated signal transduction	543	-0.2949	-1.29156	0.002613	0.002613	0.008303
MF	GO:0003707	steroid hormone receptor activity	55	0.548918	1.72584	0.002617	0.002617	0.008303
BP	GO:0071385	cellular response to glucocorticoid stimulus	55	0.543857	1.709931	0.002617	0.002617	0.008303
BP	GO:0006584	catecholamine metabolic process	49	0.554786	1.711383	0.002637	0.002637	0.008352
BP	GO:0009712	catechol-containing compound metabolic process	49	0.554786	1.711383	0.002637	0.002637	0.008352
BP	GO:0030010	establishment of cell polarity	126	-0.41145	-1.53007	0.002667	0.002667	0.00844
BP	GO:0060914	heart formation	28	-0.62536	-1.74978	0.002685	0.002685	0.008487
BP	GO:0007019	microtubule depolymerization	38	-0.58233	-1.74715	0.002699	0.002699	0.008526
BP	GO:0003338	metanephros morphogenesis	32	-0.60439	-1.74421	0.002704	0.002704	0.008533
BP	GO:2000352	negative regulation of endothelial cell apoptotic process	27	0.65091	1.766375	0.002719	0.002719	0.008574
MF	GO:0019887	protein kinase regulator activity	163	-0.38539	-1.48392	0.002727	0.002727	0.008591
BP	GO:0070828	heterochromatin organization	48	-0.55098	-1.7335	0.002743	0.002743	0.008637
MF	GO:0042562	hormone binding	98	0.470667	1.630471	0.002777	0.002777	0.008728
BP	GO:0071294	cellular response to zinc ion	22	0.682536	1.769454	0.002782	0.002782	0.008728
BP	GO:0032373	positive regulation of sterol transport	22	0.681019	1.765522	0.002782	0.002782	0.008728
BP	GO:0032376	positive regulation of cholesterol transport	22	0.681019	1.765522	0.002782	0.002782	0.008728
BP	GO:0050810	regulation of steroid biosynthetic process	85	0.491271	1.665047	0.002793	0.002793	0.008755
BP	GO:0008306	associative learning	76	-0.48047	-1.65116	0.00281	0.00281	0.008802
BP	GO:0055081	anion homeostasis	55	0.541911	1.703812	0.002819	0.002819	0.008821
CC	GO:0030425	dendrite	557	-0.29353	-1.28929	0.002841	0.002841	0.008883
BP	GO:0031123	RNA 3'-end processing	121	-0.41522	-1.53503	0.002867	0.002867	0.008958
MF	GO:1901505	carbohydrate derivative transmembrane transporter activity	42	0.582092	1.738232	0.002884	0.002884	0.009004
BP	GO:0030003	cellular cation homeostasis	610	0.314176	1.338631	0.002891	0.002891	0.009018
BP	GO:0007517	muscle organ development	360	-0.32205	-1.36059	0.002908	0.002908	0.009063
BP	GO:0048641	regulation of skeletal muscle tissue development	45	-0.56353	-1.75175	0.002921	0.002921	0.009096
MF	GO:0022853	active ion transmembrane transporter activity	227	0.379761	1.477301	0.002998	0.002998	0.009327
BP	GO:0045913	positive regulation of carbohydrate metabolic process	71	0.508272	1.673309	0.003001	0.003001	0.00933
BP	GO:1901264	carbohydrate derivative transport	73	0.501529	1.656873	0.003009	0.003009	0.009346
BP	GO:0045682	regulation of epidermis development	77	-0.46732	-1.60957	0.003022	0.003022	0.00938
BP	GO:0060071	Wnt signaling pathway, planar cell polarity pathway	108	-0.42353	-1.53888	0.003066	0.003066	0.009508
BP	GO:0009954	proximal/distal pattern formation	31	-0.61704	-1.76985	0.003079	0.003079	0.009539
CC	GO:0097447	dendritic tree	559	-0.29297	-1.28668	0.003084	0.003084	0.009548
BP	GO:0071354	cellular response to interleukin-6	35	0.604218	1.742433	0.003098	0.003098	0.009582
MF	GO:0003713	transcription coactivator activity	304	-0.33167	-1.37654	0.003108	0.003108	0.009606
CC	GO:0099240	intrinsic component of synaptic membrane	159	-0.38738	-1.48811	0.003135	0.003135	0.009674
BP	GO:1905268	negative regulation of chromatin organization	48	-0.5443	-1.71248	0.003135	0.003135	0.009674
BP	GO:0006364	rRNA processing	190	-0.3719	-1.46079	0.003177	0.003177	0.009762
BP	GO:1904356	regulation of telomere maintenance via telomere lengthening	55	-0.52438	-1.69541	0.003178	0.003178	0.009762

BP	GO:0072413	signal transduction involved in mitotic cell cycle checkpoint	54	-0.52355	-1.68456	0.00318	0.00318	0.009762
BP	GO:1902402	signal transduction involved in mitotic DNA damage checkpoir	54	-0.52355	-1.68456	0.00318	0.00318	0.009762
BP	GO:1902403	signal transduction involved in mitotic DNA integrity checkpoir	54	-0.52355	-1.68456	0.00318	0.00318	0.009762
BP	GO:0016266	O-glycan processing	54	-0.52391	-1.68571	0.00318	0.00318	0.009762
BP	GO:0006979	response to oxidative stress	412	0.338647	1.396297	0.00322	0.00322	0.009878
BP	GO:0045742	positive regulation of epidermal growth factor receptor signali	28	-0.6222	-1.74093	0.00326	0.00326	0.009992
BP	GO:0072009	nephron epithelium development	109	-0.42567	-1.5492	0.003263	0.003263	0.009994
BP	GO:0034656	nucleobase-containing small molecule catabolic process	47	0.556142	1.699449	0.003273	0.003273	0.010016
BP	GO:0007099	centriole replication	32	-0.59667	-1.72194	0.003283	0.003283	0.01003
BP	GO:0051294	establishment of spindle orientation	32	-0.59953	-1.73019	0.003283	0.003283	0.01003
BP	GO:0045471	response to ethanol	122	0.43829	1.573296	0.003307	0.003307	0.010096
BP	GO:0006691	leukotriene metabolic process	28	0.631249	1.727526	0.003342	0.003342	0.010194
BP	GO:0015749	monosaccharide transmembrane transport	101	0.467255	1.626704	0.003373	0.003373	0.010279
BP	GO:1904888	cranial skeletal system development	65	-0.4956	-1.65141	0.003396	0.003396	0.010335
BP	GO:0051125	regulation of actin nucleation	24	-0.63354	-1.71228	0.003397	0.003397	0.010335
MF	GO:0004602	glutathione peroxidase activity	21	0.687048	1.763108	0.003426	0.003426	0.010415
BP	GO:0090175	regulation of establishment of planar polarity	109	-0.41893	-1.52464	0.003467	0.003467	0.010532
BP	GO:0034612	response to tumor necrosis factor	282	0.364748	1.450503	0.003493	0.003493	0.0106
BP	GO:0007498	mesoderm development	122	-0.41135	-1.52229	0.003497	0.003497	0.010603
BP	GO:0070873	regulation of glycogen metabolic process	33	0.609231	1.734281	0.003504	0.003504	0.010614
MF	GO:0030695	GTPase regulator activity	284	-0.33658	-1.3889	0.003506	0.003506	0.010614
BP	GO:0010970	transport along microtubule	148	-0.39367	-1.49641	0.003556	0.003556	0.010757
BP	GO:0034219	carbohydrate transmembrane transport	103	0.463623	1.616111	0.003583	0.003583	0.01083
BP	GO:0043044	ATP-dependent chromatin remodeling	66	-0.48975	-1.63606	0.003603	0.003603	0.010882
BP	GO:0001656	metanephros development	90	-0.43757	-1.5484	0.00361	0.00361	0.010894
CC	GO:0042611	MHC protein complex	23	0.670015	1.750665	0.003626	0.003626	0.010932
CC	GO:0034703	cation channel complex	207	-0.36308	-1.44195	0.003633	0.003633	0.010947
BP	GO:0060627	regulation of vesicle-mediated transport	514	0.319262	1.34147	0.003646	0.003646	0.010976
BP	GO:0001678	cellular glucose homeostasis	137	0.421004	1.536808	0.003685	0.003685	0.011085
BP	GO:0060711	labyrinthine layer development	44	-0.55416	-1.71594	0.003691	0.003691	0.011093
BP	GO:0000041	transition metal ion transport	118	0.43977	1.57071	0.003703	0.003703	0.011121
BP	GO:0050714	positive regulation of protein secretion	255	0.370929	1.458725	0.003714	0.003714	0.011145
BP	GO:0006739	NADP metabolic process	29	0.624947	1.724623	0.003731	0.003731	0.011185
BP	GO:0006888	endoplasmic reticulum to Golgi vesicle-mediated transport	199	0.386866	1.483014	0.003747	0.003747	0.011227
BP	GO:0048592	eye morphogenesis	146	-0.39741	-1.50825	0.003759	0.003759	0.011251
BP	GO:0072676	lymphocyte migration	102	0.461867	1.60975	0.003764	0.003764	0.011258
BP	GO:0072431	signal transduction involved in mitotic G1 DNA damage check	53	-0.5259	-1.68471	0.003774	0.003774	0.011269
BP	GO:1902400	intracellular signal transduction involved in G1 DNA damage c	53	-0.5259	-1.68471	0.003774	0.003774	0.011269
BP	GO:0071396	cellular response to lipid	559	0.315299	1.334323	0.003802	0.003802	0.011344

BP	GO:0001659	temperature homeostasis	171	0.404039	1.520007	0.003813	0.003813	0.011369
MF	GO:0032934	sterol binding	56	0.527682	1.66371	0.003818	0.003818	0.011375
BP	GO:0071320	cellular response to cAMP	51	0.53903	1.672961	0.003852	0.003852	0.011464
BP	GO:0090092	regulation of transmembrane receptor protein serine/threonin	220	-0.35768	-1.43192	0.003857	0.003857	0.011464
BP	GO:0048608	reproductive structure development	413	-0.30527	-1.30709	0.003857	0.003857	0.011464
BP	GO:0140053	mitochondrial gene expression	140	0.414639	1.519418	0.003868	0.003868	0.011485
BP	GO:1903844	regulation of cellular response to transforming growth factor b	109	-0.41346	-1.50476	0.003875	0.003875	0.011498
BP	GO:0009395	phospholipid catabolic process	38	0.583622	1.707019	0.003946	0.003946	0.011699
BP	GO:0032210	regulation of telomere maintenance via telomerase	50	-0.53135	-1.68381	0.003949	0.003949	0.0117
BP	GO:0008645	hexose transmembrane transport	99	0.461029	1.600125	0.003964	0.003964	0.011732
BP	GO:0001578	microtubule bundle formation	69	-0.47946	-1.61661	0.003989	0.003989	0.011798
CC	GO:0016363	nuclear matrix	102	-0.42076	-1.51713	0.004037	0.004037	0.011931
MF	GO:0008378	galactosyltransferase activity	31	-0.59908	-1.71834	0.004041	0.004041	0.011933
BP	GO:0007218	neuropeptide signaling pathway	100	-0.42423	-1.52429	0.004045	0.004045	0.011937
BP	GO:0030036	actin cytoskeleton organization	617	-0.29051	-1.28508	0.00411	0.00411	0.012119
BP	GO:0072006	nephron development	141	-0.39356	-1.48754	0.004155	0.004155	0.012242
BP	GO:1904659	glucose transmembrane transport	96	0.464314	1.604091	0.004165	0.004165	0.012253
BP	GO:0043200	response to amino acid	98	0.461961	1.60031	0.004166	0.004166	0.012253
BP	GO:0006517	protein deglycosylation	27	0.638055	1.731493	0.004183	0.004183	0.012295
BP	GO:0050701	interleukin-1 secretion	58	0.525536	1.667896	0.004187	0.004187	0.012297
BP	GO:0006809	nitric oxide biosynthetic process	67	0.508261	1.65665	0.004197	0.004197	0.012315
BP	GO:0003071	renal system process involved in regulation of systemic arteria	25	0.651608	1.736951	0.004221	0.004221	0.012377
BP	GO:0002793	positive regulation of peptide secretion	274	0.363098	1.439124	0.00424	0.00424	0.012423
BP	GO:0061458	reproductive system development	416	-0.30398	-1.30324	0.0043	0.0043	0.01258
MF	GO:0005262	calcium channel activity	121	-0.40221	-1.48692	0.004301	0.004301	0.01258
BP	GO:0002444	myeloid leukocyte mediated immunity	527	0.315895	1.329856	0.00434	0.00434	0.012686
BP	GO:0045814	negative regulation of gene expression, epigenetic	56	-0.50467	-1.63572	0.004377	0.004377	0.012784
BP	GO:0042440	pigment metabolic process	68	0.505508	1.652458	0.004402	0.004402	0.012846
CC	GO:0000152	nuclear ubiquitin ligase complex	41	-0.54922	-1.6723	0.004456	0.004456	0.012993
MF	GO:0016538	cyclin-dependent protein serine/threonine kinase regulator ac	46	-0.54762	-1.71155	0.004473	0.004473	0.013033
BP	GO:0000184	nuclear-transcribed mRNA catabolic process, nonsense-media	115	-0.41091	-1.50631	0.004522	0.004522	0.013155
BP	GO:0002062	chondrocyte differentiation	115	-0.41267	-1.51275	0.004522	0.004522	0.013155
BP	GO:0060037	pharyngeal system development	24	-0.62929	-1.70079	0.004529	0.004529	0.013166
BP	GO:0000083	regulation of transcription involved in G1/S transition of mitoti	26	-0.61745	-1.699	0.004582	0.004582	0.013295
BP	GO:0070126	mitochondrial translational termination	87	0.475405	1.616057	0.004583	0.004583	0.013295
BP	GO:0031145	anaphase-promoting complex-dependent catabolic process	79	-0.45465	-1.57643	0.004587	0.004587	0.013295
MF	GO:0043178	alcohol binding	85	0.480843	1.629706	0.004588	0.004588	0.013295
BP	GO:2000573	positive regulation of DNA biosynthetic process	65	-0.48229	-1.60709	0.004594	0.004594	0.013304
BP	GO:1904035	regulation of epithelial cell apoptotic process	71	0.49864	1.641597	0.004602	0.004602	0.013304

BP	GO:0001707	mesoderm formation	67	-0.47911	-1.60637	0.004602	0.004602	0.013304
MF	GO:0030247	polysaccharide binding	24	0.660766	1.74153	0.004678	0.004678	0.013513
BP	GO:0048009	insulin-like growth factor receptor signaling pathway	34	0.595625	1.706971	0.004734	0.004734	0.013666
CC	GO:0005796	Golgi lumen	95	0.462639	1.596632	0.004753	0.004753	0.01371
MF	GO:0004553	hydrolase activity, hydrolyzing O-glycosyl compounds	82	0.479415	1.613865	0.004795	0.004795	0.01382
MF	GO:0003684	damaged DNA binding	61	-0.49157	-1.61875	0.004809	0.004809	0.013848
BP	GO:2000826	regulation of heart morphogenesis	38	-0.55868	-1.67619	0.00482	0.00482	0.013869
BP	GO:0048754	branching morphogenesis of an epithelial tube	150	-0.38408	-1.46153	0.004832	0.004832	0.013894
BP	GO:1902807	negative regulation of cell cycle G1/S phase transition	99	-0.42241	-1.51659	0.004843	0.004843	0.013914
MF	GO:0060589	nucleoside-triphosphatase regulator activity	322	-0.32526	-1.359	0.004855	0.004855	0.01394
BP	GO:0002065	columnar/cuboidal epithelial cell differentiation	104	-0.41272	-1.49263	0.004862	0.004862	0.013949
BP	GO:0071621	granulocyte chemotaxis	109	0.442405	1.561445	0.004903	0.004903	0.014054
BP	GO:0019883	antigen processing and presentation of endogenous antigen	22	0.669077	1.734562	0.004922	0.004922	0.014098
BP	GO:0006977	DNA damage response, signal transduction by p53 class media	52	-0.52126	-1.66667	0.00494	0.00494	0.014138
BP	GO:0061448	connective tissue development	256	-0.33776	-1.37704	0.004969	0.004969	0.01421
BP	GO:0050716	positive regulation of interleukin-1 secretion	36	0.585794	1.693259	0.00498	0.00498	0.014232
BP	GO:0072171	mesonephric tubule morphogenesis	66	-0.47858	-1.59877	0.005004	0.005004	0.014289
BP	GO:0033238	regulation of cellular amine metabolic process	79	0.479379	1.60284	0.005012	0.005012	0.014301
BP	GO:1902414	protein localization to cell junction	87	-0.43961	-1.54728	0.005017	0.005017	0.014304
MF	GO:0005154	epidermal growth factor receptor binding	32	-0.58435	-1.68638	0.005021	0.005021	0.014305
BP	GO:2000134	negative regulation of G1/S transition of mitotic cell cycle	96	-0.4302	-1.53828	0.00504	0.00504	0.014349
CC	GO:0043198	dendritic shaft	33	-0.58055	-1.68456	0.005049	0.005049	0.014361
BP	GO:0048167	regulation of synaptic plasticity	180	-0.36968	-1.44235	0.005057	0.005057	0.014374
MF	GO:0016675	oxidoreductase activity, acting on a heme group of donors	24	0.655214	1.726896	0.005103	0.005103	0.014494
BP	GO:0060326	cell chemotaxis	271	0.357818	1.417453	0.005162	0.005162	0.014651
BP	GO:0042733	embryonic digit morphogenesis	56	-0.49818	-1.61469	0.005173	0.005173	0.014671
MF	GO:0035064	methylated histone binding	57	-0.49406	-1.60622	0.005198	0.005198	0.014718
MF	GO:0140034	methylation-dependent protein binding	57	-0.49406	-1.60622	0.005198	0.005198	0.014718
CC	GO:0099699	integral component of synaptic membrane	149	-0.38098	-1.44975	0.005221	0.005221	0.014774
BP	GO:0031290	retinal ganglion cell axon guidance	21	-0.65075	-1.70482	0.005251	0.005251	0.014838
BP	GO:0051054	positive regulation of DNA metabolic process	170	-0.37271	-1.44145	0.005252	0.005252	0.014838
BP	GO:0048701	embryonic cranial skeleton morphogenesis	45	-0.53882	-1.67495	0.005258	0.005258	0.014843
BP	GO:0031297	replication fork processing	23	-0.63247	-1.69276	0.00527	0.00527	0.014866
BP	GO:0061512	protein localization to cilium	47	-0.52827	-1.65548	0.00528	0.00528	0.014882
BP	GO:0007283	spermatogenesis	461	-0.29744	-1.28569	0.005302	0.005302	0.014933
BP	GO:0006144	purine nucleobase metabolic process	20	0.684706	1.743379	0.005335	0.005335	0.015015
BP	GO:0003231	cardiac ventricle development	124	-0.40034	-1.48539	0.00535	0.00535	0.015044
MF	GO:0000062	fatty-acyl-CoA binding	21	0.676405	1.735797	0.005353	0.005353	0.015044
BP	GO:0001649	osteoblast differentiation	199	-0.35781	-1.41308	0.005359	0.005359	0.015049

BP	GO:0030593	neutrophil chemotaxis	92	0.465763	1.596599	0.005369	0.005369	0.015054
BP	GO:1901186	positive regulation of ERBB signaling pathway	30	-0.60109	-1.71099	0.005369	0.005369	0.015054
BP	GO:0071356	cellular response to tumor necrosis factor	263	0.360212	1.421006	0.005379	0.005379	0.015071
BP	GO:0051781	positive regulation of cell division	83	-0.44533	-1.55818	0.005384	0.005384	0.015072
BP	GO:0009268	response to pH	36	0.579845	1.676061	0.005395	0.005395	0.015072
BP	GO:0106106	cold-induced thermogenesis	141	0.409661	1.504205	0.005396	0.005396	0.015072
BP	GO:0120161	regulation of cold-induced thermogenesis	141	0.409661	1.504205	0.005396	0.005396	0.015072
BP	GO:1904036	negative regulation of epithelial cell apoptotic process	38	0.574342	1.679877	0.0054	0.0054	0.015072
BP	GO:0042254	ribosome biogenesis	262	-0.33588	-1.37302	0.005421	0.005421	0.015119
BP	GO:0044275	cellular carbohydrate catabolic process	42	0.563895	1.683893	0.005562	0.005562	0.015503
BP	GO:0060416	response to growth hormone	38	0.57244	1.674315	0.005607	0.005607	0.015616
MF	GO:0005096	GTPase activator activity	253	-0.33826	-1.37633	0.00563	0.00563	0.015668
MF	GO:0003823	antigen binding	52	0.529412	1.648454	0.005667	0.005667	0.015758
BP	GO:1905515	non-motile cilium assembly	48	-0.52257	-1.64413	0.005683	0.005683	0.015791
BP	GO:0048259	regulation of receptor-mediated endocytosis	95	0.455328	1.571401	0.005744	0.005744	0.015948
BP	GO:0048593	camera-type eye morphogenesis	115	-0.40244	-1.47526	0.005755	0.005755	0.015969
BP	GO:0009260	ribonucleotide biosynthetic process	145	0.404034	1.490961	0.005764	0.005764	0.01598
BP	GO:0001837	epithelial to mesenchymal transition	129	-0.39125	-1.46053	0.005782	0.005782	0.016017
BP	GO:0032365	intracellular lipid transport	39	0.565444	1.663812	0.005798	0.005798	0.016027
BP	GO:0050771	negative regulation of axonogenesis	67	-0.47273	-1.585	0.005802	0.005802	0.016027
BP	GO:0003407	neural retina development	67	-0.47483	-1.59203	0.005802	0.005802	0.016027
BP	GO:0031571	mitotic G1 DNA damage checkpoint	59	-0.49	-1.60528	0.005808	0.005808	0.016027
BP	GO:0044819	mitotic G1/S transition checkpoint	59	-0.49	-1.60528	0.005808	0.005808	0.016027
BP	GO:0006692	prostanoid metabolic process	38	0.570483	1.66859	0.005815	0.005815	0.016027
BP	GO:0006693	prostaglandin metabolic process	38	0.570483	1.66859	0.005815	0.005815	0.016027
CC	GO:0000307	cyclin-dependent protein kinase holoenzyme complex	42	-0.54148	-1.65903	0.005828	0.005828	0.016049
BP	GO:0008038	neuron recognition	47	-0.52412	-1.64248	0.005866	0.005866	0.016144
CC	GO:0034707	chloride channel complex	50	-0.51976	-1.64708	0.005924	0.005924	0.016291
BP	GO:0006890	retrograde vesicle-mediated transport, Golgi to endoplasmic r	84	-0.44135	-1.54657	0.005998	0.005998	0.016481
BP	GO:0032206	positive regulation of telomere maintenance	46	-0.52944	-1.65475	0.006029	0.006029	0.016554
BP	GO:0048863	stem cell differentiation	249	-0.33906	-1.37764	0.006048	0.006048	0.016593
BP	GO:0021987	cerebral cortex development	106	-0.4097	-1.48516	0.006114	0.006114	0.016763
BP	GO:0045807	positive regulation of endocytosis	96	0.450628	1.556808	0.006148	0.006148	0.016845
BP	GO:0006813	potassium ion transport	223	-0.34906	-1.39913	0.006219	0.006219	0.017027
BP	GO:0032890	regulation of organic acid transport	55	0.516561	1.624107	0.006241	0.006241	0.017061
BP	GO:0003151	outflow tract morphogenesis	77	-0.44736	-1.54082	0.006246	0.006246	0.017061
BP	GO:0021675	nerve development	77	-0.45212	-1.55721	0.006246	0.006246	0.017061
BP	GO:0060324	face development	47	-0.52152	-1.63434	0.006257	0.006257	0.017061
BP	GO:0001823	mesonephros development	102	-0.41161	-1.48414	0.006258	0.006258	0.017061

CC	GO:0000314	organellar small ribosomal subunit	28	0.619096	1.694269	0.006267	0.006267	0.017061
CC	GO:0005763	mitochondrial small ribosomal subunit	28	0.619096	1.694269	0.006267	0.006267	0.017061
BP	GO:0032543	mitochondrial translation	120	0.427487	1.529507	0.006268	0.006268	0.017061
BP	GO:0046605	regulation of centrosome cycle	52	-0.50948	-1.62902	0.006323	0.006323	0.017197
BP	GO:0015837	amine transport	98	0.457612	1.585246	0.006348	0.006348	0.017252
BP	GO:0099601	regulation of neurotransmitter receptor activity	72	-0.46049	-1.56549	0.006382	0.006382	0.017332
BP	GO:0051169	nuclear transport	321	-0.32178	-1.34382	0.006402	0.006402	0.017349
MF	GO:0001217	DNA-binding transcription repressor activity	235	-0.34158	-1.38032	0.006402	0.006402	0.017349
MF	GO:0001227	DNA-binding transcription repressor activity, RNA polymerase	235	-0.34158	-1.38032	0.006402	0.006402	0.017349
CC	GO:0044322	endoplasmic reticulum quality control compartment	22	0.663889	1.721114	0.00642	0.00642	0.017373
BP	GO:0043299	leukocyte degranulation	509	0.313592	1.316664	0.00642	0.00642	0.017373
BP	GO:0042558	pteridine-containing compound metabolic process	32	0.591931	1.670441	0.006426	0.006426	0.017376
BP	GO:0002474	antigen processing and presentation of peptide antigen via MH	96	0.448665	1.550027	0.006545	0.006545	0.017684
BP	GO:0001756	somitogenesis	68	-0.46582	-1.56559	0.006595	0.006595	0.017786
BP	GO:1990542	mitochondrial transmembrane transport	60	0.505251	1.61345	0.006599	0.006599	0.017786
CC	GO:0048786	presynaptic active zone	66	-0.46922	-1.5675	0.006605	0.006605	0.017786
BP	GO:0098840	protein transport along microtubule	64	-0.47377	-1.5745	0.006607	0.006607	0.017786
BP	GO:0099118	microtubule-based protein transport	64	-0.47377	-1.5745	0.006607	0.006607	0.017786
BP	GO:0006346	DNA methylation-dependent heterochromatin assembly	21	-0.64044	-1.67779	0.006752	0.006752	0.018164
BP	GO:0045995	regulation of embryonic development	116	-0.39767	-1.45937	0.006778	0.006778	0.01822
BP	GO:0060675	ureteric bud morphogenesis	65	-0.47421	-1.58016	0.006792	0.006792	0.018226
CC	GO:0090543	Flemming body	24	-0.61765	-1.66933	0.006794	0.006794	0.018226
BP	GO:0072028	nephron morphogenesis	78	-0.44708	-1.5469	0.006797	0.006797	0.018226
MF	GO:0031210	phosphatidylcholine binding	24	0.641053	1.689573	0.006804	0.006804	0.018226
MF	GO:0050997	quaternary ammonium group binding	24	0.641053	1.689573	0.006804	0.006804	0.018226
BP	GO:0048864	stem cell development	85	-0.43531	-1.52864	0.006815	0.006815	0.018241
BP	GO:0042744	hydrogen peroxide catabolic process	32	0.59021	1.665584	0.006841	0.006841	0.018293
BP	GO:1902017	regulation of cilium assembly	47	-0.5188	-1.62582	0.006844	0.006844	0.018293
BP	GO:0006401	RNA catabolic process	364	-0.30674	-1.2982	0.006903	0.006903	0.018436
MF	GO:0019894	kinesin binding	38	-0.5487	-1.64624	0.00694	0.00694	0.018521
BP	GO:1902806	regulation of cell cycle G1/S phase transition	152	-0.3759	-1.43307	0.006944	0.006944	0.018521
MF	GO:0016836	hydro-lyase activity	43	0.548463	1.647314	0.006957	0.006957	0.018542
MF	GO:0008134	transcription factor binding	637	-0.28128	-1.24763	0.006983	0.006983	0.018597
MF	GO:0005543	phospholipid binding	397	0.327326	1.344305	0.006997	0.006997	0.018599
BP	GO:0001755	neural crest cell migration	57	-0.48213	-1.56743	0.006997	0.006997	0.018599
BP	GO:0044783	G1 DNA damage checkpoint	60	-0.48359	-1.58806	0.006999	0.006999	0.018599
MF	GO:0051020	GTPase binding	494	-0.29091	-1.26311	0.007009	0.007009	0.018614
BP	GO:0007178	transmembrane receptor protein serine/threonine kinase signa	320	-0.31556	-1.31734	0.007059	0.007059	0.018734
BP	GO:0017015	regulation of transforming growth factor beta receptor signalir	107	-0.40527	-1.47039	0.00713	0.00713	0.018907

BP	GO:0090277	positive regulation of peptide hormone secretion	90	0.457465	1.562439	0.007177	0.007177	0.019019
BP	GO:0072529	pyrimidine-containing compound catabolic process	37	0.565706	1.645177	0.007237	0.007237	0.019165
MF	GO:0019901	protein kinase binding	618	-0.28329	-1.25325	0.00725	0.00725	0.019181
BP	GO:0042311	vasodilation	29	0.609066	1.680799	0.007254	0.007254	0.019181
BP	GO:0006913	nucleocytoplasmic transport	318	-0.31812	-1.3277	0.007283	0.007283	0.019245
BP	GO:0045596	negative regulation of cell differentiation	650	-0.28113	-1.24737	0.007315	0.007315	0.019316
BP	GO:0048332	mesoderm morphogenesis	69	-0.4621	-1.55809	0.007379	0.007379	0.019472
BP	GO:0035418	protein localization to synapse	78	-0.44291	-1.53244	0.007397	0.007397	0.019505
BP	GO:0042359	vitamin D metabolic process	22	0.655161	1.698487	0.00749	0.00749	0.019735
BP	GO:0070372	regulation of ERK1 and ERK2 cascade	266	0.352669	1.392443	0.007602	0.007602	0.020018
BP	GO:0006493	protein O-linked glycosylation	87	-0.42742	-1.50436	0.007626	0.007626	0.020065
BP	GO:1990266	neutrophil migration	106	0.438175	1.539376	0.007655	0.007655	0.020126
BP	GO:0045907	positive regulation of vasoconstriction	31	0.592451	1.656181	0.0077	0.0077	0.020232
CC	GO:0035580	specific granule lumen	60	0.501489	1.601434	0.007798	0.007798	0.020476
MF	GO:0005549	odorant binding	86	-0.42736	-1.50179	0.007813	0.007813	0.020498
BP	GO:0090263	positive regulation of canonical Wnt signaling pathway	137	-0.38145	-1.43821	0.007842	0.007842	0.02056
BP	GO:0016358	dendrite development	222	-0.34281	-1.37363	0.007925	0.007925	0.020763
BP	GO:0090183	regulation of kidney development	55	-0.48728	-1.57547	0.007944	0.007944	0.020781
MF	GO:0031491	nucleosome binding	53	-0.49278	-1.57862	0.007944	0.007944	0.020781
MF	GO:1901567	fatty acid derivative binding	27	0.61782	1.67658	0.007948	0.007948	0.020781
BP	GO:0014032	neural crest cell development	81	-0.43543	-1.51777	0.007957	0.007957	0.020789
BP	GO:0071897	DNA biosynthetic process	176	-0.36535	-1.42104	0.007993	0.007993	0.020869
BP	GO:0035082	axoneme assembly	43	-0.52633	-1.61961	0.008016	0.008016	0.020913
BP	GO:0097755	positive regulation of blood vessel diameter	55	0.509445	1.601736	0.008053	0.008053	0.020996
MF	GO:0005254	chloride channel activity	74	-0.44799	-1.534	0.008146	0.008146	0.021224
BP	GO:0046390	ribose phosphate biosynthetic process	152	0.391099	1.450919	0.00819	0.00819	0.021324
BP	GO:0002819	regulation of adaptive immune response	154	0.39162	1.454394	0.008211	0.008211	0.021362
BP	GO:0072088	nephron epithelium morphogenesis	76	-0.43933	-1.50979	0.00823	0.00823	0.021396
BP	GO:0051193	regulation of cofactor metabolic process	35	0.57241	1.650706	0.008261	0.008261	0.021463
MF	GO:0050839	cell adhesion molecule binding	456	-0.29449	-1.27191	0.008276	0.008276	0.021487
CC	GO:0045171	intercellular bridge	55	-0.48528	-1.56902	0.008342	0.008342	0.021618
BP	GO:0010769	regulation of cell morphogenesis involved in differentiation	286	-0.32277	-1.33285	0.008342	0.008342	0.021618
BP	GO:0001764	neuron migration	147	-0.37361	-1.41963	0.008344	0.008344	0.021618
BP	GO:0061387	regulation of extent of cell growth	106	-0.40419	-1.46519	0.008355	0.008355	0.021633
BP	GO:0099173	postsynapse organization	154	-0.37052	-1.41508	0.008395	0.008395	0.021719
CC	GO:0005643	nuclear pore	82	-0.43374	-1.51346	0.008405	0.008405	0.021731
BP	GO:0061053	somite development	86	-0.42589	-1.49663	0.008413	0.008413	0.021738
BP	GO:0014033	neural crest cell differentiation	90	-0.42207	-1.49357	0.008424	0.008424	0.021749
BP	GO:0006140	regulation of nucleotide metabolic process	107	0.431774	1.519196	0.008443	0.008443	0.021784

BP	GO:0003143	embryonic heart tube morphogenesis	66	-0.4617	-1.54236	0.008607	0.008607	0.022191
BP	GO:0014031	mesenchymal cell development	85	-0.42895	-1.50632	0.008619	0.008619	0.022191
CC	GO:0005684	U2-type spliceosomal complex	85	-0.4296	-1.50859	0.008619	0.008619	0.022191
BP	GO:1902115	regulation of organelle assembly	177	-0.36412	-1.41612	0.00865	0.00865	0.022255
BP	GO:0003179	heart valve morphogenesis	50	-0.50035	-1.58557	0.008689	0.008689	0.02234
BP	GO:0022604	regulation of cell morphogenesis	456	-0.29417	-1.27051	0.008736	0.008736	0.022445
MF	GO:0042379	chemokine receptor binding	60	0.498086	1.590568	0.008798	0.008798	0.02259
BP	GO:1990138	neuron projection extension	164	-0.3638	-1.40158	0.008811	0.008811	0.022606
BP	GO:0000302	response to reactive oxygen species	211	0.366069	1.412024	0.008828	0.008828	0.022635
BP	GO:0051954	positive regulation of amine transport	34	0.57383	1.644508	0.008851	0.008851	0.02268
BP	GO:0034114	regulation of heterotypic cell-cell adhesion	25	0.628422	1.675145	0.008865	0.008865	0.022682
BP	GO:0018210	peptidyl-threonine modification	125	-0.38609	-1.43283	0.008866	0.008866	0.022682
BP	GO:0045840	positive regulation of mitotic nuclear division	49	-0.50342	-1.58871	0.00887	0.00887	0.022682
BP	GO:0051047	positive regulation of secretion	413	0.321229	1.325135	0.008937	0.008937	0.022835
BP	GO:0097530	granulocyte migration	127	0.411492	1.486257	0.008981	0.008981	0.022933
BP	GO:0060291	long-term synaptic potentiation	84	-0.42728	-1.49727	0.008996	0.008996	0.02295
BP	GO:0034637	cellular carbohydrate biosynthetic process	84	0.458103	1.548358	0.009	0.009	0.02295
BP	GO:0016573	histone acetylation	138	-0.3788	-1.4295	0.009068	0.009068	0.023105
BP	GO:0048732	gland development	423	0.31978	1.321866	0.009073	0.009073	0.023105
BP	GO:1904358	positive regulation of telomere maintenance via telomere leng	37	-0.54315	-1.62201	0.009098	0.009098	0.023152
CC	GO:0031253	cell projection membrane	309	-0.31486	-1.30939	0.009105	0.009105	0.023154
BP	GO:1903825	organic acid transmembrane transport	131	0.404551	1.468208	0.009117	0.009117	0.023154
BP	GO:1905039	carboxylic acid transmembrane transport	131	0.404551	1.468208	0.009117	0.009117	0.023154
BP	GO:0002683	negative regulation of immune system process	387	0.323294	1.325303	0.009132	0.009132	0.023174
MF	GO:0042605	peptide antigen binding	23	0.642629	1.679109	0.009384	0.009384	0.023798
BP	GO:0006855	drug transmembrane transport	75	0.464499	1.541686	0.009398	0.009398	0.023818
MF	GO:0008009	chemokine activity	47	0.528738	1.615707	0.009615	0.009615	0.024352
BP	GO:0071526	semaphorin-plexin signaling pathway	40	-0.53348	-1.61845	0.009653	0.009653	0.024425
MF	GO:0042165	neurotransmitter binding	54	0.506439	1.587707	0.009658	0.009658	0.024425
BP	GO:0008361	regulation of cell size	170	-0.36288	-1.40342	0.009664	0.009664	0.024425
BP	GO:0006213	pyrimidine nucleoside metabolic process	33	0.571417	1.626635	0.009687	0.009687	0.024466
BP	GO:0006096	glycolytic process	106	0.427303	1.501182	0.009814	0.009814	0.024753
BP	GO:0006757	ATP generation from ADP	106	0.427303	1.501182	0.009814	0.009814	0.024753
BP	GO:0097120	receptor localization to synapse	52	-0.4871	-1.55744	0.009879	0.009879	0.024879
BP	GO:0034383	low-density lipoprotein particle clearance	34	0.570329	1.634476	0.009881	0.009881	0.024879
BP	GO:0097064	ncRNA export from nucleus	35	-0.54746	-1.61363	0.009884	0.009884	0.024879
MF	GO:0140030	modification-dependent protein binding	125	-0.38406	-1.42528	0.009897	0.009897	0.024896
CC	GO:0031252	cell leading edge	380	-0.30305	-1.28733	0.009908	0.009908	0.024906
BP	GO:0051607	defense response to virus	220	0.361061	1.399746	0.009934	0.009934	0.024956

BP	GO:0072091	regulation of stem cell proliferation	62	-0.46229	-1.52831	0.00998	0.00998	0.025054
BP	GO:0014706	striated muscle tissue development	348	-0.30525	-1.28607	0.009987	0.009987	0.025054
MF	GO:0004129	cytochrome-c oxidase activity	23	0.639082	1.669841	0.010023	0.010023	0.025095
MF	GO:0015002	heme-copper terminal oxidase activity	23	0.639082	1.669841	0.010023	0.010023	0.025095
MF	GO:0016676	oxidoreductase activity, acting on a heme group of donors, ox	23	0.639082	1.669841	0.010023	0.010023	0.025095
CC	GO:0030426	growth cone	162	-0.36463	-1.40286	0.010078	0.010078	0.025214
BP	GO:1990845	adaptive thermogenesis	152	0.387459	1.437414	0.010095	0.010095	0.025241
BP	GO:1905330	regulation of morphogenesis of an epithelium	177	-0.35973	-1.39905	0.010127	0.010127	0.025302
BP	GO:0006479	protein methylation	139	-0.3764	-1.42044	0.01017	0.01017	0.025372
BP	GO:0008213	protein alkylation	139	-0.3764	-1.42044	0.01017	0.01017	0.025372
MF	GO:0051536	iron-sulfur cluster binding	59	0.497633	1.583522	0.010182	0.010182	0.025372
MF	GO:0051540	metal cluster binding	59	0.497633	1.583522	0.010182	0.010182	0.025372
BP	GO:0016925	protein sumoylation	77	-0.43382	-1.49418	0.010276	0.010276	0.02559
BP	GO:0032212	positive regulation of telomere maintenance via telomerase	34	-0.55127	-1.61147	0.010303	0.010303	0.02564
BP	GO:0009895	negative regulation of catabolic process	274	0.34399	1.36339	0.010324	0.010324	0.025676
BP	GO:0045738	negative regulation of DNA repair	23	-0.6105	-1.63394	0.010352	0.010352	0.025727
BP	GO:0003279	cardiac septum development	106	-0.39746	-1.4408	0.010393	0.010393	0.025812
CC	GO:0098576	lumenal side of membrane	31	0.58255	1.628504	0.010406	0.010406	0.02582
BP	GO:2001021	negative regulation of response to DNA damage stimulus	64	-0.45663	-1.51754	0.01041	0.01041	0.02582
BP	GO:0010827	regulation of glucose transmembrane transport	69	0.475159	1.556392	0.010425	0.010425	0.025839
BP	GO:0019229	regulation of vasoconstriction	56	0.500768	1.578852	0.01045	0.01045	0.025884
BP	GO:0018107	peptidyl-threonine phosphorylation	118	-0.38392	-1.4126	0.01047	0.01047	0.025917
BP	GO:0046365	monosaccharide catabolic process	62	0.49007	1.57371	0.010617	0.010617	0.026263
BP	GO:0097435	supramolecular fiber organization	617	-0.27827	-1.23095	0.010638	0.010638	0.026298
BP	GO:0051031	tRNA transport	33	-0.55814	-1.61955	0.01068	0.01068	0.026383
BP	GO:1901136	carbohydrate derivative catabolic process	182	0.374113	1.415818	0.010705	0.010705	0.026429
BP	GO:0060384	innervation	27	-0.58778	-1.62893	0.010726	0.010726	0.026462
BP	GO:2000045	regulation of G1/S transition of mitotic cell cycle	140	-0.37509	-1.41794	0.010764	0.010764	0.026538
BP	GO:2000027	regulation of animal organ morphogenesis	249	-0.33162	-1.3474	0.010799	0.010799	0.026607
BP	GO:1902750	negative regulation of cell cycle G2/M phase transition	92	-0.41545	-1.4756	0.010859	0.010859	0.026736
BP	GO:0002263	cell activation involved in immune response	678	0.292646	1.255115	0.010935	0.010935	0.026907
CC	GO:0000803	sex chromosome	24	-0.60349	-1.63107	0.010945	0.010945	0.026914
BP	GO:0061333	renal tubule morphogenesis	78	-0.43272	-1.49719	0.010996	0.010996	0.027019
BP	GO:0016578	histone deubiquitination	22	-0.6189	-1.64056	0.011071	0.011071	0.02717
BP	GO:1902275	regulation of chromatin organization	161	-0.36137	-1.39047	0.011072	0.011072	0.02717
BP	GO:0051101	regulation of DNA binding	117	-0.38675	-1.42032	0.011116	0.011116	0.027261
BP	GO:0043414	macromolecule methylation	236	-0.33321	-1.34625	0.011147	0.011147	0.027319
MF	GO:0048018	receptor ligand activity	448	0.311616	1.293395	0.011186	0.011186	0.027397
BP	GO:0002366	leukocyte activation involved in immune response	675	0.292084	1.252031	0.011314	0.011314	0.027691

BP	GO:0003205	cardiac chamber development	163	-0.36113	-1.39053	0.011326	0.011326	0.027702
BP	GO:0050766	positive regulation of phagocytosis	66	0.475561	1.547041	0.011386	0.011386	0.027832
BP	GO:0002548	monocyte chemotaxis	62	0.487376	1.56506	0.011418	0.011418	0.027877
BP	GO:0060537	muscle tissue development	363	-0.30248	-1.27927	0.01142	0.01142	0.027877
CC	GO:0030427	site of polarized growth	167	-0.35972	-1.38881	0.011494	0.011494	0.028041
CC	GO:0043197	dendritic spine	148	-0.36755	-1.39712	0.011504	0.011504	0.028046
BP	GO:0009110	vitamin biosynthetic process	23	0.634323	1.657406	0.011516	0.011516	0.028058
MF	GO:0019904	protein domain specific binding	673	-0.27498	-1.22294	0.011556	0.011556	0.028137
BP	GO:0002011	morphogenesis of an epithelial sheet	49	-0.49286	-1.55537	0.01163	0.01163	0.028298
CC	GO:0042734	presynaptic membrane	158	-0.36154	-1.38722	0.011689	0.011689	0.028422
MF	GO:0005245	voltage-gated calcium channel activity	47	-0.49997	-1.56681	0.011732	0.011732	0.02851
BP	GO:0034113	heterotypic cell-cell adhesion	60	0.491769	1.570395	0.011798	0.011798	0.02865
BP	GO:1903311	regulation of mRNA metabolic process	296	-0.3138	-1.30085	0.011821	0.011821	0.028686
BP	GO:0055069	zinc ion homeostasis	36	0.555162	1.604716	0.011828	0.011828	0.028686
BP	GO:1903579	negative regulation of ATP metabolic process	24	0.623398	1.643043	0.011907	0.011907	0.028859
BP	GO:0051785	positive regulation of nuclear division	62	-0.45789	-1.51378	0.011976	0.011976	0.029007
BP	GO:0090276	regulation of peptide hormone secretion	197	0.363644	1.393052	0.012005	0.012005	0.029059
BP	GO:0070542	response to fatty acid	83	0.448914	1.51306	0.012031	0.012031	0.029102
BP	GO:0048536	spleen development	33	-0.55069	-1.59791	0.012039	0.012039	0.029102
BP	GO:0030278	regulation of ossification	179	-0.35425	-1.38009	0.012071	0.012071	0.029161
BP	GO:0019751	polyol metabolic process	121	0.411492	1.474835	0.012112	0.012112	0.029234
BP	GO:0070371	ERK1 and ERK2 cascade	283	0.339264	1.349761	0.012117	0.012117	0.029234
CC	GO:0043596	nuclear replication fork	39	-0.5275	-1.59132	0.012179	0.012179	0.029353
MF	GO:0030546	signaling receptor activator activity	453	0.310316	1.290764	0.012182	0.012182	0.029353
BP	GO:0035282	segmentation	97	-0.40235	-1.44155	0.012259	0.012259	0.029519
CC	GO:0031248	protein acetyltransferase complex	77	-0.4295	-1.47931	0.012492	0.012492	0.030035
CC	GO:1902493	acetyltransferase complex	77	-0.4295	-1.47931	0.012492	0.012492	0.030035
BP	GO:0006997	nucleus organization	119	-0.38356	-1.41337	0.012497	0.012497	0.030035
BP	GO:0048168	regulation of neuronal synaptic plasticity	48	-0.49283	-1.55056	0.012542	0.012542	0.030122
MF	GO:0017056	structural constituent of nuclear pore	24	-0.59821	-1.61681	0.012644	0.012644	0.030348
BP	GO:0009132	nucleoside diphosphate metabolic process	143	0.389901	1.436201	0.012685	0.012685	0.030427
BP	GO:0022898	regulation of transmembrane transporter activity	242	-0.32862	-1.3315	0.012694	0.012694	0.030428
BP	GO:0045604	regulation of epidermal cell differentiation	53	-0.47789	-1.53091	0.01291	0.01291	0.030918
BP	GO:0072078	nephron tubule morphogenesis	74	-0.4325	-1.48097	0.012915	0.012915	0.030918
BP	GO:0010717	regulation of epithelial to mesenchymal transition	79	-0.42547	-1.47527	0.012964	0.012964	0.031012
BP	GO:0006650	glycerophospholipid metabolic process	302	0.333483	1.335045	0.012978	0.012978	0.031012
MF	GO:0048029	monosaccharide binding	70	0.465105	1.527327	0.012979	0.012979	0.031012
CC	GO:0044309	neuron spine	150	-0.36495	-1.3887	0.013025	0.013025	0.031074
BP	GO:0061647	histone H3-K9 modification	46	-0.49977	-1.56201	0.01303	0.01303	0.031074

MF	GO:0005310	dicarboxylic acid transmembrane transporter activity	39	0.538008	1.583084	0.013046	0.013046	0.031074
BP	GO:0048477	oogenesis	76	-0.43014	-1.4782	0.013047	0.013047	0.031074
BP	GO:1903827	regulation of cellular protein localization	491	-0.28508	-1.23771	0.013054	0.013054	0.031074
CC	GO:0034705	potassium channel complex	93	-0.40491	-1.44082	0.01306	0.01306	0.031074
BP	GO:0042398	cellular modified amino acid biosynthetic process	48	0.513374	1.5757	0.013064	0.013064	0.031074
BP	GO:0006835	dicarboxylic acid transport	95	0.432747	1.49347	0.013072	0.013072	0.031074
BP	GO:0035337	fatty-acyl-CoA metabolic process	38	0.542051	1.585429	0.013084	0.013084	0.031083
BP	GO:0043094	cellular metabolic compound salvage	31	0.574455	1.605875	0.013111	0.013111	0.031128
BP	GO:0090103	cochlea morphogenesis	23	-0.60163	-1.61019	0.013175	0.013175	0.03126
BP	GO:0032881	regulation of polysaccharide metabolic process	41	0.532823	1.58407	0.013223	0.013223	0.031331
BP	GO:0006515	protein quality control for misfolded or incompletely synthesiz	26	0.608597	1.638906	0.013224	0.013224	0.031331
BP	GO:0042073	intraciliary transport	50	-0.48744	-1.54465	0.013231	0.013231	0.031331
BP	GO:0006409	tRNA export from nucleus	31	-0.55818	-1.60103	0.013277	0.013277	0.031401
BP	GO:0071431	tRNA-containing ribonucleoprotein complex export from nucle	31	-0.55818	-1.60103	0.013277	0.013277	0.031401
BP	GO:0032652	regulation of interleukin-1 production	89	0.434963	1.484365	0.013344	0.013344	0.031539
BP	GO:0016233	telomere capping	35	-0.53639	-1.58099	0.013372	0.013372	0.031586
BP	GO:0045165	cell fate commitment	259	-0.3224	-1.31643	0.013414	0.013414	0.031665
BP	GO:0006882	cellular zinc ion homeostasis	35	0.557128	1.606638	0.013424	0.013424	0.031669
BP	GO:0048871	multicellular organismal homeostasis	465	0.308266	1.285509	0.013533	0.013533	0.031904
BP	GO:0010458	exit from mitosis	30	-0.56274	-1.60184	0.013615	0.013615	0.032067
CC	GO:0005741	mitochondrial outer membrane	158	0.38021	1.41486	0.013625	0.013625	0.032067
CC	GO:0005775	vacuolar lumen	157	0.379356	1.409993	0.013628	0.013628	0.032067
MF	GO:0005253	anion channel activity	87	-0.41279	-1.45289	0.013646	0.013646	0.032091
BP	GO:0001990	regulation of systemic arterial blood pressure by hormone	39	0.536612	1.578976	0.013667	0.013667	0.03212
BP	GO:1902667	regulation of axon guidance	43	-0.50666	-1.55907	0.013685	0.013685	0.032142
BP	GO:0031124	mRNA 3'-end processing	81	-0.41935	-1.46172	0.013726	0.013726	0.032217
BP	GO:0050772	positive regulation of axonogenesis	83	-0.41427	-1.44949	0.013759	0.013759	0.032273
CC	GO:1904949	ATPase complex	78	-0.42378	-1.46627	0.013794	0.013794	0.032337
BP	GO:0021846	cell proliferation in forebrain	28	-0.57609	-1.61193	0.013806	0.013806	0.032344
BP	GO:0035137	hindlimb morphogenesis	36	-0.53182	-1.57965	0.013892	0.013892	0.032524
BP	GO:0033014	tetrapyrrole biosynthetic process	27	0.601573	1.63249	0.014014	0.014014	0.032789
CC	GO:0070069	cytochrome complex	26	0.605319	1.630081	0.014064	0.014064	0.032858
BP	GO:0006760	folic acid-containing compound metabolic process	26	0.604776	1.628617	0.014064	0.014064	0.032858
BP	GO:0051168	nuclear export	175	-0.35269	-1.3707	0.01407	0.01407	0.032858
BP	GO:0043967	histone H4 acetylation	53	-0.47538	-1.52288	0.014101	0.014101	0.032907
MF	GO:0099095	ligand-gated anion channel activity	20	-0.626	-1.62046	0.014108	0.014108	0.032907
MF	GO:0042887	amide transmembrane transporter activity	43	0.52513	1.577234	0.014119	0.014119	0.032911
BP	GO:0007519	skeletal muscle tissue development	146	-0.36327	-1.37869	0.014199	0.014199	0.033077
CC	GO:0031968	organelle outer membrane	178	0.368516	1.393366	0.014264	0.014264	0.033207

BP	GO:0009914	hormone transport	301	0.331984	1.328402	0.014283	0.014283	0.033231
MF	GO:0019207	kinase regulator activity	187	-0.34423	-1.35069	0.014325	0.014325	0.033307
BP	GO:0006284	base-excision repair	41	-0.5124	-1.56019	0.014336	0.014336	0.033311
MF	GO:0015485	cholesterol binding	49	0.509333	1.571169	0.014405	0.014405	0.033451
BP	GO:0030595	leukocyte chemotaxis	202	0.360213	1.382381	0.014449	0.014449	0.033534
BP	GO:0071333	cellular response to glucose stimulus	120	0.40745	1.457814	0.014691	0.014691	0.034074
MF	GO:0042162	telomeric DNA binding	33	-0.54244	-1.57398	0.014757	0.014757	0.034206
BP	GO:0003158	endothelium development	116	0.409739	1.460208	0.014806	0.014806	0.034298
CC	GO:0017053	transcription repressor complex	76	-0.42687	-1.46698	0.014853	0.014853	0.034386
MF	GO:0008198	ferrous iron binding	23	0.628324	1.641733	0.014929	0.014929	0.034538
MF	GO:0003729	mRNA binding	236	-0.32925	-1.33025	0.015005	0.015005	0.034694
BP	GO:0001658	branching involved in ureteric bud morphogenesis	59	-0.45668	-1.49612	0.015021	0.015021	0.034709
BP	GO:0031099	regeneration	176	0.3683	1.390137	0.015053	0.015053	0.034762
BP	GO:0010742	macrophage derived foam cell differentiation	34	0.557965	1.599041	0.015233	0.015233	0.035132
BP	GO:0090077	foam cell differentiation	34	0.557965	1.599041	0.015233	0.015233	0.035132
BP	GO:0071900	regulation of protein serine/threonine kinase activity	481	-0.28439	-1.2328	0.015345	0.015345	0.03537
BP	GO:0006414	translational elongation	121	0.404029	1.448086	0.015433	0.015433	0.03555
MF	GO:0030020	extracellular matrix structural constituent conferring tensile str	40	-0.51215	-1.55374	0.015444	0.015444	0.035554
BP	GO:0051570	regulation of histone H3-K9 methylation	22	-0.60498	-1.60365	0.015763	0.015763	0.036256
BP	GO:0000726	non-recombinational repair	62	-0.45251	-1.49597	0.015768	0.015768	0.036256
BP	GO:0040020	regulation of meiotic nuclear division	31	-0.55206	-1.58347	0.015778	0.015778	0.036256
BP	GO:0035050	embryonic heart tube development	75	-0.42532	-1.45839	0.015797	0.015797	0.036276
BP	GO:0032355	response to estradiol	129	0.396405	1.435641	0.015895	0.015895	0.036478
MF	GO:0004620	phospholipase activity	94	0.429255	1.476977	0.015924	0.015924	0.036522
BP	GO:0060795	cell fate commitment involved in formation of primary germ la	34	-0.53438	-1.56209	0.015941	0.015941	0.036539
BP	GO:0048843	negative regulation of axon extension involved in axon guidanc	26	-0.57716	-1.58816	0.016037	0.016037	0.036736
MF	GO:0005343	organic acid:sodium symporter activity	28	0.587487	1.607763	0.016085	0.016085	0.036825
BP	GO:0007156	homophilic cell adhesion via plasma membrane adhesion mole	162	-0.35647	-1.37148	0.016166	0.016166	0.036987
BP	GO:0021602	cranial nerve morphogenesis	28	-0.56873	-1.59132	0.016299	0.016299	0.037268
CC	GO:0019867	outer membrane	180	0.365743	1.383486	0.016362	0.016362	0.037373
BP	GO:0010824	regulation of centrosome duplication	38	-0.52118	-1.56369	0.016387	0.016387	0.037373
BP	GO:0007492	endoderm development	75	-0.42497	-1.4572	0.016397	0.016397	0.037373
BP	GO:0015800	acidic amino acid transport	65	0.469389	1.521038	0.016413	0.016413	0.037373
BP	GO:0045992	negative regulation of embryonic development	26	-0.57559	-1.58382	0.016418	0.016418	0.037373
BP	GO:0006353	DNA-templated transcription, termination	70	-0.43127	-1.45752	0.01642	0.01642	0.037373
MF	GO:0019865	immunoglobulin binding	23	0.623688	1.629619	0.016421	0.016421	0.037373
BP	GO:0051445	regulation of meiotic cell cycle	47	-0.48635	-1.52412	0.016425	0.016425	0.037373
BP	GO:0071331	cellular response to hexose stimulus	122	0.401786	1.442261	0.016537	0.016537	0.037604
BP	GO:0044380	protein localization to cytoskeleton	49	-0.48143	-1.51929	0.016558	0.016558	0.037613

BP	GO:0001704	formation of primary germ layer	113	-0.38042	-1.39132	0.016561	0.016561	0.037613
BP	GO:0050821	protein stabilization	163	0.372237	1.39179	0.016622	0.016622	0.037712
BP	GO:0045197	establishment or maintenance of epithelial cell apical/basal po	39	-0.51453	-1.55219	0.016625	0.016625	0.037712
BP	GO:0071326	cellular response to monosaccharide stimulus	123	0.400195	1.439122	0.016745	0.016745	0.03796
BP	GO:0048546	digestive tract morphogenesis	49	-0.48086	-1.51752	0.016755	0.016755	0.037961
MF	GO:0034212	peptide N-acetyltransferase activity	62	-0.45019	-1.4883	0.016766	0.016766	0.037963
BP	GO:0031047	gene silencing by RNA	139	-0.365	-1.3774	0.016812	0.016812	0.038043
MF	GO:0030594	neurotransmitter receptor activity	115	-0.37909	-1.38967	0.016855	0.016855	0.038118
BP	GO:0032527	protein exit from endoplasmic reticulum	41	0.524189	1.558401	0.016942	0.016942	0.038291
BP	GO:0002822	regulation of adaptive immune response based on somatic rec	139	0.386045	1.413721	0.016975	0.016975	0.038343
BP	GO:0009135	purine nucleoside diphosphate metabolic process	125	0.398628	1.438205	0.017081	0.017081	0.038534
BP	GO:0009179	purine ribonucleoside diphosphate metabolic process	125	0.398628	1.438205	0.017081	0.017081	0.038534
BP	GO:0032412	regulation of ion transmembrane transporter activity	234	-0.32581	-1.31571	0.017134	0.017134	0.038632
CC	GO:0034708	methyltransferase complex	102	-0.39091	-1.4095	0.017158	0.017158	0.038661
BP	GO:0009185	ribonucleoside diphosphate metabolic process	127	0.39541	1.42817	0.017181	0.017181	0.038687
BP	GO:0072132	mesenchyme morphogenesis	52	-0.47162	-1.50796	0.01719	0.01719	0.038687
BP	GO:1900027	regulation of ruffle assembly	27	-0.57081	-1.5819	0.017238	0.017238	0.038772
BP	GO:1904666	regulation of ubiquitin protein ligase activity	22	-0.60178	-1.59517	0.017264	0.017264	0.038806
MF	GO:0031490	chromatin DNA binding	88	-0.40644	-1.43273	0.017325	0.017325	0.038919
BP	GO:0021543	pallium development	160	-0.35517	-1.36574	0.017379	0.017379	0.039017
MF	GO:0004713	protein tyrosine kinase activity	133	-0.36539	-1.37146	0.017497	0.017497	0.039248
BP	GO:0051972	regulation of telomerase activity	46	-0.48714	-1.52254	0.017503	0.017503	0.039248
MF	GO:0015399	primary active transmembrane transporter activity	108	0.408226	1.439159	0.017613	0.017613	0.03947
BP	GO:2000780	negative regulation of double-strand break repair	21	-0.60714	-1.59057	0.017629	0.017629	0.039485
CC	GO:0097546	ciliary base	28	-0.56373	-1.57735	0.017641	0.017641	0.039488
CC	GO:0016323	basolateral plasma membrane	211	0.351758	1.356822	0.017656	0.017656	0.039496
BP	GO:0002221	pattern recognition receptor signaling pathway	180	0.364117	1.377334	0.017694	0.017694	0.039558
BP	GO:0002063	chondrocyte development	45	-0.49102	-1.52636	0.017722	0.017722	0.039595
BP	GO:0006521	regulation of cellular amino acid metabolic process	61	0.476071	1.52549	0.017761	0.017761	0.03966
BP	GO:0071222	cellular response to lipopolysaccharide	186	0.361625	1.37376	0.017844	0.017844	0.03982
BP	GO:0009152	purine ribonucleotide biosynthetic process	133	0.39089	1.421065	0.017885	0.017885	0.039889
BP	GO:0060041	retina development in camera-type eye	144	-0.36265	-1.37436	0.017917	0.017917	0.039913
BP	GO:0001890	placenta development	145	-0.36115	-1.37013	0.017928	0.017928	0.039913
BP	GO:0007062	sister chromatid cohesion	49	-0.47761	-1.50726	0.017938	0.017938	0.039913
BP	GO:0030501	positive regulation of bone mineralization	36	-0.52287	-1.55307	0.017943	0.017943	0.039913
BP	GO:0110110	positive regulation of animal organ morphogenesis	79	-0.41382	-1.43488	0.01795	0.01795	0.039913
BP	GO:0010874	regulation of cholesterol efflux	22	0.62616	1.623303	0.017976	0.017976	0.039947
BP	GO:1901654	response to ketone	189	0.360513	1.372409	0.017989	0.017989	0.039953
BP	GO:0015872	dopamine transport	51	0.496216	1.54008	0.018045	0.018045	0.040049

BP	GO:0051056	regulation of small GTPase mediated signal transduction	323	-0.30161	-1.26067	0.018054	0.018054	0.040049
BP	GO:0051702	interaction with symbiont	74	0.450771	1.49099	0.018314	0.018314	0.040601
BP	GO:0000729	DNA double-strand break processing	21	-0.60535	-1.58589	0.01838	0.01838	0.040723
CC	GO:0005758	mitochondrial intermembrane space	65	0.466845	1.512794	0.018415	0.018415	0.040777
BP	GO:0014902	myotube differentiation	93	-0.39666	-1.41145	0.018485	0.018485	0.040908
CC	GO:0008021	synaptic vesicle	181	-0.34121	-1.33287	0.018694	0.018694	0.041345
MF	GO:0097472	cyclin-dependent protein kinase activity	29	-0.55757	-1.56873	0.018737	0.018737	0.041416
BP	GO:0046031	ADP metabolic process	113	0.405671	1.440583	0.018783	0.018783	0.041493
MF	GO:0098631	cell adhesion mediator activity	59	-0.45147	-1.47904	0.018826	0.018826	0.041564
MF	GO:0017124	SH3 domain binding	127	-0.36929	-1.37627	0.018852	0.018852	0.041597
BP	GO:0043467	regulation of generation of precursor metabolites and energy	140	0.382791	1.402714	0.018952	0.018952	0.041792
BP	GO:0009620	response to fungus	48	0.502669	1.542841	0.018983	0.018983	0.041837
BP	GO:0010638	positive regulation of organelle organization	566	-0.2756	-1.21119	0.019039	0.019039	0.041933
BP	GO:0006029	proteoglycan metabolic process	91	-0.39873	-1.41328	0.019072	0.019072	0.041983
BP	GO:0043586	tongue development	20	-0.61434	-1.59026	0.019187	0.019187	0.042211
BP	GO:0071467	cellular response to pH	21	0.627782	1.611019	0.019272	0.019272	0.042372
MF	GO:0008066	glutamate receptor activity	27	-0.56102	-1.55477	0.019345	0.019345	0.042507
BP	GO:0050427	3'-phosphoadenosine 5'-phosphosulfate metabolic process	23	0.61919	1.617865	0.019407	0.019407	0.042619
CC	GO:0005771	multivesicular body	45	0.510478	1.545716	0.019519	0.019519	0.042805
BP	GO:0031057	negative regulation of histone modification	39	-0.50866	-1.53449	0.019524	0.019524	0.042805
BP	GO:0003341	cilium movement	51	-0.4691	-1.49406	0.019527	0.019527	0.042805
BP	GO:1902668	negative regulation of axon guidance	28	-0.55923	-1.56475	0.019559	0.019559	0.042851
BP	GO:0060538	skeletal muscle organ development	153	-0.35397	-1.35027	0.019604	0.019604	0.042924
BP	GO:0051955	regulation of amino acid transport	31	0.561666	1.570122	0.019771	0.019771	0.043265
CC	GO:0035869	ciliary transition zone	58	-0.45402	-1.4815	0.019852	0.019852	0.043415
BP	GO:0048645	animal organ formation	63	-0.44028	-1.45748	0.019903	0.019903	0.043503
MF	GO:0016757	transferase activity, transferring glycosyl groups	254	0.335946	1.320746	0.019929	0.019929	0.043534
BP	GO:0008542	visual learning	47	-0.48045	-1.50564	0.019945	0.019945	0.043543
BP	GO:0034035	purine ribonucleoside bisphosphate metabolic process	24	0.606444	1.598358	0.019987	0.019987	0.043609
BP	GO:0090224	regulation of spindle organization	39	-0.50658	-1.52821	0.020104	0.020104	0.043839
BP	GO:0021537	telencephalon development	239	-0.32153	-1.30124	0.020193	0.020193	0.043983
BP	GO:0043902	positive regulation of multi-organism process	33	-0.52628	-1.52709	0.020194	0.020194	0.043983
BP	GO:0008299	isoprenoid biosynthetic process	27	0.585373	1.588529	0.020289	0.020289	0.044163
BP	GO:0007528	neuromuscular junction development	46	-0.48211	-1.5068	0.02042	0.02042	0.044406
BP	GO:0009267	cellular response to starvation	134	0.386913	1.407418	0.020424	0.020424	0.044406
MF	GO:0042626	ATPase-coupled transmembrane transporter activity	101	0.41499	1.444749	0.020437	0.020437	0.044407
BP	GO:0051965	positive regulation of synapse assembly	58	-0.45315	-1.47869	0.020453	0.020453	0.044418
CC	GO:0008076	voltage-gated potassium channel complex	84	-0.40432	-1.4168	0.020592	0.020592	0.044692
BP	GO:0034504	protein localization to nucleus	249	-0.31907	-1.2964	0.020734	0.020734	0.044976

MF	GO:0008301	DNA binding, bending	20	-0.60991	-1.5788	0.02088	0.02088	0.045193
BP	GO:0033866	nucleoside bisphosphate biosynthetic process	63	0.465751	1.502409	0.020883	0.020883	0.045193
BP	GO:0034030	ribonucleoside bisphosphate biosynthetic process	63	0.465751	1.502409	0.020883	0.020883	0.045193
BP	GO:0034033	purine nucleoside bisphosphate biosynthetic process	63	0.465751	1.502409	0.020883	0.020883	0.045193
BP	GO:0043410	positive regulation of MAPK cascade	495	0.298784	1.25201	0.020935	0.020935	0.045279
BP	GO:1903532	positive regulation of secretion by cell	388	0.312626	1.281734	0.020983	0.020983	0.045356
BP	GO:0002719	negative regulation of cytokine production involved in immune	25	0.593754	1.582733	0.021106	0.021106	0.045595
BP	GO:0007129	synapsis	32	-0.52975	-1.5288	0.021244	0.021244	0.045866
BP	GO:1900180	regulation of protein localization to nucleus	111	-0.3808	-1.38974	0.021259	0.021259	0.045873
BP	GO:0032370	positive regulation of lipid transport	59	0.473303	1.506102	0.021362	0.021362	0.046067
BP	GO:0060251	regulation of glial cell proliferation	28	-0.55445	-1.55138	0.021477	0.021477	0.046288
BP	GO:1902622	regulation of neutrophil migration	32	0.554229	1.564046	0.021559	0.021559	0.046439
CC	GO:0005604	basement membrane	91	-0.39449	-1.39827	0.021682	0.021682	0.046647
BP	GO:0030517	negative regulation of axon extension	41	-0.49732	-1.51428	0.021697	0.021697	0.046647
BP	GO:0001708	cell fate specification	97	-0.3877	-1.38909	0.021704	0.021704	0.046647
BP	GO:0045684	positive regulation of epidermis development	35	-0.51724	-1.52457	0.021705	0.021705	0.046647
MF	GO:0099604	ligand-gated calcium channel activity	27	-0.5573	-1.54446	0.021835	0.021835	0.046898
BP	GO:0072522	purine-containing compound biosynthetic process	165	0.365897	1.370534	0.02206	0.02206	0.047354
BP	GO:0048013	ephrin receptor signaling pathway	85	-0.40122	-1.40893	0.022249	0.022249	0.047732
BP	GO:0021545	cranial nerve development	50	-0.46741	-1.48119	0.022314	0.022314	0.047845
BP	GO:0009615	response to virus	297	0.326093	1.302942	0.022365	0.022365	0.047925
BP	GO:0006303	double-strand break repair via nonhomologous end joining	57	-0.45243	-1.47088	0.022591	0.022591	0.048382
BP	GO:0035459	vesicle cargo loading	23	0.6133	1.602477	0.022606	0.022606	0.048386
BP	GO:0006165	nucleoside diphosphate phosphorylation	121	0.393064	1.408785	0.022661	0.022661	0.048475
BP	GO:0048588	developmental cell growth	215	-0.32649	-1.30265	0.022679	0.022679	0.048486
BP	GO:0045778	positive regulation of ossification	79	-0.40722	-1.41198	0.022736	0.022736	0.048553
CC	GO:0120114	Sm-like protein family complex	72	-0.42222	-1.43537	0.022736	0.022736	0.048553
CC	GO:0030532	small nuclear ribonucleoprotein complex	62	-0.44041	-1.45596	0.022754	0.022754	0.048564
BP	GO:0045446	endothelial cell differentiation	98	0.415621	1.439782	0.022813	0.022813	0.048661
BP	GO:0071219	cellular response to molecule of bacterial origin	193	0.353086	1.349776	0.023016	0.023016	0.049067
CC	GO:0097542	ciliary tip	43	-0.49082	-1.51035	0.023069	0.023069	0.049152
BP	GO:0048525	negative regulation of viral process	91	0.417872	1.430352	0.023103	0.023103	0.049186
BP	GO:0002832	negative regulation of response to biotic stimulus	87	0.421899	1.43417	0.023112	0.023112	0.049186
BP	GO:0006865	amino acid transport	139	0.37791	1.383927	0.023148	0.023148	0.049235
MF	GO:0019900	kinase binding	691	-0.26576	-1.18405	0.023221	0.023221	0.049362
BP	GO:0090022	regulation of neutrophil chemotaxis	26	0.583953	1.572543	0.0233	0.0233	0.049501
BP	GO:0042476	odontogenesis	127	-0.36438	-1.35798	0.023361	0.023361	0.049602
BP	GO:0006164	purine nucleotide biosynthetic process	153	0.370357	1.375129	0.023393	0.023393	0.049621
BP	GO:0032692	negative regulation of interleukin-1 production	30	0.560323	1.553293	0.023397	0.023397	0.049621

BP	GO:0042403	thyroid hormone metabolic process	20	0.627341	1.597317	0.023474	0.023474	0.049757
BP	GO:0090087	regulation of peptide transport	696	0.284254	1.220892	0.023502	0.023502	0.049788
BP	GO:0010518	positive regulation of phospholipase activity	58	0.473362	1.502312	0.023729	0.023729	0.05024
BP	GO:0031060	regulation of histone methylation	55	-0.45238	-1.46263	0.023833	0.023833	0.050432
BP	GO:0060325	face morphogenesis	31	-0.53344	-1.53007	0.02386	0.02386	0.05046
CC	GO:0005680	anaphase-promoting complex	20	-0.6026	-1.55988	0.02389	0.02389	0.050495
BP	GO:0005979	regulation of glycogen biosynthetic process	28	0.568753	1.556494	0.024023	0.024023	0.050719
BP	GO:0010962	regulation of glucan biosynthetic process	28	0.568753	1.556494	0.024023	0.024023	0.050719
BP	GO:1903829	positive regulation of cellular protein localization	292	-0.304	-1.25618	0.024083	0.024083	0.050805
BP	GO:0002707	negative regulation of lymphocyte mediated immunity	38	0.520003	1.520943	0.024091	0.024091	0.050805
BP	GO:0048048	embryonic eye morphogenesis	34	-0.51583	-1.50787	0.024106	0.024106	0.050806
BP	GO:0016202	regulation of striated muscle tissue development	125	-0.36406	-1.35106	0.024124	0.024124	0.050816
BP	GO:0050728	negative regulation of inflammatory response	128	0.385687	1.394438	0.024153	0.024153	0.050848
BP	GO:0042832	defense response to protozoan	23	0.610296	1.594628	0.024312	0.024312	0.051155
BP	GO:1904062	regulation of cation transmembrane transport	311	-0.29985	-1.24839	0.024352	0.024352	0.05121
MF	GO:0016798	hydrolase activity, acting on glycosyl bonds	104	0.405387	1.418291	0.024477	0.024477	0.051443
BP	GO:0061337	cardiac conduction	139	-0.35845	-1.35269	0.024491	0.024491	0.051445
BP	GO:0031349	positive regulation of defense response	357	0.312315	1.270527	0.024549	0.024549	0.051536
BP	GO:0002251	organ or tissue specific immune response	24	0.59595	1.570698	0.024665	0.024665	0.051751
BP	GO:0002200	somatic diversification of immune receptors	65	-0.43252	-1.44123	0.02477	0.02477	0.051942
BP	GO:0048261	negative regulation of receptor-mediated endocytosis	23	0.607997	1.588619	0.025165	0.025165	0.052741
MF	GO:0008276	protein methyltransferase activity	60	-0.44019	-1.44556	0.025195	0.025195	0.052746
BP	GO:0002701	negative regulation of production of molecular mediator of im	35	0.532323	1.535105	0.025196	0.025196	0.052746
BP	GO:0032612	interleukin-1 production	101	0.406328	1.414593	0.025397	0.025397	0.053136
BP	GO:0019083	viral transcription	170	-0.34288	-1.32607	0.02542	0.02542	0.053155
BP	GO:0000245	spliceosomal complex assembly	48	-0.47137	-1.48305	0.025475	0.025475	0.05324
BP	GO:0051222	positive regulation of protein transport	408	0.305677	1.258808	0.025494	0.025494	0.053249
BP	GO:0070198	protein localization to chromosome, telomeric region	27	-0.55332	-1.53344	0.025666	0.025666	0.053578
BP	GO:0046939	nucleotide phosphorylation	123	0.388686	1.397735	0.025701	0.025701	0.053621
BP	GO:0042692	muscle cell differentiation	330	-0.29689	-1.24346	0.025761	0.025761	0.053699
BP	GO:0001947	heart looping	60	-0.43878	-1.44091	0.025795	0.025795	0.053699
BP	GO:0007269	neurotransmitter secretion	163	-0.34398	-1.32447	0.025797	0.025797	0.053699
BP	GO:0099643	signal release from synapse	163	-0.34398	-1.32447	0.025797	0.025797	0.053699
CC	GO:0000123	histone acetyltransferase complex	67	-0.42705	-1.43181	0.02581	0.02581	0.053699
BP	GO:0048634	regulation of muscle organ development	128	-0.36082	-1.34616	0.025883	0.025883	0.053821
BP	GO:0030832	regulation of actin filament length	161	-0.34497	-1.32736	0.025903	0.025903	0.053833
BP	GO:0097066	response to thyroid hormone	25	0.585026	1.559467	0.02596	0.02596	0.053921
BP	GO:0048638	regulation of developmental growth	316	-0.29769	-1.24232	0.026008	0.026008	0.053973
BP	GO:0002323	natural killer cell activation involved in immune response	31	0.550985	1.540266	0.026015	0.026015	0.053973

BP	GO:0003197	endocardial cushion development	44	-0.47987	-1.4859	0.02603	0.02603	0.053974
BP	GO:0106027	neuron projection organization	83	-0.39879	-1.39532	0.026122	0.026122	0.054135
BP	GO:0070252	actin-mediated cell contraction	113	-0.37289	-1.3638	0.026171	0.026171	0.054206
BP	GO:0010453	regulation of cell fate commitment	27	-0.5518	-1.5292	0.02624	0.02624	0.05432
BP	GO:0009116	nucleoside metabolic process	97	0.408769	1.413731	0.026263	0.026263	0.054322
MF	GO:0004693	cyclin-dependent protein serine/threonine kinase activity	28	-0.54652	-1.52917	0.02627	0.02627	0.054322
BP	GO:0051261	protein depolymerization	97	-0.38311	-1.37261	0.026326	0.026326	0.054407
MF	GO:0005267	potassium channel activity	119	-0.36586	-1.34816	0.026429	0.026429	0.054589
BP	GO:0002709	regulation of T cell mediated immunity	69	0.447073	1.464394	0.026464	0.026464	0.05463
BP	GO:0010226	response to lithium ion	22	0.608261	1.576899	0.026749	0.026749	0.05519
BP	GO:0003148	outflow tract septum morphogenesis	27	-0.55143	-1.5282	0.026815	0.026815	0.055294
BP	GO:0030539	male genitalia development	23	0.602741	1.574888	0.026871	0.026871	0.05538
MF	GO:0005249	voltage-gated potassium channel activity	83	-0.39807	-1.3928	0.026919	0.026919	0.055448
BP	GO:0062009	secondary palate development	24	-0.56557	-1.52859	0.026986	0.026986	0.055555
BP	GO:0048841	regulation of axon extension involved in axon guidance	32	-0.52124	-1.50425	0.027037	0.027037	0.055629
BP	GO:0051952	regulation of amine transport	91	0.413079	1.413948	0.027086	0.027086	0.055699
BP	GO:0070169	positive regulation of biomineral tissue development	43	-0.48264	-1.48518	0.027175	0.027175	0.055799
BP	GO:0110151	positive regulation of biomineralization	43	-0.48264	-1.48518	0.027175	0.027175	0.055799
BP	GO:0097327	response to antineoplastic agent	88	0.41548	1.41602	0.027193	0.027193	0.055799
BP	GO:0046835	carbohydrate phosphorylation	21	0.611833	1.57009	0.027195	0.027195	0.055799
BP	GO:0051788	response to misfolded protein	24	0.591201	1.558183	0.027217	0.027217	0.055813
BP	GO:0003401	axis elongation	31	-0.52649	-1.51012	0.027323	0.027323	0.056001
BP	GO:0045981	positive regulation of nucleotide metabolic process	42	0.504833	1.507523	0.027606	0.027606	0.056503
BP	GO:1900544	positive regulation of purine nucleotide metabolic process	42	0.504833	1.507523	0.027606	0.027606	0.056503
BP	GO:0007219	Notch signaling pathway	178	-0.3373	-1.31346	0.027614	0.027614	0.056503
BP	GO:0031644	regulation of nervous system process	127	-0.36059	-1.34384	0.027664	0.027664	0.056574
BP	GO:0006611	protein export from nucleus	162	-0.34321	-1.32045	0.027714	0.027714	0.056644
MF	GO:0030545	receptor regulator activity	482	0.297699	1.2446	0.027749	0.027749	0.056655
BP	GO:0001580	detection of chemical stimulus involved in sensory perception	39	0.512442	1.507854	0.027749	0.027749	0.056655
BP	GO:0030168	platelet activation	149	0.36684	1.357578	0.02781	0.02781	0.05673
CC	GO:0070603	SWI/SNF superfamily-type complex	74	-0.41173	-1.40984	0.027816	0.027816	0.05673
MF	GO:0015081	sodium ion transmembrane transporter activity	141	0.37135	1.363535	0.027944	0.027944	0.056958
BP	GO:0051973	positive regulation of telomerase activity	36	-0.50509	-1.50024	0.027976	0.027976	0.056993
BP	GO:0003208	cardiac ventricle morphogenesis	72	-0.41543	-1.4123	0.028321	0.028321	0.057663
BP	GO:0007612	learning	140	-0.35336	-1.3358	0.028359	0.028359	0.057709
BP	GO:0030198	extracellular matrix organization	348	-0.29079	-1.22516	0.028407	0.028407	0.057753
BP	GO:1903018	regulation of glycoprotein metabolic process	40	0.508367	1.503334	0.028411	0.028411	0.057753
BP	GO:1902410	mitotic cytokinetic process	22	-0.57933	-1.53567	0.028523	0.028523	0.057949
BP	GO:0030201	heparan sulfate proteoglycan metabolic process	36	-0.50371	-1.49616	0.028555	0.028555	0.057981

CC	GO:0030684	preribosome	68	-0.41985	-1.4111	0.028777	0.028777	0.058401
BP	GO:0010823	negative regulation of mitochondrion organization	49	0.484709	1.495212	0.028809	0.028809	0.058434
CC	GO:1902554	serine/threonine protein kinase complex	86	-0.39338	-1.38237	0.028846	0.028846	0.058453
CC	GO:0097525	spliceosomal snRNP complex	56	-0.44361	-1.43783	0.02885	0.02885	0.058453
BP	GO:1903901	negative regulation of viral life cycle	75	0.432412	1.43519	0.028994	0.028994	0.058713
MF	GO:0016860	intramolecular oxidoreductase activity	43	0.499509	1.50028	0.029057	0.029057	0.058808
MF	GO:0008194	UDP-glycosyltransferase activity	130	0.379455	1.375355	0.029104	0.029104	0.058871
BP	GO:0031062	positive regulation of histone methylation	32	-0.51743	-1.49324	0.029162	0.029162	0.058956
BP	GO:0043062	extracellular structure organization	349	-0.29048	-1.22374	0.029208	0.029208	0.059018
MF	GO:0016741	transferase activity, transferring one-carbon groups	164	0.359911	1.347802	0.029226	0.029226	0.059023
BP	GO:0002385	mucosal immune response	21	0.608793	1.562289	0.029336	0.029336	0.059212
CC	GO:1904724	tertiary granule lumen	55	0.468873	1.474173	0.029394	0.029394	0.059297
MF	GO:0017046	peptide hormone binding	48	0.487022	1.494817	0.029598	0.029598	0.059676
BP	GO:0007281	germ cell development	230	-0.31611	-1.27264	0.02962	0.02962	0.059688
BP	GO:0019731	antibacterial humoral response	29	0.554356	1.529819	0.029637	0.029637	0.05969
BP	GO:1900076	regulation of cellular response to insulin stimulus	69	0.442237	1.448554	0.029671	0.029671	0.059695
BP	GO:0009314	response to radiation	411	-0.28283	-1.21059	0.029672	0.029672	0.059695
BP	GO:0072384	organelle transport along microtubule	76	-0.40567	-1.3941	0.029707	0.029707	0.059734
BP	GO:0021510	spinal cord development	103	-0.37795	-1.36695	0.029731	0.029731	0.059749
BP	GO:0031076	embryonic camera-type eye development	37	-0.4984	-1.48838	0.02981	0.02981	0.059874
MF	GO:0031492	nucleosomal DNA binding	25	-0.55805	-1.52159	0.029825	0.029825	0.059874
CC	GO:0000315	organellar large ribosomal subunit	53	0.471942	1.475986	0.029998	0.029998	0.060156
CC	GO:0005762	mitochondrial large ribosomal subunit	53	0.471942	1.475986	0.029998	0.029998	0.060156
BP	GO:1904019	epithelial cell apoptotic process	94	0.406476	1.398599	0.030056	0.030056	0.060239
BP	GO:0045737	positive regulation of cyclin-dependent protein serine/threonir	28	-0.54037	-1.51197	0.030105	0.030105	0.060307
BP	GO:0046718	viral entry into host cell	115	0.38823	1.381523	0.030173	0.030173	0.06041
BP	GO:0045939	negative regulation of steroid metabolic process	24	0.587949	1.549611	0.030193	0.030193	0.060418
BP	GO:0003009	skeletal muscle contraction	41	-0.48472	-1.47592	0.030221	0.030221	0.06044
MF	GO:0061733	peptide-lysine-N-acetyltransferase activity	54	-0.4452	-1.43247	0.030405	0.030405	0.060776
MF	GO:0008187	poly-pyrimidine tract binding	33	-0.50965	-1.47882	0.030485	0.030485	0.060904
MF	GO:0042826	histone deacetylase binding	107	-0.37367	-1.35573	0.030556	0.030556	0.061012
BP	GO:0097529	myeloid leukocyte migration	189	0.34828	1.32584	0.030676	0.030676	0.061193
MF	GO:0030215	semaphorin receptor binding	23	-0.56962	-1.52454	0.030679	0.030679	0.061193
CC	GO:0032153	cell division site	62	-0.43105	-1.42504	0.030739	0.030739	0.061278
MF	GO:0015276	ligand-gated ion channel activity	133	-0.35512	-1.3329	0.030877	0.030877	0.061472
MF	GO:0022834	ligand-gated channel activity	133	-0.35512	-1.3329	0.030877	0.030877	0.061472
MF	GO:1990939	ATP-dependent microtubule motor activity	44	-0.47546	-1.47224	0.030886	0.030886	0.061472
MF	GO:0042054	histone methyltransferase activity	46	-0.4664	-1.45772	0.030922	0.030922	0.061511
BP	GO:0003215	cardiac right ventricle morphogenesis	20	-0.59037	-1.52823	0.031038	0.031038	0.06171

BP	GO:0035904	aorta development	53	-0.44669	-1.43097	0.031182	0.031182	0.061935
MF	GO:0019199	transmembrane receptor protein kinase activity	78	-0.40091	-1.38713	0.031188	0.031188	0.061935
BP	GO:0003203	endocardial cushion morphogenesis	35	-0.50412	-1.4859	0.031202	0.031202	0.061935
BP	GO:0007265	Ras protein signal transduction	423	-0.28213	-1.21073	0.031271	0.031271	0.06204
BP	GO:0032330	regulation of chondrocyte differentiation	46	-0.46594	-1.45628	0.031311	0.031311	0.062085
BP	GO:0002704	negative regulation of leukocyte mediated immunity	49	0.481364	1.484894	0.031447	0.031447	0.062321
MF	GO:0030507	spectrin binding	25	-0.55477	-1.51263	0.031535	0.031535	0.06243
BP	GO:0048668	collateral sprouting	25	-0.55495	-1.51312	0.031535	0.031535	0.06243
MF	GO:0098632	cell-cell adhesion mediator activity	50	-0.45458	-1.44054	0.031596	0.031596	0.062517
BP	GO:0060249	anatomical structure homeostasis	393	-0.28509	-1.21555	0.031638	0.031638	0.062568
BP	GO:0034764	positive regulation of transmembrane transport	185	0.350095	1.329016	0.031677	0.031677	0.062611
BP	GO:0061371	determination of heart left/right asymmetry	65	-0.42444	-1.41432	0.031962	0.031962	0.06314
BP	GO:0051146	striated muscle cell differentiation	257	-0.30829	-1.25812	0.032112	0.032112	0.063404
BP	GO:1900542	regulation of purine nucleotide metabolic process	105	0.396786	1.390798	0.032258	0.032258	0.063658
BP	GO:0097479	synaptic vesicle localization	48	-0.46087	-1.45001	0.03253	0.03253	0.064161
BP	GO:0060428	lung epithelium development	35	-0.50236	-1.48068	0.032558	0.032558	0.064182
BP	GO:0006298	mismatch repair	33	-0.50823	-1.47471	0.032621	0.032621	0.064273
BP	GO:0099560	synaptic membrane adhesion	26	-0.54693	-1.50496	0.032646	0.032646	0.064287
BP	GO:0042417	dopamine metabolic process	37	0.512489	1.490413	0.032672	0.032672	0.064304
BP	GO:0048846	axon extension involved in axon guidance	37	-0.49497	-1.47813	0.032714	0.032714	0.064319
BP	GO:1902284	neuron projection extension involved in neuron projection guid	37	-0.49497	-1.47813	0.032714	0.032714	0.064319
CC	GO:0031970	organelle envelope lumen	73	0.433536	1.432247	0.032899	0.032899	0.064648
BP	GO:0036314	response to sterol	29	0.550184	1.518305	0.033161	0.033161	0.065128
CC	GO:0031256	leading edge membrane	157	-0.34249	-1.31326	0.03318	0.03318	0.065132
BP	GO:0042310	vasoconstriction	74	0.430823	1.425008	0.033608	0.033608	0.065938
BP	GO:0006402	mRNA catabolic process	336	-0.29242	-1.22697	0.033668	0.033668	0.06602
BP	GO:0071322	cellular response to carbohydrate stimulus	131	0.375178	1.361605	0.033754	0.033754	0.066153
BP	GO:1902624	positive regulation of neutrophil migration	27	0.563836	1.530083	0.034093	0.034093	0.066784
CC	GO:0090734	site of DNA damage	62	-0.42745	-1.41313	0.034132	0.034132	0.066824
BP	GO:0009309	amine biosynthetic process	22	0.597374	1.548677	0.034239	0.034239	0.066999
BP	GO:0051223	regulation of protein transport	667	0.2814	1.205332	0.034296	0.034296	0.067075
BP	GO:0060996	dendritic spine development	91	-0.38503	-1.36474	0.03433	0.03433	0.067107
BP	GO:0045739	positive regulation of DNA repair	53	-0.44347	-1.42063	0.034359	0.034359	0.067128
BP	GO:0070861	regulation of protein exit from endoplasmic reticulum	25	0.572834	1.526969	0.034403	0.034403	0.067177
BP	GO:0031668	cellular response to extracellular stimulus	246	0.328786	1.289023	0.034522	0.034522	0.067374
BP	GO:1901976	regulation of cell cycle checkpoint	28	-0.53268	-1.49045	0.034708	0.034708	0.067626
BP	GO:0071496	cellular response to external stimulus	312	0.315268	1.266343	0.034716	0.034716	0.067626
BP	GO:0007588	excretion	61	0.451622	1.447147	0.034724	0.034724	0.067626
BP	GO:0002820	negative regulation of adaptive immune response	45	0.487576	1.476369	0.034724	0.034724	0.067626

BP	GO:0050663	cytokine secretion	219	0.335465	1.299478	0.03491	0.03491	0.067954
BP	GO:0008286	insulin receptor signaling pathway	132	0.373161	1.35583	0.035125	0.035125	0.068337
BP	GO:0060323	head morphogenesis	36	-0.49542	-1.47152	0.035308	0.035308	0.068656
BP	GO:0051642	centrosome localization	25	-0.54983	-1.49917	0.035524	0.035524	0.069014
MF	GO:0004714	transmembrane receptor protein tyrosine kinase activity	62	-0.42575	-1.40749	0.035529	0.035529	0.069014
BP	GO:0002706	regulation of lymphocyte mediated immunity	143	0.36393	1.340534	0.035556	0.035556	0.069031
BP	GO:0061842	microtubule organizing center localization	26	-0.54226	-1.49212	0.035701	0.035701	0.069244
BP	GO:0071715	icosanoid transport	48	0.480579	1.47504	0.035722	0.035722	0.069244
BP	GO:1901571	fatty acid derivative transport	48	0.480579	1.47504	0.035722	0.035722	0.069244
BP	GO:1902117	positive regulation of organelle assembly	65	-0.42183	-1.40562	0.035757	0.035757	0.069268
BP	GO:0035019	somatic stem cell population maintenance	71	-0.40935	-1.38784	0.035771	0.035771	0.069268
MF	GO:0036002	pre-mRNA binding	35	-0.49789	-1.46752	0.035853	0.035853	0.069389
CC	GO:0015629	actin cytoskeleton	467	-0.2746	-1.18814	0.036094	0.036094	0.069821
BP	GO:0061311	cell surface receptor signaling pathway involved in heart devel	30	-0.51882	-1.47682	0.036242	0.036242	0.070069
BP	GO:0009166	nucleotide catabolic process	63	0.447554	1.44371	0.036396	0.036396	0.070331
BP	GO:0050684	regulation of mRNA processing	117	-0.36138	-1.32714	0.036435	0.036435	0.070369
BP	GO:0048813	dendrite morphogenesis	136	-0.34863	-1.31197	0.036525	0.036525	0.070507
BP	GO:1900034	regulation of cellular response to heat	74	-0.40275	-1.3791	0.036559	0.036559	0.070529
BP	GO:0060349	bone morphogenesis	111	-0.36682	-1.33869	0.03659	0.03659	0.070529
BP	GO:0090023	positive regulation of neutrophil chemotaxis	22	0.595311	1.543328	0.036593	0.036593	0.070529
BP	GO:0001912	positive regulation of leukocyte mediated cytotoxicity	54	0.462016	1.448439	0.03662	0.03662	0.070543
BP	GO:0009101	glycoprotein biosynthetic process	315	-0.29247	-1.22026	0.036645	0.036645	0.070554
MF	GO:0004402	histone acetyltransferase activity	53	-0.44175	-1.41515	0.036743	0.036743	0.070707
BP	GO:0060236	regulation of mitotic spindle organization	35	-0.49612	-1.4623	0.036822	0.036822	0.070822
BP	GO:0009251	glucan catabolic process	23	0.586	1.531145	0.036895	0.036895	0.070926
BP	GO:0031110	regulation of microtubule polymerization or depolymerization	71	-0.40821	-1.38399	0.03697	0.03697	0.071035
BP	GO:0019430	removal of superoxide radicals	22	0.594892	1.54224	0.037021	0.037021	0.071095
BP	GO:0009755	hormone-mediated signaling pathway	218	0.333491	1.290825	0.037051	0.037051	0.071116
BP	GO:1902930	regulation of alcohol biosynthetic process	72	0.432649	1.426167	0.037089	0.037089	0.071152
BP	GO:0010842	retina layer formation	22	-0.56547	-1.49892	0.037155	0.037155	0.071242
BP	GO:0033273	response to vitamin	89	0.406321	1.386619	0.037244	0.037244	0.071305
BP	GO:0003281	ventricular septum development	70	-0.4089	-1.38188	0.037245	0.037245	0.071305
BP	GO:0032409	regulation of transporter activity	253	-0.30619	-1.24583	0.037246	0.037246	0.071305
CC	GO:0097431	mitotic spindle pole	25	-0.54755	-1.49295	0.037424	0.037424	0.07161
BP	GO:0072527	pyrimidine-containing compound metabolic process	90	0.405893	1.386298	0.03748	0.03748	0.071677
BP	GO:0097756	negative regulation of blood vessel diameter	80	0.417195	1.397611	0.037497	0.037497	0.071677
BP	GO:0008064	regulation of actin polymerization or depolymerization	160	-0.33803	-1.2998	0.037688	0.037688	0.071992
BP	GO:0043277	apoptotic cell clearance	44	0.489051	1.473147	0.037701	0.037701	0.071992
BP	GO:1900026	positive regulation of substrate adhesion-dependent cell sprea	35	0.517336	1.491887	0.037794	0.037794	0.072103

BP	GO:0042063	gliogenesis	269	-0.30023	-1.23008	0.037798	0.037798	0.072103
BP	GO:0007405	neuroblast proliferation	59	-0.42875	-1.40461	0.037853	0.037853	0.072171
BP	GO:0120032	regulation of plasma membrane bounded cell projection asser	148	-0.34265	-1.30248	0.038067	0.038067	0.072542
BP	GO:0098742	cell-cell adhesion via plasma-membrane adhesion molecules	259	-0.30374	-1.24021	0.038295	0.038295	0.072939
BP	GO:0035384	thioester biosynthetic process	49	0.473317	1.460071	0.038547	0.038547	0.073344
BP	GO:0071616	acyl-CoA biosynthetic process	49	0.473317	1.460071	0.038547	0.038547	0.073344
BP	GO:0009713	catechol-containing compound biosynthetic process	20	0.60582	1.542521	0.038626	0.038626	0.073418
BP	GO:0042423	catecholamine biosynthetic process	20	0.60582	1.542521	0.038626	0.038626	0.073418
BP	GO:1903845	negative regulation of cellular response to transforming growt	72	-0.40565	-1.37903	0.038692	0.038692	0.073506
BP	GO:0002224	toll-like receptor signaling pathway	136	0.368466	1.343701	0.03879	0.03879	0.073655
BP	GO:1904353	regulation of telomere capping	25	-0.54549	-1.48733	0.038944	0.038944	0.073872
BP	GO:0061217	regulation of mesonephros development	25	-0.54556	-1.48752	0.038944	0.038944	0.073872
CC	GO:0099091	postsynaptic specialization, intracellular component	21	-0.57389	-1.50346	0.03901	0.03901	0.073959
BP	GO:0060563	neuroepithelial cell differentiation	49	-0.45111	-1.42361	0.03903	0.03903	0.07396
BP	GO:0003170	heart valve development	58	-0.43072	-1.40548	0.039302	0.039302	0.074438
BP	GO:0019080	viral gene expression	183	-0.32664	-1.27688	0.039344	0.039344	0.074476
BP	GO:0005980	glycogen catabolic process	22	0.592111	1.535031	0.039375	0.039375	0.074476
BP	GO:0043470	regulation of carbohydrate catabolic process	81	0.415355	1.393144	0.039397	0.039397	0.074476
BP	GO:1904407	positive regulation of nitric oxide metabolic process	40	0.495524	1.465353	0.039403	0.039403	0.074476
BP	GO:0010517	regulation of phospholipase activity	69	0.433102	1.418634	0.039495	0.039495	0.074613
BP	GO:1901655	cellular response to ketone	90	0.403309	1.377471	0.039673	0.039673	0.074911
BP	GO:0002673	regulation of acute inflammatory response	43	0.487613	1.46455	0.039697	0.039697	0.074919
BP	GO:0030833	regulation of actin filament polymerization	145	-0.34154	-1.29575	0.039817	0.039817	0.075077
BP	GO:0072698	protein localization to microtubule cytoskeleton	44	-0.46372	-1.43587	0.039821	0.039821	0.075077
BP	GO:0010528	regulation of transposition	21	-0.57206	-1.49866	0.039947	0.039947	0.075193
BP	GO:0010529	negative regulation of transposition	21	-0.57206	-1.49866	0.039947	0.039947	0.075193
BP	GO:0050912	detection of chemical stimulus involved in sensory perception	44	0.486215	1.464603	0.039967	0.039967	0.075193
BP	GO:0002562	somatic diversification of immune receptors via germline recor	57	-0.43091	-1.40094	0.039984	0.039984	0.075193
BP	GO:0016444	somatic cell DNA recombination	57	-0.43091	-1.40094	0.039984	0.039984	0.075193
BP	GO:0010212	response to ionizing radiation	130	-0.35242	-1.31772	0.040017	0.040017	0.075216
BP	GO:0035115	embryonic forelimb morphogenesis	32	-0.50488	-1.45702	0.04017	0.04017	0.075443
BP	GO:0031647	regulation of protein stability	261	0.321984	1.26928	0.040178	0.040178	0.075443
MF	GO:0005109	frizzled binding	39	-0.47878	-1.44435	0.040209	0.040209	0.075463
MF	GO:0035250	UDP-galactosyltransferase activity	20	-0.5772	-1.49414	0.040256	0.040256	0.075468
MF	GO:0022824	transmitter-gated ion channel activity	61	-0.42247	-1.3912	0.040272	0.040272	0.075468
MF	GO:0022835	transmitter-gated channel activity	61	-0.42247	-1.3912	0.040272	0.040272	0.075468
BP	GO:0051235	maintenance of location	294	0.315251	1.259292	0.040402	0.040402	0.075673
BP	GO:0015695	organic cation transport	32	0.528254	1.490744	0.040423	0.040423	0.075674
MF	GO:0003730	mRNA 3'-UTR binding	89	-0.3825	-1.35053	0.040554	0.040554	0.075881

CC	GO:0005769	early endosome	318	0.311793	1.254491	0.040578	0.040578	0.075887
BP	GO:0051931	regulation of sensory perception	34	-0.49699	-1.45279	0.04063	0.04063	0.075946
BP	GO:0006656	phosphatidylcholine biosynthetic process	37	0.503012	1.462851	0.040736	0.040736	0.076107
BP	GO:0032094	response to food	34	0.518484	1.485896	0.040758	0.040758	0.076109
CC	GO:0043202	lysosomal lumen	89	0.404026	1.378786	0.040829	0.040829	0.076203
BP	GO:0006110	regulation of glycolytic process	72	0.428824	1.413558	0.040898	0.040898	0.076295
BP	GO:0051494	negative regulation of cytoskeleton organization	127	-0.35193	-1.31159	0.040984	0.040984	0.076416
BP	GO:2000008	regulation of protein localization to cell surface	38	-0.4817	-1.44522	0.041257	0.041257	0.076865
BP	GO:1901292	nucleoside phosphate catabolic process	69	0.4317	1.414041	0.041299	0.041299	0.076865
BP	GO:0055123	digestive system development	139	-0.34357	-1.29655	0.041303	0.041303	0.076865
BP	GO:0006970	response to osmotic stress	80	0.413836	1.386359	0.041307	0.041307	0.076865
BP	GO:0032196	transposition	25	-0.54193	-1.47763	0.041413	0.041413	0.077024
BP	GO:0046596	regulation of viral entry into host cell	29	0.540834	1.492503	0.042073	0.042073	0.078211
BP	GO:0051347	positive regulation of transferase activity	609	-0.26169	-1.15617	0.04211	0.04211	0.078242
BP	GO:0090189	regulation of branching involved in ureteric bud morphogenes	22	-0.55915	-1.48218	0.042222	0.042222	0.07841
BP	GO:0045429	positive regulation of nitric oxide biosynthetic process	39	0.496202	1.460068	0.042245	0.042245	0.078413
BP	GO:0072503	cellular divalent inorganic cation homeostasis	460	0.29322	1.221	0.042365	0.042365	0.078596
MF	GO:0008327	methyl-CpG binding	21	-0.56941	-1.49173	0.042386	0.042386	0.078596
BP	GO:0060491	regulation of cell projection assembly	150	-0.33983	-1.29312	0.042437	0.042437	0.078617
BP	GO:0050706	regulation of interleukin-1 beta secretion	44	0.482337	1.452921	0.042439	0.042439	0.078617
CC	GO:0031300	intrinsic component of organelle membrane	325	0.309518	1.248549	0.042561	0.042561	0.078803
BP	GO:0071772	response to BMP	156	-0.33727	-1.29161	0.042633	0.042633	0.078859
BP	GO:0071773	cellular response to BMP stimulus	156	-0.33727	-1.29161	0.042633	0.042633	0.078859
BP	GO:0002823	negative regulation of adaptive immune response based on sc	40	0.492051	1.455084	0.042721	0.042721	0.078981
CC	GO:0035861	site of double-strand break	49	-0.44754	-1.41236	0.042775	0.042775	0.079037
BP	GO:0003014	renal system process	114	0.377945	1.344839	0.042793	0.042793	0.079037
BP	GO:2000177	regulation of neural precursor cell proliferation	83	-0.3853	-1.34814	0.042871	0.042871	0.079142
BP	GO:0006541	glutamine metabolic process	21	0.591653	1.518304	0.043041	0.043041	0.079415
BP	GO:0030072	peptide hormone secretion	238	0.325297	1.273334	0.043073	0.043073	0.079435
BP	GO:0009593	detection of chemical stimulus	447	0.292943	1.216144	0.043193	0.043193	0.079618
BP	GO:0099003	vesicle-mediated transport in synapse	207	-0.31666	-1.25759	0.043599	0.043599	0.080325
MF	GO:0002020	protease binding	122	0.371885	1.334927	0.043774	0.043774	0.080579
BP	GO:0071450	cellular response to oxygen radical	24	0.56753	1.495794	0.043802	0.043802	0.080579
BP	GO:0071451	cellular response to superoxide	24	0.56753	1.495794	0.043802	0.043802	0.080579
BP	GO:0043576	regulation of respiratory gaseous exchange	22	-0.55768	-1.47828	0.043911	0.043911	0.08074
BP	GO:0006909	phagocytosis	250	0.321835	1.26417	0.044085	0.044085	0.08102
CC	GO:0032154	cleavage furrow	49	-0.44648	-1.409	0.044155	0.044155	0.081109
BP	GO:0044247	cellular polysaccharide catabolic process	25	0.558436	1.488587	0.044322	0.044322	0.081376
BP	GO:0002718	regulation of cytokine production involved in immune respons	83	0.409622	1.380627	0.044516	0.044516	0.08169

MF	GO:0030021	extracellular matrix structural constituent conferring compressi	22	0.582151	1.50921	0.044725	0.044725	0.082029
CC	GO:0098858	actin-based cell projection	197	-0.31886	-1.25703	0.044744	0.044744	0.082029
BP	GO:0015012	heparan sulfate proteoglycan biosynthetic process	31	-0.50286	-1.44235	0.044834	0.044834	0.082152
BP	GO:0032743	positive regulation of interleukin-2 production	30	-0.51055	-1.45326	0.044871	0.044871	0.082179
BP	GO:0060968	regulation of gene silencing	92	-0.37728	-1.34003	0.045043	0.045043	0.082455
BP	GO:0051170	import into nucleus	155	-0.33558	-1.28362	0.045113	0.045113	0.082542
BP	GO:0001909	leukocyte mediated cytotoxicity	103	0.3865	1.347273	0.045183	0.045183	0.082603
BP	GO:0045601	regulation of endothelial cell differentiation	32	0.52384	1.478288	0.045191	0.045191	0.082603
BP	GO:0003298	physiological muscle hypertrophy	26	0.553766	1.491251	0.04534	0.04534	0.082744
BP	GO:0003301	physiological cardiac muscle hypertrophy	26	0.553766	1.491251	0.04534	0.04534	0.082744
BP	GO:0061049	cell growth involved in cardiac muscle cell development	26	0.553766	1.491251	0.04534	0.04534	0.082744
BP	GO:1990573	potassium ion import across plasma membrane	47	-0.45157	-1.41512	0.045366	0.045366	0.082744
CC	GO:0070382	exocytic vesicle	197	-0.3186	-1.25602	0.045386	0.045386	0.082744
BP	GO:0042542	response to hydrogen peroxide	130	0.367439	1.331804	0.045402	0.045402	0.082744
BP	GO:0035195	gene silencing by miRNA	109	-0.36118	-1.31449	0.045482	0.045482	0.082851
CC	GO:0030139	endocytic vesicle	293	0.31336	1.251345	0.045529	0.045529	0.082896
BP	GO:0046777	protein autophosphorylation	229	-0.30808	-1.23975	0.045903	0.045903	0.083535
BP	GO:0097061	dendritic spine organization	75	-0.39482	-1.35379	0.045991	0.045991	0.083654
MF	GO:0016667	oxidoreductase activity, acting on a sulfur group of donors	52	0.458844	1.428723	0.046145	0.046145	0.083893
BP	GO:0070286	axonemal dynein complex assembly	23	-0.54875	-1.46867	0.046302	0.046302	0.084137
BP	GO:0014823	response to activity	60	0.440137	1.405516	0.046791	0.046791	0.084984
BP	GO:0019886	antigen processing and presentation of exogenous peptide an	94	-0.37335	-1.33088	0.047007	0.047007	0.085335
CC	GO:0005697	telomerase holoenzyme complex	21	-0.56182	-1.47185	0.047074	0.047074	0.085416
BP	GO:0098743	cell aggregation	21	-0.56104	-1.4698	0.047262	0.047262	0.085714
BP	GO:0030048	actin filament-based movement	135	-0.34351	-1.29191	0.047727	0.047727	0.086515
BP	GO:0046685	response to arsenic-containing substance	32	0.520036	1.467552	0.047886	0.047886	0.086761
BP	GO:0050905	neuromuscular process	100	-0.36835	-1.32352	0.047937	0.047937	0.086812
MF	GO:0008536	Ran GTPase binding	38	-0.47464	-1.42406	0.048197	0.048197	0.087225
BP	GO:1904951	positive regulation of establishment of protein localization	423	0.293872	1.21477	0.048212	0.048212	0.087225
CC	GO:0005770	late endosome	224	0.325772	1.265949	0.048333	0.048333	0.087401
MF	GO:0030145	manganese ion binding	57	0.444842	1.407181	0.0484	0.0484	0.08748
BP	GO:0046530	photoreceptor cell differentiation	62	-0.41513	-1.37239	0.048703	0.048703	0.087984
BP	GO:0097178	ruffle assembly	37	-0.47754	-1.42608	0.04878	0.04878	0.088082
BP	GO:0048565	digestive tract development	128	-0.34714	-1.2951	0.048891	0.048891	0.088238
MF	GO:0038024	cargo receptor activity	69	0.425544	1.393877	0.048917	0.048917	0.088243
BP	GO:0071806	protein transmembrane transport	53	0.452296	1.414543	0.049124	0.049124	0.088574
BP	GO:0019233	sensory perception of pain	94	-0.37199	-1.32603	0.049217	0.049217	0.088697
BP	GO:2000278	regulation of DNA biosynthetic process	100	-0.36764	-1.32095	0.049353	0.049353	0.088899
BP	GO:0071622	regulation of granulocyte chemotaxis	39	0.488911	1.438615	0.049493	0.049493	0.089108

BP	GO:0050954	sensory perception of mechanical stimulus	151	-0.33488	-1.27566	0.049632	0.049632	0.089316
BP	GO:0070199	establishment of protein localization to chromosome	21	-0.55747	-1.46043	0.0497	0.0497	0.089395
MF	GO:0046873	metal ion transmembrane transporter activity	424	-0.27529	-1.18071	0.049805	0.049805	0.08954

rank
2654
1562
2698
2336
2091
2271
2157
2447
2922
2722
2922
2079
4509
3614
1875
2646
2266
4509
2047
2125
2698
2336
1418
2260
2477
2910
2477
2106
2077
2091
1817
1942
1751
2722
1915
2106
1894

2197
2260
2690
2080
2197
2447
2260
1875
4538
2142
3092
2646
1953
2051
2211
1962
2069
2080
3596
1908
1892
2388
3153
2115
4538
2069
2835
2211
1875
2112
1875
1647
2734
2093
1991
2069
2081
2079
2069

1200
1931
2138
1600
2128
2122
2098
2681
2662
1515
2574
2098
1600
2098
2420
2061
1604
1688
1875
2211
1604
1875
1539
1604
1821
2080
2237
1815
2738
4237
2211
2087
1883
2924
2106
2398
2080
2080
2134

1127
1716
2037
2690
1815
2278
2690
1875
2395
2223
1953
2140
2197
1815
2608
3416
2051
2223
3363
1600
2094
1617
814
2063
4272
2157
5228
1236
1649
2715
2042
2613
2585
2122
1649
1474
1815
2054
1748

1748
1771
2084
2223
1771
1075
2270
3833
2061
4493
2216
2398
939
4229
1748
1815
2061
3323
1954
1954
2733
2751
1701
2219
1600
1617
1771
2388
1676
1013
1332
697
1409
1409
721
721
721
721
2398

1815
1931
1803
1409
1409
711
1911
995
812
2219
2082
1701
2398
2214
1600
1600
1600
2378
1551
1485
2081
2440
1409
2249
995
2061
2172
2378
812
1706
2507
1890
1600
2396
2396
1047
3112
1890
1300

1815
2523
1874
2630
2172
2080
1749
1716
1629
1925
2937
812
1822
1704
1968
1047
2558
794
1749
794
1816
1075
2157
684
3033
2432
1409
995
711
995
995
1485
2923
2061
1749
3301
2042
1815
2061

2001
3141
2061
1600
1600
4493
1771
1112
1617
920
1138
1815
1047
1771
1409
3419
2085
1379
995
1890
1617
3301
1557
4257
1502
3141
3141
1474
5103
1908
1340
1013
2013
1638
1617
2013
2929
2243
1409

4912
2577
2954
2954
1534
2243
1762
1707
1138
1766
1178
4493
1047
1047
1890
2038
2038
2038
4493
1725
2314
3776
5274
1409
1959
4532
1409
1716
1780
2091
2396
920
1847
4493
2114
1047
971
5355
5390

711
5135
3514
2098
1480
2063
5274
711
2877
2061
2061
1312
2085
6039
1822
1533
4862
1617
5103
3265
1649
2179
2179
2038
2061
2061
1815
1816
2114
920
5103
5103
1409
1178
2428
2555
4717
3917
5097

5097
3751
3195
4992
2844
1114
3962
3625
2685
2628
3406
2215
1409
920
971
1815
2507
3105
920
3917
4752
4862
6616
4717
2555
4771
2469
4992
3917
3319
3776
5277
4911
920
1845
4490
1617
1766
1047

2038
2038
2140
1701
5355
5355
1003
2425
2428
4992
2428
4862
1515
983
5071
3610
3610
3822
2428
684
1617
1534
4532
4876
2243
3319
4912
794
1471
1471
1146
616
2084
2243
4911
4294
5471
2494
4532

3962
1276
2054
5228
1466
1466
1146
1780
1515
1617
1098
1098
1456
1563
1859
1859
971
2259
8980
4294
3350
8113
8113
5000
889
889
869
1785
889
3824
1580
1600
2406
3610
3610
1771
696
2259
1600

794
1598
713
1527
889
1716
1544
2391
769
972
1182
3406
5038
1002
2270
735
1859
2013
2439
4297
4278
4294
3842
5207
4294
2258
3842
3905
804
2811
572
1771
1771
408
1563
1669
2492
735
1771

6084
1456
4912
3879
4644
889
1794
1047
1047
5193
8849
4862
7073
4862
3062
3616
3616
2080
4934
3628
3842
2791
4968
2974
6152
3776
4753
1875
670
2259
8988
4926
1035
1536
1536
1845
924
1051
1953

1276
1294
423
5668
5025
4412
4876
5827
3874
2258
335
726
548
4781
2804
4412
1845
1707
2494
4149
3842
4926
5163
5026
4911
3879
3842
4911
5133
7558
3842
4644
5071
5314
5386
5778
3708
5318
2974

7148
4968
4610
4164
4052
3874
4052
5827
3844
4191
4912
6059
5025
5120
5277
4926
4217
8931
8931
6059
5183
5581
5163
5392
8931
5120
4926
4396
5343
4191
5907
4396
3003
4222
5609
4305
3733
4131
4396

3616
5071
2627
3905
5609
9106
8931
4443
5009
3842
2817
4992
3865
3651
4191
4159
6547
5594
4629
7838
5066
6098
5412
4305
8931
4271
3733
5025
4164
5097
4131
5293
5298
4087
5339
5343
6216
5343
4912

4164
4386
3683
4258
3865
5581
4992
5609
3683
3865
5194
5581
4423
5636
5698
4305
5277
3625
5071
4412
3522
5066
4060
4433
5698
6142
4052
5343
5698
4288
5698
4433
4258
5390
5698
5274
5025
4401
4401

5314
5071
4401
8034
2271
2343
1958
3060
3328
1815
2537
3328
1511
2211
3481
1836
1836
2388
1689
2615
1815
2112
2182
2455
2923
1129
2063
2817
4418
4418
1807
2260
626
2934
2084
2817
1701
2432
2140

1717
3471
3081
2224
1534
1812
8922
8922
1298
8922
8922
1959
2395
8922
3350
3350
1151
1614
2172
3038
912
1534
5228
5228
5228
1457
1815
3083
1959
754
3378
2214
1002
1551
5228
5228
5228
2681
2394

2259
2259
1527
8931
865
2259
2259
1995
4218
5593
8849
8849
865
865
1617
1600
2389
1800
1669
1115
971
1780
1794
971
1715
2342
2018
4217
1603
1674
1600
2224
2224
3548
3548
5265
8985
4191
5609

4258
4433
5392
5229
8788
2852
2852
2266
2528
2008
2008
2182
3246
2140
4100
4100
1689
1689
2391
5122
4799
5600
5267
2758
2733
2733
2817
5600
2084
4734
1474
8113
742
889
3538
2201
994
4318
1968

3538
3206
3054
2738
1781
5025
1762
2182
2114
2114
2523
765
3105
3105
2717
1669
1762
1617
1617
3442
1408
912
2473
8894
1617
1190
1781
4164
4161
4161
1265
5229
4191
5025
5218
6826
4258
5073
4332

4950
4255
3481
1845
3833
2686
1129
1219
4862
4100
5600
4890
4135
5647
4229
4207
2639
2394
4164
3602
3602
3625
1151
1988
1159
5827
5593
2473
971
4217
7558
3076
1674
2779
5249
3857
3007
6408
5073

4100
4862
4890
1725
2834
2198
1551
1468
6012
5365
5696
5696
2182
3073
3937
3073
2789
2842
3073
3073
5274
2317
2717
1800
3833
1815
1041
2008
2260
2654
8894
4482
2224
2970
3033
763
3363
2216
2087

1689
4753
4272
4532
5097
2829
1080
3379
5420
3206
4721
2008
3073
3073
3616
430
2111
4161
6098
4131
1815
2260
1962
2805
2157
2179
6677
2654
1649
2615
8113
5277
4721
4131
2031
3265
5204
3092
4625

2218
4939
3350
6087
9106
4610
4164
5410
4562
5609
5038
3060
6826
2802
2717
2182
2182
3265
5262
4164
6672
1766
883
4482
2271
1985
3323
3148
4433
5097
2413
2413
3123
2970
2063
4191
3538
2140
5696

3833
1080
9118
4164
6605
1617
4191
4191
2574
2219
883
1785
2391
5025
5365
2970
2087
2970
4407
4271
8113
2474
3060
4164
4257
4257
6672
846
2198
2111
4164
7812
7966
4396
6142
2970
5600
1070
1409

2179
1784
1823
2150
8113
5410
4257
3514
2467
448
4213
865
2008
5038
2951
3228
2839
2528
2639
1815
4164
4255
6672
4028
2717
2134
2970
1649
2504
2504
2198
4331
2523
4102
4432
2923
2001
8931
2789

4278
4759
3602
1815
2241
2241
1648
3520
4915
2844
1772
5473
5456
2419
1766
742
742
2558
6540
860
5120
8922
1797
2528
5314
2974
1196
1815
1511
4541
3350
5048
5120
2259
5232
2910
3905
9106
8113

4911
4911
4911
3669
3303
3460
4443
3378
4532
2804
1649
1433
2756
3160
1488
3265
3350
2631
4212
2111
5298
5593
2756
6690
3261
4679
4191
1627
1815
2216
2937
1945
2439
3292
5576
3833
4911
4911
2699

2861
1340
1485
2982
3844
5555
1709
1996
8113
2160
4852
5932
5835
3073
4196
2873
2160
1742
4735
846
3538
1866
1945
3844
4331
2970
5365
1195
4367
1960
8988
4235
5056
5093
5696
2586
1437
8113
2198

4856
2724
1962
2065
4096
4968
3520
4217
4911
5298
3520
2455
3041
4911
4541
846
4164
3994
3918
1616
4911
2933
3641
4278
2481
5212
6892
6892
2243
2084
6687
3160
4862
6059
6677
1896
3921
2050
5025

1614
3460
2631
4323
1949
2861
2861
1772
9106
2237
860
4433
3076
6871
1617
4852
2690
4739
1717
4051
3187
4911
4911
1021
1021
6986
2983
2901
1218
8113
5314
5120
2523
4102
430
4164
5343
6142
4257

5696
5696
5696
3801
1253
2305
7031
5441
5441
4225
2652
2914
4545
4911
3532
3428
5525
5525
5365
4540
4164
4881
5804
1205
1205
4071
2738
5928
8263
4407
4911
3133
5106
1800
4051
5937
6826
2714
1709

1888
2260
5778
2654
7031
5194
4856
3918
398
3474
4677
3145
1742
2819
1179
6207
4412
3848
5942
2050
4071
7967
5867
2654
2901
2690
3538
4164
3081
5194
4921
4051
3037
4051
3357
7137
4911
4071
1874

4191
4071
9942
5984
3647
4051
3833
3545
3350
1771
777
6035
5609
1962
3145
2530
2114
7999
2297
8113
2667
1771
1771
3342
2988
1771
876
4051
887
5026
1706
1803
1803
3849
1617
8620
6922
5672
3538

5025
5101
4278
4278
4278
4433
2861
4547
7172
7172
3387
3387
7013
8113
2128
4691
4739
3041
4844
2111
3448
4720
1803
4222
8620
2652
5343
4911
4547
1236
3081
5122
4164
5480
5135
2417
7804
1820
3081

5066
2523
4678
5101
4433
3641
2165
5066
2257
3428
6515
2207
8263
2093
1041
5377
1954
1780
3148
3962
1638
3474
6032
1820
5249
7308
7308
8028
3641
7013
2054
4213
4531
4164
5056
2270
2536
3641
3905

1715
2182
5183
4102
2571
1848
1468
1938
2829
2111
3792
7148
8620
8620
846
5559
5314
2093
2861
2031
1979
3468
2901
2455
4396
8922
2974
7308
5654
5066
3406
4493
1571
8927
7999
4811
3355
3520
1979

1962
3432
4820
1340
2455
1814
7920
3728
6778
3213
7835
4903
2401
2523
2523
3148
5543
3967
3905
8045
2555
5249
1836
2157
3366
4051
404
5713
5668
1979
4532
5470
586
4540
8258
1864
5107
1814
3593

5309
2043
2965
1814
4208
7178
7013
5011
4251
3538
2054
2054
4429
6672
2054
5249
3848
1787
5249
5120
5636
8286
649
4691
4983
1784
4005
3741
4628
4005
2690
4532
3471
5260
4419
4255
2391
2142
1209

4179
735
6377
2875
3600
4332
6986
1803
4865
6563
1874
448
5385
5343
2469
935
3344
2069
3051
4410
5143
4933
3520
430
3625
5312
1376
6454
2069
6523
4164
3303
4301
2469
3538
649
4131
4102
6774

2821
1600
1600
1600
2043
1962
2087
4190
5183
742
6034
1451
2004
4051
4889
4541
4191
2690
6738
5299
3538
4911
3558
2054
3552
3962
9894
9894
3821
4005
5412
2966
4250
1848
5778
3658
4865
2690
1621

549
2477
1908
5122
6142
4367
2111
2111
4984
2198
1229
2974
1156
3315
4429
4096
4191
4091
1953
5386
320
7091
2087
2639
8679
8650
2103
7527
2054
3970
4164
4348
4348
7308
2974
3007
3370
4217
2512

5056
2358
4191
3497
2260
6986
4637
4102
3492
2214
4164
1826
4102
2428
4051
1253
4419
4419
2182
1603
3792
3752
1874
1874
4179
3647
8927
1820
161
1577
7308
1085
8286
6361
6558
2439
876
697
5343

9089
3116
6659
9894
2966
2647
935
2686
2439
1674
1953
2639
2419
5193
2001
2211
5370
5945
3610
5576
5739
5543
5543
2198
1331
3533
1669
2716
7704
8269
6660
2349
2970
1747
4811
4811
3514
7008
3520

4547
4258
5056
4759
4781
2198
2653
2439
2811
5027
2136
4164
3600
1874
3155
640
5074
3037
2241
4051
4051
2875
1159
5044
3448
8988
1814
1417
8020
1845
2477
2304
5038
2557
2786
5274
3172
1844
2198

2646
2211
6142
4159
4258
3492
4159
1386
1386
3674
3752
6874
4068
3520
3283
8922
4412
8656
5156
1316
3944
5310
7704
6523
2179
5163
1303
2848
3105
3694
2052
4739
4213
7812
2260
2606
3007
3409
743

5222
4863
4361
3555
1600
1600
2106
2106
1709
3370
7966
6395
4032
2707
3647
8613
1759
1762
2923
1908
2142
486
3007
3593
4385
4385
2392
5386
5386
2064
3115
2969
4258
5835
4811
4811
2711
1823
7232

1775
2773
1301
1041
3468
1762
3136
4087
3283
3967
1742
4385
3160
5443
6395
2923
2722
6290
4361
846
4250
5021
5021
2198
8230
2966
6992
822
1954
1457
4332
2192
1303
1303
2871
3141
3245
2179
3482

2543
2031
5310
5785
8403
7013
3982
2420
1771
1771
1771
1230
4332
3478
7013
2697
5636
2358
3960
5867
2232
626
8258
1829
4196
1477
3641
7031
2103
3067
3428
5804
3906
3967
2724
4666
2773
7967
1907

2774
7527
4191
