# Supplementary material for: Comprehensive analysis of complement-associated molecular features in hepatocellular carcinoma: Complement-associated molecular features in hepatocellular carcinoma
Source: Acta Biochim Biophys Sin (Shanghai). 2022 Aug 2;54(11):1694–707. doi: 10.3724/abbs.2022097 (PMC9828444; doi:10.3724/abbs.2022097)
Supplement: Supplementary_table_7 [file Supplementary_table_7.pdf]

Supplementary Table S7. Clinicopathologic features of the training and validation cohorts from TCGA-L

	Training cohort (N=255)	Validatio n cohort (N=96)	Overall (N=351)	P-value
**Gender**				0.1737
Female	77 (0.30)	37 (0.39)	114 (0.32)	
Male	178 (0.70)	59 (0.61)	237 (0.68)	
**Race**				0.6107
White	121 (0.47)	48 (0.50)	169 (0.48)	
Black or African Americ	11 (0.04)	6 (0.06)	17 (0.05)	
Asian	117 (0.46)	37 (0.39)	154 (0.44)	
American Indian or Alasl	1 (0.00)	0 (0.00)	1 (0.00)	
Missing	5 (2.0%)	5 (5.2%)	10 (2.8%)	
**T stage**				0.1388
T1	130 (0.51)	43 (0.45)	173 (0.49)	
T2	61 (0.24)	25 (0.26)	86 (0.25)	
T3	57 (0.22)	19 (0.20)	76 (0.22)	
T4	6 (0.02)	7 (0.07)	13 (0.04)	
Missing	1 (0.4%)	2 (2.1%)	3 (0.9%)	
**N stage**				0.2612
N0	180 (0.71)	62 (0.65)	242 (0.69)	
N1	3 (0.01)	0 (0.00)	3 (0.01)	
NX	72 (0.28)	34 (0.35)	106 (0.30)	
**M stage**				0.2379
M0	188 (0.74)	66 (0.69)	254 (0.72)	
M1	4 (0.02)	0 (0.00)	4 (0.01)	
MX	63 (0.25)	30 (0.31)	93 (0.26)	
**TNM stage**				0.4645
I	125 (0.49)	39 (0.41)	164 (0.47)	
II	55 (0.22)	23 (0.24)	78 (0.22)	
III	58 (0.23)	23 (0.24)	81 (0.23)	
IV	4 (0.02)	0 (0.00)	4 (0.01)	
Missing	13 (5.1%)	11 (11.5%)	24 (6.8%)	
**Pathological grade**				0.3184
1	33 (0.13)	18 (0.19)	51 (0.15)	
2	123 (0.48)	45 (0.47)	168 (0.48)	
3	90 (0.35)	26 (0.27)	116 (0.33)	
4	7 (0.03)	4 (0.04)	11 (0.03)	
Missing	2 (0.8%)	3 (3.1%)	5 (1.4%)	

.IHC cohort
