# Supplementary material for: Comprehensive analysis of complement-associated molecular features in hepatocellular carcinoma: Complement-associated molecular features in hepatocellular carcinoma
Source: Acta Biochim Biophys Sin (Shanghai). 2022 Aug 2;54(11):1694–707. doi: 10.3724/abbs.2022097 (PMC9828444; doi:10.3724/abbs.2022097)
Supplement: Supplementary_table_5 [file Supplementary_table_5.pdf]

Supplementary Table S5. Differentially expressed miRNAs between complement score-low and score-high groups

name	logFC	AveExpr	t	P.Value	adj.P.Val	B
hsa-mir-5589	2.708557	3.059615	11.5345	1.88E-24	3.51E-21	43.51219
hsa-mir-885	2.630087	7.833047	10.8995	1.85E-22	1.73E-19	38.93753
hsa-mir-1295b	2.07765	3.338656	10.33963	9.95E-21	6.20E-18	34.97058
hsa-mir-122	2.565417	13.74779	9.588049	1.87E-18	8.72E-16	29.76568
hsa-mir-99b	-1.21682	13.38332	-8.3821	5.84E-15	2.18E-12	21.77926
hsa-mir-29c	1.103144	10.68205	7.601693	8.01E-13	2.14E-10	16.9104
hsa-mir-378c	1.381084	3.862973	7.524074	1.29E-12	3.01E-10	16.44107
hsa-mir-192	1.268405	14.92728	7.173234	1.07E-11	1.91E-09	14.35618
hsa-mir-99a	1.494762	9.026092	7.050161	2.20E-11	3.17E-09	13.63948
hsa-mir-139	1.320128	6.703402	6.963306	3.67E-11	4.90E-09	13.13845
hsa-mir-194-1	1.273223	12.50657	6.898589	5.34E-11	6.66E-09	12.76772
hsa-mir-194-2	1.283303	12.78219	6.866144	6.45E-11	7.53E-09	12.58271
hsa-mir-215	1.621448	6.518213	6.661005	2.09E-10	2.30E-08	11.42622
hsa-mir-125b-1	1.061132	8.78075	6.290069	1.65E-09	1.40E-07	9.395455
hsa-mir-125b-2	1.209618	4.390548	6.262864	1.92E-09	1.56E-07	9.249687
hsa-mir-1468	1.123039	3.543078	6.04188	6.31E-09	4.37E-07	8.082209
hsa-let-7c	1.136588	10.67196	5.93485	1.11E-08	7.01E-07	7.527585
hsa-mir-135b	-1.00406	1.004028	-5.9328	1.12E-08	7.01E-07	7.517038
hsa-mir-100	1.056003	12.06899	5.174907	5.07E-07	2.21E-05	3.80451
hsa-mir-6715a	1.326431	3.334861	5.044987	9.39E-07	3.82E-05	3.20758
hsa-mir-323a	-1.04149	1.86009	-4.91712	1.70E-06	5.89E-05	2.631902
hsa-mir-216a	1.95908	3.431263	4.844163	2.38E-06	7.53E-05	2.308765
hsa-mir-429	-1.40385	3.278164	-4.73235	3.94E-06	0.000112	1.821139
hsa-mir-216b	1.979997	3.244353	4.645743	5.79E-06	0.000153	1.449852
hsa-mir-217	2.259174	8.464426	4.645527	5.79E-06	0.000153	1.448934
hsa-mir-200c	-1.18251	5.649025	-4.59145	7.35E-06	0.000183	1.219968
hsa-mir-141	-1.07453	3.041065	-4.3224	2.32E-05	0.000483	0.114147
hsa-mir-200a	-1.31622	5.744183	-3.884	0.000135	0.002092	-1.5655
hsa-mir-200b	-1.29405	5.641443	-3.842	0.000159	0.002379	-1.71821
hsa-mir-183	-1.26411	10.41197	-3.74313	0.000231	0.003226	-2.07199
hsa-mir-203a	1.145735	9.21627	3.371244	0.000882	0.009873	-3.32914
hsa-mir-196a-1	-1.02604	1.572378	-3.25366	0.001316	0.013447	-3.70197
